# Dynamic integration of CXCL8 or CCL20 signaling with IL-15 or IL-32α reprograms human T-cell functional plasticity

**DOI:** 10.3389/fimmu.2026.1910983

**Published:** 2026-08-18

**Authors:** Messias de Oliveira Pacheco, Larissa Figueiredo Alves Diniz, Gabriela Alves Theodoro, Sabrina do Nascimento, Matheus Farinaro Vesco, Helder Nakaya, Luiz Vicente Rizzo, Fernanda Agostini Rocha, Patrícia Severino, Luciana Cavalheiro Marti

**Affiliations:** 1 Faculdade Israelita de Ciências da Saúde Albert Einstein, Hospital Israelita Albert Einstein - Department of Experimental Research, São Paulo, Brazil; 2 Faculdade Israelita de Ciências da Saúde Albert Einstein, Hospital Israelita Albert Einstein – Medical School, São Paulo, Brazil; 3 Medical Institute of Bioregulation, Kyushu University, Fukuoka, Japan; 4 Melanoma and Skin Tumors Sector, Plastic Surgery Discipline, Escola Paulista de Medicina/Universidade Federal de São Paulo EPM/UNIFESP), São Paulo, Brazil

**Keywords:** CCL20, cell migration and inflammation, chemokines, CXCL8, cytokines, IL-15, IL-32

## Abstract

**Introduction:**

Tissue-derived chemokines can direct immune cell migration by engaging specific receptors, thereby controlling the localization and trafficking of immune cells within tissues. However, how chemokines modulate cytokines-driven transcriptional programs in T cells remain unclear. This study investigated how CXCL8 and CCL20 influence human T cell responses to IL-15 and IL-32α.

**Methods:**

Peripheral blood T cells from health donors were evaluated for the expression and functional activity of the chemokine receptors CCR6, CXCR1, and CXCR2. Chemokine induced migration was assessed using *in vitro* migration assays. T cells were stimulated with CCL20 or CXCL8 alone or in combination with IL-15 or IL-32α, and transcriptional responses were characterized by transcriptomic profiling. Selected transcriptional findings were further evaluated through functional and protein-level assays.

**Results:**

Peripheral blood T cells expressed CCR6, CXCR1, and CXCR2, which were downregulated during *vitro* culture, but remained functionally responsive in migration assays, highlighting the influence of the microenvironmental on receptor expression. Transcriptomic profiling showed that CCL20 and CXCL8 independently reprogrammed GPCR-associated networks involved in migration, cytoskeletal dynamics, adhesion, and chemokine scavenging. IL-15 induced robust JAK/STAT-dependent inflammatory and survival programs together with CISH upregulation, indicating coordinated activation and negative-feedback regulation. In contrast, IL-32α modulated PI3K/AKT, adhesion, and NOTCH1-related pathways and was associated with increased T cell proliferation. Combined stimulation revealed context-dependent integration of these signaling programs. IL-15 plus CCL20 enhanced inflammatory and migratory signatures, whereas CXCL8 plus IL-15 produced a transcriptional profile largely dominated by IL-15. IL-32α plus CXCL8 induced cooperative programs involving vesicular remodeling, mTOR-associated metabolism, cytoskeletal organization, and cell-cycle regulation.

**Discussion:**

These findings demonstrated that chemokine and cytokine signals are integrated in a context-dependent manner to regulate T cells migration, activation, survival and proliferation. Rather than acting solely as chemoattractant, CCL20 and CXCL8 can reshape cytokine-driven transcriptional profile programs, revealing additional regulatory layer that may contribute to T cell functional plasticity within inflammatory and tumor microenvironment.

## Introduction

Tissue cells, whether in a resting state or activated by injury, infection or mutation, continuously secrete chemokines to communicate their location and physiological status to the immune system (1). The precise positioning of leukocytes within an organ or tissue is crucial for orchestrating both physiological and pathological immune responses (1). Secondary lymphoid organs also depend on chemokine gradients to guide T cell and dendritic cell trafficking, promoting their encounter and the potential initiation of an adaptive immune response (2–4).

Traditionally, the biological effects of chemokines have been interpreted primarily as a function of their local concentration and the expression of their cognate receptors. While these parameters are undoubtedly important, we propose that they alone are insufficient to predict the biological outcome of chemokine signaling. Rather, the capacity of chemokine–receptor interactions to elicit a functional response is determined by additional regulatory mechanisms, including receptor specificity, post-translational modifications such as kinase-mediated receptor phosphorylation, receptor density, and modulation by other mediators within the tissue microenvironment (5, 6).

Among these modulators, cytokines are particularly important because they can profoundly influence chemokine receptor expression, signaling competence, and downstream cellular responses (7, 8). Moreover, although chemokines signal through G protein-coupled receptors (GPCRs), which are distinct from canonical cytokine signaling pathways, extensive crosstalk exists between these systems. Consequently, effective chemokine signaling depends not only on ligand availability and receptor expression but also on the activation state of the responding cell, the surrounding cytokine milieu, receptor post-translational regulation, and the balance between conventional and atypical chemokine receptors (7). The integration of these factors ultimately determines the magnitude and quality of immune cell migration and function.

Within inflamed epithelial and tumor microenvironments, coordinated cytokine and chemokine signaling shapes immune responses. Upon activation by TNF-α, IFN-γ, or IL-32α, epithelial and tumor cells secrete CXCL8 and CCL20 (9, 10). CXCL8 drives neutrophil recruitment and endothelial activation, whereas CCL20 attracts CCR6^+^ T cells and immature dendritic cells, thereby promoting adaptive immune priming (11). Although CXCL8 and CCL20 are key regulators of immune-cell trafficking, the mechanisms governing their coordinated regulation remain incompletely understood (12, 13). In parallel, IL-15 produced by stromal, dendritic cells in several inflammatory conditions can enhance T-cell and NK-cell cytotoxicity (14), while IL-32α amplifies inflammatory circuits by regulating CXCL8 and CCL20 and sensitizing immune cells to IL-15 through NF-κB activation (15). Together, these pathways integrate chemotactic and activation cues that coordinate effector T-cell migration, functional activation, and survival.

In this study, we investigated how IL-15 and IL-32α modulate T-cell responses to the chemokines CXCL8 and CCL20, focusing primarily on transcriptional changes. Selected findings from the gene expression analysis were subsequently validated in independent functional assays, including protein expression, proliferation, and migration, as appropriate for the biological pathways identified. We hypothesized that IL-15 and IL-32α differentially modulate chemokine-induced signaling, thereby contributing to the functional plasticity of T cells in inflammatory and tumor-associated microenvironments.

## Materials and methods

### Study design

**Figure 1:**
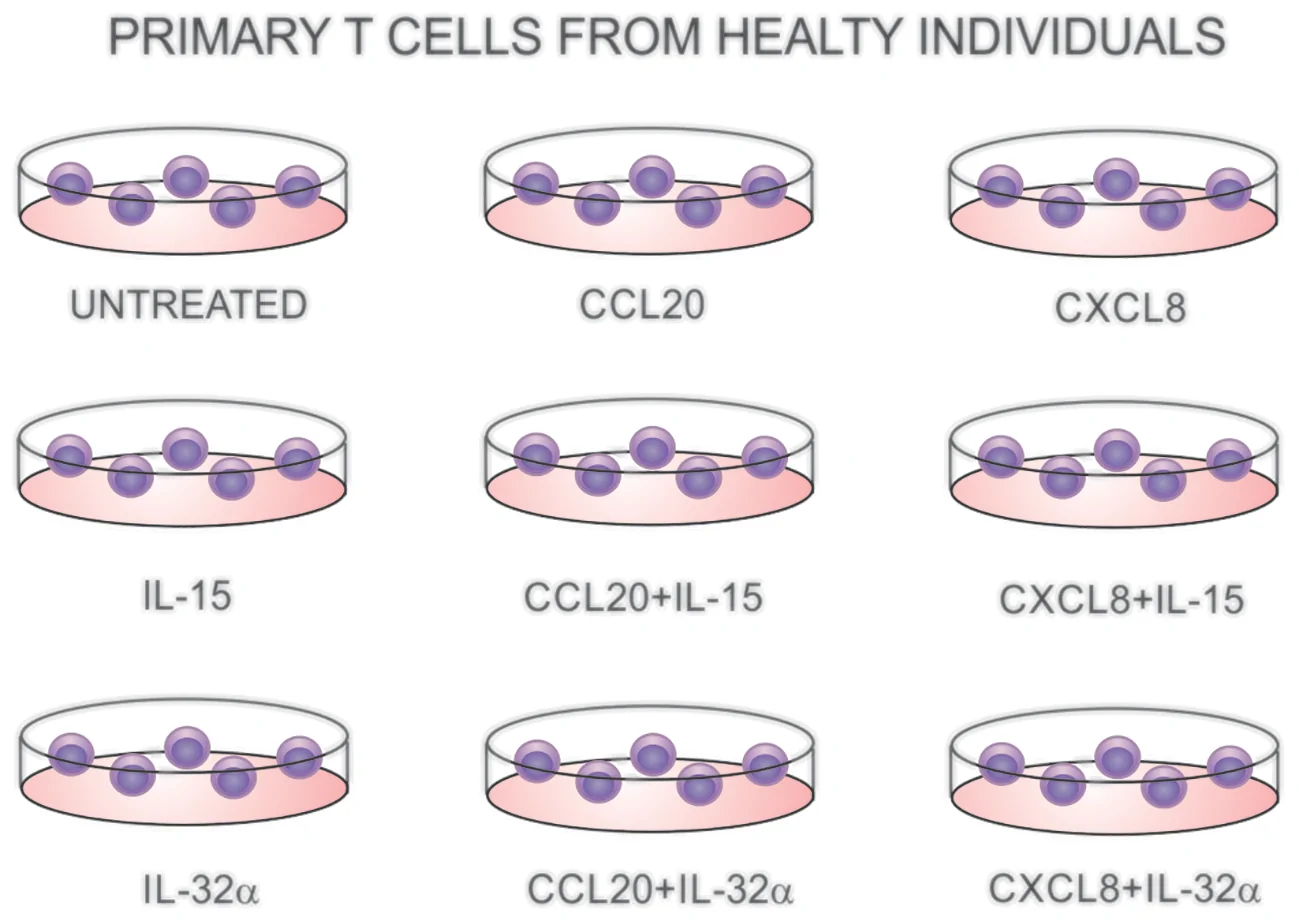
Study design - Schematic representation of each condition studied for T cells. The T cells from peripheral blood of healthy donors were obtained after mononuclear cells isolation by density gradient followed by magnetic column selection of CD3^+^ T cells. The T cells (1x10^6^/condition) were cultured in AIM-V medium supplemented with 1% L-glutamine, 1% HEPES, 1% non-essential amino acids (all from Gibco, New York, NY), 10% of AB pooled human serum (Valley Biomedical - Winchester, VA) in presence of 1ng/mL of CCL20 or CXCL8 (R&D Systems) in combination with 1000pg/mL of IL-15 or IL-32α (R&D Systems), for 18 hours. After this period, the mRNA was extracted, and cells were evaluated for gene expression by microarray. These same conditions were used for results validation tests including protein expression or proliferation assays.

The overall objective of this study was to determine the role of CXCL8 and CCL20 in combination or not with IL-15 and IL-32α signaling in T cells to elucidate mechanisms triggered by these molecules in adaptive immune cells. The cells scheme of treatments was performed as described in **Figure 1**. Outcomes of this signaling were measured by specific changes in cell gene expression profiles, phenotype and function measured by flow cytometry, and proliferation and migration assays. All experiments were repeated at least 3 times, and numbers of experimental repeats are indicated in the figure legends.

### Sample obtainment

A total of 40 peripheral blood samples were collected from healthy volunteers by venipuncture, with an average volume of 50 mL per donor. Donors were selected based on self-reported healthy status and absence of current infections prior to sample collection. Blood was transferred into EDTA-containing tubes immediately after collection. All volunteers provided written informed consent prior to enrollment, in accordance with ethical guidelines. The study protocol was approved by the Ethics Committee of Hospital Israelita Albert Einstein (Certificate of Presentation for Ethical Review – CAAE: 93808318.6.0000.0071).

### T cell separation

An aliquot of whole blood was immediately stained for lymphocyte-specific markers and chemokine receptors, as described in the following section. Peripheral blood cells were then diluted 1:1 in phosphate-buffered saline (PBS) and carefully layered over 5 mL of Ficoll-Paque with a density of 1.077 g/mL (Cytiva, UK) in a 15 mL conical tube. The samples were centrifuged at 500 × *g* for 30 minutes at 22 °C. Mononuclear cells were collected from the interface, washed, and centrifuged again at 500 × *g* for 5 minutes.

Cells were subsequently separated based on CD3 expression using magnetic selection columns (Miltenyi Biotec, Bergisch Gladbach, Germany). When required, further purification by fluorescence-activated cell sorting (FACS) was performed using a FACSAria™ Fusion cytometer (BD Biosciences). The purity of all isolated cell populations exceeded 98%.

### T cell culture

The T cells from peripheral blood of healthy donors were obtained after mononuclear cells isolation by density gradient followed by magnetic column selection of CD3 T cells as previously described. The T cells (1x10^6^/condition) were cultured in AIM-V medium supplemented with 1% L-glutamine, 1% HEPES, 1% non-essential amino acids (all from Gibco, New York, NY), 10% of AB pooled human serum (Valley Biomedical - Winchester, VA) in presence of 1ng/mL of CCL20 or CXCL8 (R&D Systems) in combination with 1ng/mL of IL-15 or IL-32α (R&D Systems), for 18 hours. After this period, the mRNA was extracted, and cells were evaluated by microarray. These same conditions were used for results validation tests including protein expression or proliferation assays.

### T cell proliferation assay

After selection, 1x10^6^ T cells per condition were labeled with CellTrace™ Violet (Invitrogen) and cultured in AIM-V medium supplemented with 1% L-glutamine, 1% HEPES, 1% non-essential amino acids (all from Gibco, New York, NY), 10% of AB pooled human serum (Valley Biomedical - Winchester, VA) either under unstimulated conditions or stimulated with phytohemagglutinin (PHA, 1 µg/mL-Sigma) in the presence or absence of IL-32α at concentrations of 1ng/mL and 10ng/mL. The cultures were maintained for three days. Following incubation, cells were stained with anti-CD3-FITC (clone: HIT3a) from BD Pharmingen and analyzed by flow cytometry to assess violet (BV421) fluorescence intensity to determine their proliferation index.

The experimental conditions were optimized according to the biological endpoint evaluated. For the proliferation assay, cytokine concentrations of 1 and 10 ng/mL were used because functional responses such as cell proliferation often require higher cytokine concentrations than those needed to induce transcriptional changes. In addition, proliferation assays are conventionally performed over 72 hours, rather than 18 hours, to allow sufficient time for measurable cell division.

Proliferation analyses were performed using the FlowJo software (BD Biosciences), employing the Cell Proliferation module to determine the proliferation index (PI). The PI reported by FlowJo (using the Proliferation Platform for CellTrace) is derived from the deconvolution of the fluorescence peaks into successive cell generations. After fitting the proliferation peaks, FlowJo estimates how many original precursor cells gave rise to each generation producing a number, which is named by the system as proliferation index.

### T cell migration assay

After selection, T cells (1 × 10^6^) were seeded into the upper chamber of 3.0-µm pore transwell inserts (Corning, NY). The lower chamber contained methylcellulose-based semi-solid culture medium (Stem cells Technologies, Canada), either without supplementation or supplemented with the indicated reagents, such as CCL20 (10 or 100 ng/mL; R&D Systems) or CXCL8 (100 ng/mL; R&D Systems). For T cells were incubated at 37 °C in 5% CO_2_ for 18h. Migration was assessed by quantifying T cells that traversed to the lower chamber, for CXCL8 migration total T cells (CD3) were used, while for CCL20 migration, sorted CD3:CCR6 negative and positive were used. Migrated cells were collected and analyzed by flow cytometry following staining with CD3 (for CXCL8 conditions) or CD3 plus CCR6 (for CCL20 conditions). Cell counts were determined by acquiring the entire recovered volume, with all recorded events included in the analysis.

The migration assay required higher chemokine concentrations in the lower chamber of the Transwell inserts to establish a stable chemotactic gradient capable of promoting directional T-cell migration.

### Flow cytometry assays

Cells were stained with the following panel of commercial antibodies: CD45-Alexa 700 (clone HI30), CD3-APC-H7 (clone SK7), CD4-BV510 (clone L120), CD8-PE-CF594 (clone RPA-T8), CXCR1-PerCP-Cy5.5 (clone 5A12), CXCR2-FITC (clone 5E8-CXCR2), CCR2-APC (clone 48607), CCR6-PE-Cy7 (clone 11A9), CCR10-PE (clone 1B5), CXCR7/ACKR3-BV421 (clone 10D1), CCRL1/ACKR4-BV605 (clone 13E11), CD54/ICAM-1-APC (clone HA58), CD102/ICAM-2-PE (clone CBR-IC2/2), CD62P/P-selectin-PerCP-Cy5.5 (clone AK-4), CD62L/L-selectin-BV421 (clone DREG-56), and D6/ACKR2-FITC (clone 196124). Antibodies were obtained from BD Biosciences, BD Pharmingen, BD Horizon, BioLegend, and R&D Systems.

Two types of samples were analyzed: cultured cells and whole blood. For whole-blood samples, red blood cells were lysed prior to staining, using Excellyse Live (Exbio Praha, Czech Republic). Control tubes were included for each staining condition, and fluorescence-minus-one (FMO) controls were prepared for chemokine receptors, adhesion molecules, and selectins.

After staining, cells were incubated for 20 minutes in the dark, followed by washing with buffer solution (phosphate-buffered saline supplemented with human albumin and sodium azide). Samples were centrifuged at 500×*g* for 10 minutes, the supernatant was discarded, and 300 µL of the same buffer solution was added prior to acquisition.

Data acquisition was performed on an LSR II Fortessa flow cytometer (BD Biosciences), and data were analyzed using FlowJo software (BD Biosciences). All analyses included doublet-exclusion gating to ensure accuracy. The gate strategy for all analysis performed was included as **Supplementary Figure 1**.

### *NOTCH1* expression of human T cells

For *NOTCH-1* expression, experimental conditions included three biological replicates of T cells cultured with no stimuli, stimulated with IL-32α (1 and 10ng) or PHA. After cell culture, total RNA was isolated using RNeasy^®^ Mini kit (Qiagen) following the manufacturer’s instructions. RNA quality and quantity were evaluated by spectrometry using the NanoDrop equipment (Thermo Scientific). Reverse Transcription of RNA (1 µg) to cDNA was performed using QuantiTec Reverse Transcription kit (Qiagen). qPCR was performed using 100 ng cDNA and the QuantiFast^®^ SYBR^®^ Green PCR Kit (Qiagen) in the QuantStudio 6 Flex Real-time PCR System (Thermo Fisher Scientific). The amplification program consisted of an initial denaturation at 95 °C for 1 minute followed by 40 cycles at 95 °C for 10 seconds, and the annealing and extension phase took place at 60 °C for 30 seconds. The qRT-PCR assays were performed in triplicate; Ct values were normalized using the *RPLA13a* gene and the relative gene expression was calculated using the CT comparative method (2^-ΔΔ Ct^). The sequence of gene-specific primers for *RPL13a* sense GAGAAGGCTGGGGCTCA and antisense GTCCTTCCACGATACCAAA (400nM) and for *NOTCH-1* sense AGGCAATCCGAGGACTAT and antisense AGAACGCACTCGTTGATG (200nM).

### Microarray-based transcriptomic profiling of human T cells

Gene expression profiling was performed in three independent T-cell samples treated with different combinations of cytokines and chemokines, as described in **Figure 1**, totaling nine experimental conditions per sample. After 18hs of cell culture, total RNA was extracted from human samples using a RNeasy^®^ Mini kit (Qiagen) following the manufacturer’s instructions. RNA concentration and purity were assessed using spectrophotometry (A260/A280 and A260/A230 ratios), and RNA integrity was evaluated using capillary electrophoresis.

Genome-wide gene expression profiling was performed using the Human Clariom™ S Array (Thermo Fisher Scientific), which interrogates all known and well-annotated exons, including protein-coding transcripts and selected non-coding RNAs. For each sample, 100 to 250 ng of total RNA was processed using the GeneChip WT PLUS Reagent Kit according to the manufacturer’s instructions. Following hybridization, signals were detected with the GeneChip Scanner 3000 7G, and raw data were acquired using the Affymetrix GeneChip Command Console software. Data quality control, normalization, and differential expression analyses were conducted using the Transcriptome Analysis Console software (version 4.0.1) (Thermo Fisher Scientific).

Briefly, total RNA was converted to double-stranded cDNA, followed by *in vitro* transcription to generate complementary RNA (cRNA). The cRNA was subsequently reverse-transcribed to produce single-stranded sense cDNA, which was fragmented and biotin-labeled. Labeled cDNA was hybridized to Clariom S Human Arrays at 45 °C for 16 hours in a hybridization oven with constant rotation. After hybridization, arrays were washed and stained using the GeneChip Fluidics Station and scanned using a GeneChip Scanner to generate CEL files.

### Microarray data processing and analysis

Raw intensity data (CEL files) generated from the Clariom™ S Human Arrays were imported into the Transcriptome Analysis Console (TAC) software (Thermo Fisher Scientific) for preprocessing and downstream analysis. Background correction, normalization, and probe-level summarization were performed using the Robust Multi-array Average (RMA) algorithm implemented within TAC, including log2 transformation of expression values.

Quality control was assessed using standard metrics provided by the software, including signal intensity distributions, principal component analysis (PCA), and sample correlation plots, to evaluate overall data quality and identify potential technical outliers.

Gene-level expression estimates were generated using the latest Clariom™ S Human array annotation files available at the time of analysis. Differential gene expression analysis was performed within TAC using linear modeling approaches based on empirical Bayes statistics. Statistical significance was determined by calculating p-values for each transcript, followed by correction for multiple hypothesis testing using the Benjamini–Hochberg false discovery rate (FDR) method. Transcripts with an adjusted p-value < 0.05 and an absolute log2 fold change ≥ 1 were considered differentially expressed.

Gene expression changes are presented as fold changes. Hierarchical clustering of differentially expressed genes was visualized as heat maps using Euclidean distance with Ward or complete linkage methods. Gene Ontology and KEGG pathway enrichment analyses were conducted using DAVID Bioinformatics Resources v6.8, with significance defined as p ≤ 0.05 after Benjamini–Hochberg false discovery rate correction. Raw and processed data are available in the Gene Expression Omnibus under accession number GSE318146.

### Statistics

Experimental results were analyzed for significance using one-way analysis of variance (ANOVA) with Tukey’s multiple comparisons test (for multiple groups) or unpaired Student’s t test. Statistical analyses were performed using GraphPad Prism, version 7.02. The p values where considered significant when p ¾ 0.05, all data are represented as means ± SEM.1910983.

## Results

### Baseline expression of CCR6, CXCR1, and CXCR2 on human T cells from peripheral blood is downregulated during *in vitro* culture without stimulation

**Figure 2:**
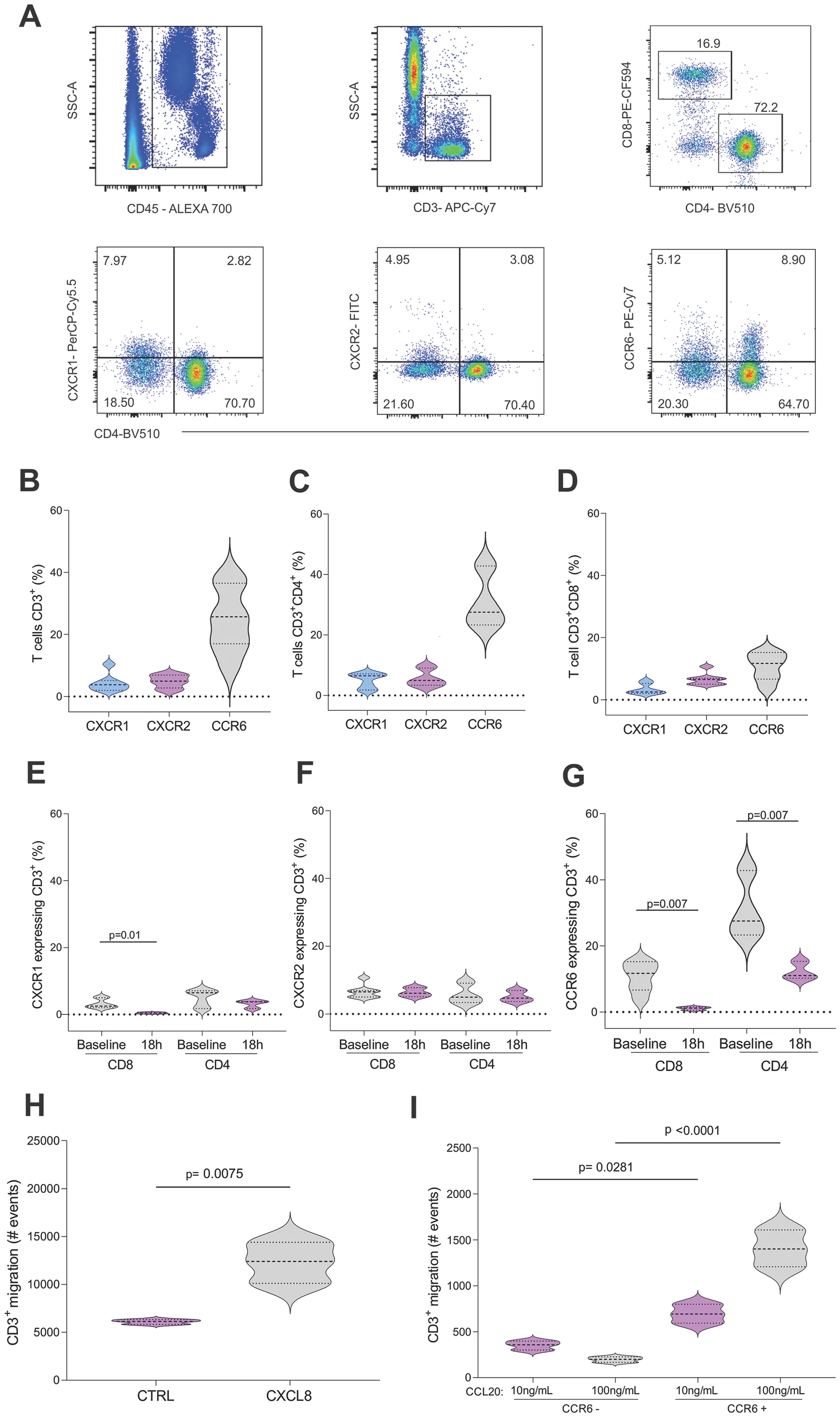
The expression of CCL20 and CXCL8 receptors is downregulated in culture condition. **(A)** Flow cytometry gate strategy, **(B–D)** Expression of CXCR1, CXCR2 and CCR6 evaluated by flow cytometry in freshly isolated T cells (n=7), **(E–G)** Flow cytometric analysis of CXCR1, CXCR2, and CCR6 expression on positively selected CD3^+^ T cells immediately after magnetic isolation (baseline) and following 18h of culture in AIM-V medium, which is specifically formulated to support T-cell culture (n=7), **(H)** migration of CD3 T cells to CXCL8 gradient (n=3), **(I)** migration of CCR6- and CCR6+ T cells (CD3) to CCL20 gradients (n=3).

Peripheral blood T cells, and CD4 or CD8 subsets, were analyzed for the baseline expression of CCL20 receptor (CCR6) and CXCL8 receptors (CXCR1 and CXCR2). After the T cells selection, the flow cytometric analysis revealed as baseline a low surface levels of CXCR1 (4.92 ± 2.46% in CD4 and 3.35 ± 1.67% in CD8 T cells) and CXCR2 (5.59 ± 2.78% and 6.56 ± 2.03%, respectively), whereas CCR6 was more prominently expressed (30.91 ± 8.67% in CD4 and 10.53 ± 4.43% in CD8 T cells) (**Figures 2A–D**).

After 18 hours of *in vitro* culture in AIM-V medium (specific T cell culture medium) and in the absence of stimulation, T cells exhibited a reduction in basal chemokine receptor expression compared to a previous baseline analysis (immediately after CD3 selection), with a significant decline in CXCR1 (*p =* 0.010) and CCR6 (*p =* 0.0070) in CD8^+^ T cells, and CCR6 (*p =* 0.0070) in CD4^+^ T cells (**Figures 2E–G**).

To determine whether the reduced surface expression observed after *in vitro* culture affected receptor functionality, we performed transwell migration assays, quantifying migrated cells by event counts in the lower chamber. Despite the decline in receptor expression, T cells retained functional responsiveness to their cognate ligands. CXCR1/2 signaling remained intact, as significantly more T cells migrated toward CXCL8 compared with control conditions (12,302 ± 2,138 vs. 6,097 ± 253; *p =* 0.0075) (**Figure 2H**).

Similarly, to assess CCR6 functionality, T cells were sorted into CCR6^+^ and CCR6⁻ populations. CCR6^+^ which demonstrated markedly enhanced migration toward CCL20 at both tested concentrations (10 ng/mL: 695 ± 104; 100 ng/mL: 1,405 ± 200) compared with CCR6⁻ cells (352 ± 49 and 197 ± 30, respectively; *p = 0*.0281 and *p* < 0.0001) (**Figure 2I**).

### Transcriptional profile of T cells in response to CCL20, CXCL8, IL-32α and IL-15 treatments

**Figure 3:**
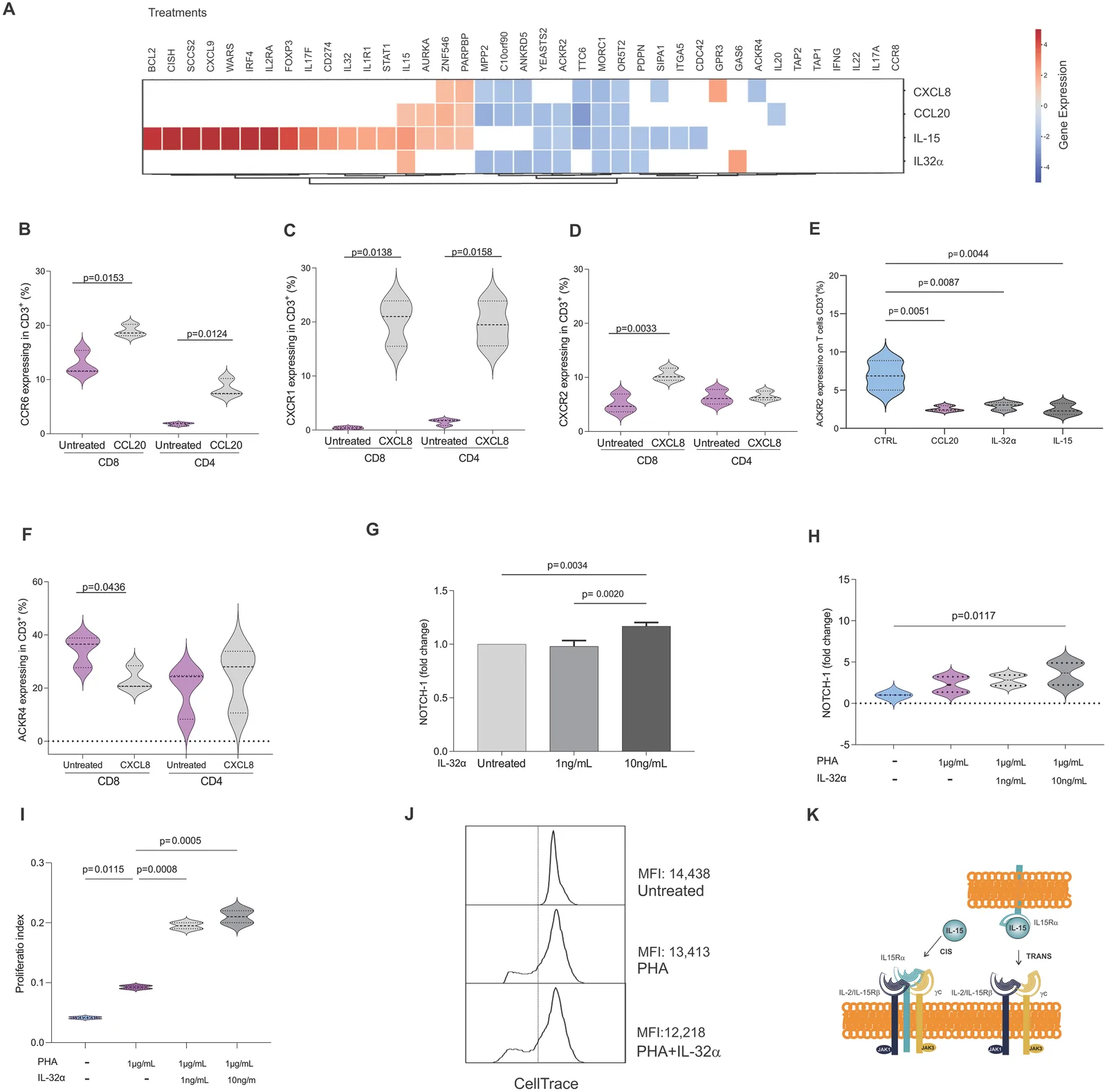
T cells transcriptional profile regulated by CCL20, CXCL8, IL-15 and IL-32α. Cells from peripheral blood of healthy donors were obtained after mononuclear cells isolation by density gradient followed by magnetic column selection of CD3 T cells. The T cells (1x10^6^/condition) were cultured in AIM-V supplemented medium (control group) and in the presence of CCL20 for 18h. **(A)** Genes up and down regulated with high fold change or genes that are part of the same pathway in T cells after individual treatments (n=3), **(B)** CCR6 expression in untreated CD8 and CD4 T cells or after treatment with CCL20 (n=7), **(C)** CXCR1 and **(D)** CXCR2 expression in untreated CD8 and CD4 T cells or after treatment with CXCL8 (n=7), **(E)** ACKR2 expression on T cells CD3+ untreated and treated with CCL20, IL-32α and IL-15, **(F)** ACKR4 expression in CD3CD8 and CD3CD4 untreated and treated with CXCL8, **(G)** NOTCH-1 expression on untreated or T cells treated with 1ng/mL or 10ng/mL of IL-32α, **(H)** NOTCH-1 expression on untreated or IL-32α-treated and PHA-activated T cells, **(I)** Proliferation of IL-32α-treated and PHA-activated T cells, **(J)** Representative flow cytometry plots of proliferative profile of activated T cells, (n=3). **(K)** Schematic representation of IL-15 multi-subunit complex.

The transcriptomic profile of T cells cultured in the presence or absence of CCL20, CXCL8, IL-15, and IL-32α was compared (**Figure 3A**; **Supplementary Tables 1**, **S2**), revealing stimulus-specific transcriptional programs involving chemokine signaling, cytoskeletal dynamics, adhesion, and immune regulation.

First, we evaluate the chemokines influence on their receptor, CCL20 significantly increased CCR6 protein expression on both CD8^+^ T cells (*p* = 0.0124) and CD4^+^ (*p* = 0.0153) (**Figure 3B**), demonstrating that receptor expression remains dynamically responsive to environmental cues. Notably, CXCL8 also significantly upregulated its receptors, increasing CXCR1 expression on CD8^+^ T cells and CD4^+^ (*p* = 0.0138 and *p* = 0.0158) and CXCR2 expression on CD8^+^ T cells (*p* = 0.0033) (**Figures 3C, D**; **Supplementary Figures 2A–C**).

Upon CCL20 stimulation, among the differentially expressed genes, *interferon alpha 14 (IFNA14)* and *interleukin 20 (IL20)* were significantly downregulated (*p* = 0.0394), suggesting a transient attenuation of antiviral immune pathways that may accompany early CCL20-driven T cell activation. KEGG pathway enrichment analysis demonstrated that CCL20 induces broad reprogramming of gene networks associated with G protein–coupled receptor (GPCR) signaling, with downstream effects on cellular metabolism, cytoskeletal remodeling, and adhesion (**Figure 3A**; **Supplementary Tables 1A**, **S2A**).

Consistently, as expected genes associated with cytoskeletal organization were modulated. The small GTPase *ras-related C3 botulinum toxin substrate 3 (RAC3)* was upregulated (*p* = 0.0223), whereas its downstream effectors, *p21-activated kinase 3 (PAK3)* and *CDC42 effector protein 3 (CDC42EP3)*, were downregulated (*p* = 0.0435 and p = 0.0303, respectively). This transcriptional configuration suggests a cytoskeletal state favoring dynamic actin turnover rather than stable front–rear polarity, as persistent directional migration typically requires coordinated activation of downstream effectors such as PAK kinases and CDC42. In parallel, *integrin beta 6 (ITGB6)* was upregulated (p = 0.0357), along with an increase in *interleukin 15 (IL15)* expression (*p* = 0.0490), possibly reflecting transcriptional programs linked to cytoskeletal rearrangement. Additionally, the atypical chemokine receptor *ACKR2 (atypical chemokine receptor 2)* was downregulated (*p* = 0.0243), whereas *C-X-C motif chemokine ligand 13 (CXCL13)* was upregulated (*p* = 0.0375) suggesting coordinated modulation that may enhance chemokine availability through reduced scavenging and increased ligand production (**Supplementary Tables 1A, S2A**). The downregulation of ACKR2 in T cells CD3^+^ was also confirmed at protein level (*p* = 0.0051) (**Figure 3E**).

CXCL8 stimulation similarly impacted GPCR-related signaling pathways. Significant downregulation was observed in *Rho GTPase activating protein 23 (ARHGAP23)* (*p* = 0.0065), *Ras and Rab interactor 1 (RIN1)* (*p* = 0.0438), and *phospholipase A2 group IVD (PLA2G4D)* (*p* = 0.0031), genes involved in second-messenger generation. In contrast, *G protein-coupled receptor 3 (GPR3)* was upregulated (*p* = 0.0072), suggesting compensatory activation within GPCR signaling pathways. Furthermore, *ACKR4 (atypical chemokine receptor 4)* expression was reduced (*p* = 0.0436), which may enhance inflammatory persistence by limiting scavenging of *C-C motif chemokine ligands CCL19, CCL21, and CCL25*, despite the absence of direct CXCL8 binding. Protein-level analysis confirmed this modulation (**Figure 3F**), demonstrating a more pronounced CXCL8-driven reduction of ACKR4 in CD8^+^ compared to CD4^+^ T cells. Together, these data suggest that CXCL8 modulates not only canonical receptor expression but also downstream GPCR signaling and chemokine-scavenging pathways, potentially favoring a T-cell phenotype more responsive to migratory cues and sustained inflammatory stimuli within cytokine-rich microenvironments (**Figure 3A**; **Supplementary Table 1B**).

In parallel, KEGG pathway enrichment analysis demonstrated that IL-32α stimulation upregulated pathways associated with positive regulation of cell adhesion mediated by integrin (*p* = 0.0096), cell adhesion (*p* = 0.0879), activation of protein kinase B activity (PKB/AKT) (*p* = 0.0363), and positive regulation of immune responses (*p* = 0.0998). Correspondingly, IL-32α increased the expression of genes involved in adhesion and cytoskeletal remodeling, including *growth arrest specific 6 (GAS6)*, *ras-related C3 botulinum toxin substrate 3 (RAC3)*, *neurotrophic receptor tyrosine kinase 3 (NTRK3)*, and *cadherin 4 (CDH4)*, while *fibronectin leucine rich transmembrane protein 3 (FLRT3)*, *podoplanin (PDPN)*, *papilin proteoglycan-like sulfated glycoprotein (PAPLN)*, and *fibrillin 3 (FBN3)* were downregulated (**Supplementary Table 2B**).

Notably, T cells under treatment of IL-32α and not previously described upregulated *protocadherin beta 6 (PCDHB6)* expression. This non-classical cadherin undergoes γ-secretase–dependent processing, releasing intracellular fragments that regulate gene expression, including *Notch homolog 1 (NOTCH1)*. Consistent with this mechanism and not previously described, the IL-32α alone increased NOTCH1 expression, and its combination with phytohemagglutinin (PHA) induced its synergistic upregulation (**Figures 3G, H**; **Supplementary Table 1C**), indicating cooperative signaling between cytokine exposure and mitogenic stimulation.

Furthermore, IL-32α activated the phosphatidylinositol 3-kinase/protein kinase B (PI3K/AKT) pathway, a central regulator of proliferation and survival. Functionally, IL-32α significantly enhanced PHA-induced T-cell proliferation (**Figures 3I, J**; **Supplementary Table 2C**), confirming its pro-survival and co-stimulatory effects. These findings are consistent with previous reports linking IL-32α to enhanced transforming growth factor beta (TGF-β) and IL-15 signaling, pathways critical for immune homeostasis and long-term T-cell maintenance. Unlike other IL-32 isoforms, IL-32α is primarily associated with regulatory and survival functions rather than overt pro-inflammatory activity. The treatment of T cells with IL-32α downregulated the genic expression of ACKR2 (*p* = 0.0018), which also was confirmed at protein level (*p* = 0.0087) (**Figure 3E**). Although IL-32α is classified as a cytokine, no canonical receptor has been identified and the up-regulated genes observed here do not confirm a classical cytokine signaling.

IL-15 signals through a receptor complex composed of IL-15Rα (CD215), IL-2/IL-15Rβ (CD122), and the common γ-chain (CD132), which was not investigated in this study. This is a well-characterized signaling pathway that promotes proliferation, survival, and differentiation via activation of the JAK/STAT pathway (**Figure 3K**).

IL-15 stimulation induced robust activation of multiple signaling pathways and upregulation of key pro-inflammatory mediators, including *tumor necrosis factor alpha (TNF-α)* (p = 0.0019) and *interleukin 17F (IL17F)* (*p* = 0.0387), as well as cytokine receptors *interleukin 2 receptor alpha (IL2Rα/CD25)* (*p* < 0.001) and *interleukin 1 receptor type 1 (IL1R1)* (*p* = 0.0376). Consistent with activation of the Janus kinase/signal transducer and activator of transcription (JAK/STAT) pathway, *signal transducer and activator of transcription 1 (STAT1)* (*p* = 0.0011) and *interferon regulatory factor 4 (IRF4)* (*p* < 0.001) were markedly upregulated. The induction of *cytokine inducible SH2-containing protein (CISH)* (*p* < 0.001) indicates the establishment of a negative-feedback mechanism regulating cytokine signaling intensity. Additional genes, including *forkhead box P3 (FOXP3)* (*p* = 0.0002), *cluster of differentiation 274 (CD274/PD-L1)* (*p* = 0.0113), and *B-cell lymphoma 2 (BCL2)* (*p* = 0.0012), were also upregulated, collectively reflecting a transcriptional state integrating activation, regulatory control, and survival. BCL2 upregulation supports enhanced T-cell longevity (**Supplementary Tables 1D**, **S2D**). In addition, IL-15 also downregulated the ACKR2 gene expression (*p =* 0.0214), which was also confirmed at protein level (*p =* 0.0044) (**Figure 3E**).

### The presence of CCL20 or CXCL8 in combination with IL-15 or IL-32α induce significant alterations in the T cells profile

**Figure 4:**
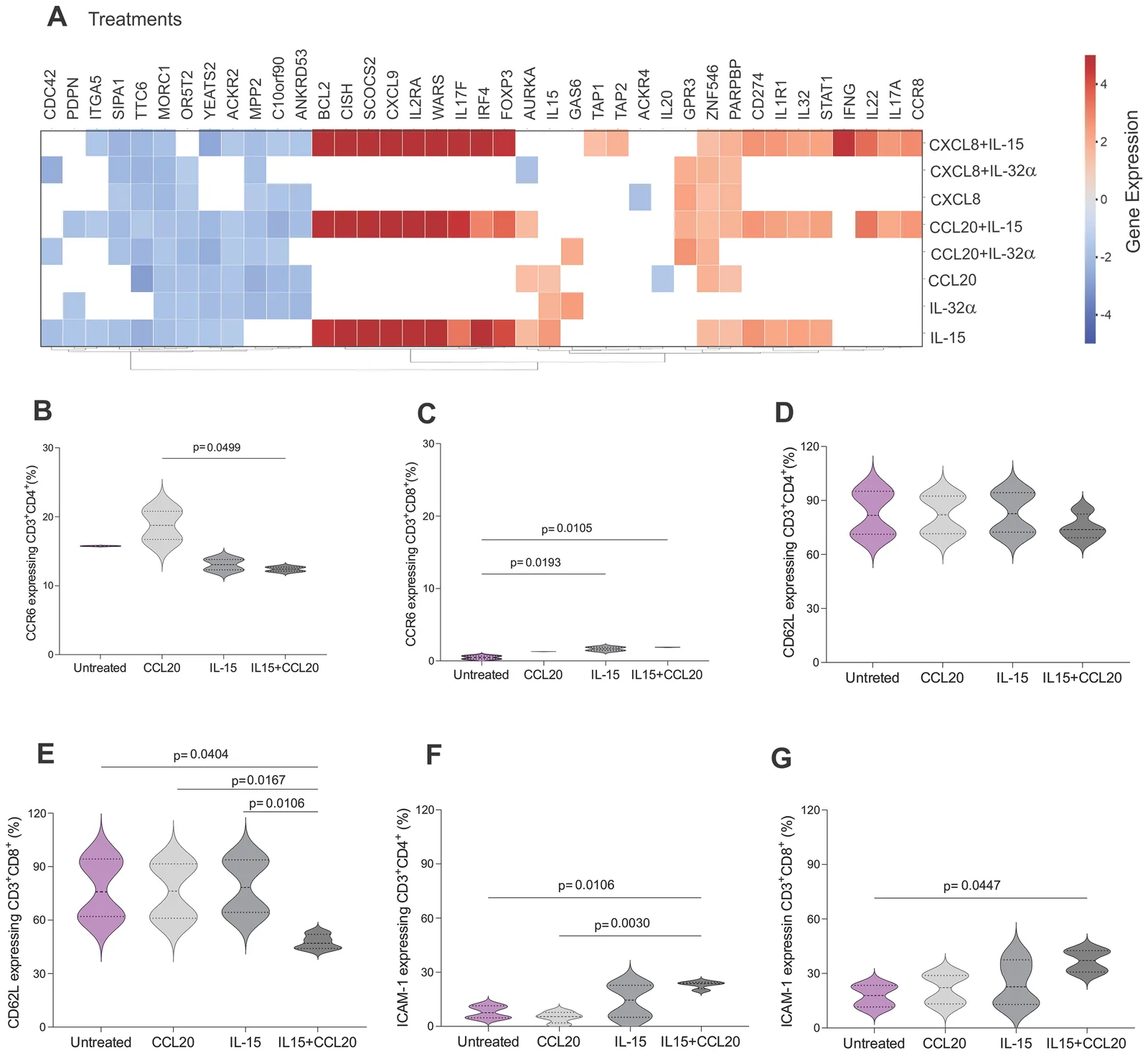
T cells transcriptional profile regulated by a combination of CCL20 and CXCL8 with IL-15 or IL-32α. Cells from peripheral blood of healthy donors were obtained after mononuclear cells isolation by density gradient followed by magnetic column selection of CD3^+^ T cells. The T cells (1x10^6^/condition) were cultured in AIM-V supplemented medium (untreated group) and in the presence of CCL20 in combination with IL-15 or IL-32α for 18h. **(A)** Heat map of T cells gene expressions treated with chemokines and cytokines individually and in combination (n=3). **(B)** CCR6 expression in CD4 T cells or **(C)** CD8 T cells under the treatment of CCL20, IL-15 or CCL20+IL-15, **(D)** CD62L expression CD4 T cells and, **(E)** Flow cytometry plots demonstrating the CD62L expressing CD3^+^CD8^+^ T cells under the treatment of CCL20, IL-15 or CCL20+IL-15, **(F)** CD54 expression on CD4 T cells or **(G)** CD8 T cells under the treatment of CCL20, IL-15 or CCL20+IL-15.

Transcriptomic profiles of T cells were also assessed in combinatory treatments of chemokines and cytokines (**Figure 4A**; **Supplementary Tables 1**, **S2**).

### Transcriptional profile of T cells treated with CCL20 in combination with IL-15

T cells were stimulated with the combination of CCL20 and IL-15, which induced a transcriptional program that extended beyond the effects observed with either stimulus alone, revealing a clear synergistic interaction. KEGG pathway enrichment analysis demonstrated significant overrepresentation of pathways related to inflammatory response (*p* = 0.0042), cell-cell signaling (*p* = 0.0244), response to cytokine (*p* = 0.0054), chemokine-mediated signaling (*p=*0.019), and positive regulation of cell migration (*p=*0.049) (**Supplementary Tables 1E**, **S2E**). Consistent with this enhanced activation state, co-stimulated cells exhibited increased expression of IL-17A and IL-17F, indicating engagement of type 17 inflammatory programs.

Among IL-17 family members, IL-17A is the most extensively characterized cytokine, although key aspects of its regulation and context-dependent functions remain incompletely defined (16). Differentiation of IL-17–producing T cells typically depends on IL-23 and the transcription factor RORγt and is strongly associated with expression of the chemokine receptor CCR6, whose exclusive ligand is CCL20 (17). The concurrent presence of IL-15 and CCL20 therefore establishes a mechanistic framework in which cytokine-driven survival and activation signals converge with CCR6 mediated chemokine sensing, collectively favoring IL-17A pathway activation.

To determine whether these transcriptional changes were accompanied by modulation of CCR6 surface expression, T cells were exposed *in vitro* for 18 hours to CCL20, IL-15, or their combination. Neither cytokine alone significantly altered CCR6 expression in CD4 T cells relative to control conditions, although IL-15 plus CCL20 reduced CCR6 expression (12.4 ± 0.4%) compared with CCL20 alone (18.8 ± 2.9%) *p=*0.0499 (**Figure 4B**). In contrast, co-stimulation with CCL20 plus IL-15 resulted in a significant upregulation of CCR6 in CD8 T cells (1.9 ± 0.01%) compared to absence of treatment (0.5 ± 0.3%) *p=*0.0105 (**Figure 4C**), suggesting that IL-15 enhances chemokine responsiveness specifically within cytotoxic subsets. These findings indicate that IL-15 and CCL20 act cooperatively to potentiate interconnected signaling networks governing inflammation and migration, resulting in a broader and more robust activation profile than that elicited by either cytokine alone.

To functionally validate these transcriptional signatures, we assessed the surface expression of molecules involved in T-cell trafficking and adhesion, including the selectin CD62L (L-selectin) and the adhesion molecules ICAM-1 and ICAM-2. Co-stimulation with IL-15 plus CCL20 led to a marked reduction of CD62L expression in CD8 T cells (47.7 ± 4.2%) compared to the absence of treatment (77.4 ± 18%) *p=*0.0404 (**Figure 4E**) and a concomitant increase in ICAM-1 expression in both CD4 and CD8 (23.1 ± 2.0% and 36.8 ± 6.3% respectively) compared to absence of treatment (7.9 ± 3.6% and 17.6 ± 6.2% respectively) *p=*0.0106 and *p=*0.0447 (**Figures 4F, G**; **Supplementary Figure 3**). These changes were observed exclusively under combined stimulation and were not induced by either cytokine alone. The phenotype was particularly pronounced in CD8 T cells, further supporting selective activation and migratory priming of effector subsets.

### Transcriptional profile of T cells treated with CCL20 in combination with IL-32α

A parallel analysis of IL-32α–based combinations revealed both overlapping and distinct cooperative effects. Co-treatment with IL-32α and CCL20 reproduced several adhesion-related transcriptional responses observed with the individual stimuli but markedly amplified the expression of genes involved in focal adhesion formation and cytoskeletal remodeling. Upregulated transcripts included G protein–coupled receptor 3 (*GPR3*), actin-associated protein (*CAP2*), cadherin 4 (*CDH4*), dynein cytoplasmic 1 intermediate chain 1 (*DYNC1I1*), and the Rho guanine nucleotide exchange factor *ARHGEF3* (**Figure 4A**). This cooperative transcriptional profile suggests that IL-32α and CCL20 jointly reinforce migratory programs through both integrin-dependent and integrin-independent mechanisms, potentially facilitating the assembly of adhesion structures required for efficient T-cell movement (**Supplementary Tables 1F**, **S2F**).

### Transcriptional profile of T cells treated with CXCL8 in combination with IL-15 or IL-32α

In contrast, combinations involving CXCL8 displayed a distinct pattern of interaction. When CXCL8 was combined with IL-15, the resulting transcriptional profile closely resembled that induced by IL-15 alone, indicating dominance of IL-15–driven signaling and little evidence of synergistic interaction (**Figure 4A**; **Supplementary Tables 1G**, **S2G**).

Conversely, co-stimulation with IL-32α and CXCL8 generated a unique transcriptional landscape characterized by enrichment of pathways related to toxin transport, cell-cycle regulation, and intracellular signal transduction (**Figure 4A**; **Supplementary Tables 1H**, **S2H**).

Within the toxin transport module, expression of Vacuolar Protein Sorting–Associated Protein 11 (VPS11), a core component of the CORVET and HOPS complexes involved in endolysosomal tethering and trafficking, was reduced. In contrast, Dynamin-1 (DNM1), encoding a motor GTPase essential for vesicle scission and microtubule-dependent transport, was upregulated, suggesting enhanced vesicular dynamics. Cell-cycle–related genes exhibited opposing regulation at the G_2_/M checkpoint: Checkpoint kinase 2 (*CHEK2*), which enforces mitotic arrest in response to DNA damage, was downregulated, whereas Aurora kinase A (*AURKA*), a key regulator of mitotic spindle assembly and cell-cycle progression, was upregulated. This pattern aligns with the proliferative bias previously associated with IL-15–driven signaling (**Supplementary Table 1H**).

Signal transduction pathways were also reshaped by IL-32α and CXCL8 co-stimulation. TIR domain–containing adaptor molecule 1 (*TICAM1*), a critical mediator of Toll-like receptor 3 signaling and interferon-β induction, was downregulated, potentially dampening antiviral and NF-κB–dependent inflammatory responses. Conversely, repression of Nitrogen Permease Regulator–Like 3 (*NPRL3*), a negative regulator of mTORC1 activity, is consistent with enhanced metabolic fitness and pro-survival signaling (**Supplementary Table 2H**).

## Discussion

Chemokine and cytokine networks can influence complementary cellular programs that regulate T cell activation, migration, and persistence (18). In our study, IL-15, IL-32α, CCL20, and CXCL8 exhibited context-dependent transcriptional effects. Certain combinations, such as IL-15 with CXCL8, resulted in a reinforced IL-15 dominant signaling, whereas others, particularly IL-32α with CXCL8, induced a distinct cooperative transcriptional profile involving vesicular remodeling, mTOR-associated metabolic activity, and cytoskeletal organization. These results indicate that cytokine and chemokine interactions can either reinforce shared signaling pathways or reprogram distinct transcriptional modules, illustrating the plasticity of T cells responses under complex inflammatory conditions and the dynamic equilibrium between activation, regulation, and tissue retention that characterizes immune homeostasis and tumor microenvironments (19–22).

This study demonstrated that, in freshly isolated T cells, approximately 30% of CD4^+^ and 10% of CD8^+^ T cells expressed CCR6, supporting the concept that this receptor is restricted to distinct T-cell subsets with selective migratory responsiveness to CCL20. Previous studies have shown that CCR6 is preferentially expressed by effector and memory T cells (17, 23), as well as by Th17 and regulatory T (Treg) cells, enabling their recruitment to inflammatory and tumor sites through the CCL20-CCR6 axis (19, 22, 24).

In the tumor microenvironment, this axis has been primarily associated with the recruitment of regulatory T cells (Tregs). In nasopharyngeal carcinoma, CCR6 is predominantly expressed by FOXP3^+^ Tregs (25), whereas in colorectal cancer, blockade of CCL20 abolished the Tregs tumor-driven recruitment mediated by CCL20 secretion (26). These findings highlight the dual immunological role of the CCL20–CCR6 axis, which can influence both pro-inflammatory and immunosuppressive immune responses depending on the cellular and disease context.

Interestingly, although CCR6 surface expression decreased during *in vitro* culture, T cells remained functionally competent and retained their capacity to undergo CCL20-mediated chemotaxis, suggesting that reduced receptor expression does not necessarily compromise migratory responsiveness. Furthermore, CCL20 stimulation not only increased CCR6 expression on CD8^+^ T cells but also altered the transcriptional profile of genes involved in G protein-coupled receptor (GPCR)-mediated signaling pathways that regulate cell migration and adhesion. These findings are consistent with studies demonstrating that chemokine receptor signaling activates small GTPases, particularly Rap1, which promotes talin-dependent integrin activation, cytoskeletal remodeling, and directional T-cell migration (27, 28). Together, these observations suggest that CCL20-mediated transcriptional reprogramming may reinforce the molecular machinery required for efficient T-cell trafficking, even in the presence of reduced surface CCR6 expression.

The combination of CCL20 and IL-15 produced a transcriptional profile that was predominantly driven by IL-15 signaling. Notably, however, this combination also induces a distinct set of uniquely regulated genes, including *IL17A, ICAM1*, and *ITGA4*, suggesting a functional crosstalk in which chemokine-driven migratory cues integrate with cytokine-mediated adhesion pathways. This coordinate response indicates that CCL20 and IL-15 can act synergically to promote cellular motility and stable tissue engagement (19, 22, 29, 30).

This interpretation is consistent with previous studies demonstrating cooperative signaling networks that integrate inflammatory cytokines and chemokines to regulate leukocyte migration and their tissue localization. For example, IL-17A and myostatin have been shown to synergistically enhance CCL20 expression in fibroblast-like stromal cells through SMAD-dependent transcriptional mechanisms, illustrating how cytokine-mediated signals can amplify chemokine-driven cellular recruitment. These findings suggest that the IL-15–CCL20 axis establishes a broader transcriptional program that coordinates directional migration with integrin-mediated adhesion, thereby supporting the persistence and organization of specialized effector T-cell subsets within structured immune niches (29).

The combined treatment with IL-32α and CCL20 reproduced several adhesion-related responses observed with individual stimulations but amplified the expression of genes governing focal adhesion and cytoskeletal remodeling. Upregulated transcripts including G protein–coupled receptor 3 *(GPR3)*, actin-binding/signaling *(CAP2)*, cadherin 4 *(CDH4)*, dynein cytoplasmic 1 intermediate chain 1 *(DYNC1/1)*, and Rho guanine nucleotide exchange factor *(ARHGEF3)*. This cooperative transcriptional profile suggests that IL-32α and CCL20 jointly reinforce migration programs through both integrin-dependent and integrin-independent mechanisms, potentially promoting the formation of adhesion structures required for efficient T-cell movement.

CXCL8 is a chemokine strongly implicated in tumor biology and has been associated with angiogenesis, myeloid recruitment, and immunosuppressive microenvironmental remodeling (31–34). Although classically linked to neutrophil trafficking, human CD8^+^ T cells retain CXCR1 within intracellular vesicles that can be rapidly mobilized upon activation (35). Consistent with this, early studies identified a CXCL8-responsive CD8^+^ T-cell subset with heightened cytotoxic potential (36, 37). In our study, however, CXCL8 alone did not markedly alter the transcriptional landscape of T cells.

CXCL8 combined with IL-15, presents a transcriptional profile predominantly shaped by IL-15 signaling. Notably, when CXCL8 was combined with IL-32α, the transcriptional response shifted toward a hybrid profile, integrating gene programs characteristic of both the CXCL8 and IL-32α pathways (21, 38). This cooperative effect suggests that IL-32α and CXCL8 engage in a form of signaling plasticity, whereby transient chemotactic cues are transformed into more sustained transcriptional outputs that enhance T-cell survival, functional stability, and tissue engagement (21, 38).

IL-32α primarily activated gene programs associated with cell survival and proliferation, and the transcriptional profile suggested that its effects may be mediated, at least in part, through a G-protein coupled receptor like signaling mechanism. An intriguing finding was the up-regulation of *PCDHB6*, a protocadherin processed by γ-secretase, which points to an indirect connection with NOTCH1 activity, a relationship that was experimentally validated in this study. The link between IL-32α and cytokine signaling is likely driven by the induction of IL-15, whose expression in T cells increased following IL-32α stimulation, a pattern also observed upon CCL20 treatment. These findings also align with previous reports indicating that IL-32α promotes pro-inflammatory cytokine production through NF-κB and p38, MAPK activation in cancer models, a response that is further amplified by IL-15–driven signaling (39, 40).

A reciprocal relationship between IL-32 and cytokine signaling has previously been described in human T cells. IL-2 receptor signaling induces IL-32 expression whereas T-cell receptor (TCR) stimulation is required for its secretion, with the highest IL-32 expression detected in intratumoral Tregs (41). Consistent with these findings, another study reported that IL-32 was most highly expressed in CD4^+^ T cells subsets in esophageal squamous cell carcinoma (42). Moreover, IL-32 knockdown reduced FOXP3 in CD4^+^ T cells and IFN-γ production by CD8^+^ T cells effector function within the tumor microenvironment (42).

In the cytokine milieu, IL-15 functions as a central node, balancing effector activation with regulatory restraint. In our dataset, IL-15 simultaneously upregulated pro-inflammatory mediators, including *IL1R1, IL2RA, IL15, IL17F, IL32, CXCL9, TNF*, and *STAT1* together with negative-feedback regulators such as *CISH, CD274 (PD-L1), FOXP3*, and *SOCS2*. This dual transcriptional program consists of the well-established role of IL-15 in promoting immune activation while preserving immune homeostasis. Indeed, IL-15 supports the survival of memory CD8^+^ T cells through induction of BCL-2, while preventing excessive stimulation (20, 43). The concomitant induction of inflammatory and regulatory pathways suggests that IL-15 maintains T-cell functionality in a state of sustained readiness while limiting the development of dysfunctional responses. Supporting this interpretation, IL-15 has been shown to preferentially expand the TCF1^+^ progenitor exhausted CD8^+^ T-cell subset over terminally differentiated exhausted cells in both chronic viral infection and human renal cell carcinoma, while downregulating genes associated with T-cell receptor signaling and apoptosis (44).

Among atypical chemokine receptors, ACKR2 and ACKR4 exert complementary roles in shaping chemokine gradients and regulating leukocyte trafficking. ACKR2 (also known as D6) constitutively internalizes and degrades inflammatory chemokines through β-arrestin–mediated trafficking, thereby limiting chemokine availability and contributing to the resolution of inflammation (45). Efficient ligand scavenging by ACKR2 depends on N-terminal tyrosine sulfation which is critical for its chemokine-binding capacity (46). Although ACKR2 was originally characterized as a scavenger of inflammatory CC chemokines, its ligand repertoire has recently been expanded. A study recently demonstrated that ACKR2 also binds CXCL12 and scavenges CXCL11 and CXCL5, identifying these CXC chemokines as previously unrecognized ligands. (47).

ACKR4, in contrast, primarily shapes CCL21 gradients and regulates dendritic-cell migration (48). In T cells, ACKRs are abundantly localized within cytoplasmic vesicles and show variable surface expression, a dynamic that has been described but not yet mechanistically explained (49). The positive or negative modulation of ACKR2 and ACKR4 at the plasma membrane remains largely uncharacterized, and our results provide new insight into this underexplored layer of regulation.

In our dataset, the reduced expression of these scavenger receptors under most treatment conditions, together with the protein level confirmation of ACKR2 downregulation of at protein level, suggests a diminished capacity of T cells to clear chemokines, potentially prolonging GPCR signaling and reinforcing inflammatory circuits. This reduction in chemokine scavenging may sustain local chemokine gradients, thereby promoting persistent inflammatory signaling and enhancing the recruitment of both effector and regulatory T-cell subsets to inflamed or tumor-like microenvironments (35, 46, 48). Conversely, the study from Tang et al. demonstrated that ACKR2 overexpression in bystander cells is sufficient to abolish cancer-associated fibroblast (CAF)-mediated chemoattraction of cytotoxic T cells *in vitro* (47), supporting the notion that reduced ACKR2 expression may preserve chemokine gradients and facilitate T cell recruitment.

Together, these findings reveal that interactions between chemokine and cytokine establish a multilayered regulatory framework that fine-tunes T cell behavior under inflammatory conditions. Rather than acting as isolated cues, IL-15, IL-32α, CCL20, and CXCL8 orchestrate dynamic and context-dependent transcriptional circuits that either amplify dominant pathways (as seen with IL-15–mediated responses) or promote cooperative signaling states with emergent functions, including enhanced adhesion, cytoskeletal remodeling, vesicular trafficking, and metabolic rewiring.

The identification of CCL20 as a central coordinator of migratory and adhesion programs, through the integration of CCR6 signaling, small GTPases, and integrin activation underscores its broader role in guiding T cells recruitment and positioning within inflamed or remodeled tissues, including tumors. Consistent with this concept tumor-derived CCL20 has been shown to preferentially recruit regulatory T cells and is associated with shorter progression-free survival (25).

Likewise, the discovery that IL-32α modulates GPCR-like signaling and functionally intersects with NOTCH-1 and IL-15 pathways provide new insight into how survival and activation programs are coordinately regulated. Finally, the reduced expression of ACKR2 and ACKR4 across most conditions suggests diminished chemokine scavenging, leading to prolonged chemokine availability. This shift may sustain GPCR signaling, preserve chemokine gradients, and ultimately favor recruitment and retention of effector and regulatory T-cells retention in tumor-like microenvironments.

At the translational level, therapeutic strategies targeting CXCL8 have demonstrated only modest clinical efficacy, either as monotherapy or in combination with immune checkpoint blockade and have not consistently achieved durable tumor regression (50, 51). Our findings provide a potential mechanistic explanation for these limited clinical outcomes. Specifically, we show that chemokines possess overlapping and functionally redundant biological activities and that cytokine stimulation further amplifies these chemokine networks. Consequently, inhibition of CXCL8 alone may be insufficient, as compensatory signaling through alternative chemokine pathways can maintain chemotactic gradients and inflammatory responses. Such transcriptional redundancy may sustain the recruitment of immunoregulatory cells, preserve inflammatory signaling, and promote tumor-supportive functions despite CXCL8 blockade, highlighting the potential benefit of therapeutic strategies that concurrently target both cytokine- and chemokine-driven signaling networks rather than a single chemokine axis.

Furthermore, IL-15 is widely incorporated into adoptive cell therapy protocols, including the *ex vivo* expansion of tumor-infiltrating lymphocytes (TILs), CAR-T cells, CAR-NK cells, and TCR-engineered T cells, owing to its ability to promote T-cell survival, proliferation, metabolic fitness, and long-term persistence (52: 53, 54).

However, our findings indicate that, in addition to inducing a robust pro-inflammatory transcriptional program, IL-15 simultaneously upregulates several negative regulatory molecules in T cells, including CISH, CD274 (PD-L1), FOXP3, and SOCS2. Notably, CISH was among the most highly induced IL-15-responsive genes in our dataset. Given that CISH functions as a key intracellular negative regulator of IL-15 signaling and is currently being targeted by gene-editing strategies to enhance the persistence and antitumor activity of adoptive cell therapies, our findings suggest that IL-15-induced activation of this regulatory axis may represent an important mechanism limiting the long-term efficacy of IL-15-based cellular immunotherapies (55).

This coordinated induction of immunoregulatory pathways suggests that IL-15 not only enhances effector function but also activates intrinsic negative-feedback mechanisms that may restrain sustained T-cell activation. These observations raise the possibility that prolonged IL-15 exposure during cell manufacturing or following adoptive transfer could contribute to adaptive regulatory programs that ultimately limit the durability and therapeutic efficacy of IL-15-based cellular immunotherapies.

Collectively, these integrated transcriptional responses illustrate the remarkable plasticity of T-cell states and demonstrate that coordinated chemokine and cytokine signaling shapes T-cell functional diversification in a highly context-dependent manner. Beyond regulating migration, these signaling networks simultaneously control activation, adhesion, metabolic adaptation, immune regulation, and tissue retention, emphasizing the extensive functional crosstalk between inflammatory mediators. Our findings further suggest that the biological effects of individual cytokines or chemokines cannot be fully understood in isolation but rather emerge from complex, interconnected signaling networks capable of compensatory and cooperative responses. This concept has important translational implications. The functional redundancy among chemokines may partly explain the limited efficacy of therapeutic strategies targeting a single chemokine axis, such as CXCL8, whereas the dual immunostimulatory and immunoregulatory activities of IL-15 highlight the need to carefully optimize cytokine exposure during adoptive cell therapy manufacturing. Together, these observations support the development of next-generation immunotherapeutic strategies that simultaneously modulate complementary cytokine and chemokine pathways while minimizing intrinsic negative-feedback mechanisms. Such approaches may improve immune-cell trafficking, sustain effector-cell functionality, enhance persistence after adoptive transfer, and ultimately achieve more durable antitumor responses. More broadly, our study provides a systems-level framework for understanding how cytokine–chemokine interactions coordinate T-cell behavior and offers new mechanistic insights that may guide the rational design of combination immunotherapies for cancer and other chronic inflammatory diseases.

## Data Availability

The datasets presented in this study can be found in online repositories. The names of the repository/repositories and accession number(s) can be found in the article/**Supplementary Material**.
