# Supplementary material for: Dynamic integration of CXCL8 or CCL20 signaling with IL-15 or IL-32α reprograms human T-cell functional plasticity
Source: Front Immunol. 2026 Aug 18;17:1910983. doi: 10.3389/fimmu.2026.1910983 (PMC13529521; doi:10.3389/fimmu.2026.1910983)
Supplement: Supplementary file 1 [file DataSheet1.pdf]

Supplementary Table S1

Differential gene expression profiles of with the treatments: CCL20, CXCL8, IL-15, IL-32α, and their combinatorial stimulations compared with untreated T cells.

(A) CCL20 treated T cells

ID	FC CCL20 vs CONTROL	P-val	Gene Symbol	Description
TC0100015793.hg.1	5.13	0.0011	POGZ	Transcript Identified by AceView. Entrez Gene ID(s) 23126
TC0100010925.hg.1	3.28	0.0466	OCLM	oculomedin
TC1800008235.hg.1	2.69	0.0167	ABHD3	Transcript Identified by AceView. Entrez Gene ID(s) 171586
TC0600013007.hg.1	2.59	0.0366	FAM184A	family with sequence similarity 184. member A
TC0400010898.hg.1	2.36	0.0251	TMPRSS11F	transmembrane protease. serine 11F
TC0200012248.hg.1	2.35	0.0084	FEZ2	fasciculation and elongation protein zeta 2 (zygin II)
TC1700012383.hg.1	2.34	0.0133	OMG	oligodendrocyte myelin glycoprotein
TC0300011049.hg.1	2.24	0.0341	QRICH1	Transcript Identified by AceView. Entrez Gene ID(s) 54870
TC0100014365.hg.1	2.12	0.0258	CYP2J2	cytochrome P450. family 2. subfamily J. polypeptide 2
TC0700011954.hg.1	2.08	0.0329	CYP3A7; CYP3A7-CYP3A51P	cytochrome P450. family 3. subfamily A. polypeptide 7; CYP3A7-CYP3A51P readthrough
TC1400008622.hg.1	2.04	0.0016	OR5AU1	olfactory receptor. family 5. subfamily AU. member 1
TC1100011283.hg.1	2.03	0.0064	RIN1	Ras and Rab interactor 1
TC1900011874.hg.1	2.03	0.0328	ZNF878	zinc finger protein 878
TC1500007238.hg.1	2.01	0.0105	TMOD2	tropomodulin 2 (neuronal)

TC0100010303.hg.1	2	0.0434	SLAMF6	Memczak2013 ANTISENSE. coding. INTERNAL. intronic best transcript NM_052931
TC0700007201.hg.1	1.99	0.0071	ANLN	Transcript Identified by AceView. Entrez Gene ID(s) 54443
TC1100010033.hg.1	1.96	0.0479	TRIM66	tripartite motif containing 66
TC1900011633.hg.1	1.94	0.0466	ZBTB45	Memczak2013 ALT_ACCEPTOR. ALT_DONOR. coding. INTERNAL. intronic best transcript NM_032792
TC0X00009197.hg.1	1.93	0.0335	PHKA2	phosphorylase kinase. alpha 2 (liver)
TSUnmapped00000384.hg.1	1.91	0.0076	ZNF546	zinc finger protein 546
TC0300006793.hg.1	1.9	0.0485	KAT2B	Transcript Identified by AceView. Entrez Gene ID(s) 8850
TC1500006806.hg.1	1.89	0.0296	RYR3	ryanodine receptor 3
TC0400009935.hg.1	1.89	0.0446	CCDC96	coiled-coil domain containing 96
TC1100009990.hg.1	1.88	0.0435	OR6A2	olfactory receptor. family 6. subfamily A. member 2
TC0100015629.hg.1	1.87	0.0083	FMO5	flavin containing monooxygenase 5
TC1200010493.hg.1	1.87	0.0104	DBX2	developing brain homeobox 2
TC2000009851.hg.1	1.86	0.0222	SAMD10	sterile alpha motif domain containing 10
TC0900009458.hg.1	1.86	0.0287	RLN1	relaxin 1
TC0400011043.hg.1	1.83	0.0308	CDKL2	cyclin-dependent kinase-like 2 (CDC2-related kinase)
TC0600009171.hg.1	1.82	0.0144	RFPL4B	ret finger protein-like 4B
TC0200012031.hg.1	1.82	0.0274	HADHA	Transcript Identified by AceView. Entrez Gene ID(s) 3030
TC0600013190.hg.1	1.81	0.0496	MOXD1	monooxygenase. DBH-like 1
TC0200016693.hg.1	1.8	0.015	LRRTM4	leucine rich repeat transmembrane neuronal 4
TC1000008482.hg.1	1.8	0.022	CEP55	centrosomal protein 55kDa
TC1500006522.hg.1	1.79	0.025	GOLGA8DP	golgin A8 family. member D. pseudogene

TC0800006781.hg.1	1.79	0.0288	ZNF705CP	zinc finger protein 705C. pseudogene
TC0400007918.hg.1	1.79	0.0375	CXCL13	chemokine (C-X-C motif) ligand 13
TC0500013186.hg.1	1.77	0.0048	PTCD2	pentatricopeptide repeat domain 2
TC1900011471.hg.1	1.77	0.0227	UBE2S	Zhang2013 ALT_ACCEPTOR. ALT_DONOR. coding. INTERNAL. intronic. OVERLAPTX best transcript NM_014501
TC0800009854.hg.1	1.76	0.0215	BIN3-IT1	BIN3 intronic transcript 1
TC0100014502.hg.1	1.76	0.0316	WDR78	WD repeat domain 78
TC0100008921.hg.1	1.76	0.0464	CLCA2	chloride channel accessory 2
TC2200008465.hg.1	1.74	0.0178	SELM	selenoprotein M; selenoprotein M [Source:EntrezGene;Acc:140606]; Salzman2013 ANNOTATED. CDS. coding. OVCODE. OVERLAPTX. OVEXON. UTR3 best transcript NM_080430; Salzman2013 ANNOTATED. CDS. coding. OVCODE. OVERLAPTX. OVEXON. UTR3. UTR5 best transcript NM_080430; Transcript Identified by AceView. Entrez Gene ID(s) 140606
TC0200016505.hg.1	1.72	0.0124	CCDC138	coiled-coil domain containing 138
TC0600014240.hg.1	1.71	0.0269	LOC100130357; RP1-257A7.4	uncharacterized LOC100130357; novel transcript. antisense to PHACTR1
TC1800006487.hg.1	1.71	0.0452	SMCHD1	structural maintenance of chromosomes flexible hinge domain containing 1
TC0X00011300.hg.1	1.71	0.0465	ZC3H12B	zinc finger CCCH-type containing 12B
TC0100013802.hg.1	1.69	0.0013	HPCAL4	hippocalcin like 4
TC2200007909.hg.1	1.69	0.0131	MICAL3	microtubule associated monooxygenase. calponin and LIM domain containing 3
TSUnmapped00000614.hg.1	1.69	0.0208	ZNF660	zinc finger protein 660
TC1800006935.hg.1	1.69	0.0464	PSMA8	proteasome subunit alpha 8

TC1400010760.hg.1	1.68	0.0214	ATP6V1D	ATPase. H+ transporting. lysosomal 34kDa. V1 subunit D
TC2000007213.hg.1	1.68	0.0451	MMP24	matrix metallopeptidase 24 (membrane-inserted)
TC0200013594.hg.1	1.67	0.027	COA5	cytochrome c oxidase assembly factor 5
TC0800007554.hg.1	1.66	0.0258	UBE2V2	ubiquitin conjugating enzyme E2 variant 2
TC1200007853.hg.1	1.66	0.0499	GPR182	G protein-coupled receptor 182
TC0300007500.hg.1	1.65	0.0054	ITIH3	inter-alpha-trypsin inhibitor heavy chain 3
TC0600007769.hg.1	1.65	0.0193	SNRPC	small nuclear ribonucleoprotein polypeptide C
TSUnmapped00000406.hg.1	1.65	0.0282	ZNF660	zinc finger protein 660
TC0700013341.hg.1	1.64	0.022	CHN2	chimerin 2
TC0100014776.hg.1	1.64	0.0241	DDAH1	dimethylarginine dimethylaminohydrolase 1
TC1500007185.hg.1	1.64	0.0421	FGF7	fibroblast growth factor 7
TC0100017935.hg.1	1.63	0.0193	RGS7	regulator of G-protein signaling 7
TC1600011526.hg.1	1.62	0.0352	PRSS53	protease. serine 53
TC1200007157.hg.1	1.62	0.0423	REP15	RAB15 effector protein
TC0X00007937.hg.1	1.6	0.0273	DRP2	dystrophin related protein 2
TC0200016744.hg.1	1.6	0.0357	ITGB6	integrin beta 6
TC1900010951.hg.1	1.6	0.0369	SNRPD2	small nuclear ribonucleoprotein D2 polypeptide
TC0200016417.hg.1	1.6	0.0416	CENPA	centromere protein A
TC2200008680.hg.1	1.59	0.0061	BAIAP2L2	BAI1-associated protein 2-like 2
TC0400007988.hg.1	1.59	0.007	THAP9	THAP domain containing 9
TC0100011512.hg.1	1.59	0.0301	DTL	denticleless E3 ubiquitin protein ligase homolog (Drosophila)
TC2200008800.hg.1	1.59	0.0363	DNAJB7	DnaJ (Hsp40) homolog. subfamily B. member 7
TC0500008324.hg.1	1.59	0.0478	EPB41L4A-AS2	EPB41L4A antisense RNA 2 (head to head)

TC1400006823.hg.1	1.59	0.0488	SCFD1	sec1 family domain containing 1
TC0100015791.hg.1	1.58	0.0339	POGZ	Transcript Identified by AceView. Entrez Gene ID(s) 23126
TC0600012932.hg.1	1.57	0.0201	HS3ST5	heparan sulfate (glucosamine) 3-O-sulfotransferase 5
TC0200013772.hg.1	1.57	0.022	RGPD3	RANBP2-like and GRIP domain containing 3
TC1700009219.hg.1	1.57	0.0223	RAC3	ras-related C3 botulinum toxin substrate 3 (rho family. small GTP binding protein Rac3)
TC1000008508.hg.1	1.57	0.0318	CYP2C9	cytochrome P450. family 2. subfamily C. polypeptide 9
TC0500009109.hg.1	1.57	0.0404	GPX3	glutathione peroxidase 3
TC0300007290.hg.1	1.57	0.0421	KLHL18	kelch-like family member 18
TC0400006714.hg.1	1.56	0.0057	TADA2B	Transcript Identified by AceView. Entrez Gene ID(s) 93624
TC2000009550.hg.1	1.56	0.0201	AURKA	aurora kinase A
TC0300008782.hg.1	1.56	0.0489	IFT122	intraflagellar transport 122
TC0900008337.hg.1	1.56	0.0494	ZNF462	zinc finger protein 462
TC0700006632.hg.1	1.55	0.0438	OCM	oncomodulin
TC1200007740.hg.1	1.54	0.0292	HOXC8	homeobox C8
TC1600009978.hg.1	1.54	0.0301	ZNF785	zinc finger protein 785
TC0400008388.hg.1	1.54	0.0472	GAR1	GAR1 homolog. ribonucleoprotein
TC0100012535.hg.1	1.53	0.018	HES5	hes family bHLH transcription factor 5
TC1700008489.hg.1	1.53	0.0209	C17orf64	chromosome 17 open reading frame 64
TC0X00011393.hg.1	1.53	0.0333	CT47A5; CT47A2; CT47A1; CT47A11; CT47A12	cancer/testis antigen family 47. member A5; cancer/testis antigen family 47. member A2; cancer/testis antigen family 47. member A1; cancer/testis antigen family 47. member A11; cancer/testis antigen family 47. member A12
TC1700012271.hg.1	1.53	0.0382	GOSR2	golgi SNAP receptor complex member 2
TC1800006905.hg.1	1.53	0.0403	CABYR	calcium binding tyrosine-(Y)-phosphorylation regulated

TC0700012009.hg.1	1.53	0.0431	PCOLCE-AS1	PCOLCE antisense RNA 1
TC1600008972.hg.1	1.52	0.0033	WDR24	WD repeat domain 24
TC0800012376.hg.1	1.52	0.0229	ZNF707	zinc finger protein 707
TC0100007020.hg.1	1.52	0.033	CLCNKB	chloride channel. voltage-sensitive Kb
TC0600007087.hg.1	1.52	0.0367	CAP2	CAP. adenylate cyclase-associated protein. 2 (yeast)
TC1100007610.hg.1	1.51	0.0091	OR5D14	olfactory receptor. family 5. subfamily D. member 14
TC0500013141.hg.1	1.51	0.0093	PDCD6	programmed cell death 6
TC2000006961.hg.1	1.51	0.018	CSTL1	cystatin-like 1
TC1800007367.hg.1	1.5	0.0025	TCF4	Memczak2013 ANTISENSE. coding. INTERNAL. intronic best transcript NM_001243234
TC0100017193.hg.1	1.5	0.0252	KCNH1	potassium channel. voltage gated eag related subfamily H. member 1
TC0300012038.hg.1	1.49	0.0307	CCDC191	coiled-coil domain containing 191
TC0100014127.hg.1	1.49	0.0408	SPATA6	spermatogenesis associated 6
TC1100010514.hg.1	1.48	0.0463	ELF5	E74-like factor 5 (ets domain transcription factor)
TC1800009243.hg.1	1.47	0.0398	SERPINB10	serpin peptidase inhibitor. clade B (ovalbumin). member 10
TC2000009220.hg.1	1.46	0.0029	TP53TG5	TP53 target 5
TC1900007004.hg.1	1.46	0.0231	C19orf52	chromosome 19 open reading frame 52
TC1600006752.hg.1	1.46	0.0383	NUDT16L1	nudix hydrolase 16 like 1
TC0300013083.hg.1	1.46	0.0457	LRRC34	leucine rich repeat containing 34
TC0300011105.hg.1	1.45	0.0483	RASSF1	Ras association (RalGDS/AF-6) domain family member 1
TC2000009209.hg.1	1.44	0.0078	KCNS1	potassium voltage-gated channel. modifier subfamily S. member 1
TC2200008346.hg.1	1.44	0.0108	MN1	meningioma (disrupted in balanced translocation) 1

TC1700009693.hg.1	1.44	0.015	RNF222	ring finger protein 222
TC1000012156.hg.1	1.44	0.0268	TEX36	testis expressed 36
TC0200014802.hg.1	1.44	0.0391	SCN3A	sodium channel. voltage gated. type III alpha subunit
TC0400008861.hg.1	1.44	0.049	IL15	interleukin 15
TC0900012139.hg.1	1.43	0.0318	PGM5	phosphoglucomutase 5
TC0800008371.hg.1	1.43	0.0326	SPAG1	sperm associated antigen 1
TC0X00009507.hg.1	1.43	0.0336	CXorf36	chromosome X open reading frame 36
TC1400009299.hg.1	1.43	0.0418	C14orf105	chromosome 14 open reading frame 105
TC1900011822.hg.1	1.42	0.0417	ZNF586	zinc finger protein 586
TC1600009096.hg.1	1.41	0.0075	CASKIN1	CASK interacting protein 1
TC1200008642.hg.1	1.4	0.0076	PARPBP	PARP1 binding protein
TC0Y00007240.hg.1	-1.4	0.0126	CSPG4P1Y; DNM1P24	chondroitin sulfate proteoglycan 4 pseudogene 1. Y-linked; dynamin 1 pseudogene 24
TC0100009056.hg.1	-1.41	0.0465	CCDC18	coiled-coil domain containing 18
TC1700010639.hg.1	-1.42	0.005	KRT10	keratin 10. type I
TC0X00011144.hg.1	-1.42	0.0274	PNMA5	paraneoplastic Ma antigen family member 5
TC0500009303.hg.1	-1.42	0.028	GABRA1	gamma-aminobutyric acid (GABA) A receptor. alpha 1
TC0200016683.hg.1	-1.42	0.0445	C2orf42	chromosome 2 open reading frame 42
TC0600008525.hg.1	-1.43	0.0467	KHDC3L	KH domain containing 3-like. subcortical maternal complex member
TC0100010651.hg.1	-1.44	0.0098	LOC730159; RP11-360D2.1	uncharacterized LOC730159; putative novel transcript
TC0700013617.hg.1	-1.44	0.026	GCC1	GRIP and coiled-coil domain containing 1
TC1500009760.hg.1	-1.45	0.0429	PIF1	PIF1 5-to-3 DNA helicase
TC0600012008.hg.1	-1.46	0.0051	DEFB133	defensin. beta 133
TC0100013992.hg.1	-1.46	0.0158	TCTEX1D4	Tctex1 domain containing 4

TC1700009811.hg.1	-1.46	0.0224	HS3ST3A1	Jeck2013 ALT_ACCEPTOR. ALT_DONOR. coding. INTERNAL. intronic best transcript NM_006042
TC0200012030.hg.1	-1.46	0.0284	HADHA	hydroxyacyl-CoA dehydrogenase/3-ketoacyl-CoA thiolase/enoyl-CoA hydratase (trifunctional protein). alpha subunit
TC0X00006676.hg.1	-1.46	0.0299	BMX	BMX non-receptor tyrosine kinase
TC2000009624.hg.1	-1.46	0.0336	APCDD1L	adenomatosis polyposis coli down-regulated 1-like
TC0900009855.hg.1	-1.46	0.0418	BAG1	BCL2-associated athanogene
TC0300010228.hg.1	-1.46	0.0492	FANCD2OS	FANCD2 opposite strand
TC0500011470.hg.1	-1.47	0.0027	FAM172A; POU5F2; MIR2277	family with sequence similarity 172. member A; POU domain class 5. transcription factor 2; microRNA 2277
TC1800007490.hg.1	-1.47	0.0045	CDH20	cadherin 20. type 2
TC0200014246.hg.1	-1.47	0.0182	POTEF	POTE ankyrin domain family. member F
TC0300007669.hg.1	-1.47	0.0303	SYNPR	synaptoporin
TC0100015865.hg.1	-1.47	0.0413	S100A5	S100 calcium binding protein A5
TC0900009561.hg.1	-1.47	0.0472	LINC01235	long intergenic non-protein coding RNA 1235
TC0100010267.hg.1	-1.47	0.0477	DUSP23	dual specificity phosphatase 23
TC0600011605.hg.1	-1.47	0.0498	TCP11	t-complex 11. testis-specific
TC1400007695.hg.1	-1.48	0.0088	EIF2B2	eukaryotic translation initiation factor 2B. subunit 2 beta. 39kDa
TC1700007922.hg.1	-1.48	0.0095	G6PC	glucose-6-phosphatase. catalytic subunit
TC0400006802.hg.1	-1.48	0.0146	USP17L19	ubiquitin specific peptidase 17-like family member 19
TC0200007047.hg.1	-1.48	0.0325	DPYSL5	dihydropyrimidinase-like 5
TC2000007942.hg.1	-1.48	0.0411	FAM217B	family with sequence similarity 217. member B
TC1000007007.hg.1	-1.48	0.0463	ARMC3	armadillo repeat containing 3

TC0700011530.hg.1	-1.49	0.0094	WBSCR16	Williams-Beuren syndrome chromosome region 16
TC2000008807.hg.1	-1.49	0.0096	DEFB119	defensin. beta 119
TC0900008665.hg.1	-1.5	0.0084	OR1J2	olfactory receptor. family 1. subfamily J. member 2
TC0200006831.hg.1	-1.5	0.0236	VSNL1	visinin like 1
TC0900009700.hg.1	-1.5	0.0362	IFNA14	interferon. alpha 14
TC1500006811.hg.1	-1.5	0.0381	CHRM5	cholinergic receptor. muscarinic 5
TC2000006964.hg.1	-1.5	0.0426	CST8	cystatin 8 (cystatin-related epididymal specific)
TSUnmapped00000086.hg.1	-1.5	0.0499	SAG	S-antigen; retina and pineal gland (arrestin)
TC0200013003.hg.1	-1.51	0.0161	FIGLA	folliculogenesis specific bHLH transcription factor
TC0400009080.hg.1	-1.51	0.0229	NPY2R	neuropeptide Y receptor Y2
TC1700008014.hg.1	-1.51	0.0384	ADAM11	ADAM metallopeptidase domain 11
TC0700012850.hg.1	-1.51	0.0391	TAS2R38	taste receptor. type 2. member 38
TC1300008766.hg.1	-1.52	0.025	ENOX1	ecto-NOX disulfide-thiol exchanger 1
TC0800007284.hg.1	-1.52	0.0368	KCNU1	potassium channel. subfamily U. member 1
TC1100009110.hg.1	-1.52	0.0372	NNMT	nicotinamide N-methyltransferase
TC1700012039.hg.1	-1.52	0.0423	LOC100130370; RP11-1055B8.3	uncharacterized LOC100130370; novel transcript
TC0200013668.hg.1	-1.53	0.0015	CREG2	cellular repressor of E1A-stimulated genes 2
TC1700007230.hg.1	-1.53	0.0082	CDRT15L2	CMT1A duplicated region transcript 15-like 2
TC0500012164.hg.1	-1.53	0.0271	GFRA3	GDNF family receptor alpha 3
TC1200010756.hg.1	-1.53	0.0308	KRT6B	keratin 6B. type II
TC1700012398.hg.1	-1.53	0.0351	KRT27	keratin 27. type I
TC2100007821.hg.1	-1.54	0.0198	ADAMTS1	ADAM metallopeptidase with thrombospondin type 1 motif 1

TC1600007285.hg.1	-1.54	0.0309	HS3ST4	heparan sulfate (glucosamine) 3-O-sulfotransferase 4
TC1100012197.hg.1	-1.54	0.0373	GUCY1A2	guanylate cyclase 1. soluble. alpha 2
TC0100011389.hg.1	-1.54	0.0394	IL20	interleukin 20
TC1900007415.hg.1	-1.54	0.0462	DDX49	DEAD (Asp-Glu-Ala-Asp) box polypeptide 49
TC0700008961.hg.1	-1.55	0.0324	ASB15	ankyrin repeat and SOCS box containing 15
TC0200012946.hg.1	-1.55	0.0478	BMP10	bone morphogenetic protein 10
TC0500013016.hg.1	-1.56	0.0087	COL23A1	collagen. type XXIII. alpha 1
TC0100013647.hg.1	-1.56	0.0254	TRIM62	tripartite motif containing 62
TC0200009067.hg.1	-1.56	0.0295	TMEM37	transmembrane protein 37
TC1600008330.hg.1	-1.56	0.0385	HPR	haptoglobin-related protein
TC1600007978.hg.1	-1.57	0.0024	SLC12A3	solute carrier family 12 (sodium/chloride transporter). member 3
TC0900008842.hg.1	-1.57	0.0055	SLC25A25	Zhang2013 ALT_ACCEPTOR. ALT_DONOR. coding. INTERNAL. intronic best transcript NM_001006641
TC1600007723.hg.1	-1.57	0.0111	GPT2	glutamic pyruvate transaminase (alanine aminotransferase) 2
TC0400008086.hg.1	-1.57	0.0388	IBSP	integrin-binding sialoprotein
TC2100006883.hg.1	-1.58	0.0079	KRTAP20-3	keratin associated protein 20-3
TC1200007882.hg.1	-1.58	0.0364	GLI1	GLI family zinc finger 1
TC0300012319.hg.1	-1.58	0.0448	goyborbu; MCM2	Transcript Identified by AceView; Memczak2013 ANTISENSE. CDS. coding. INTERNAL. UTR3 best transcript NM_004526
TC0100010167.hg.1	-1.59	0.0069	TSACC	TSSK6 activating co-chaperone
TC1800006447.hg.1	-1.59	0.02	CLUL1	clusterin-like 1 (retinal)
TC1100011914.hg.1	-1.59	0.0322	TRIM64B	tripartite motif containing 64B
TC0100009408.hg.1	-1.6	0.0148	PIFO	primary cilia formation
TC1600009949.hg.1	-1.6	0.025	FAM57B	family with sequence similarity 57. member B
TC1500006562.hg.1	-1.6	0.0338	NPAP1	nuclear pore associated protein 1

TC1300007707.hg.1	-1.6	0.0382	GPC5	glypican 5
TC1100007614.hg.1	-1.6	0.0393	OR5D16	olfactory receptor. family 5. subfamily D. member 16
TC0700009222.hg.1	-1.61	0.0154	LRGUK	leucine-rich repeats and guanylate kinase domain containing
TSUnmapped00000539.hg.1	-1.61	0.0234	LRIG1	
TSUnmapped00000052.hg.1	-1.61	0.0282	HHAT	hedgehog acyltransferase
TC1100006752.hg.1	-1.61	0.0467	RBMXL2	RNA binding motif protein. X-linked-like 2
TC0500013385.hg.1	-1.62	0.0061	SH3TC2	SH3 domain and tetratricopeptide repeats 2
TC1500007839.hg.1	-1.62	0.0111	ISLR	immunoglobulin superfamily containing leucine-rich repeat
TC0100016205.hg.1	-1.62	0.0215	C1orf111	chromosome 1 open reading frame 111
TC1500008002.hg.1	-1.62	0.0215	ANKRD34C	ankyrin repeat domain 34C
TC0700009145.hg.1	-1.62	0.0242	MEST	mesoderm specific transcript
TC0100013024.hg.1	-1.62	0.0352	FAM131C	family with sequence similarity 131. member C
TC1500007143.hg.1	-1.62	0.0364	SEMA6D	sema domain. transmembrane domain (TM). and cytoplasmic domain. (semaphorin) 6D
TC1100007626.hg.1	-1.62	0.0406	OR8H3	olfactory receptor. family 8. subfamily H. member 3
TC0100015872.hg.1	-1.63	0.0124	S100A14	S100 calcium binding protein A14
TC2000008027.hg.1	-1.63	0.0136	NTSR1	neurotensin receptor 1 (high affinity)
TC0900008679.hg.1	-1.63	0.0252	OR1K1	olfactory receptor. family 1. subfamily K. member 1
TC2200006552.hg.1	-1.63	0.0274	TMEM191B	transmembrane protein 191B
TC0700007002.hg.1	-1.63	0.0389	HOXA-AS3	HOXA cluster antisense RNA 3
TC2200007247.hg.1	-1.63	0.0452	MYH9	Memczak2013 ANTISENSE. CDS. coding. INTERNAL best transcript NM_002473

TSUnmapped00000078.hg.1	-1.64	0.0178	SLC16A1	solute carrier family 16 (monocarboxylate transporter). member 1
TC0100012950.hg.1	-1.64	0.0228	LRRC38	leucine rich repeat containing 38
TC0100015784.hg.1	-1.64	0.0355	SELENBP1	selenium binding protein 1
TSUnmapped00000525.hg.1	-1.64	0.0363	BCL2L14	BCL2-like 14 (apoptosis facilitator)
TC0400006984.hg.1	-1.65	0.0316	CLRN2	clarin 2
TC0400007345.hg.1	-1.65	0.0352	LIMCH1	LIM and calponin homology domains 1
TC0500006836.hg.1	-1.66	0.0324	FBXL7	F-box and leucine-rich repeat protein 7
TC1900011956.hg.1	-1.67	0.001	CYP2A7	cytochrome P450. family 2. subfamily A. polypeptide 7
TC0100007194.hg.1	-1.67	0.0016	UBXN10	UBX domain protein 10
TC0100015401.hg.1	-1.67	0.0043	IGSF3	immunoglobulin superfamily. member 3
TC0400012821.hg.1	-1.67	0.0194	C4orf22	chromosome 4 open reading frame 22
TC1800007570.hg.1	-1.67	0.0448	CDH7	cadherin 7. type 2
TC0400010694.hg.1	-1.68	0.0074	ERVMER34-1	endogenous retrovirus group MER34. member 1
TC1000011599.hg.1	-1.68	0.0145	CPN1	carboxypeptidase N. polypeptide 1
TC0100010867.hg.1	-1.68	0.0165	LAMC2	laminin. gamma 2
TSUnmapped00000641.hg.1	-1.68	0.0266	PRAMEF2	PRAME family member 2
TC1500008271.hg.1	-1.68	0.0312	WDR93	WD repeat domain 93
TC0200014244.hg.1	-1.68	0.0313	FAR2P1	fatty acyl-CoA reductase 2 pseudogene 1
TC1200012678.hg.1	-1.68	0.0471	PTPRQ	protein tyrosine phosphatase. receptor type. Q
TC1700007771.hg.1	-1.69	0.0313	GSDMA	gasdermin A
TC1600008821.hg.1	-1.69	0.0362	ZNF469	zinc finger protein 469
TC1500007237.hg.1	-1.7	0.0013	SCG3	secretogranin III
TC2200008393.hg.1	-1.7	0.0292	RHBDD3	rhomboid domain containing 3
TC1000012481.hg.1	-1.7	0.0411	CYP2C19	cytochrome P450. family 2. subfamily C. polypeptide 19
TC0X00008125.hg.1	-1.71	0.0435	PAK3	p21 protein (Cdc42/Rac)-activated kinase 3

TC1900009297.hg.1	-1.71	0.0491	TBXA2R	thromboxane A2 receptor
TC0400012856.hg.1	-1.72	0.0206	RAPGEF2	Rap guanine nucleotide exchange factor 2
TC0300013813.hg.1	-1.72	0.0243	ACKR2	atypical chemokine receptor 2
TC1500010051.hg.1	-1.72	0.0303	RPP25	ribonuclease P/MRP 25kDa subunit
TC0100009553.hg.1	-1.72	0.0366	C1orf137	chromosome 1 open reading frame 137
TC1100010809.hg.1	-1.72	0.0413	OR4C12	olfactory receptor. family 4. subfamily C. member 12
TC1300008072.hg.1	-1.73	0.0119	SPACA7	sperm acrosome associated 7
TC1100009883.hg.1	-1.73	0.0169	C11orf40	chromosome 11 open reading frame 40
TC0900008847.hg.1	-1.73	0.0194	LCN2	lipocalin 2
TC1800006655.hg.1	-1.73	0.0245	TXNDC2	thioredoxin domain containing 2 (spermatozoa)
TC1900011764.hg.1	-1.73	0.0414	GEMIN7	gem nuclear organelle associated protein 7
TC0900009922.hg.1	-1.74	0.0113	FAM205C	family with sequence similarity 205. member C
TC0100016339.hg.1	-1.74	0.0175	DPT	dermatopontin
TC1800008734.hg.1	-1.74	0.0194	STARD6	StAR-related lipid transfer domain containing 6
TC1900010971.hg.1	-1.74	0.0224	IGFL3	IGF like family member 3
TC1900006647.hg.1	-1.74	0.0416	CACTIN-AS1	CACTIN antisense RNA 1
TC1100013055.hg.1	-1.75	0.0278	CARNS1	carnosine synthase 1
TC0900010355.hg.1	-1.76	0.0134	C9orf135-AS1	C9orf135 antisense RNA 1 (head to head)
TC1100010857.hg.1	-1.76	0.0314	OR5M3	olfactory receptor. family 5. subfamily M. member 3
TC1100007307.hg.1	-1.76	0.0335	C11orf74	chromosome 11 open reading frame 74
TC1900006882.hg.1	-1.76	0.0478	CERS4	ceramide synthase 4
TC1200012577.hg.1	-1.77	0.0344	GALNT8	polypeptide N-acetylgalactosaminyltransferase 8
TC0100006910.hg.1	-1.77	0.0439	PRAMEF2	PRAME family member 2
TC0700007406.hg.1	-1.77	0.045	MYO1G	Memczak2013 ANTISENSE. CDS. coding. INTERNAL best transcript NM_033054
TC0100015593.hg.1	-1.78	0.0113	ANKRD35	ankyrin repeat domain 35

TC0300006773.hg.1	-1.78	0.0361	LOC339862; RP11-320G10.1; skerdorbo	uncharacterized LOC339862; novel transcript; Transcript Identified by AceView
TC0X00010468.hg.1	-1.78	0.0495	FRMPD3-AS1	FRMPD3 antisense RNA 1
TC0100009345.hg.1	-1.79	0.0103	CELSR2	cadherin. EGF LAG seven-pass G-type receptor 2
TC1100013066.hg.1	-1.79	0.0312	KRTAP5-10	keratin associated protein 5-10
TC1500006643.hg.1	-1.8	0.0224	GABRA5	gamma-aminobutyric acid (GABA) A receptor. alpha 5
TC1000008539.hg.1	-1.8	0.0463	DNTT	DNA nucleotidylexotransferase
TC0300011941.hg.1	-1.81	0.0013	MYH15	myosin. heavy chain 15
TC1500006959.hg.1	-1.81	0.0305	CHST14	carbohydrate (N-acetylgalactosamine 4-0) sulfotransferase 14
TC2000008802.hg.1	-1.82	0.0181	DEFB116	defensin. beta 116
TC0100014048.hg.1	-1.82	0.0419	POMGNT1	protein O-linked mannose N-acetylglucosaminyltransferase 1 (beta 1.2-)
TC1000011028.hg.1	-1.83	0.0031	MYOZ1	myozenin 1
TC1400010604.hg.1	-1.83	0.0062	MIA2	melanoma inhibitory activity 2
TC0100007747.hg.1	-1.83	0.0115	GJB3	gap junction protein beta 3
TC0500009281.hg.1	-1.83	0.0467	PTTG1	pituitary tumor-transforming 1
TC1100010850.hg.1	-1.84	0.0098	OR5T2	olfactory receptor. family 5. subfamily T. member 2
TC0600013204.hg.1	-1.84	0.0234	VNN3	vanin 3
TC0100018348.hg.1	-1.86	0.0278	CNIH3	cornichon family AMPA receptor auxiliary protein 3
TC0400009037.hg.1	-1.86	0.0373	MND1	meiotic nuclear divisions 1
TC1900010720.hg.1	-1.86	0.0401	C19orf54	chromosome 19 open reading frame 54
TC0100015850.hg.1	-1.88	0.023	SPRR2B	small proline-rich protein 2B
TC0100015853.hg.1	-1.89	0.0242	SPRR2G	small proline-rich protein 2G
TC1300009560.hg.1	-1.9	0.0186	RNF113B	ring finger protein 113B

TC0300011951.hg.1	-1.9	0.0338	MORC1	MORC family CW-type zinc finger 1
TC0200016026.hg.1	-1.9	0.0461	NMUR1	neuromedin U receptor 1
TC0200014361.hg.1	-1.92	0.0051	LYPD1	LY6/PLAUR domain containing 1
TC0100013749.hg.1	-1.92	0.0214	RSPO1	R-spondin 1
TC0200007854.hg.1	-1.94	0.0061	MEIS1	Meis homeobox 1
TC0300007499.hg.1	-1.94	0.0162	ITIH1	inter-alpha-trypsin inhibitor heavy chain 1
TC0900009417.hg.1	-1.94	0.048	RFX3	Memczak2013 ALT_ACCEPTOR. ALT_DONOR. coding. INTERNAL. intronic best transcript NM_002919
TC0400006783.hg.1	-1.95	0.0099	USP17L10	ubiquitin specific peptidase 17-like family member 10
TC0500007531.hg.1	-1.95	0.0219	PDE4D	Memczak2013 ANTISENSE. coding. INTERNAL. intronic best transcript NM_001165899
TC1300009004.hg.1	-1.95	0.0368	ATP7B	ATPase. Cu++ transporting. beta polypeptide
TC0200007978.hg.1	-1.97	0.0089	ANKRD53	ankyrin repeat domain 53
TC0200008811.hg.1	-1.97	0.0463	SH3RF3	SH3 domain containing ring finger 3
TC0700011066.hg.1	-1.98	0.0026	COBL	cordon-bleu WH2 repeat protein
TC0400011535.hg.1	-1.98	0.0141	DKK2	dickkopf WNT signaling pathway inhibitor 2
TC0300013350.hg.1	-1.98	0.0193	YEATS2	Memczak2013 ANTISENSE. coding. INTERNAL. intronic best transcript NM_018023
TC0600011124.hg.1	-1.98	0.0305	HIST1H3B	histone cluster 1. H3b
TC2100007377.hg.1	-1.99	0.0268	KRTAP10-10	keratin associated protein 10-10
TC1000012108.hg.1	-2.01	0.0426	CPXM2	carboxypeptidase X (M14 family). member 2
TC1000012270.hg.1	-2.03	0.0219	EBF3	early B-cell factor 3
TC0500013355.hg.1	-2.03	0.0233	P4HA2	prolyl 4-hydroxylase. alpha polypeptide II
TC0100010255.hg.1	-2.05	0.0132	OR10J4	olfactory receptor. family 10. subfamily J. member 4 (gene/pseudogene) [Source:HGNC Symbol;Acc:HGNC:15408]

TC2200008186.hg.1	-2.06	0.0143	CHCHD10	coiled-coil-helix-coiled-coil-helix domain containing 10
TC2000007936.hg.1	-2.07	0.0041	PHACTR3	phosphatase and actin regulator 3
TC0500013239.hg.1	-2.1	0.0076	PCDHB3	protocadherin beta 3
TC0X00007768.hg.1	-2.1	0.0127	CYLC1	cylicin. basic protein of sperm head cytoskeleton 1
TC1000012198.hg.1	-2.13	0.0004	C10orf90	chromosome 10 open reading frame 90
TC1600009533.hg.1	-2.13	0.0236	MYH11	myosin. heavy chain 11. smooth muscle
TC1700007402.hg.1	-2.15	0.0347	FLOT2	Memczak2013 ANTISENSE. coding. INTERNAL. UTR3 best transcript NM_004475
TC0600008073.hg.1	-2.17	0.031	SRF	serum response factor
TC1100009407.hg.1	-2.19	0.0134	CCDC15	coiled-coil domain containing 15
TC0200016649.hg.1	-2.19	0.0303	CDC42EP3	CDC42 effector protein (Rho GTPase binding) 3
TC1500010360.hg.1	-2.2	0.0145	DET1	de-etiolated homolog 1 (Arabidopsis)
TC0100011091.hg.1	-2.23	0.0088	C1orf53	chromosome 1 open reading frame 53
TC1000008658.hg.1	-2.23	0.0124	PAX2	paired box 2
TC0700009300.hg.1	-2.24	0.002	LOC100130880; AC022173.2	uncharacterized LOC100130880; novel transcript
TC0200011803.hg.1	-2.26	0.0265	NBAS	neuroblastoma amplified sequence
TC1600009962.hg.1	-2.33	0.0072	TBC1D10B	TBC1 domain family. member 10B
TC0800006488.hg.1	-2.37	0.0336	ARHGEF10	Jeck2013 ALT_ACCEPTOR. ALT_DONOR. coding. INTERNAL. intronic best transcript NM_014629
TC0100016445.hg.1	-2.39	0.0024	SERPINC1	serpin peptidase inhibitor. clade C (antithrombin). member 1
TC0900011067.hg.1	-2.41	0.0103	OR13C2	olfactory receptor. family 13. subfamily C. member 2
TC1000009985.hg.1	-2.42	0.0348	C10orf113	chromosome 10 open reading frame 113
TC1700010802.hg.1	-2.44	0.0003	MPP2	membrane protein. palmitoylated 2

TC0800012322.hg.1	-2.45	0.0274	CA13	carbonic anhydrase XIII
TC1600010004.hg.1	-2.81	0.0298	ZNF646	Memczak2013 ANTISENSE. coding. INTERNAL. intronic best transcript NM_014699
TC1400010603.hg.1	-2.96	0.0001	TTC6	tetratricopeptide repeat domain 6

(B) CXCL8 treated T cells

ID	FC CXCL8 vs CONTROL	P-val	Gene Symbol	Description
TC0100010925.hg.1	3.18	0.0179	OCLM	oculomedin
TC0900006881.hg.1	2.62	0.0306	IFT74	intraflagellar transport 74
TC0100015793.hg.1	2.44	0.0386	POGZ	Transcript Identified by AceView, Entrez Gene ID(s) 23126
TC0100007512.hg.1	2.29	0.0072	GPR3	G protein-coupled receptor 3
TC1700006611.hg.1	2.19	0.015	CYB5D2	cytochrome b5 domain containing 2
TC1500007757.hg.1	2.06	0.0057	NR2E3	nuclear receptor subfamily 2, group E, member 3
TC0400006862.hg.1	2.03	0.0188	WDR1	Jeck2013 ANTISENSE, CDS, coding, INTERNAL, intronic, OVCODE, OVEXON best transcript NM_017491
TC0500013242.hg.1	1.96	0.0253	PCDHB6	protocadherin beta 6
TC1900009304.hg.1	1.95	0.0437	PIP5K1C	Zhang2013 ALT_ACCEPTOR, ALT_DONOR, coding, INTERNAL, intronic best transcript NM_012398
TSUnmapped00000224.hg.1	1.92	0.0142	ZDHHC3	zinc finger, DHHC-type containing 3
TC1000007081.hg.1	1.9	0.0228	PDSS1	prenyl (decaprenyl) diphosphate synthase, subunit 1

TC0500009409.hg.1	1.89	0.0123	GABRP	gamma-aminobutyric acid (GABA) A receptor, pi
TC0300006793.hg.1	1.84	0.0271	KAT2B	Transcript Identified by AceView, Entrez Gene ID(s) 8850
TC1200012512.hg.1	1.83	0.0395	DDX51	DEAD (Asp-Glu-Ala-Asp) box polypeptide 51
TC0300012880.hg.1	1.8	0.0101	C3orf33	chromosome 3 open reading frame 33
TC1500007238.hg.1	1.75	0.0343	TMOD2	tropomodulin 2 (neuronal)
TC0100016155.hg.1	1.75	0.0479	USF1	upstream transcription factor 1
TC1200011327.hg.1	1.74	0.0209	CSRP2	cysteine and glycine-rich protein 2
TC0X00010113.hg.1	1.73	0.0266	MAGEE2	MAGE family member E2
TC0200010902.hg.1	1.73	0.044	MOGAT1	monoacylglycerol O-acyltransferase 1
TC1200008642.hg.1	1.72	0.0196	PARPBP	PARP1 binding protein
TSUnmapped00000117.hg.1	1.71	0.0133	ZNF660	zinc finger protein 660
TC0800012452.hg.1	1.7	0.006	EIF3E	eukaryotic translation initiation factor 3, subunit E
TC0700009522.hg.1	1.7	0.0106	OR2A14; OR2A2	olfactory receptor, family 2, subfamily A, member 14; olfactory receptor, family 2, subfamily A, member 2
TC0200010445.hg.1	1.7	0.0458	CASP10	caspase 10
TC1900007004.hg.1	1.69	0.0039	C19orf52	chromosome 19 open reading frame 52
TC0200012031.hg.1	1.69	0.0205	HADHA	Transcript Identified by AceView, Entrez Gene ID(s) 3030
TC1500008757.hg.1	1.68	0.0128	ATP10A	ATPase, class V, type 10A
TC0X00011318.hg.1	1.67	0.0156	TMSB15B	thymosin beta 15B
TC0200008089.hg.1	1.66	0.0386	TLX2	T-cell leukemia homeobox 2
TC1900010482.hg.1	1.65	0.0053	ATP4A	ATPase, H+/K+ exchanging, alpha polypeptide
TC1900007067.hg.1	1.64	0.0098	ZNF136	zinc finger protein 136
TC1900012026.hg.1	1.64	0.0392	ZNF468	zinc finger protein 468
TC1900011327.hg.1	1.64	0.0468	ZNF160	zinc finger protein 160

TC0400009935.hg.1	1.64	0.0482	CCDC96	coiled-coil domain containing 96
TC0X00009029.hg.1	1.63	0.0259	FAM9A	family with sequence similarity 9, member A
TC0700012443.hg.1	1.63	0.0335	FEZF1	FEZ family zinc finger 1
TC1300010005.hg.1	1.62	0.0111	MCF2L	MCF.2 cell line derived transforming sequence like
TC0500011433.hg.1	1.61	0.0103	CETN3	centrin 3
TC0800007377.hg.1	1.61	0.0244	ADAM18	ADAM metallopeptidase domain 18
TC0X00010875.hg.1	1.6	0.0269	PLAC1	placenta specific 1
TC1100010984.hg.1	1.59	0.0253	OR10V1	olfactory receptor, family 10, subfamily V, member 1
TC1800007595.hg.1	1.59	0.0469	CCDC102B	coiled-coil domain containing 102B
TC0X00007937.hg.1	1.58	0.0367	DRP2	dystrophin related protein 2
TC1900009765.hg.1	1.56	0.0482	LYL1	lymphoblastic leukemia associated hematopoiesis regulator 1
TSUnmapped00000384.hg.1	1.55	0.0046	ZNF546	zinc finger protein 546
TC1100008138.hg.1	1.55	0.0229	CABP4	calcium binding protein 4
TC0800012475.hg.1	1.55	0.0302	JRK	Jrk helix-turn-helix protein
TC2000007341.hg.1	1.55	0.0481	FAM83D	family with sequence similarity 83, member D
TC1000010439.hg.1	1.54	0.0356	HNRNPF	Memczak2013 ALT_ACCEPTOR, ALT_DONOR, coding, INTERNAL, intronic best transcript NM_001098207
TSUnmapped00000120.hg.1	1.54	0.0478	ATG16L1	autophagy related 16-like 1
TC0800012351.hg.1	1.53	0.0229	WDYHV1	WDYHV motif containing 1
TC0200007606.hg.1	1.53	0.0312	C2orf73	chromosome 2 open reading frame 73
TC1600006752.hg.1	1.52	0.0166	NUDT16L1	nudix hydrolase 16 like 1
TC0200007071.hg.1	1.52	0.0207	GCKR	Transcript Identified by AceView, Entrez Gene ID(s) 2646

TC1200011873.hg.1	1.52	0.0402	MMAB	methylmalonic aciduria (cobalamin deficiency) cblB type
TC0100009958.hg.1	1.49	0.0309	CGN	cingulin
TC1100011283.hg.1	1.49	0.0438	RIN1	Ras and Rab interactor 1
TC0700013585.hg.1	1.48	0.0188	LRRD1	leucine-rich repeats and death domain containing 1
TC0100017664.hg.1	1.48	0.0217	NUP133	nucleoporin 133kDa
TC0500013300.hg.1	1.48	0.0358	C9	complement component 9
TC1900008326.hg.1	1.47	0.042	FOSB	FBJ murine osteosarcoma viral oncogene homolog B
TC1800007394.hg.1	1.46	0.0275	ST8SIA3	ST8 alpha-N-acetyl-neuraminide alpha-2,8-sialyltransferase 3
TC0200016332.hg.1	1.46	0.0307	KIF1A	kinesin family member 1A
TC1900010172.hg.1	1.45	0.012	ZNF676	zinc finger protein 676
TC1900012008.hg.1	1.45	0.0307	SIGLEC11	sialic acid binding Ig-like lectin 11
TC1100007184.hg.1	1.45	0.0438	DNAJC24	DnaJ (Hsp40) homolog, subfamily C, member 24
TC1900008445.hg.1	1.43	0.0344	ZNF114	zinc finger protein 114
TC1900010622.hg.1	1.42	0.0371	LGALS7; LGALS7B	lectin, galactoside-binding, soluble, 7; lectin, galactoside-binding, soluble, 7B
TC0600008515.hg.1	1.42	0.0415	KCNQ5	Jeck2013 ALT_ACCEPTOR, ALT_DONOR, coding, INTERNAL, intronic best transcript NM_001160133
TC1600007985.hg.1	1.4	0.0387	CETP	cholesteryl ester transfer protein, plasma
TC1100010524.hg.1	-1.4	0.0496	APIP	Transcript Identified by AceView, Entrez Gene ID(s) 51074
TC1200012578.hg.1	-1.41	0.0085	KCNA6; GALNT8	potassium channel, voltage gated shaker related subfamily A, member 6; polypeptide N-acetylgalactosaminyltransferase 8
TC0500007396.hg.1	-1.41	0.0492	SNX18	sorting nexin 18
TC0500007050.hg.1	-1.42	0.0157	PDZD2	PDZ domain containing 2
TC0800012284.hg.1	-1.42	0.0183	EXTL3	exostosin-like glycosyltransferase 3

TC1300007232.hg.1	-1.42	0.0271	CCDC70	coiled-coil domain containing 70
TC1200007356.hg.1	-1.42	0.0348	MUC19	mucin 19, oligomeric
TC0800011697.hg.1	-1.43	0.0163	FER1L6-AS1	FER1L6 antisense RNA 1
TC0100015862.hg.1	-1.43	0.0179	S100A7	S100 calcium binding protein A7
TC1300008072.hg.1	-1.43	0.026	SPACA7	sperm acrosome associated 7
TC0500009303.hg.1	-1.43	0.0287	GABRA1	gamma-aminobutyric acid (GABA) A receptor, alpha 1
TC0500013246.hg.1	-1.43	0.0316	PCDHB10	protocadherin beta 10
TC0700013586.hg.1	-1.43	0.0404	C7orf76; SHFM1	chromosome 7 open reading frame 76; split hand/foot malformation (ectrodactyly) type 1
TC0Y00006512.hg.1	-1.45	0.0157	TSPY2	testis specific protein, Y-linked 2
TC0200015023.hg.1	-1.45	0.0363	EVX2	even-skipped homeobox 2
TC0500009185.hg.1	-1.45	0.0411	KIF4B	kinesin family member 4B
TC0800011015.hg.1	-1.45	0.0462	PSKH2	protein serine kinase H2
TC1700010802.hg.1	-1.46	0.0211	MPP2	membrane protein, palmitoylated 2
TC0100010082.hg.1	-1.46	0.024	TDRD10	tudor domain containing 10
TC1600008329.hg.1	-1.47	0.0401	HP	haptoglobin
TC0200013108.hg.1	-1.48	0.0161	LBX2	ladybird homeobox 2
TC2200009151.hg.1	-1.48	0.0287	MAPK11	mitogen-activated protein kinase 11
TC0600006779.hg.1	-1.48	0.0387	KU-MEL-3; RP4-529N6.1	uncharacterized LOC497048; uncharacterized LOC497048 [Source:EntrezGene;Acc:497048]; putative novel transcript
TC0Y00006812.hg.1	-1.48	0.0433	DAZ2; DAZ4	deleted in azoospermia 2; deleted in azoospermia 4
TC0800007886.hg.1	-1.5	0.0062	PREX2	phosphatidylinositol-3,4,5-trisphosphate-dependent Rac exchange factor 2
TC0400009080.hg.1	-1.5	0.0222	NPY2R	neuropeptide Y receptor Y2
TC1900010988.hg.1	-1.5	0.0268	PNMAL1	paraneoplastic Ma antigen family-like 1
TC1700010658.hg.1	-1.5	0.0303	KRTAP4-2	keratin associated protein 4-2
TC0500012164.hg.1	-1.5	0.0387	GFRA3	GDNF family receptor alpha 3

TC1200012534.hg.1	-1.5	0.0458	POLE	Transcript Identified by AceView, Entrez Gene ID(s) 5426
TC1300007752.hg.1	-1.51	0.0275	CLDN10	claudin 10
TC0700012813.hg.1	-1.51	0.0438	DENND2A	DENN/MADD domain containing 2A
TC0200011273.hg.1	-1.51	0.0479	ESPNL	espin-like
TC0200007047.hg.1	-1.52	0.0265	DPYSL5	dihydropyrimidinase-like 5
TC1800007233.hg.1	-1.52	0.045	KATNAL2	katanin p60 subunit A-like 2
TC0600012329.hg.1	-1.52	0.0456	IMPG1	interphotoreceptor matrix proteoglycan 1
TC1300008086.hg.1	-1.52	0.0458	ATP11A	ATPase, class VI, type 11A
TC1500006562.hg.1	-1.52	0.049	NPAP1	nuclear pore associated protein 1
TC1200009914.hg.1	-1.53	0.0145	TAS2R7	taste receptor, type 2, member 7
TC2100006980.hg.1	-1.53	0.0236	ITSN1	Transcript Identified by AceView, Entrez Gene ID(s) 6453
TC1800007727.hg.1	-1.53	0.0299	LOC400661; AC034110.1	uncharacterized LOC400661; novel transcript
TC1700007704.hg.1	-1.54	0.0065	ARHGAP23	Rho GTPase activating protein 23
TC0200012776.hg.1	-1.54	0.0258	TMEM17	transmembrane protein 17
TC1000007007.hg.1	-1.54	0.0465	ARMC3	armadillo repeat containing 3
TC0400008083.hg.1	-1.54	0.0485	DSPP	dentin sialophosphoprotein
TC0100009408.hg.1	-1.55	0.021	PIFO	primary cilia formation
TSUnmapped00000627.hg.1	-1.55	0.0315	KIF15	kinesin family member 15
TC1100010850.hg.1	-1.55	0.0405	OR5T2	olfactory receptor, family 5, subfamily T, member 2

TC1200011543.hg.1	-1.55	0.0474	PGAM1	Homo sapiens phosphoglycerate mutase 1 (brain), mRNA (cDNA clone MGC:8462 IMAGE:2821567), complete cds.; Homo sapiens phosphoglycerate mutase 1 (brain), mRNA (cDNA clone MGC:19732 IMAGE:3604026), complete cds.; Homo sapiens phosphoglycerate mutase 1 (brain), mRNA (cDNA clone MGC:15086 IMAGE:4299008), complete cds.; Homo sapiens phosphoglycerate mutase 1 (brain), mRNA (cDNA clone MGC:61519 IMAGE:5787678), complete cds.; Homo sapiens phosphoglycerate mutase 1 (brain), mRNA (cDNA clone MGC:71323 IMAGE:6584466), complete cds.; Homo sapiens phosphoglycerate mutase 1 (brain), mRNA (cDNA clone MGC:87445 IMAGE:5262935), complete cds.; Homo sapiens phosphoglycerate mutase 1 (brain), mRNA (cDNA clone MGC:88744 IMAGE:6297173), complete cds.; Homo sapiens phosphoglycerate mutase 1 (brain), mRNA (cDNA clone MGC:189702 IMAGE:8862699), complete cds.
TC0100008185.hg.1	-1.56	0.0124	CYP4A22	cytochrome P450, family 4, subfamily A, polypeptide 22
TC0100015401.hg.1	-1.56	0.0168	IGSF3	immunoglobulin superfamily, member 3
TC2100006883.hg.1	-1.56	0.0195	KRTAP20-3	keratin associated protein 20-3
TC1900009896.hg.1	-1.56	0.0405	CYP4F11	cytochrome P450, family 4, subfamily F, polypeptide 11
TC0400007901.hg.1	-1.56	0.0447	SHROOM3	shroom family member 3
TC1900010508.hg.1	-1.57	0.0077	CLIP3	CAP-GLY domain containing linker protein 3

TC1000007518.hg.1	-1.57	0.0198	SYT15	synaptotagmin XV
TC0Y00007324.hg.1	-1.57	0.0237	TSPY8	testis specific protein, Y-linked 8
TSUnmapped00000086.hg.1	-1.57	0.0326	SAG	S-antigen; retina and pineal gland (arrestin)
TC1700012410.hg.1	-1.58	0.0261	KRTAP4-3	keratin associated protein 4-3
TC2100007888.hg.1	-1.58	0.0323	KRTAP23-1	keratin associated protein 23-1
TC1100013045.hg.1	-1.58	0.0338	SIPA1	signal-induced proliferation-associated 1
TC0300008332.hg.1	-1.58	0.0391	TAGLN3	transgelin 3
TC1100012314.hg.1	-1.59	0.012	PIH1D2	PIH1 domain containing 2
TC0500007258.hg.1	-1.59	0.0127	GHR	growth hormone receptor
TSUnmapped00000641.hg.1	-1.59	0.0206	PRAMEF2	PRAME family member 2
TC1100011202.hg.1	-1.59	0.0284	TMEM262	transmembrane protein 262
TC0Y00006780.hg.1	-1.59	0.0308	DAZ2; DAZ3	deleted in azoospermia 2; deleted in azoospermia 3
TC1000011973.hg.1	-1.59	0.0426	PRLHR	prolactin releasing hormone receptor
TC1600011517.hg.1	-1.6	0.0067	DOC2A	double C2-like domains, alpha
TC0300010962.hg.1	-1.6	0.023	MYL3	myosin, light chain 3, alkali; ventricular, skeletal, slow
TC0200012409.hg.1	-1.6	0.0278	THADA	Transcript Identified by AceView, Entrez Gene ID(s) 63892
TC0600009343.hg.1	-1.6	0.0416	NKAIN2	Na+/K+ transporting ATPase interacting 2
TC2200008706.hg.1	-1.6	0.0433	FAM227A	family with sequence similarity 227, member A
TC0X00010493.hg.1	-1.61	0.0348	GUCY2F	guanylate cyclase 2F, retinal
TC0X00006489.hg.1	-1.61	0.0433	ARSF	arylsulfatase F
TC0800012297.hg.1	-1.62	0.0498	SMIM19	small integral membrane protein 19
TC1400010604.hg.1	-1.63	0.0149	MIA2	melanoma inhibitory activity 2
TC2200008393.hg.1	-1.63	0.0297	RHBDD3	rhomboid domain containing 3
TC1200012833.hg.1	-1.64	0.0123	PAH	phenylalanine hydroxylase

TC1900009079.hg.1	-1.64	0.043	MIER2	Zhang2013 ALT_ACCEPTOR, ALT_DONOR, coding, INTERNAL, intronic, OVERLAPTX best transcript NM_017550
TC1600007723.hg.1	-1.66	0.0011	GPT2	glutamic pyruvate transaminase (alanine aminotransferase) 2
TC2000008807.hg.1	-1.66	0.0046	DEFB119	defensin, beta 119
TC1100009912.hg.1	-1.66	0.0096	OR52A1	olfactory receptor, family 52, subfamily A, member 1
TC0100018180.hg.1	-1.66	0.0122	CLCNKA	chloride channel, voltage-sensitive Ka
TC0X00010468.hg.1	-1.66	0.037	FRMPD3-AS1	FRMPD3 antisense RNA 1
TC1100007770.hg.1	-1.67	0.0149	MS4A5	membrane-spanning 4-domains, subfamily A, member 5
TSUnmapped00000415.hg.1	-1.68	0.0077	INPP5D	inositol polyphosphate-5-phosphatase D
TC1000012270.hg.1	-1.68	0.0257	EBF3	early B-cell factor 3
TC0100016982.hg.1	-1.68	0.0274	MYBPH	myosin binding protein H
TC0600011131.hg.1	-1.68	0.0288	HIST1H1T	histone cluster 1, H1t
TC0X00008888.hg.1	-1.68	0.0317	PPP2R3B	protein phosphatase 2, regulatory subunit B, beta
TC0Y00006863.hg.1	-1.68	0.0317	PPP2R3B	protein phosphatase 2, regulatory subunit B, beta
TC1900009368.hg.1	-1.68	0.0344	TICAM1	toll-like receptor adaptor molecule 1
TC1200011495.hg.1	-1.69	0.0246	LUM	lumican
TC1100010857.hg.1	-1.7	0.0278	OR5M3	olfactory receptor, family 5, subfamily M, member 3
TC1100009617.hg.1	-1.7	0.0282	GLB1L3	galactosidase beta 1 like 3
TC0100015109.hg.1	-1.7	0.04	LOC101928436; RP11-153F1.1; RP11-347K2.2; teyfawbu	uncharacterized LOC101928436; novel transcript; Transcript Identified by AceView
TC1900011946.hg.1	-1.7	0.0499	FBXO27	F-box protein 27

TC0600013228.hg.1	-1.71	0.0333	SLC2A12	solute carrier family 2 (facilitated glucose transporter), member 12
TC0200010839.hg.1	-1.71	0.0445	TUBA4B	tubulin, alpha 4b
TC1000012198.hg.1	-1.73	0.0071	C10orf90	chromosome 10 open reading frame 90
TC1700010648.hg.1	-1.76	0.0144	KRTAP1-5	keratin associated protein 1-5
TC0700012448.hg.1	-1.76	0.0417	RNF133	ring finger protein 133
TC0900007129.hg.1	-1.77	0.0046	MELK	maternal embryonic leucine zipper kinase
TC0800007284.hg.1	-1.78	0.0068	KCNU1	potassium channel, subfamily U, member 1
TC1400010651.hg.1	-1.78	0.0153	DLK1	delta-like 1 homolog (Drosophila)
TC2100008377.hg.1	-1.78	0.0414	KRTAP10-3	keratin associated protein 10-3
TC0800008349.hg.1	-1.79	0.0215	OSR2	odd-skipped related transciption factor 2
TC0700010912.hg.1	-1.79	0.0344	MYL7	myosin light chain 7
TC0300008847.hg.1	-1.81	0.0217	ACKR4	atypical chemokine receptor 4
TC0300009302.hg.1	-1.82	0.0047	PQLC2L	PQ loop repeat containing 2-like
TC0500009077.hg.1	-1.82	0.025	HMGXB3	HMG box domain containing 3
TC0100013992.hg.1	-1.83	0.0014	TCTEX1D4	Tctex1 domain containing 4
TC0200007978.hg.1	-1.84	0.0163	ANKRD53	ankyrin repeat domain 53
TC1100012677.hg.1	-1.85	0.0078	OR8B12	olfactory receptor, family 8, subfamily B, member 12
TSUnmapped00000810.hg.1	-1.85	0.0253	PRAMEF19	PRAME family member 19 [Source:EntrezGene;Acc:645414]
TC2200008186.hg.1	-1.87	0.0142	CHCHD10	coiled-coil-helix-coiled-coil-helix domain containing 10
TC0X00011241.hg.1	-1.87	0.0348	TMLHE-AS1; LOC101927830; CLIC2; RP13-228J13.1	Homo sapiens TMLHE antisense RNA 1 (TMLHE-AS1), long non-coding RNA.; uncharacterized LOC101927830; Transcript Identified by AceView, Entrez Gene ID(s) 1193; novel transcript
TC0100006910.hg.1	-1.88	0.0176	PRAMEF2	PRAME family member 2

TC0200011890.hg.1	-1.88	0.0184	LAPTM4A	Memczak2013 ALT_ACCEPTOR, ALT_DONOR, coding, INTERNAL, intronic best transcript NM_014713
TC1300009305.hg.1	-1.9	0.0419	LOC100129307	putative UPF0607 protein ENSP00000383144
TC0500011875.hg.1	-1.91	0.0358	PPIC	peptidylprolyl isomerase C (cyclophilin C)
TC1200007365.hg.1	-1,92	0,0043	PDZRN4	PDZ domain containing ring finger 4
TC1200009834.hg.1	-1,92	0,0198	MFAP5	microfibrillar associated protein 5
TC1500009221.hg.1	-1,92	0,0441	TP53BP1	tumor protein p53 binding protein 1
TC0900008842.hg.1	-1,94	0,0077	SLC25A25	Zhang2013 ALT_ACCEPTOR, ALT_DONOR, coding, INTERNAL, intronic best transcript NM_001006641
TC0900010794.hg.1	-1,94	0,0201	OGN	osteoglycin
TC0X00007930.hg.1	-1,94	0,0365	ARL13A	ADP-ribosylation factor like GTPase 13A
TC0200016482.hg.1	-1,96	0,0469	CTNNA2	catenin (cadherin-associated protein), alpha 2
TC1900011913.hg.1	-1,98	0,013	LINC00663	long intergenic non-protein coding RNA 663
TC0400008134.hg.1	-1,98	0,0167	MMRN1	multimerin 1
TC1700007757.hg.1	-1,99	0,0071	GRB7	growth factor receptor bound protein 7
TC1200011491.hg.1	-2	0,0088	EPYC	epiphycan
TC0100012942.hg.1	-2	0,0214	PRAMEF18	PRAME family member 18
TC0X00008826.hg.1	-2	0,023	FLNA	Jeck2013 ANTISENSE, CDS, coding, INTERNAL, OVCODE, OVEXON best transcript NM_001110556
TC0X00009677.hg.1	-2,01	0,0181	AKAP4	A kinase (PRKA) anchor protein 4
TC1400010603.hg.1	-2,03	0,016	TTC6	tetratricopeptide repeat domain 6
TC2000007936.hg.1	-2,07	0,0032	PHACTR3	phosphatase and actin regulator 3
TC1000010135.hg.1	-2,08	0,0379	ARMC4	armadillo repeat containing 4
TC0X00009727.hg.1	-2,1	0,0123	SPANXN5	SPANX family, member N5
TC0300011951.hg.1	-2,12	0,021	MORC1	MORC family CW-type zinc finger 1
TC1500009179.hg.1	-2,16	0,0031	PLA2G4D	phospholipase A2, group IVD (cytosolic)

TC0900009417.hg.1	-2,21	0,0167	RFX3	Memczak2013 ALT_ACCEPTOR, ALT_DONOR, coding, INTERNAL, intronic best transcript NM_002919
TC0300009454.hg.1	-2,24	0,0187	LRRIQ4	leucine-rich repeats and IQ motif containing 4
TC0900007654.hg.1	-2,35	0,0023	GNAQ; skeyplorbu	Memczak2013 ANTISENSE, coding, INTERNAL, intronic best transcript NM_002072; Transcript Identified by AceView
TC0700009668.hg.1	-2,38	0,0455	ZBED6CL	ZBED6 C-terminal like
TC0300007387.hg.1	-2,66	0,0175	RNF123	ring finger protein 123

(C) IL-32α treated T cells

ID	FC IL32 vs CONTROL	P-val	Gene Symbol	Description
TC0100010925.hg.1	4.01	0.0063	OCLM	oculomedin
TC1600006789.hg.1	2.7	0.0171	A2BP1	Transcript Identified by AceView, Entrez Gene ID(s) 54715
TC1700011407.hg.1	2.64	0.0036	CYB561	cytochrome b561
TC1200012512.hg.1	2.48	0.0016	DDX51	DEAD (Asp-Glu-Ala-Asp) box polypeptide 51
TC1100012660.hg.1	2.46	0.0044	OR8D2	olfactory receptor, family 8, subfamily D, member 2 (gene/pseudogene)
TC1300009924.hg.1	2.43	0.0057	GAS6	growth arrest-specific 6
TC1300008091.hg.1	2.36	0.0301	ATP11A	Transcript Identified by AceView, Entrez Gene ID(s) 23250
TC0500013242.hg.1	2.23	0.0346	PCDHB6	protocadherin beta 6
TC2000009090.hg.1	2.21	0.017	DHX35	Memczak2013 ANTISENSE, coding, INTERNAL, intronic best transcript NM_021931
TC0700010358.hg.1	2.15	0.0211	AGR3	anterior gradient 3, protein disulphide isomerase family member
TC0500009109.hg.1	2.1	0.0021	GPX3	glutathione peroxidase 3
TC0800007993.hg.1	2.01	0.0156	PI15	peptidase inhibitor 15
TC1500007802.hg.1	1.98	0.0167	GOLGA6B	golgin A6 family, member B
TC0400012881.hg.1	1.97	0.0038	DUX4	double homeobox 4
TC1900009639.hg.1	1.97	0.0183	AP1M2	adaptor-related protein complex 1, mu 2 subunit
TC1200012516.hg.1	1.96	0.0168	GALNT9	polypeptide N-acetylgalactosaminyltransferase 9
TC1500010718.hg.1	1.91	0.0013	DISP2	dispatched homolog 2 (Drosophila)
TC0700012443.hg.1	1.91	0.0032	FEZF1	FEZ family zinc finger 1

TC0600009426.hg.1	1.91	0.0327	LAMA2	laminin, alpha 2
TC0200016073.hg.1	1.89	0.0356	KCNJ13	potassium channel, inwardly rectifying subfamily J, member 13
TC0400008444.hg.1	1.88	0.0138	LARP7	Zhang2013 ALT_DONOR, coding, INTERNAL, intronic, OVERLAPTX, OVEXON best transcript NM_016648
TC0100008618.hg.1	1.88	0.0189	AK4	adenylate kinase 4
TC0300008713.hg.1	1.86	0.0468	SEC61A1	Sec61 translocon alpha 1 subunit
TC0400008861.hg.1	1.85	0.0484	IL15	interleukin 15
TC0100008183.hg.1	1.83	0.0254	CYP4Z1	cytochrome P450, family 4, subfamily Z, polypeptide 1
TC0400009935.hg.1	1.83	0.045	CCDC96	coiled-coil domain containing 96
TC1700009219.hg.1	1.82	0.0208	RAC3	ras-related C3 botulinum toxin substrate 3 (rho family, small GTP binding protein Rac3)
TC0100015793.hg.1	1.82	0.0283	POGZ	Transcript Identified by AceView, Entrez Gene ID(s) 23126
TC0200010727.hg.1	1.81	0.0242	TMEM169	Transcript Identified by AceView, Entrez Gene ID(s) 92691
TC1000008377.hg.1	1.8	0.0255	LIPJ	lipase, family member J
TC0100018461.hg.1	1.77	0.0107	AKNAD1	AKNA domain containing 1
TC1400008597.hg.1	1.75	0.0322	RNASE12; RNASE11	ribonuclease, RNase A family, 12 (non-active); ribonuclease, RNase A family, 11 (non-active)
TC1200009912.hg.1	1.74	0.0447	STYK1	serine/threonine/tyrosine kinase 1
TC2000008344.hg.1	1.73	0.027	HAO1	hydroxyacid oxidase (glycolate oxidase) 1
TSUnmapped00000532.hg.1	1.73	0.0485	LRIG1	
TC1500007757.hg.1	1.72	0.0164	NR2E3	nuclear receptor subfamily 2, group E, member 3

TC2000009248.hg.1	1.72	0.0493	NEURL2	neuralized E3 ubiquitin protein ligase 2
TC0300012880.hg.1	1.71	0.0209	C3orf33	chromosome 3 open reading frame 33
TC0100018464.hg.1	1.7	0.0021	PPM1J	protein phosphatase, Mg2+/Mn2+ dependent, 1J
TC0700008389.hg.1	1.7	0.0123	ASB4	ankyrin repeat and SOCS box containing 4
TC0200008679.hg.1	1.7	0.0173	SLC9A4	solute carrier family 9, subfamily A (NHE4, cation proton antiporter 4), member 4
TC0500013230.hg.1	1.7	0.019	PCBD2	pterin-4 alpha-carbinolamine dehydratase/dimerization cofactor of hepatocyte nuclear factor 1 alpha (TCF1) 2
TC1100007841.hg.1	1.7	0.0253	SCGB2A2	secretoglobin, family 2A, member 2
TC1900010824.hg.1	1.69	0.0016	PSG11	pregnancy specific beta-1-glycoprotein 11
TC0800009429.hg.1	1.69	0.0157	DEFB1	defensin, beta 1
TC0400012942.hg.1	1.68	0.0075	C4orf36	chromosome 4 open reading frame 36
TC0700006867.hg.1	1.67	0.0204	DNAH11	dynein, axonemal, heavy chain 11
TC1600008738.hg.1	1.67	0.0253	FOXC2	forkhead box C2
TC1400008622.hg.1	1.67	0.0262	OR5AU1	olfactory receptor, family 5, subfamily AU, member 1
TC1300009023.hg.1	1.67	0.0271	LECT1	leukocyte cell derived chemotaxin 1
TC0800009493.hg.1	1.67	0.0428	USP17L3; USP17L8	ubiquitin specific peptidase 17-like family member 3; ubiquitin specific peptidase 17-like family member 8
TC1900011086.hg.1	1.66	0.0143	NTN5	netrin 5
TC1200008804.hg.1	1.66	0.016	FAM222A	family with sequence similarity 222, member A

TC0300009688.hg.1	1.66	0.0444	HTR3E	5-hydroxytryptamine (serotonin) receptor 3E, ionotropic
TC0400012645.hg.1	1.65	0.0266	SORBS2	Transcript Identified by AceView, Entrez Gene ID(s) 8470
TC0X00008940.hg.1	1.64	0.0135	MXRA5	matrix-remodelling associated 5
TC0X00007169.hg.1	1.63	0.0299	SSX6	synovial sarcoma, X breakpoint 6 (pseudogene)
TC1100007610.hg.1	1.62	0.0013	OR5D14	olfactory receptor, family 5, subfamily D, member 14
TC0700009522.hg.1	1.62	0.0274	OR2A14; OR2A2	olfactory receptor, family 2, subfamily A, member 14; olfactory receptor, family 2, subfamily A, member 2
TC1500009738.hg.1	1.6	0.0322	KIAA0101; CSNK1G1	KIAA0101; casein kinase 1, gamma 1
TC0700008760.hg.1	1.6	0.0367	SLC26A4	solute carrier family 26 (anion exchanger), member 4
TC1200007646.hg.1	1.6	0.0413	ACVRL1	activin A receptor type IL
TC0X00006723.hg.1	1.59	0.0059	CDKL5	cyclin-dependent kinase-like 5
TC1500010350.hg.1	1.59	0.026	NTRK3	neurotrophic tyrosine kinase, receptor, type 3
TSUnmapped00000117.hg.1	1.59	0.0322	ZNF660	zinc finger protein 660
TC1100008669.hg.1	1.59	0.0365	PRSS23	protease, serine, 23
TC0800011142.hg.1	1.59	0.0431	LOC100288748; RP11-22C11.2	uncharacterized LOC100288748; novel transcript, antisense to ESRP1
TC0900011277.hg.1	1.57	0.0233	KIF12	kinesin family member 12
TC0200016224.hg.1	1.56	0.0331	PER2	period circadian clock 2
TC0600012188.hg.1	1.55	0.0242	LGSN	lengsin, lens protein with glutamine synthetase domain

TC0X00011159.hg.1	1.55	0.0269	HAUS7; TREX2	HAUS augmin like complex subunit 7; three prime repair exonuclease 2
TC1200008344.hg.1	1.55	0.0307	LRRIQ1	leucine-rich repeats and IQ motif containing 1
TC1700008851.hg.1	1.54	0.0355	GPRC5C	G protein-coupled receptor, class C, group 5, member C
TC1200012679.hg.1	1.54	0.0473	ACSS3	acyl-CoA synthetase short-chain family member 3
TC0500012605.hg.1	1.53	0.03	SOX30	SRY box 30
TC2200008515.hg.1	1.53	0.05	BPIFC	BPI fold containing family C
TC0100009509.hg.1	1.52	0.0199	SYCP1	synaptonemal complex protein 1
TC0200009871.hg.1	1.52	0.0291	CSRNP3	cysteine-serine-rich nuclear protein 3
TC1500008170.hg.1	1.52	0.0367	ALPK3	alpha kinase 3
TC0800009886.hg.1	1.52	0.0496	NKX2-6	NK2 homeobox 6
TC2000006829.hg.1	1.51	0.0057	POLR3F	polymerase (RNA) III (DNA directed) polypeptide F, 39 kDa
TC0200009167.hg.1	1.51	0.0307	CNTNAP5	contactin associated protein-like 5
TC0700010412.hg.1	1.51	0.0368	SP8	Sp8 transcription factor
TC0X00009185.hg.1	1.51	0.0381	SCML2	sex comb on midleg-like 2 (Drosophila)
TC0500011433.hg.1	1.5	0.0185	CETN3	centrin 3
TC1600008139.hg.1	1.5	0.0293	CES4A	carboxylesterase 4A
TC0400011503.hg.1	1.5	0.0326	PPA2	pyrophosphatase (inorganic) 2
TC1700010408.hg.1	1.5	0.0349	CCT6B	chaperonin containing TCP1, subunit 6B (zeta 2)
TC1900007004.hg.1	1.46	0.0033	C19orf52	chromosome 19 open reading frame 52
TC0400008160.hg.1	1.46	0.0203	GRID2	glutamate receptor, ionotropic, delta 2
TSUnmapped00000438.hg.1	1.46	0.0249	HMBS	hydroxymethylbilane synthase

TC2200006955.hg.1	1.46	0.0317	CRYBA4	crystallin beta A4
TC2000009917.hg.1	1.46	0.0338	BPI	bactericidal/permeability-increasing protein
TC1700012433.hg.1	1.45	0.0126	HOXB6	homeobox B6
TC2000007973.hg.1	1.45	0.0154	CDH4	cadherin 4, type 1, R-cadherin (retinal)
TC0600010520.hg.1	1.45	0.0385	MYLK4	myosin light chain kinase family member 4
TC1400010753.hg.1	1.45	0.0461	SLC35F4	solute carrier family 35, member F4
TC2200009356.hg.1	1.45	0.0483	ARFGAP3	ADP-ribosylation factor GTPase activating protein 3
TC0100011621.hg.1	1.45	0.0484	TGFB2; TGFB2-OT1	transforming growth factor beta 2; TGFB2 overlapping transcript 1
TC1200009941.hg.1	1.44	0.0205	PRB3	proline-rich protein BstNI subfamily 3
TC1000011023.hg.1	1.44	0.0372	MSS51	MSS51 mitochondrial translational activator
TC1800007358.hg.1	1.43	0.0488	DYNAP	dynactin associated protein
TC0400006714.hg.1	1.41	0.0193	TADA2B	Transcript Identified by AceView, Entrez Gene ID(s) 93624
TC0900012139.hg.1	1.41	0.0254	PGM5	phosphoglucomutase 5
TC0800012475.hg.1	1.41	0.0473	JRK	Jrk helix-turn-helix protein
TC0100013382.hg.1	-1.4	0.0481	AIM1L	absent in melanoma 1-like
TC1100007620.hg.1	-1.4	0.0369	OR5AS1	olfactory receptor, family 5, subfamily AS, member 1
TC1400007168.hg.1	-1.4	0.0079	GPR137C	G protein-coupled receptor 137C
TC1500007237.hg.1	-1.41	0.0421	SCG3	secretogranin III
TC0200013003.hg.1	-1.42	0.0377	FIGLA	folliculogenesis specific bHLH transcription factor
TC1100009912.hg.1	-1.42	0.045	OR52A1	olfactory receptor, family 52, subfamily A, member 1
TC1900012000.hg.1	-1.42	0.0153	TEAD2	TEA domain family member 2

TC2200008528.hg.1	-1.42	0.0266	LARGE	like-glycosyltransferase
TC0100016121.hg.1	-1.43	0.032	KCNJ10	potassium channel, inwardly rectifying subfamily J, member 10
TC1600007723.hg.1	-1.44	0.0107	GPT2	glutamic pyruvate transaminase (alanine aminotransferase) 2
TC0200012417.hg.1	-1.45	0.0233	ABCG5	ATP binding cassette subfamily G member 5
TC0500007396.hg.1	-1.45	0.033	SNX18	sorting nexin 18
TC0900008665.hg.1	-1.45	0.0152	OR1J2	olfactory receptor, family 1, subfamily J, member 2
TC1300008766.hg.1	-1.45	0.0428	ENOX1	ecto-NOX disulfide-thiol exchanger 1
TC0700013617.hg.1	-1.46	0.0209	GCC1	GRIP and coiled-coil domain containing 1
TC1900011908.hg.1	-1.46	0.0071	PDE4C	phosphodiesterase 4C, cAMP-specific
TC2000008807.hg.1	-1.46	0.0142	DEFB119	defensin, beta 119
TC0100016902.hg.1	-1.47	0.0227	ASCL5	achaete-scute family bHLH transcription factor 5
TC0200015023.hg.1	-1.47	0.0148	EVX2	even-skipped homeobox 2
TC1000009454.hg.1	-1.47	0.0254	ADAM8	Memczak2013 ANTISENSE, CDS, coding, INTERNAL best transcript NM_001109
TC1700008440.hg.1	-1.47	0.0231	PPM1E	protein phosphatase, Mg2+/Mn2+ dependent, 1E
TC0100013246.hg.1	-1.48	0.0455	WNT4	wingless-type MMTV integration site family, member 4
TC2200009107.hg.1	-1.48	0.0369	C22orf34	chromosome 22 open reading frame 34
TSUnmapped00000292.hg.1	-1.48	0.0244	TGM4	transglutaminase 4
TC0500009743.hg.1	-1.49	0.0214	BTNL8	butyrophilin-like 8

TC1200009239.hg.1	-1.5	0.0201	KMT5A	lysine (K)-specific methyltransferase 5A
TC0400011429.hg.1	-1.51	0.0279	DDIT4L	DNA-damage-inducible transcript 4-like
TC1300008086.hg.1	-1.51	0.0391	ATP11A	ATPase, class VI, type 11A
TC0100010905.hg.1	-1.52	0.0199	SWT1	SWT1 RNA endoribonuclease homolog
TC0300013915.hg.1	-1.52	0.0327	TPRG1	tumor protein p63 regulated 1
TC0700012605.hg.1	-1.52	0.0334	COPG2; TSGA13	coatomer protein complex subunit gamma 2; testis specific 13
TC1900011679.hg.1	-1.52	0.0288	CYP4F3	cytochrome P450, family 4, subfamily F, polypeptide 3
TC0200008573.hg.1	-1.54	0.0397	UNC50	unc-50 homolog (C. elegans)
TC1100009384.hg.1	-1.54	0.0374	OR8G1	olfactory receptor, family 8, subfamily G, member 1 (gene/pseudogene)
TC1600008170.hg.1	-1.54	0.042	C16orf86	chromosome 16 open reading frame 86
TC0100018454.hg.1	-1.55	0.0099	ARHGAP29	Rho GTPase activating protein 29
TC0700013598.hg.1	-1.55	0.0491	LRCH4	leucine-rich repeats and calponin homology (CH) domain containing 4
TC1100011914.hg.1	-1.55	0.0318	TRIM64B	tripartite motif containing 64B
TC1400008070.hg.1	-1.55	0.0236	SERPINA13P	serpin peptidase inhibitor, clade A (alpha-1 antiproteinase, antitrypsin), member 13, pseudogene
TC1800007233.hg.1	-1.55	0.0263	KATNAL2	katanin p60 subunit A-like 2
TC0900010333.hg.1	-1.56	0.0079	PRKACG	protein kinase, cAMP-dependent, catalytic, gamma
TC1400008857.hg.1	-1.56	0.0167	GPR33	G protein-coupled receptor 33 (gene/pseudogene)

TC1500007839.hg.1	-1.56	0.0266	ISLR	immunoglobulin superfamily containing leucine-rich repeat
TC0100011091.hg.1	-1.57	0.0164	C1orf53	chromosome 1 open reading frame 53
TC1700010276.hg.1	-1.57	0.0158	TMIGD1	transmembrane and immunoglobulin domain containing 1
TC1200010050.hg.1	-1.58	0.0397	LMO3	LIM domain only 3 (rhombotin-like 2)
TC1500008002.hg.1	-1.58	0.0307	ANKRD34C	ankyrin repeat domain 34C
TC1800007727.hg.1	-1.58	0.0422	LOC400661; AC034110.1	uncharacterized LOC400661; novel transcript
TC0100010621.hg.1	-1.59	0.0073	C1orf105	chromosome 1 open reading frame 105
TC1600008008.hg.1	-1.59	0.0152	DRC7	dynein regulatory complex subunit 7
TC1700008421.hg.1	-1.59	0.0498	OR4D1	olfactory receptor, family 4, subfamily D, member 1
TC1700010856.hg.1	-1.59	0.0411	C1QL1	complement component 1, q subcomponent-like 1
TC2000008445.hg.1	-1.59	0.0025	FLRT3	fibronectin leucine rich transmembrane protein 3
TC2200008507.hg.1	-1.59	0.0489	SLC5A4	solute carrier family 5 (glucose activated ion channel), member 4
TC0300008029.hg.1	-1.6	0.0068	EPHA3	EPH receptor A3
TC0400012019.hg.1	-1.6	0.0317	GYPA	glycophorin A (MNS blood group)
TC0700011066.hg.1	-1.6	0.0358	COBL	cordon-bleu WH2 repeat protein
TSUnmapped00000639.hg.1	-1.6	0.0249	SERTAD4	SERTA domain containing 4
TC0800009466.hg.1	-1.61	0.0328	DEFB104A; DEFB104B	defensin, beta 104A; defensin, beta 104B
TC0100006931.hg.1	-1.62	0.0218	PDPN	podoplanin

TC0600007623.hg.1	-1.63	0.0254	SKIV2L	SKI2 homolog, superkiller viralicidic activity 2-like
TC1000012270.hg.1	-1.63	0.0226	EBF3	early B-cell factor 3
TC1700006627.hg.1	-1.63	0.0207	SPNS2	spinster homolog 2 (Drosophila)
TC1700012410.hg.1	-1.64	0.0191	KRTAP4-3	keratin associated protein 4-3
TC2100006883.hg.1	-1.64	0.0028	KRTAP20-3	keratin associated protein 20-3
TC0X00011144.hg.1	-1.65	0.0019	PNMA5	paraneoplastic Ma antigen family member 5
TC0100015764.hg.1	-1.66	0.0094	FAM63A	family with sequence similarity 63, member A
TC0200013668.hg.1	-1.66	0.0243	CREG2	cellular repressor of E1A-stimulated genes 2
TC0900009139.hg.1	-1.66	0.0368	FCN2	ficolin (collagen/fibrinogen domain containing lectin) 2
TC1000011973.hg.1	-1.66	0.0442	PRLHR	prolactin releasing hormone receptor
TSUnmapped00000630.hg.1	-1.66	0.0272	ZNF780A	zinc finger protein 780A
TC0300007014.hg.1	-1.67	0.0298	ARPP21	Transcript Identified by AceView, Entrez Gene ID(s) 10777
TC1400010604.hg.1	-1.67	0.0073	MIA2	melanoma inhibitory activity 2
TC1900006770.hg.1	-1.67	0.0223	DUS3L	Memczak2013 ANTISENSE, CDS, coding, INTERNAL best transcript NM_020175
TSUnmapped00000412.hg.1	-1.67	0.0375	BCL2L14	BCL2-like 14 (apoptosis facilitator)
TC0100014109.hg.1	-1.68	0.0479	TRABD2B	TraB domain containing 2B
TC0500009185.hg.1	-1.68	0.018	KIF4B	kinesin family member 4B
TC1900009517.hg.1	-1.68	0.0203	FBN3	fibrillin 3
TC0700013254.hg.1	-1.7	0.0308	PTPRN2	protein tyrosine phosphatase, receptor type, N polypeptide 2
TC0600007985.hg.1	-1.72	0.033	TSPO2	translocator protein 2

TC1900011976.hg.1	-1.72	0.0464	ZNF285	zinc finger protein 285
TC0400011535.hg.1	-1.73	0.0427	DKK2	dickkopf WNT signaling pathway inhibitor 2
TC0400012060.hg.1	-1.73	0.0037	TTC29	tetratricopeptide repeat domain 29
TC1900011201.hg.1	-1.73	0.0029	FAM71E1	family with sequence similarity 71, member E1
TC0100013723.hg.1	-1.74	0.0443	MRPS15	mitochondrial ribosomal protein S15
TC0600014279.hg.1	-1.74	0.0059	DAXX	death-domain associated protein
TC1100010850.hg.1	-1.74	0.0324	OR5T2	olfactory receptor, family 5, subfamily T, member 2
TC1900011946.hg.1	-1.74	0.0352	FBXO27	F-box protein 27
TC1300008080.hg.1	-1.75	0.0445	TUBGCP3	Memczak2013 ANTISENSE, coding, INTERNAL, intronic best transcript NM_006322
TC0700011010.hg.1	-1.77	0.0195	SUN3	Sad1 and UNC84 domain containing 3
TC0300011951.hg.1	-1.78	0.0168	MORC1	MORC family CW-type zinc finger 1
TC0500009077.hg.1	-1.78	0.0494	HMGXB3	HMG box domain containing 3
TC1100011202.hg.1	-1.79	0.0088	TMEM262	transmembrane protein 262
TC0500013370.hg.1	-1.8	0.0089	SIL1	SIL1 nucleotide exchange factor
TC0900009855.hg.1	-1.8	0.0108	BAG1	BCL2-associated athanogene
TC0100010121.hg.1	-1.82	0.0237	HCN3	hyperpolarization activated cyclic nucleotide gated potassium channel 3
TC0300013813.hg.1	-1.82	0.0018	ACKR2	atypical chemokine receptor 2
TC0300010739.hg.1	-1.84	0.0089	PLCD1	phospholipase C, delta 1
TC1600006686.hg.1	-1.84	0.0296	OR2C1	olfactory receptor, family 2, subfamily C, member 1
TC1900008111.hg.1	-1.85	0.0434	SPTBN4	spectrin, beta, non-erythrocytic 4

TC0700009300.hg.1	-1.86	0.0012	LOC100130880; AC022173.2	uncharacterized LOC100130880; novel transcript
TSUnmapped00000086.hg.1	-1.87	0.0251	SAG	S-antigen; retina and pineal gland (arrestin)
TC0400006783.hg.1	-1.9	0.0092	USP17L10	ubiquitin specific peptidase 17-like family member 10
TC2000009588.hg.1	-1.9	0.0247	MTRNR2L3	MT-RNR2-like 3
TC0100015805.hg.1	-1.91	0.0112	CELF3	CUGBP, Elav-like family member 3
TC1300008072.hg.1	-1.91	0.0037	SPACA7	sperm acrosome associated 7
TC0600011131.hg.1	-1.93	0.0082	HIST1H1T	histone cluster 1, H1t
TC0500013385.hg.1	-1.94	0.0008	SH3TC2	SH3 domain and tetratricopeptide repeats 2
TC1100007774.hg.1	-1.95	0.0369	MS4A13	membrane-spanning 4-domains, subfamily A, member 13
TC1700009960.hg.1	-1.95	0.0374	ATPAF2	ATP synthase mitochondrial F1 complex assembly factor 2
TC0200006703.hg.1	-1.99	0.0113	ATP6V1C2	ATPase, H+ transporting, lysosomal 42kDa, V1 subunit C2
TC0500007901.hg.1	-1.99	0.0404	THBS4	thrombospondin 4
TC1500007645.hg.1	-1.99	0.031	MAP2K5	mitogen-activated protein kinase kinase 5
TC2000006441.hg.1	-2	0.0282	SOX12	SRY box 12
TC0300013350.hg.1	-2.01	0.0478	YEATS2	Memczak2013 ANTISENSE, coding, INTERNAL, intronic best transcript NM_018023
TC1000012198.hg.1	-2.02	0.0204	C10orf90	chromosome 10 open reading frame 90
TC0200013627.hg.1	-2.03	0.0436	AFF3	Memczak2013 ALT_ACCEPTOR, ALT_DONOR, coding, INTERNAL, intronic best transcript NM_001025108

TC0200016026.hg.1	-2.04	0.0396	NMUR1	neuromedin U receptor 1
TC0500009012.hg.1	-2.04	0.0446	TCERG1	Transcript Identified by AceView, Entrez Gene ID(s) 10915
TC0800008663.hg.1	-2.13	0.0322	MAL2	mal, T-cell differentiation protein 2 (gene/pseudogene)
TC0200007978.hg.1	-2.16	0.0175	ANKRD53	ankyrin repeat domain 53
TC1700010802.hg.1	-2.18	0.0005	MPP2	membrane protein, palmitoylated 2
TC0800009941.hg.1	-2.2	0.035	PNMA2	paraneoplastic Ma antigen 2
TC1900008604.hg.1	-2.2	0.0011	MGC45922; CTD-2568A17.5	uncharacterized LOC284365; uncharacterized LOC284365 [Source:EntrezGene;Acc:284365]; novel transcript
TC1400007632.hg.1	-2.29	0.0347	PAPLN	papilin, proteoglycan-like sulfated glycoprotein
TC0100010397.hg.1	-2.42	0.0401	NUF2	NUF2, NDC80 kinetochore complex component

(D) IL-15 treated T cells

ID	FC IL15 vs CONTROL	P-val	Gene Symbol	Description
TC1200008474.hg.1	9.94	0.000000134	SOCS2	suppressor of cytokine signaling 2
TC0600007596.hg.1	8.8	0.0019	TNF	tumor necrosis factor
TC1700010461.hg.1	8.78	0.0327	CCL3L3; CCL3L1	chemokine (C-C motif) ligand 3-like 3; chemokine (C-C motif) ligand 3-like 1
TC0100012734.hg.1	7.42	0.0412	TNFRSF9	tumor necrosis factor receptor superfamily, member 9
TC1400010195.hg.1	6.66	0.0365	WARS	tryptophanyl-tRNA synthetase
TC0600006442.hg.1	6.12	0.00000289	IRF4	interferon regulatory factor 4
TC0100014857.hg.1	5.19	0.0011	GBP5	guanylate binding protein 5
TC0400011052.hg.1	5.07	0.0029	CXCL9	chemokine (C-X-C motif) ligand 9
TC1200007461.hg.1	4.84	0.0204	PCED1B	PC-esterase domain containing 1B
TC1800008891.hg.1	4.63	0.0012	BCL2	B-cell CLL/lymphoma 2
TC0X00009667.hg.1	4.14	0.0002	FOXP3	forkhead box P3
TC1400007165.hg.1	4.01	0.000000186	PTGER2	prostaglandin E receptor 2
TC0300013555.hg.1	3.83	0.0066	MB21D2	Mab-21 domain containing 2
TC1200007822.hg.1	3.68	0.0069	IKZF4	IKAROS family zinc finger 4
TC0600007862.hg.1	3.61	0.0027	PIM1	Pim-1 proto-oncogene, serine/threonine kinase
TC1000008891.hg.1	3.59	0.0021	DUSP5	dual specificity phosphatase 5
TC0700011785.hg.1	3.58	0.0002	CDK6	cyclin-dependent kinase 6
TC0100014769.hg.1	3.55	0.0044	MCOLN3	mucolipin 3
TC0100014852.hg.1	3.51	0.0007	GBP1	guanylate binding protein 1, interferon-inducible
TC1900010807.hg.1	3.39	0.00000907	CEACAM1	carcinoembryonic antigen-related cell adhesion molecule 1 (biliary glycoprotein)
TC0600012036.hg.1	3.29	0.0387	IL17F	interleukin 17F

TC0100014930.hg.1	3.26	0.0397	EVI5	Transcript Identified by AceView, Entrez Gene ID(s) 7813
TC0100012453.hg.1	3.15	0.0057	TNFRSF4	tumor necrosis factor receptor superfamily, member 4
TC0300011124.hg.1	28.21	0.0000 00015	CISH	cytokine inducible SH2-containing protein
TC0200016505.hg.1	2.98	0.0000 955	CCDC138	coiled-coil domain containing 138
TC0100011012.hg.1	2.93	0.0184	RGS1	regulator of G-protein signaling 1
TC1800008580.hg.1	2.86	0.0267	PSTPIP2	proline-serine-threonine phosphatase interacting protein 2
TC0600009439.hg.1	2.77	0.0001	TMEM200A	transmembrane protein 200A
TC0300012123.hg.1	2.72	0.0214	CD80	CD80 molecule
TC0300011259.hg.1	2.71	0.0048	ARHGEF3	Rho guanine nucleotide exchange factor 3
TC0300010399.hg.1	2.68	0.0384	SH3BP5	SH3-domain binding protein 5 (BTK-associated)
TC0900006559.hg.1	2.66	0.0113	CD274	CD274 molecule
TC0800010685.hg.1	2.65	0.0324	MYBL1	v-myb avian myeloblastosis viral oncogene homolog-like 1
TC1700011787.hg.1	2.64	0.0003	RNF157	ring finger protein 157
TC1900011779.hg.1	2.61	0.0003	FLT3LG	fms-related tyrosine kinase 3 ligand
TC1100012981.hg.1	2.6	0.0006	RCN1	reticulocalbin 1, EF-hand calcium binding domain
TC0400010699.hg.1	2.6	0.0469	SCFD2	sec1 family domain containing 2
TC0500012017.hg.1	2.57	0.0107	IRF1	interferon regulatory factor 1
TC0800008032.hg.1	2.54	0.0132	ZC2HC1A	zinc finger, C2HC-type containing 1A
TC0400008861.hg.1	2.52	0.0032	IL15	interleukin 15
TC0100007481.hg.1	2.52	0.0041	PIGV	phosphatidylinositol glycan anchor biosynthesis class V
TC0200009091.hg.1	2.43	0.0347	RALB	v-ral simian leukemia viral oncogene homolog B

TC1200012815.hg.1	2.38	0.0279	METTL1	methyltransferase like 1
TC1600007557.hg.1	2.36	0.0007	ZNF267	zinc finger protein 267
TC0900008314.hg.1	2.36	0.0422	FSD1L	fibronectin type III and SPRY domain containing 1-like
TC0200016274.hg.1	2.3	0.0495	HDAC4	Transcript Identified by AceView, Entrez Gene ID(s) 9759
TC1600008139.hg.1	2.29	0.0000 274	CES4A	carboxylesterase 4A
TC0600010688.hg.1	2.29	0.0392	LYRM4	LYR motif containing 4
TC1600006658.hg.1	2.28	0.0175	IL32	interleukin 32
TC0600011945.hg.1	2.27	0.0021	RCAN2	regulator of calcineurin 2
TC0600011560.hg.1	2.26	0.0348	UQCC2	ubiquinol-cytochrome c reductase complex assembly factor 2
TC0100014014.hg.1	2.23	0.0344	PRDX1	peroxiredoxin 1
TC0200006583.hg.1	2.22	0.0441	RNF144A	ring finger protein 144A
TC0600014140.hg.1	2.2	0.0001	PRIM2	Salzman2013 ANNOTATED, CDS, coding, OVCODE, OVEXON, UTR3 best transcript NM_000947
TC0300006483.hg.1	2.2	0.0036	BHLHE40	basic helix-loop-helix family, member e40
TC0X00006823.hg.1	2.17	0.0111	POLA1	polymerase (DNA directed), alpha 1, catalytic subunit
TC0700009232.hg.1	2.17	0.002	BPGM	2,3-bisphosphoglycerate mutase
TC0200008666.hg.1	2.17	0.0376	IL1R1	interleukin 1 receptor, type I
TC0200015242.hg.1	2.16	0.0011	STAT1	signal transducer and activator of transcription 1
TC2200007242.hg.1	2.14	0.0179	APOL1	apolipoprotein L1
TC0X00009986.hg.1	2.13	0.016	RP5-1091N2.9; IL2RG	Transcript Identified by AceView, Entrez Gene ID(s) 3561; putative novel transcript
TC0900011177.hg.1	2.13	0.0073	TXN	thioredoxin
TC0100014855.hg.1	2.11	0.028	GBP4	guanylate binding protein 4
TC1500006963.hg.1	2.1	0.0037	CASC5	cancer susceptibility candidate 5

TC0700008857.hg.1	2.1	0.0032	MDFIC	MyoD family inhibitor domain containing
TC0600007595.hg.1	2.08	0.0223	LTA	lymphotoxin alpha
TC0100014365.hg.1	2.08	0.024	CYP2J2	cytochrome P450, family 2, subfamily J, polypeptide 2
TC1600010732.hg.1	2.06	0.0345	NQO1	NAD(P)H dehydrogenase, quinone 1
TC0600013780.hg.1	2.06	0.0143	LPAL2	lipoprotein, Lp(a)-like 2, pseudogene
TC1200012561.hg.1	2.05	0.0499	ZNF891	zinc finger protein 891
TC0900011302.hg.1	2.05	0.0002	TNFSF8	tumor necrosis factor (ligand) superfamily, member 8
TC0600011674.hg.1	2.04	0.0387	PPIL1	peptidylprolyl isomerase (cyclophilin)-like 1
TC1400009992.hg.1	2.03	0.0135	FBLN5	fibulin 5
TSUnmapped00000558.hg.1	2.02	0.0012	HYOU1	hypoxia up-regulated 1
TSUnmapped00000263.hg.1	2.02	0.0025	BCL2L14	BCL2-like 14 (apoptosis facilitator)
TC1200010556.hg.1	2.02	0.0225	HDAC7	histone deacetylase 7
TC0700007477.hg.1	2.02	0.0094	UPP1	uridine phosphorylase 1
TC0300006793.hg.1	2.02	0.0046	KAT2B	Transcript Identified by AceView, Entrez Gene ID(s) 8850
TC1000009691.hg.1	14.26	0.000000000798	IL2RA	interleukin 2 receptor, alpha
TC0200016584.hg.1	1.97	0.0256	HSPE1	heat shock 10kDa protein 1
TC0100018208.hg.1	1.97	0.0145	ZBTB8B	zinc finger and BTB domain containing 8B
TC0800006980.hg.1	1.96	0.0189	SLC39A14	solute carrier family 39 (zinc transporter), member 14
TC1900011741.hg.1	1.95	0.0095	CEACAM5	carcinoembryonic antigen-related cell adhesion molecule 5
TC1000007272.hg.1	1.95	0.0426	CREM	cAMP responsive element modulator
TC0700009233.hg.1	1.95	0.0225	BPGM	Transcript Identified by AceView, Entrez Gene ID(s) 669
TC0200015329.hg.1	1.95	0.0272	GTF3C3	general transcription factor IIIC subunit 3

TSUnmapped00000484.hg.1	1.94	0.0103	HYOU1	hypoxia up-regulated 1
TC0600014074.hg.1	1.93	0.0129	HIVEP1	human immunodeficiency virus type I enhancer binding protein 1
TC0200011855.hg.1	1.93	0.0183	SMC6	Transcript Identified by AceView, Entrez Gene ID(s) 79677
TC0100017834.hg.1	1.93	0.0079	GNG4	guanine nucleotide binding protein (G protein), gamma 4
TSUnmapped00000244.hg.1	1.92	0.0473	HYOU1	hypoxia up-regulated 1
TC0700012443.hg.1	1.92	0.002	FEZF1	FEZ family zinc finger 1
TC1100012510.hg.1	1.91	0.0438	HYOU1	hypoxia up-regulated 1
TC0400011043.hg.1	1.91	0.0314	CDKL2	cyclin-dependent kinase-like 2 (CDC2-related kinase)
TC0300008713.hg.1	1.91	0.0024	SEC61A1	Sec61 translocon alpha 1 subunit
TC2200007214.hg.1	1.9	0.0483	APOL6	apolipoprotein L, 6
TC0100009580.hg.1	1.9	0.0376	WDR3	WD repeat domain 3
TC2200008592.hg.1	1.89	0.0318	APOL2	apolipoprotein L, 2
TC1700006611.hg.1	1.89	0.0175	CYB5D2	cytochrome b5 domain containing 2
TC1200008906.hg.1	1.89	0.0455	TRAFD1	TRAF-type zinc finger domain containing 1
TC2200009356.hg.1	1.88	0.013	ARFGAP3	ADP-ribosylation factor GTPase activating protein 3
TSUnmapped00000329.hg.1	1.87	0.0298	SURF4	surfeit 4
TSUnmapped00000076.hg.1	1.87	0.0296	SURF4	surfeit 4
TC1700009219.hg.1	1.87	0.0168	RAC3	ras-related C3 botulinum toxin substrate 3 (rho family, small GTP binding protein Rac3)
TC0300009704.hg.1	1.87	0.0278	FAM131A	family with sequence similarity 131, member A
TC1600011234.hg.1	1.86	0.0367	APRT	adenine phosphoribosyltransferase

TC2200007910.hg.1	1.85	0.0024	MICAL3	Transcript Identified by AceView, Entrez Gene ID(s) 57553
TC1600009675.hg.1	1.85	0.0404	ZP2	zona pellucida glycoprotein 2 (sperm receptor)
TC0700013065.hg.1	1.85	0.0386	ABCF2	ATP binding cassette subfamily F member 2
TC0400011994.hg.1	1.85	0.0146	INPP4B	inositol polyphosphate-4-phosphatase type II B
TC1100006506.hg.1	1.83	0.038	MUC2	mucin 2, oligomeric mucus/gel-forming
TC0500009409.hg.1	1.83	0.0478	GABRP	gamma-aminobutyric acid (GABA) A receptor, pi
TC1200008525.hg.1	1.82	0.0401	SNRPF	small nuclear ribonucleoprotein polypeptide F
TC0200009806.hg.1	1.82	0.0043	TANK	TRAF family member-associated NFKB activator
TSUnmapped00000383.hg.1	1.81	0.0204	HYOU1	hypoxia up-regulated 1
TSUnmapped00000233.hg.1	1.81	0.032	HYOU1	hypoxia up-regulated 1
TC0200015332.hg.1	1.81	0.0219	PGAP1	post-GPI attachment to proteins 1
TC1300007780.hg.1	1.8	0.0045	RAP2A	RAP2A, member of RAS oncogene family
TC0600012562.hg.1	1.8	0.0252	BACH2	Memczak2013 ALT_ACCEPTOR, ALT_DONOR, coding, INTERNAL, intronic best transcript NM_001170794
TC0100016476.hg.1	1.8	0.0115	KIAA0040	KIAA0040
TC1600009395.hg.1	1.79	0.0038	SOCS1	suppressor of cytokine signaling 1
TC1400007440.hg.1	1.79	0.0369	MTHFD1	methylenetetrahydrofolate dehydrogenase (NADP+ dependent) 1, methenyltetrahydrofolate cyclohydrolase, formyltetrahydrofolate synthetase
TC1100009824.hg.1	1.79	0.0463	CARS	cysteinyl-tRNA synthetase
TC0800007431.hg.1	1.79	0.0089	AP3M2	adaptor-related protein complex 3, mu 2 subunit

TC0300014021.hg.1	1.79	0.0328	CPOX	coproporphyrinogen oxidase
TC1900011422.hg.1	1.78	0.0273	LENG9	leukocyte receptor cluster (LRC) member 9
TC1700012433.hg.1	1.78	0.0167	HOXB6	homeobox B6
TC0400012458.hg.1	1.78	0.0163	HPGD	hydroxyprostaglandin dehydrogenase 15-(NAD)
TC1800007905.hg.1	1.77	0.0227	LPIN2	lipin 2
TC1000011266.hg.1	1.77	0.0481	LRIT2	leucine-rich repeat, immunoglobulin-like and transmembrane domains 2
TC0600008515.hg.1	1.77	0.0016	KCNQ5	Jeck2013 ALT_ACCEPTOR, ALT_DONOR, coding, INTERNAL, intronic best transcript NM_001160133
TC2200007780.hg.1	1.76	0.0035	CRELD2	cysteine rich with EGF-like domains 2
TC1700008282.hg.1	1.76	0.0225	NME1; NME1-NME2; NME2	NME/NM23 nucleoside diphosphate kinase 1; NME1-NME2 readthrough; NME/NM23 nucleoside diphosphate kinase 2
TC0700007467.hg.1	1.76	0.0302	C7orf65	chromosome 7 open reading frame 65
TC0100012778.hg.1	1.75	0.0136	ENO1	enolase 1, (alpha)
TC1900009394.hg.1	1.74	0.0128	C19orf70	chromosome 19 open reading frame 70
TC0800010043.hg.1	1.74	0.037	PPP2CB	protein phosphatase 2, catalytic subunit, beta isozyme
TC0600008027.hg.1	1.74	0.0168	BYSL	bystin-like
TC2000008950.hg.1	1.73	0.0036	ACSS2	Memczak2013 ANTISENSE, CDS, coding, INTERNAL best transcript NM_001076552
TC0900008207.hg.1	1.73	0.0071	SEC61B	Sec61 translocon beta subunit
TC0700010941.hg.1	1.73	0.0414	MYO1G	myosin IG
TC1100013000.hg.1	1.72	0.0099	CLP1; AP000662.4	Transcript Identified by AceView, Entrez Gene ID(s) 10978; novel transcript, sense overlapping CLP1
TC0500012605.hg.1	1.72	0.0056	SOX30	SRY box 30
TC0X00008940.hg.1	1.71	0.0468	MXRA5	matrix-remodelling associated 5
TC1100013021.hg.1	1.7	0.0411	FEN1	flap structure-specific endonuclease 1

TC0500006730.hg.1	1.7	0.0261	CCT5	chaperonin containing TCP1, subunit 5 (epsilon)
TC1700006804.hg.1	1.69	0.0466	PFAS	phosphoribosylformylglycinamidine synthase
TC1200009459.hg.1	1.69	0.0103	PUS1	pseudouridylate synthase 1
TC0X00011159.hg.1	1.68	0.0183	HAUS7; TREX2	HAUS augmin like complex subunit 7; three prime repair exonuclease 2
TC0400007592.hg.1	1.68	0.0191	PAICS	phosphoribosylaminoimidazole carboxylase, phosphoribosylaminoimidazole succinocarboxamide synthetase
TC2000007341.hg.1	1.67	0.034	FAM83D	family with sequence similarity 83, member D
TC1700011181.hg.1	1.67	0.0007	MMD	monocyte to macrophage differentiation-associated
TC0600012560.hg.1	1.67	0.0406	BACH2	Memczak2013 ALT_ACCEPTOR, ALT_DONOR, coding, INTERNAL, intronic best transcript NM_001170794
TC0100011512.hg.1	1.67	0.0392	DTL	denticleless E3 ubiquitin protein ligase homolog (Drosophila)
TC1900011781.hg.1	1.66	0.0396	PRMT1	protein arginine methyltransferase 1
TC0700008149.hg.1	1.66	0.0416	PHTF2	putative homeodomain transcription factor 2
TC0500010148.hg.1	1.66	0.045	DNAH5	dynein, axonemal, heavy chain 5
TC0100008885.hg.1	1.66	0.0455	SPATA1	spermatogenesis associated 1
TC1200011711.hg.1	1.65	0.0006	GNPTAB	N-acetylglucosamine-1-phosphate transferase, alpha and beta subunits
TC0300007437.hg.1	1.65	0.007	MANF	mesencephalic astrocyte-derived neurotrophic factor
TC2000009392.hg.1	1.64	0.0466	B4GALT5	UDP-Gal:betaGlcNAc beta 1,4-galactosyltransferase, polypeptide 5
TC0100010648.hg.1	1.63	0.0099	PRDX6	peroxiredoxin 6
TC2200007825.hg.1	1.62	0.0162	SHANK3	SH3 and multiple ankyrin repeat domains 3
TC1900009804.hg.1	1.62	0.0338	C19orf67	chromosome 19 open reading frame 67

TC1100007184.hg.1	1.62	0.0183	DNAJC24	DnaJ (Hsp40) homolog, subfamily C, member 24
TC0200012031.hg.1	1.61	0.0139	HADHA	Transcript Identified by AceView, Entrez Gene ID(s) 3030
TC0200009813.hg.1	1.61	0.0015	PSMD14	proteasome 26S subunit, non-ATPase 14
TC1200011722.hg.1	1.6	0.0033	PMCH	pro-melanin-concentrating hormone
TC0200011450.hg.1	1.6	0.0406	FAM150B	family with sequence similarity 150, member B
TSUnmapped00000327.hg.1	1.59	0.0471	HYOU1	hypoxia up-regulated 1
TC1900007004.hg.1	1.59	0.0057	C19orf52	chromosome 19 open reading frame 52
TC1500007196.hg.1	1.59	0.0193	SLC27A2	solute carrier family 27 (fatty acid transporter), member 2
TC1300009954.hg.1	1.59	0.0257	DNAJC15	DnaJ (Hsp40) homolog, subfamily C, member 15
TC0500009147.hg.1	1.59	0.0246	GRIA1	Transcript Identified by AceView, Entrez Gene ID(s) 2890
TC0300006714.hg.1	1.59	0.0183	EAF1	ELL associated factor 1
TC0100012343.hg.1	1.59	0.0099	OR2L2	olfactory receptor, family 2, subfamily L, member 2
TSUnmapped00000384.hg.1	1.58	0.0417	ZNF546	zinc finger protein 546
TC1400010721.hg.1	1.58	0.0267	PSME2; MIR7703	proteasome activator subunit 2; microRNA 7703
TC1400009770.hg.1	1.58	0.0156	ZDHHC22	zinc finger, DHHC-type containing 22
TC1100009365.hg.1	1.58	0.007	OR8D4	olfactory receptor, family 8, subfamily D, member 4
TC0600009169.hg.1	1.58	0.0362	FAM229B	family with sequence similarity 229, member B
TC2000009550.hg.1	1.57	0.0168	AURKA	aurora kinase A
TC2000009220.hg.1	1.57	0.012	TP53TG5	TP53 target 5

TC1800008399.hg.1	1.57	0.0106	B4GALT6	UDP-Gal:betaGlcNAc beta 1,4-galactosyltransferase, polypeptide 6
TC1600006653.hg.1	1.57	0.0264	THOC6	THO complex 6
TC1400006823.hg.1	1.57	0.0318	SCFD1	sec1 family domain containing 1
TC1000012423.hg.1	1.57	0.0246	PFKP	phosphofructokinase, platelet
TC1000008431.hg.1	1.57	0.0021	PCGF5	polycomb group ring finger 5
TC0200010202.hg.1	1.57	0.0433	FSIP2	fibrous sheath interacting protein 2
TC2000008860.hg.1	1.56	0.0427	COMMD7	COMM domain containing 7
TC1200009672.hg.1	1.56	0.0069	C12orf4	chromosome 12 open reading frame 4
TC1100009450.hg.1	1.56	0.0375	DCPS	decapping enzyme, scavenger
TC1100008797.hg.1	1.56	0.0337	PANX1	pannexin 1
TC1400010722.hg.1	1.55	0.0225	IPO4	importin 4
TC1000010439.hg.1	1.55	0.0281	HNRNPF	Memczak2013 ALT_ACCEPTOR, ALT_DONOR, coding, INTERNAL, intronic best transcript NM_001098207
TC0600011903.hg.1	1.55	0.044	MRPL14	mitochondrial ribosomal protein L14
TC0400008084.hg.1	1.55	0.0469	DMP1	dentin matrix acidic phosphoprotein 1
TC1000011003.hg.1	1.54	0.0265	PLA2G12B	phospholipase A2, group XIIB
TC0700008397.hg.1	1.54	0.0205	DYNC1I1	dynein, cytoplasmic 1, intermediate chain 1
TC0100008194.hg.1	1.54	0.0316	FOXD2	forkhead box D2
TC1600010604.hg.1	1.53	0.0089	FAM96B	family with sequence similarity 96, member B
TC1500010941.hg.1	1.53	0.0396	LYSMD4	LysM, putative peptidoglycan-binding, domain containing 4
TC1900011055.hg.1	1.52	0.022	BSPH1	binder of sperm protein homolog 1
TC0700009020.hg.1	1.52	0.0221	SND1; SND1-IT1	staphylococcal nuclease and tudor domain containing 1; SND1 intronic transcript 1
TC0400011414.hg.1	1.52	0.0076	TRMT10A	tRNA methyltransferase 10A
TC0300008672.hg.1	1.52	0.0156	CFAP100	cilia and flagella associated protein 100
TC0100018323.hg.1	1.52	0.0464	IER5	immediate early response 5
TSUnmapped00000323.hg.1	1.51	0.0265	RPS6KA1	ribosomal protein S6 kinase, 90kDa, polypeptide 1

TC1400009568.hg.1	1.51	0.0241	SLC8A3	solute carrier family 8 (sodium/calcium exchanger), member 3
TC0500008845.hg.1	1.51	0.0436	PURA	purine-rich element binding protein A
TSUnmapped00000489.hg.1	1.5	0.0242	SURF4	surfeit 4
TC2000006899.hg.1	1.5	0.0134	XRN2	5-3 exoribonuclease 2
TC1200009036.hg.1	1.5	0.0332	FBXW8	F-box and WD repeat domain containing 8
TC0X00009386.hg.1	1.5	0.0423	SRPX	sushi-repeat containing protein, X-linked
TC0700010910.hg.1	1.5	0.0206	POLD2	polymerase (DNA directed), delta 2, accessory subunit
TC2000008815.hg.1	1.48	0.0102	BCL2L1	BCL2-like 1
TC2000007973.hg.1	1.48	0.0371	CDH4	cadherin 4, type 1, R-cadherin (retinal)
TC1000012510.hg.1	1.48	0.0084	ZNF511	zinc finger protein 511
TC0300007739.hg.1	1.48	0.0287	PSMC1	Homo sapiens proteasome (prosome, macropain) 26S subunit, ATPase, 1, mRNA (cDNA clone MGC:8541 IMAGE:2822718), complete cds.; Homo sapiens proteasome (prosome, macropain) 26S subunit, ATPase, 1, mRNA (cDNA clone MGC:24583 IMAGE:4133348), complete cds.; Homo sapiens proteasome (prosome, macropain) 26S subunit, ATPase, 1, mRNA (cDNA clone MGC:86994 IMAGE:5264945), complete cds.; Homo sapiens proteasome (prosome, macropain) 26S subunit, ATPase, 1, mRNA (cDNA clone MGC:88853 IMAGE:5456334), complete cds.
TC0100017668.hg.1	1.48	0.019	TAF5L	TAF5-like RNA polymerase II, p300/CBP-associated factor (PCAF)-associated factor, 65kDa
TC1800007080.hg.1	1.47	0.0397	GALNT1	polypeptide N-acetylgalactosaminyltransferase 1

TC1600009966.hg.1	1.47	0.0081	DCTPP1	dCTP pyrophosphatase 1
TC1200008642.hg.1	1.47	0.0122	PARPBP	PARP1 binding protein
TC0900011793.hg.1	1.47	0.0305	MED27	mediator complex subunit 27
TC0100015330.hg.1	1.47	0.0371	PHTF1	putative homeodomain transcription factor 1
TC0200008230.hg.1	1.46	0.0134	TMSB10	thymosin beta 10
TC1100007185.hg.1	1.45	0.0346	ELP4	elongator acetyltransferase complex subunit 4
TC1000008088.hg.1	1.45	0.0487	SAMD8	sterile alpha motif domain containing 8
TC0400011503.hg.1	1.45	0.0212	PPA2	pyrophosphatase (inorganic) 2
TC0600009336.hg.1	1.44	0.0446	CLVS2	clavesin 2
TC0100007672.hg.1	1.44	0.0143	EIF3I	eukaryotic translation initiation factor 3, subunit I
TC1100011157.hg.1	1.43	0.0052	PPP1R14B	protein phosphatase 1, regulatory (inhibitor) subunit 14B
TC1900011869.hg.1	1.42	0.0222	RGL3	ral guanine nucleotide dissociation stimulator-like 3
TC1900011221.hg.1	1.42	0.0031	C19orf48; SNORD88B; SNORD88C	chromosome 19 open reading frame 48; small nucleolar RNA, C/D box 88B; small nucleolar RNA, C/D box 88C
TC1900010301.hg.1	1.42	0.0404	C19orf12	chromosome 19 open reading frame 12
TC1800007218.hg.1	1.42	0.0175	C18orf25	chromosome 18 open reading frame 25
TC1100013047.hg.1	1.42	0.0227	SF3B2	splicing factor 3b subunit 2
TC1100007610.hg.1	1.42	0.0302	OR5D14	olfactory receptor, family 5, subfamily D, member 14
TC0700011905.hg.1	1.42	0.0133	BAIAP2L1	BAI1-associated protein 2-like 1
TC1700008719.hg.1	1.41	0.0415	KPNA2	karyopherin alpha 2 (RAG cohort 1, importin alpha 1)
TC1600007112.hg.1	1.41	0.0397	CCP110	centriolar coiled coil protein 110kDa
TC1200012258.hg.1	1.41	0.0331	PITPNM2	phosphatidylinositol transfer protein, membrane-associated 2
TC1200009071.hg.1	1.41	0.0067	PEBP1	phosphatidylethanolamine binding protein 1

TC0600007714.hg.1	1.41	0.0328	ITPR3	Zhang2013 ALT_ACCEPTOR, ALT_DONOR, coding, INTERNAL, intronic best transcript NM_002224
TC0500007841.hg.1	1.41	0.0412	AGGF1	angiogenic factor with G-patch and FHA domains 1
TC2000006823.hg.1	1.4	0.0121	ZNF133	zinc finger protein 133
TC0500012059.hg.1	1.4	0.0439	VDAC1	voltage-dependent anion channel 1
TC0100011855.hg.1	1.4	0.0297	GUK1	guanylate kinase 1
TC0600008972.hg.1	0.65	0.0199	PRDM1	PR domain containing 1, with ZNF domain
TC1800007570.hg.1	-2	0.0301	CDH7	cadherin 7, type 2
TC1200012577.hg.1	-2	0.009	GALNT8	polypeptide N-acetylgalactosaminyltransferase 8
TC0500007531.hg.1	-2	0.0446	PDE4D	Memczak2013 ANTISENSE, coding, INTERNAL, intronic best transcript NM_001165899
TC0300012812.hg.1	-0.83	0.0412	GPR171	G protein-coupled receptor 171
TC1900007314.hg.1	-1.4	0.0164	OCEL1	occludin/ELL domain containing 1
TC0300013813.hg.1	-1.4	0.0214	ACKR2	atypical chemokine receptor 2
TC0700009479.hg.1	-1.41	0.04	OR6V1	olfactory receptor, family 6, subfamily V, member 1
TC0500013385.hg.1	-1.41	0.0118	SH3TC2	SH3 domain and tetratricopeptide repeats 2
TC0900008665.hg.1	-1.42	0.0213	OR1J2	olfactory receptor, family 1, subfamily J, member 2
TC1700010856.hg.1	-1.43	0.0394	C1QL1	complement component 1, q subcomponent-like 1
TC1700010276.hg.1	-1.43	0.0473	TMIGD1	transmembrane and immunoglobulin domain containing 1
TC0200015645.hg.1	-1.43	0.0226	ABCA12	ATP binding cassette subfamily A member 12
TC2000009210.hg.1	-1.44	0.0437	WFDC5	WAP four-disulfide core domain 5
TC1600007723.hg.1	-1.44	0.013	GPT2	glutamic pyruvate transaminase (alanine aminotransferase) 2
TC1200007526.hg.1	-1.44	0.0349	LINC00935	long intergenic non-protein coding RNA 935

TC1100007571.hg.1	-1.44	0.0217	OR4A8	olfactory receptor, family 4, subfamily A, member 8 (gene/pseudogene) [Source:HGNC Symbol;Acc:HGNC:15165]
TC0300007438.hg.1	-1.44	0.0466	RBM15B	RNA binding motif protein 15B
TC0200012141.hg.1	-1.44	0.0285	ALK	Transcript Identified by AceView, Entrez Gene ID(s) 238
TC1900011956.hg.1	-1.45	0.0164	CYP2A7	cytochrome P450, family 2, subfamily A, polypeptide 7
TC1600009766.hg.1	-1.45	0.0476	LINC01567	long intergenic non-protein coding RNA 1567
TC1200011495.hg.1	-1.45	0.0371	LUM	lumican
TC0500007876.hg.1	-1.45	0.0291	BHMT	betaine--homocysteine S-methyltransferase
TC0100015862.hg.1	-1.45	0.0154	S100A7	S100 calcium binding protein A7
TC2200009140.hg.1	-1.46	0.0286	TTLL8	tubulin tyrosine ligase-like family member 8
TC1800008437.hg.1	-1.46	0.027	NOL4	nucleolar protein 4
TC1800007490.hg.1	-1.46	0.014	CDH20	cadherin 20, type 2
TC1500006507.hg.1	-1.46	0.0158	GOLGA6L22; GOLGA6L1	golgin A6 family-like 22; golgin A6 family-like 1
TC1100009912.hg.1	-1.46	0.0397	OR52A1	olfactory receptor, family 52, subfamily A, member 1
TC1100007865.hg.1	-1.46	0.0471	METTL12; SNORA57	methyltransferase like 12; small nucleolar RNA, H/ACA box 57
TC0800011697.hg.1	-1.46	0.0215	FER1L6-AS1	FER1L6 antisense RNA 1
TC0600011738.hg.1	-1.46	0.0223	KCNK17	potassium channel, two pore domain subfamily K, member 17
TC0100015764.hg.1	-1.46	0.045	FAM63A	family with sequence similarity 63, member A
TC2200007101.hg.1	-1.47	0.041	LINC01521	long intergenic non-protein coding RNA 1521
TC1100006525.hg.1	-1.47	0.0465	KRTAP5-5	keratin associated protein 5-5
TC0X00010484.hg.1	-1.47	0.0067	COL4A6	collagen, type IV, alpha 6
TC0300008198.hg.1	-1.47	0.0365	ZPLD1	zona pellucida-like domain containing 1
TC0200007388.hg.1	-1.47	0.0264	LINC01126	long intergenic non-protein coding RNA 1126
TC0100018301.hg.1	-1.47	0.0427	DCST1	DC-STAMP domain containing 1

TC2200006881.hg.1	-1.48	0.0422	CABIN1	calcineurin binding protein 1
TC0500013266.hg.1	-1.48	0.0299	HMP19	HMP19 protein; Neuron-specific protein family member 2 [Source:UniProtKB/Swiss-Prot;Acc:Q9Y328]; Transcript Identified by AceView, Entrez Gene ID(s) 51617
TC0500007258.hg.1	-1.48	0.0435	GHR	growth hormone receptor
TC0400011727.hg.1	-1.48	0.026	PRDM5	PR domain containing 5
TC0100013382.hg.1	-1.48	0.0293	AIM1L	absent in melanoma 1-like
TC1900011481.hg.1	-1.49	0.0411	SBK2	SH3 domain binding kinase family, member 2
TC1800006447.hg.1	-1.49	0.0487	CLUL1	clusterin-like 1 (retinal)
TC1600011519.hg.1	-1.49	0.046	SEPT1	septin 1
TC1200009914.hg.1	-1.49	0.0111	TAS2R7	taste receptor, type 2, member 7
TC1100007601.hg.1	-1.49	0.0235	OR4S2	olfactory receptor, family 4, subfamily S, member 2
TC0700012221.hg.1	-1.49	0.0407	SLC26A3	solute carrier family 26 (anion exchanger), member 3
TC0700008961.hg.1	-1.49	0.0387	ASB15	ankyrin repeat and SOCS box containing 15
TC1900009382.hg.1	-1.5	0.014	PTPRS	protein tyrosine phosphatase, receptor type, S
TC1200011993.hg.1	-1.5	0.0017	SLC8B1	solute carrier family 8 (sodium/lithium/calcium exchanger), member B1
TC1100011202.hg.1	-1.5	0.0338	TMEM262	transmembrane protein 262
TC0300012140.hg.1	-1.5	0.0233	GPR156	G protein-coupled receptor 156
TC0100010016.hg.1	-1.5	0.0486	SPRR4	small proline-rich protein 4
TC1100011914.hg.1	-1.51	0.0287	TRIM64B	tripartite motif containing 64B
TC1100010041.hg.1	-1.51	0.0367	ST5	Transcript Identified by AceView, Entrez Gene ID(s) 6764
TC0Y00006812.hg.1	-1.51	0.0322	DAZ2; DAZ4	deleted in azoospermia 2; deleted in azoospermia 4
TC0X00010893.hg.1	-1.51	0.0452	CT55	cancer/testis antigen 55

TC0900009922.hg.1	-1.52	0.003	FAM205C	family with sequence similarity 205, member C
TC0100013246.hg.1	-1.52	0.0323	WNT4	wingless-type MMTV integration site family, member 4
TC0100007194.hg.1	-1.52	0.008	UBXN10	UBX domain protein 10
TSUnmapped00000356.hg.1	-1.53	0.0403	DYRK1B	dual specificity tyrosine-(Y)-phosphorylation regulated kinase 1B
TC1100009237.hg.1	-1.53	0.0437	VPS11	vacuolar protein sorting 11 homolog (S. cerevisiae)
TC0Y00007240.hg.1	-1.53	0.0021	CSPG4P1Y; DNM1P24	chondroitin sulfate proteoglycan 4 pseudogene 1, Y-linked; dynamin 1 pseudogene 24
TC0900009855.hg.1	-1.53	0.0344	BAG1	BCL2-associated athanogene
TC0500009185.hg.1	-1.53	0.0448	KIF4B	kinesin family member 4B
TC0100012469.hg.1	-1.53	0.0212	MXRA8	matrix-remodelling associated 8
TSUnmapped00000247.hg.1	-1.54	0.0042	REXO4	REX4 homolog, 3'-5' exonuclease [Source:HGNC Symbol;Acc:HGNC:12820]
TC1500010689.hg.1	-1.54	0.0272	HERC2P2	hect domain and RLD 2 pseudogene 2
TC1200012578.hg.1	-1.54	0.0012	KCNA6; GALNT8	potassium channel, voltage gated shaker related subfamily A, member 6; polypeptide N-acetylgalactosaminyltransferase 8
TC0200008801.hg.1	-1.54	0.0039	SULT1C4	sulfotransferase family 1C member 4
TC0100017115.hg.1	-1.54	0.0171	FCAMR	Fc receptor, IgA, IgM, high affinity
TC1600009977.hg.1	-1.55	0.0315	ZNF688	zinc finger protein 688
TC1200011996.hg.1	-1.55	0.0126	LHX5	LIM homeobox 5
TC0800010808.hg.1	-1.55	0.0335	RDH10-AS1	RDH10 antisense RNA 1
TC0700013051.hg.1	-1.55	0.0146	ATG9B	autophagy related 9B
TC0700009481.hg.1	-1.55	0.044	PIP	prolactin-induced protein
TC0300013597.hg.1	-1.55	0.007	CPN2	carboxypeptidase N, polypeptide 2
TC0100015265.hg.1	-1.55	0.0092	DENND2D	DENN/MADD domain containing 2D

TC1900011679.hg.1	-1.56	0.0133	CYP4F3	cytochrome P450, family 4, subfamily F, polypeptide 3
TC1600008329.hg.1	-1.56	0.04	HP	haptoglobin
TC0900008379.hg.1	-1.56	0.0209	ACTL7A	actin-like 7A
TC0400009080.hg.1	-1.56	0.0276	NPY2R	neuropeptide Y receptor Y2
TC0200015571.hg.1	-1.56	0.0149	CRYGC	crystallin gamma C
TC2200008528.hg.1	-1.57	0.0281	LARGE	like-glycosyltransferase
TC0600008745.hg.1	-1.57	0.0455	SPACA1	sperm acrosome associated 1
TC0500007933.hg.1	-1.57	0.0222	CKMT2	creatine kinase, mitochondrial 2 (sarcomeric)
TC1900011850.hg.1	-1.58	0.0029	FUT5	fucosyltransferase 5 (alpha (1,3) fucosyltransferase)
TC0800007284.hg.1	-1.58	0.019	KCNU1	potassium channel, subfamily U, member 1
TC0700008506.hg.1	-1.58	0.0397	CYP3A43	cytochrome P450, family 3, subfamily A, polypeptide 43
TC0500013016.hg.1	-1.58	0.0187	COL23A1	collagen, type XXIII, alpha 1
TC0200010859.hg.1	-1.58	0.044	SLC4A3	solute carrier family 4 (anion exchanger), member 3
TC1100009924.hg.1	-1.59	0.0366	OR51B4	olfactory receptor, family 51, subfamily B, member 4
TC0100018124.hg.1	-1.59	0.042	OR2M7	olfactory receptor, family 2, subfamily M, member 7
TC1500008670.hg.1	-1.6	0.0169	POTEB	POTE ankyrin domain family, member B
TC1000012481.hg.1	-1.6	0.033	CYP2C19	cytochrome P450, family 2, subfamily C, polypeptide 19
TC0100011417.hg.1	-1.6	0.0173	CR1L	complement component (3b/4b) receptor 1-like
TC0200010157.hg.1	-1.61	0.0086	ITGA4	integrin alpha 4
TSUnmapped00000539.hg.1	-1.62	0.0473	LRIG1	
TC2000008684.hg.1	-1.62	0.0226	CST2	cystatin SA

TC1700010853.hg.1	-1.62	0.0235	KIF18B	Memczak2013 ALT_ACCEPTOR, coding, INTERNAL, intronic best transcript NM_001080443
TC1300009906.hg.1	-1.62	0.0152	ADPRHL1	ADP-ribosylhydrolase like 1
TC1100009615.hg.1	-1.62	0.0468	VPS26B	VPS26 retromer complex component B
TC0500012463.hg.1	-1.62	0.0091	CAMK2A	calcium/calmodulin-dependent protein kinase II alpha
TC0200010800.hg.1	-1.62	0.0149	VIL1	villin 1
TC0100016902.hg.1	-1.62	0.0271	ASCL5	achaete-scute family bHLH transcription factor 5
TC0100011413.hg.1	-1.62	0.0448	CR2	complement component (3d/Epstein Barr virus) receptor 2
TC1700010682.hg.1	-1.63	0.0362	KRT17	keratin 17, type I
TC1700009186.hg.1	-1.63	0.0113	CCDC137	coiled-coil domain containing 137
TC1400008546.hg.1	-1.63	0.0324	LINC01296; DUXAP10	long intergenic non-protein coding RNA 1296; double homeobox A pseudogene 10
TC1200010839.hg.1	-1.63	0.0003	ITGA5	integrin alpha 5
TC1200008731.hg.1	-1.63	0.0295	RFX4	regulatory factor X, 4 (influences HLA class II expression)
TC1000007950.hg.1	-1.63	0.0386	UNC5B	unc-5 netrin receptor B
TC0300013606.hg.1	-1.63	0.0202	TMEM44	transmembrane protein 44
TC1600007308.hg.1	-1.64	0.0141	KDM8	lysine (K)-specific demethylase 8
TC1500007067.hg.1	-1.64	0.0324	CKMT1A	creatine kinase, mitochondrial 1A
TC0900012193.hg.1	-1.64	0.0483	MAMDC4	MAM domain containing 4
TC2100006881.hg.1	-1.65	0.0417	KRTAP20-4	keratin associated protein 20-4
TC0X00011012.hg.1	-1.65	0.0325	SLITRK4	SLIT and NTRK-like family, member 4
TC0300008332.hg.1	-1.65	0.0352	TAGLN3	transgelin 3
TC1900007836.hg.1	-1.66	0.0361	FXYD3; MIR6887	FXYD domain containing ion transport regulator 3; microRNA 6887
TC1100012671.hg.1	-1.66	0.0429	OR8B8	olfactory receptor, family 8, subfamily B, member 8

TC1000012270.hg.1	-1.66	0.0106	EBF3	early B-cell factor 3
TC0900012270.hg.1	-1.66	0.0195	FRRS1L	ferric-chelate reductase 1-like
TC0900009054.hg.1	-1.66	0.0349	GTF3C5	Zhang2013 ALT_ACCEPTOR, ALT_DONOR, coding, INTERNAL, intronic best transcript NM_001122823
TC0800010631.hg.1	-1.66	0.0221	TTPA	tocopherol (alpha) transfer protein
TC0300007733.hg.1	-1.66	0.0158	FAM19A1	family with sequence similarity 19 (chemokine (C-C motif)-like), member A1
TC0100016045.hg.1	-1.66	0.0161	FCRL4	Fc receptor-like 4
TC1900009079.hg.1	-1.67	0.0231	MIER2	Zhang2013 ALT_ACCEPTOR, ALT_DONOR, coding, INTERNAL, intronic, OVERLAPTX best transcript NM_017550
TC1600009949.hg.1	-1.67	0.0138	FAM57B	family with sequence similarity 57, member B
TC1100009016.hg.1	-1.67	0.0375	COLCA2	colorectal cancer associated 2
TC0300011030.hg.1	-1.67	0.007	TMEM89	transmembrane protein 89
TC0300009139.hg.1	-1.67	0.0273	TM4SF4	transmembrane 4 L six family member 4
TC1100007577.hg.1	-1.68	0.0272	TRIM48	tripartite motif containing 48
TC1000011028.hg.1	-1.68	0.0276	MYOZ1	myozenin 1
TC0X00008737.hg.1	-1.68	0.0249	CNGA2	cyclic nucleotide gated channel alpha 2
TC0600011605.hg.1	-1.68	0.0367	TCP11	t-complex 11, testis-specific
TC0500008872.hg.1	-1.68	0.0292	PCDHB1	protocadherin beta 1
TC0100015847.hg.1	-1.68	0.0401	SPRR2D	small proline-rich protein 2D
TC0100006931.hg.1	-1.68	0.0462	PDPN	podoplanin
TC1100012105.hg.1	-1.69	0.047	ANGPTL5	angiopoietin like 5
TSUnmapped00000446.hg.1	-1.7	0.0394	INPP5D	inositol polyphosphate-5-phosphatase D
TC1500010051.hg.1	-1.7	0.0382	RPP25	ribonuclease P/MRP 25kDa subunit
TC1300008072.hg.1	-1.7	0.0111	SPACA7	sperm acrosome associated 7
TC1100011090.hg.1	-1.7	0.0473	EML3	echinoderm microtubule associated protein like 3

TC1100010850.hg.1	-1.7	0.0227	OR5T2	olfactory receptor, family 5, subfamily T, member 2
TC0300013350.hg.1	-1.7	0.0399	YEATS2	Memczak2013 ANTISENSE, coding, INTERNAL, intronic best transcript NM_018023
TC0100014790.hg.1	-1.7	0.024	COL24A1	collagen, type XXIV, alpha 1
TC0100011091.hg.1	-1.7	0.0451	C1orf53	chromosome 1 open reading frame 53
TC1400009780.hg.1	-1.71	0.0205	NOXRED1	NADP-dependent oxidoreductase domain containing 1
TC0300012319.hg.1	-1.71	0.0153	goyborbu; MCM2	Transcript Identified by AceView; Memczak2013 ANTISENSE, CDS, coding, INTERNAL, UTR3 best transcript NM_004526
TC0300013914.hg.1	-1.72	0.0388	TPRG1	tumor protein p63 regulated 1
TC2000008807.hg.1	-1.73	0.0008	DEFB119	defensin, beta 119
TC2000007545.hg.1	-1.73	0.0063	EYA2	EYA transcriptional coactivator and phosphatase 2
TC1900006865.hg.1	-1.73	0.0156	TGFBR3L	transforming growth factor beta receptor III like
TC1100008054.hg.1	-1.73	0.0122	PACS1	phosphofurin acidic cluster sorting protein 1
TC1900006770.hg.1	-1.74	0.0221	DUS3L	Memczak2013 ANTISENSE, CDS, coding, INTERNAL best transcript NM_020175
TC1600008330.hg.1	-1.74	0.0238	HPR	haptoglobin-related protein
TC1000009057.hg.1	-1.74	0.0383	NANOS1	nanos homolog 1 (Drosophila)
TC0300008097.hg.1	-1.74	0.0363	OR5H1	olfactory receptor, family 5, subfamily H, member 1
TC0100012786.hg.1	-1.74	0.0405	SLC2A7	solute carrier family 2 (facilitated glucose transporter), member 7
TC1900008604.hg.1	-1.75	0.049	MGC45922; CTD-2568A17.5	uncharacterized LOC284365; uncharacterized LOC284365 [Source:EntrezGene;Acc:284365]; novel transcript

TC1600006686.hg.1	-1.75	0.0394	OR2C1	olfactory receptor, family 2, subfamily C, member 1
TC1100012526.hg.1	-1.75	0.0457	USP2	ubiquitin specific peptidase 2
TC1000011973.hg.1	-1.75	0.0395	PRLHR	prolactin releasing hormone receptor
TC0600011646.hg.1	-1.75	0.0482	SLC26A8	solute carrier family 26 (anion exchanger), member 8
TC1700008186.hg.1	-1.76	0.0071	IGF2BP1	insulin-like growth factor 2 mRNA binding protein 1
TC0800010252.hg.1	-1.76	0.0402	ANK1	ankyrin 1, erythrocytic
TC0800008349.hg.1	-1.76	0.0078	OSR2	odd-skipped related transciption factor 2
TC0700012778.hg.1	-1.76	0.0482	KLRG2	killer cell lectin-like receptor subfamily G, member 2
TC0600007985.hg.1	-1.76	0.046	TSPO2	translocator protein 2
TC0X00007235.hg.1	-1.77	0.042	GAGE4; GAGE7; GAGE12G	G antigen 4; G antigen 7; G antigen 12G
TC0100016339.hg.1	-1.78	0.0239	DPT	dermatopontin
TC1900009226.hg.1	-1.79	0.0373	LINGO3	leucine rich repeat and Ig domain containing 3
TC1200008795.hg.1	-1.79	0.0065	ACACB	acetyl-CoA carboxylase beta
TC1000009893.hg.1	-1.79	0.0023	ITGA8	integrin alpha 8
TC0400012768.hg.1	-1.79	0.0034	FAM200B	family with sequence similarity 200, member B
TC1100010091.hg.1	-1.8	0.0243	MRVI1	murine retrovirus integration site 1 homolog
TC0900011286.hg.1	-1.8	0.022	DFNB31	deafness, autosomal recessive 31
TC0800008632.hg.1	-1.8	0.0328	SLC30A8	solute carrier family 30 (zinc transporter), member 8
TC0200016026.hg.1	-1.8	0.0257	NMUR1	neuromedin U receptor 1
TC0200013108.hg.1	-1.8	0.0283	LBX2	ladybird homeobox 2
TC0100018207.hg.1	-1.8	0.0216	TSSK3	testis-specific serine kinase 3
TC2000007448.hg.1	-1.81	0.0277	GDAP1L1	ganglioside induced differentiation associated protein 1-like 1
TC0400009037.hg.1	-1.81	0.0147	MND1	meiotic nuclear divisions 1

TC0100013167.hg.1	-1.81	0.0333	PLA2G2D	phospholipase A2, group IID
TC0100010167.hg.1	-1.81	0.0027	TSACC	TSSK6 activating co-chaperone
TC0900010355.hg.1	-1.82	0.0364	C9orf135-AS1	C9orf135 antisense RNA 1 (head to head)
TC0600009456.hg.1	-1.82	0.016	ENPP3	ectonucleotide pyrophosphatase/phosphodiesterase 3
TC0600008670.hg.1	-1.82	0.0092	PRSS35	protease, serine 35
TSUnmapped00000722.hg.1	-1.83	0.0498	TRAPPC4	trafficking protein particle complex 4
TSUnmapped00000415.hg.1	-1.83	0.0017	INPP5D	inositol polyphosphate-5-phosphatase D
TC1800006655.hg.1	-1.83	0.0132	TXNDC2	thioredoxin domain containing 2 (spermatozoa)
TC1300009781.hg.1	-1.83	0.0166	COL4A1	collagen, type IV, alpha 1
TC1100013174.hg.1	-1.83	0.0144	BSCL2	Berardinelli-Seip congenital lipodystrophy 2 (seipin)
TC0700011066.hg.1	-1.83	0.0138	COBL	cordon-bleu WH2 repeat protein
TC0200011257.hg.1	-1.83	0.0086	PRLH	prolactin releasing hormone
TC0900009040.hg.1	-1.84	0.0008	C9orf9	chromosome 9 open reading frame 9
TC0200010839.hg.1	-1.84	0.0365	TUBA4B	tubulin, alpha 4b
TC0600013204.hg.1	-1.85	0.0119	VNN3	vanin 3
TC0200014382.hg.1	-1.85	0.0423	TMEM163	transmembrane protein 163
TC0100007838.hg.1	-1.85	0.0234	DNALI1	dynein, axonemal, light intermediate chain 1
TC1900011913.hg.1	-1.87	0.0181	LINC00663	long intergenic non-protein coding RNA 663
TC1100008038.hg.1	-1.87	0.0141	SNX32	sorting nexin 32
TC0400008134.hg.1	-1.87	0.0293	MMRN1	multimerin 1
TC1100013045.hg.1	-1.88	0.0172	SIPA1	signal-induced proliferation-associated 1
TC0700006991.hg.1	-1.88	0.0334	C7orf71	chromosome 7 open reading frame 71
TC0300006994.hg.1	-1.88	0.0463	FBXL2	F-box and leucine-rich repeat protein 2
TC0300013095.hg.1	-1.89	0.0114	SLC7A14	solute carrier family 7, member 14
TC0X00010990.hg.1	-1.9	0.0265	SPANXC; SPANXD	SPANX family, member C; SPANX family, member D

TC0300014040.hg.1	-1.9	0.0134	TXNRD3NB; TXNRD3	thioredoxin reductase 3 neighbor; thioredoxin reductase 3
TC0300011951.hg.1	-1.9	0.0173	MORC1	MORC family CW-type zinc finger 1
TC0300007161.hg.1	-1.9	0.0482	VIPR1	vasoactive intestinal peptide receptor 1
TC1600009953.hg.1	-1.91	0.0413	GDPD3	glycerophosphodiester phosphodiesterase domain containing 3
TC1900008607.hg.1	-1.92	0.0318	KLK3	kallikrein related peptidase 3
TC1900008303.hg.1	-1.92	0.0204	CLASRP	CLK4-associating serine/arginine rich protein
TC1500007143.hg.1	-1.92	0.0127	SEMA6D	sema domain, transmembrane domain (TM), and cytoplasmic domain, (semaphorin) 6D
TC0800012423.hg.1	-1.92	0.0419	PPP1R42	protein phosphatase 1, regulatory subunit 42
TC0400007055.hg.1	-1.92	0.0167	CDC42	Transcript Identified by AceView, Entrez Gene ID(s) 998
TC0200007401.hg.1	-1.92	0.0008	DYNC2LI1	dynein, cytoplasmic 2, light intermediate chain 1
TC1200012678.hg.1	-1.93	0.0012	PTPRQ	protein tyrosine phosphatase, receptor type, Q
TC0700012448.hg.1	-1.93	0.0264	RNF133	ring finger protein 133
TC0400006783.hg.1	-1.94	0.0306	USP17L10	ubiquitin specific peptidase 17-like family member 10
TC1600010004.hg.1	-1.96	0.0427	ZNF646	Memczak2013 ANTISENSE, coding, INTERNAL, intronic best transcript NM_014699
TC1100009384.hg.1	-1.97	0.0075	OR8G1	olfactory receptor, family 8, subfamily G, member 1 (gene/pseudogene)
TC0100010785.hg.1	-1.97	0.0142	RP11-12M5.1; FAM163A	Transcript Identified by AceView, Entrez Gene ID(s) 148753; novel transcript
TC1700011892.hg.1	-1.98	0.0205	TK1	thymidine kinase 1, soluble
TC1300008793.hg.1	-1.98	0.029	TSC22D1	TSC22 domain family, member 1
TC0X00010408.hg.1	-1.98	0.0117	TCEAL5	transcription elongation factor A (SII)-like 5
TC1600008008.hg.1	-1.99	0.0002	DRC7	dynein regulatory complex subunit 7

TC1000010276.hg.1	-1.99	0.0447	NRP1	Transcript Identified by AceView, Entrez Gene ID(s) 8829
TC0X00008796.hg.1	-1.99	0.0033	ABCD1	Transcript Identified by AceView, Entrez Gene ID(s) 215
TC1600011389.hg.1	-2.01	0.0482	TP53TG3C	TP53 target 3C
TC0500013239.hg.1	-2.01	0.0263	PCDHB3	protocadherin beta 3
TC1700009857.hg.1	-2.02	0.0172	TEKT3	tektin 3
TC1500007804.hg.1	-2.05	0.0086	BBS4	Bardet-Biedl syndrome 4
TC1100009407.hg.1	-2.05	0.0269	CCDC15	coiled-coil domain containing 15
TC0600013199.hg.1	-2.05	0.0389	TAAR3	trace amine associated receptor 3 (gene/pseudogene)
TC0300013950.hg.1	-2.05	0.0197	EFHB	EF-hand domain family, member B
TC0100012942.hg.1	-2.05	0.0205	PRAMEF18	PRAME family member 18
TC1200012833.hg.1	-2.08	0.0012	PAH	phenylalanine hydroxylase
TC1200009252.hg.1	-2.08	0.0381	TCTN2	tectonic family member 2
TC1000012108.hg.1	-2.08	0.011	CPXM2	carboxypeptidase X (M14 family), member 2
TC0700007112.hg.1	-2.08	0.0496	PPP1R17	protein phosphatase 1, regulatory subunit 17
TC0700013254.hg.1	-2.09	0.0108	PTPRN2	protein tyrosine phosphatase, receptor type, N polypeptide 2
TC1900007228.hg.1	-2.11	0.0304	AC006128.2; AKAP8L	Memczak2013 ANTISENSE, coding, INTERNAL, intronic best transcript NM_014371; TEC
TC1600007575.hg.1	-2.11	0.0057	TP53TG3D	TP53 target 3D
TC1100013059.hg.1	-2.14	0.0077	MYEOV	myeloma overexpressed
TC0X00010468.hg.1	-2.14	0.0217	FRMPD3-AS1	FRMPD3 antisense RNA 1
TC0X00007229.hg.1	-2.14	0.0329	GAGE12F; GAGE12J; GAGE12D; GAGE5; GAGE6; GAGE12B; GAGE4; GAGE2E	G antigen 12F; G antigen 12J; G antigen 12D; G antigen 5; G antigen 6; G antigen 12B; G antigen 4; G antigen 2E
TC0300013665.hg.1	-2.14	0.0322	MUC4	mucin 4, cell surface associated

TC0900011064.hg.1	-2.15	0.032	OR13C4	olfactory receptor, family 13, subfamily C, member 4
TC1600006666.hg.1	-2.16	0.0196	CASP16P	caspase 16, pseudogene
TC0800006488.hg.1	-2.16	0.0317	ARHGEF10	Jeck2013 ALT_ACCEPTOR, ALT_DONOR, coding, INTERNAL, intronic best transcript NM_014629
TC1100011234.hg.1	-2.24	0.0174	LTBP3	latent transforming growth factor beta binding protein 3
TC1100006911.hg.1	-2.24	0.0165	SPON1	spondin 1, extracellular matrix protein
TC1800008804.hg.1	-2.26	0.0314	ALPK2	alpha kinase 2
TC0100015853.hg.1	-2.26	0.0043	SPRR2G	small proline-rich protein 2G
TC1100009649.hg.1	-2.33	0.0257	SIRT3	sirtuin 3
TC0100016296.hg.1	-2.33	0.0141	GPA33	glycoprotein A33 (transmembrane)
TC0300007014.hg.1	-2.37	0.0029	ARPP21	Transcript Identified by AceView, Entrez Gene ID(s) 10777
TC1000008658.hg.1	-2.38	0.0113	PAX2	paired box 2
TC1100009618.hg.1	-2.39	0.0408	GLB1L2	galactosidase beta 1 like 2
TC0900008888.hg.1	-2.39	0.0127	ENDOG	endonuclease G
TC2200007407.hg.1	-2.4	0.0015	CACNA1I	calcium channel, voltage-dependent, T type, alpha 1I subunit
TC1400010603.hg.1	-2.45	0.0024	TTC6	tetratricopeptide repeat domain 6
TC1700008350.hg.1	-2.54	0.0236	NOG	noggin
TC0200011379.hg.1	-2.59	0.0286	SNED1	sushi, nidogen and EGF-like domains 1
TC0300013068.hg.1	-2.62	0.0093	MECOM	MDS1 and EVI1 complex locus
TC2000006782.hg.1	-2.66	0.0071	OTOR	otoraplin
TC1700011007.hg.1	-2.67	0.027	TTLL6	tubulin tyrosine ligase-like family member 6
TC0300011028.hg.1	-2.77	0.0144	COL7A1; MIR711	collagen, type VII, alpha 1; microRNA 711
TC2200009345.hg.1	-2.89	0.0012	C1QTNF6	C1q and tumor necrosis factor related protein 6

TC0700007031.hg.1	-2.99	0.0447	JAZF1	Memczak2013 ANTISENSE, coding, INTERNAL, intronic best transcript NM_175061
TC0700009240.hg.1	-3.25	0.0298	AGBL3	ATP/GTP binding protein-like 3
TC1200010133.hg.1	-3.39	0.0326	SOX5	Transcript Identified by AceView, Entrez Gene ID(s) 6660

(E) CCL20 and IL-15 treated T cells

ID	FC CCL20 IL15 vs CONTROL	P-val	Gene Symbol	Description
TC0300011124.hg.1	23.64	0.0000 000349	CISH	cytokine inducible SH2-containing protein
TC1200008474.hg.1	9.48	0.0000 0024	SOCS2	suppressor of cytokine signaling 2
TC0400011052.hg.1	7.98	0.0003	CXCL9	chemokine (C-X-C motif) ligand 9
TC1400010195.hg.1	6.94	0.0384	WARS	tryptophanyl-tRNA synthetase
TC1000009691.hg.1	6.78	0.0000 000051 9	IL2RA	interleukin 2 receptor, alpha
TC0300013555.hg.1	6.38	0.002	MB21D2	Mab-21 domain containing 2
TC0100012734.hg.1	5.9	0.0367	TNFRSF9	tumor necrosis factor receptor superfamily, member 9
TC0100014857.hg.1	5.47	0.0004	GBP5	guanylate binding protein 5
TC1200007461.hg.1	5.18	0.0221	PCED1B	PC-esterase domain containing 1B
TC1800008891.hg.1	4.92	0.0009	BCL2	B-cell CLL/lymphoma 2
TC0600012036.hg.1	4.52	0.0119	IL17F	interleukin 17F
TC0100014852.hg.1	4.22	0.0001	GBP1	guanylate binding protein 1, interferon-inducible
TC0600007596.hg.1	4	0.005	TNF	tumor necrosis factor
TC1700010461.hg.1	3.87	0.0445	CCL3L3; CCL3L1	chemokine (C-C motif) ligand 3-like 3; chemokine (C-C motif) ligand 3-like 1
TC0800010685.hg.1	3.72	0.0316	MYBL1	v-myb avian myeloblastosis viral oncogene homolog-like 1
TC0X00009667.hg.1	3.61	0.0032	FOXP3	forkhead box P3
TC1400007165.hg.1	3.54	0.0000 00147	PTGER2	prostaglandin E receptor 2
TC1100011888.hg.1	3.52	0.0454	CTSC	cathepsin C
TC1000008891.hg.1	3.37	0.0028	DUSP5	dual specificity phosphatase 5
TC0100011012.hg.1	3.35	0.0092	RGS1	regulator of G-protein signaling 1

TC1200011179.hg.1	3.32	0.0282	IL22	interleukin 22
TC0600007862.hg.1	3.19	0.0034	PIM1	Pim-1 proto-oncogene, serine/threonine kinase
TC0200016548.hg.1	3.06	0.0411	GPD2	glycerol-3-phosphate dehydrogenase 2
TC0600006442.hg.1	2.98	0.0000 559	IRF4	interferon regulatory factor 4
TC1100012981.hg.1	2.77	0.0003	RCN1	reticulocalbin 1, EF-hand calcium binding domain
TC0400012458.hg.1	2.77	0.0019	HPGD	hydroxyprostaglandin dehydrogenase 15-(NAD)
TC0600011945.hg.1	2.66	0.0012	RCAN2	regulator of calcineurin 2
TC0600011674.hg.1	2.65	0.0165	PPIL1	peptidylprolyl isomerase (cyclophilin)-like 1
TC0900006559.hg.1	2.62	0.0071	CD274	CD274 molecule
TC0200009091.hg.1	2.62	0.0171	RALB	v-ral simian leukemia viral oncogene homolog B
TC2000008815.hg.1	2.61	0.0052	BCL2L1	BCL2-like 1
TC1200008906.hg.1	2.56	0.0216	TRAFD1	TRAF-type zinc finger domain containing 1
TC0300007096.hg.1	2.55	0.0464	CCR8	chemokine (C-C motif) receptor 8
TC1900007748.hg.1	2.54	0.0454	PDCD5	programmed cell death 5
TC0700011785.hg.1	2.5	0.0042	CDK6	cyclin-dependent kinase 6
TC1600007557.hg.1	2.49	0.0005	ZNF267	zinc finger protein 267
TC0600009439.hg.1	2.49	0.0007	TMEM200A	transmembrane protein 200A
TC2200007242.hg.1	2.46	0.0099	APOL1	apolipoprotein L1
TC1900010807.hg.1	2.44	0.0016	CEACAM1	carcinoembryonic antigen-related cell adhesion molecule 1 (biliary glycoprotein)
TC1600008139.hg.1	2.42	0.0000 25	CES4A	carboxylesterase 4A
TC0100018555.hg.1	2.36	0.0456	LEMD1	LEM domain containing 1

TC0200008071.hg.1	2.35	0.0435	MTHFD2	methylenetetrahydrofolate dehydrogenase (NADP+ dependent) 2, methenyltetrahydrofolate cyclohydrolase
TC0X00011159.hg.1	2.33	0.0018	HAUS7; TREX2	HAUS augmin like complex subunit 7; three prime repair exonuclease 2
TC0200015242.hg.1	2.32	0.0005	STAT1	signal transducer and activator of transcription 1
TC1900011779.hg.1	2.31	0.0026	FLT3LG	fms-related tyrosine kinase 3 ligand
TC0200008666.hg.1	2.31	0.0201	IL1R1	interleukin 1 receptor, type I
TC0100014855.hg.1	2.29	0.0203	GBP4	guanylate binding protein 4
TC1700011787.hg.1	2.27	0.0011	RNF157	ring finger protein 157
TC0200008351.hg.1	2.25	0.0228	RPIA	ribose 5-phosphate isomerase A
TSUnmapped00000076.hg.1	2.25	0.0364	SURF4	surfeit 4
TC0300011049.hg.1	2.25	0.0404	QRICH1	Transcript Identified by AceView, Entrez Gene ID(s) 54870
TC0700009232.hg.1	2.24	0.0007	BPGM	2,3-bisphosphoglycerate mutase
TC0800007210.hg.1	2.23	0.0488	WRN	Transcript Identified by AceView, Entrez Gene ID(s) 7486
TC0100017834.hg.1	2.22	0.0025	GNG4	guanine nucleotide binding protein (G protein), gamma 4
TC0600008027.hg.1	2.17	0.0015	BYSL	bystin-like
TC0600011439.hg.1	2.17	0.0392	NCR3	natural cytotoxicity triggering receptor 3
TC0700010941.hg.1	2.16	0.0104	MYO1G	myosin IG
TSUnmapped00000573.hg.1	2.15	0.0061	PRAMEF25	PRAME family member 25
TC0600008247.hg.1	2.13	0.0373	IL17A	interleukin 17A
TC0200009813.hg.1	2.11	0.0002	PSMD14	proteasome 26S subunit, non-ATPase 14
TC0900011302.hg.1	2.11	0.0003	TNFSF8	tumor necrosis factor (ligand) superfamily, member 8

TC1100012510.hg.1	2.11	0.0335	HYOU1	hypoxia up-regulated 1
TC0100006494.hg.1	2.11	0.0346	B3GALT6	UDP-Gal:betaGal beta 1,3-galactosyltransferase 6
TC0400007918.hg.1	2.09	0.0063	CXCL13	chemokine (C-X-C motif) ligand 13
TC1600006658.hg.1	2.09	0.0278	IL32	interleukin 32
TC0600014255.hg.1	2.08	0.0119	UBD	ubiquitin D
TC2200006985.hg.1	2.05	0.0173	PITPNB	Memczak2013 ANTISENSE, coding, INTERNAL, intronic best transcript NM_012399
TC0200011731.hg.1	2.05	0.0388	PDIA6	protein disulfide isomerase family A, member 6
TC0600007769.hg.1	2.04	0.0076	SNRPC	small nuclear ribonucleoprotein polypeptide C
TC0100007481.hg.1	2.03	0.0307	PIGV	phosphatidylinositol glycan anchor biosynthesis class V
TC0X00007937.hg.1	2.02	0.0143	DRP2	dystrophin related protein 2
TC0600011733.hg.1	2.02	0.0287	KCNK5	potassium channel, two pore domain subfamily K, member 5
TC1500006963.hg.1	2.01	0.0015	CASC5	cancer susceptibility candidate 5
TC0500012599.hg.1	2.01	0.037	ADAM19	ADAM metallopeptidase domain 19
TC1400010721.hg.1	1.99	0.0239	PSME2; MIR7703	proteasome activator subunit 2; microRNA 7703
TC2200008370.hg.1	1.99	0.0355	XBP1	X-box binding protein 1
TC1400009239.hg.1	1.96	0.0269	GCH1	GTP cyclohydrolase 1
TC0100007512.hg.1	1.96	0.0281	GPR3	G protein-coupled receptor 3
TC1700010618.hg.1	1.96	0.0362	TOP2A	topoisomerase (DNA) II alpha
TC0800011472.hg.1	1.95	0.017	NUDCD1	NudC domain containing 1
TC0300010643.hg.1	1.95	0.0335	CMTM6	CKLF-like MARVEL transmembrane domain containing 6
TC0400008444.hg.1	1.95	0.0349	LARP7	Zhang2013 ALT_DONOR, coding, INTERNAL, intronic, OVERLAPTX, OVEXON best transcript NM_016648

TC1400006446.hg.1	1.94	0.0084	OR11H12	olfactory receptor, family 11, subfamily H, member 12
TC0600010688.hg.1	1.94	0.0496	LYRM4	LYR motif containing 4
TC0900011177.hg.1	1.92	0.0101	TXN	thioredoxin
TC0100012778.hg.1	1.92	0.0153	ENO1	enolase 1, (alpha)
TC1500007196.hg.1	1.91	0.035	SLC27A2	solute carrier family 27 (fatty acid transporter), member 2
TC0500012662.hg.1	1.91	0.0378	ZBED8	zinc finger, BED-type containing 8
TC0700006984.hg.1	1.89	0.0458	SNX10	sorting nexin 10
TC1400008058.hg.1	1.88	0.0361	PPP4R4	protein phosphatase 4, regulatory subunit 4
TC0300007437.hg.1	1.87	0.0013	MANF	mesencephalic astrocyte-derived neurotrophic factor
TC1000007081.hg.1	1.87	0.0101	PDSS1	prenyl (decaprenyl) diphosphate synthase, subunit 1
TC0100009370.hg.1	1.87	0.0243	UBL4B	ubiquitin-like 4B
TSUnmapped00000383.hg.1	1.86	0.0192	HYOU1	hypoxia up-regulated 1
TC1700010036.hg.1	1.86	0.0338	ALDH3A1	aldehyde dehydrogenase 3 family, member A1
TC2200007214.hg.1	1.84	0.0221	APOL6	apolipoprotein L, 6
TC0300013911.hg.1	1.83	0.0319	DNAJB11	DnaJ (Hsp40) homolog, subfamily B, member 11
TC0300006483.hg.1	1.82	0.0088	BHLHE40	basic helix-loop-helix family, member e40
TC0400012881.hg.1	1.82	0.0164	DUX4	double homeobox 4
TC2000006642.hg.1	1.82	0.0167	BMP2	bone morphogenetic protein 2
TC1600009395.hg.1	1.82	0.0182	SOCS1	suppressor of cytokine signaling 1
TC2200008592.hg.1	1.82	0.0297	APOL2	apolipoprotein L, 2
TC1100007610.hg.1	1.81	0.0003	OR5D14	olfactory receptor, family 5, subfamily D, member 14

TC0400011043.hg.1	1.81	0.0135	CDKL2	cyclin-dependent kinase-like 2 (CDC2-related kinase)
TC0400007592.hg.1	1.8	0.0328	PAICS	phosphoribosylaminoimidazole carboxylase, phosphoribosylaminoimidazole succinocarboxamide synthetase
TSUnmapped00000558.hg.1	1.79	0.0071	HYOU1	hypoxia up-regulated 1
TC0300012812.hg.1	1.78	0.0282	GPR171	G protein-coupled receptor 171
TC1200008642.hg.1	1.77	0.0009	PARPBP	PARP1 binding protein
TC1000008954.hg.1	1.77	0.0094	CASP7	caspase 7
TSUnmapped00000484.hg.1	1.77	0.0157	HYOU1	hypoxia up-regulated 1
TC1600008633.hg.1	1.76	0.0043	NECAB2	N-terminal EF-hand calcium binding protein 2
TC0100014769.hg.1	1.75	0.016	MCOLN3	mucolipin 3
TC0300008713.hg.1	1.74	0.0076	SEC61A1	Sec61 translocon alpha 1 subunit
TC1200012576.hg.1	1.74	0.0188	NDUFA9	NADH dehydrogenase (ubiquinone) 1 alpha subcomplex, 9, 39kDa
TC1400006823.hg.1	1.74	0.0258	SCFD1	sec1 family domain containing 1
TC1200011711.hg.1	1.73	0.0005	GNPTAB	N-acetylglucosamine-1-phosphate transferase, alpha and beta subunits
TC0100012884.hg.1	1.73	0.0092	FBXO2	F-box protein 2
TC0600008539.hg.1	1.72	0.0494	CD109	CD109 molecule
TC0800007431.hg.1	1.71	0.0043	AP3M2	adaptor-related protein complex 3, mu 2 subunit
TC1800007218.hg.1	1.71	0.016	C18orf25	chromosome 18 open reading frame 25
TC0900008207.hg.1	1.7	0.0097	SEC61B	Sec61 translocon beta subunit
TC0200012031.hg.1	1.7	0.0266	HADHA	Transcript Identified by AceView, Entrez Gene ID(s) 3030

TC0700008857.hg.1	1.7	0.0471	MDFIC	MyoD family inhibitor domain containing
TC0800008263.hg.1	1.68	0.0241	ESRP1	epithelial splicing regulatory protein 1
TSUnmapped00000384.hg.1	1.67	0.0066	ZNF546	zinc finger protein 546
TC0200010157.hg.1	1.67	0.0147	ITGA4	integrin alpha 4
TC0100011073.hg.1	1.67	0.0165	CRB1	crumbs family member 1, photoreceptor morphogenesis associated
TC1800007367.hg.1	1.67	0.0181	TCF4	Memczak2013 ANTISENSE, coding, INTERNAL, intronic best transcript NM_001243234
TC0700013324.hg.1	1.65	0.0056	GET4	golgi to ER traffic protein 4
TSUnmapped00000489.hg.1	1.65	0.0093	SURF4	surfeit 4
TC1500010006.hg.1	1.65	0.0165	STRA6	stimulated by retinoic acid 6
TC0400012846.hg.1	1.64	0.0494	NAA15	N(alpha)-acetyltransferase 15, NatA auxiliary subunit
TC0200009806.hg.1	1.63	0.0087	TANK	TRAF family member-associated NFKB activator
TC0500009409.hg.1	1.63	0.038	GABRP	gamma-aminobutyric acid (GABA) A receptor, pi
TC0100014014.hg.1	1.63	0.0427	PRDX1	peroxiredoxin 1
TC0800010043.hg.1	1.62	0.0049	PPP2CB	protein phosphatase 2, catalytic subunit, beta isozyme
TC0100012453.hg.1	1.62	0.0088	TNFRSF4	tumor necrosis factor receptor superfamily, member 4
TC0X00007767.hg.1	1.62	0.0116	POU3F4	POU class 3 homeobox 4
TC0500012105.hg.1	1.62	0.019	NEUROG1	neurogenin 1
TC1400007644.hg.1	1.62	0.0353	ACOT1	acyl-CoA thioesterase 1
TC0X00006723.hg.1	1.61	0.0148	CDKL5	cyclin-dependent kinase-like 5

TC1900008880.hg.1	1.61	0.0486	KIR2DL4	killer cell immunoglobulin-like receptor, two domains, long cytoplasmic tail, 4
TC2200007780.hg.1	1.6	0.0078	CRELD2	cysteine rich with EGF-like domains 2
TC1700006644.hg.1	1.6	0.0292	PSMB6	proteasome subunit beta 6
TC1400009002.hg.1	1.59	0.0046	SEC23A	Sec23 homolog A, COPII coat complex component
TC2000009550.hg.1	1.59	0.0251	AURKA	aurora kinase A
TC1100009450.hg.1	1.58	0.0286	DCPS	decapping enzyme, scavenger
TC0600008972.hg.1	1.58	0.0294	PRDM1	PR domain containing 1, with ZNF domain
TC1300009088.hg.1	1.58	0.0328	DIAPH3	diaphanous-related formin 3
TC0600009140.hg.1	1.58	0.0396	GTF3C6	general transcription factor IIIC subunit 6
TC0300013831.hg.1	1.58	0.047	SEMA3B; MIR6872	sema domain, immunoglobulin domain (Ig), short basic domain, secreted, (semaphorin) 3B; microRNA 6872
TC1200010759.hg.1	1.57	0.0205	KRT5	keratin 5, type II
TC1600009040.hg.1	1.57	0.0396	C16orf91	chromosome 16 open reading frame 91
TC1100010236.hg.1	1.57	0.046	KCNJ11	potassium channel, inwardly rectifying subfamily J, member 11
TC0600011507.hg.1	1.57	0.0484	PSMB8	proteasome subunit beta 8
TC2100008272.hg.1	1.57	0.0488	WDR4	WD repeat domain 4
TC0300012880.hg.1	1.56	0.0422	C3orf33	chromosome 3 open reading frame 33
TC1200008298.hg.1	1.56	0.0434	MYF6	myogenic factor 6 (herculin)
TC1300007780.hg.1	1.56	0.0441	RAP2A	RAP2A, member of RAS oncogene family
TC0X00008940.hg.1	1.56	0.046	MXRA5	matrix-remodelling associated 5
TC0X00010678.hg.1	1.55	0.0168	C1GALT1C1	C1GALT1 specific chaperone 1
TC1200011656.hg.1	1.55	0.0467	IKBIP	IKBKB interacting protein

TC0700011905.hg.1	1.54	0.0126	BAIAP2L1	BAI1-associated protein 2-like 1
TC1300006729.hg.1	1.54	0.0211	POMP	proteasome maturation protein
TC0100011913.hg.1	1.54	0.0304	URB2	URB2 ribosome biogenesis 2 homolog (S. cerevisiae)
TC0100008996.hg.1	1.54	0.0387	ZNF326	zinc finger protein 326
TC1500009926.hg.1	1.54	0.045	LARP6	La ribonucleoprotein domain family, member 6
TC0100010648.hg.1	1.53	0.0127	PRDX6	peroxiredoxin 6
TC1600006653.hg.1	1.53	0.0336	THOC6	THO complex 6
TC0X00008716.hg.1	1.53	0.0422	MTM1	myotubularin 1
TC1200012722.hg.1	1.53	0.0468	FAM101A	family with sequence similarity 101, member A
TC2200009356.hg.1	1.52	0.0166	ARFGAP3	ADP-ribosylation factor GTPase activating protein 3
TC0600007087.hg.1	1.52	0.0301	CAP2	CAP, adenylate cyclase-associated protein, 2 (yeast)
TC0500007731.hg.1	1.52	0.0318	CARTPT	CART prepropeptide
TC0X00007256.hg.1	1.52	0.0375	BMP15	bone morphogenetic protein 15
TC0X00010770.hg.1	1.51	0.0258	ACTRT1	actin-related protein T1
TC2200009361.hg.1	1.51	0.038	MAPK12	mitogen-activated protein kinase 12
TC2000008860.hg.1	1.51	0.0484	COMMD7	COMM domain containing 7
TC1700008719.hg.1	1.5	0.021	KPNA2	karyopherin alpha 2 (RAG cohort 1, importin alpha 1)
TC0400008668.hg.1	1.5	0.0225	LARP1B	La ribonucleoprotein domain family, member 1B
TC0500006494.hg.1	1.49	0.0192	SLC6A18	solute carrier family 6 (neutral amino acid transporter), member 18
TC0400012029.hg.1	1.49	0.0257	OTUD4	OTU deubiquitinase 4
TC0500013230.hg.1	1.49	0.0276	PCBD2	pterin-4 alpha-carbinolamine dehydratase/dimerization cofactor of hepatocyte nuclear factor 1 alpha (TCF1) 2

TC0800008371.hg.1	1.49	0.0364	SPAG1	sperm associated antigen 1
TC0X00006739.hg.1	1.49	0.0484	PDHA1	pyruvate dehydrogenase (lipoamide) alpha 1
TC1100010757.hg.1	1.48	0.0213	MTCH2	mitochondrial carrier 2
TC0200008230.hg.1	1.48	0.0282	TMSB10	thymosin beta 10
TC0100018484.hg.1	1.47	0.0187	MCL1	myeloid cell leukemia 1
TC0100010686.hg.1	1.47	0.0459	CACYBP	calcyclin binding protein
TC0400008424.hg.1	1.46	0.0082	C4orf32	chromosome 4 open reading frame 32
TC1200009672.hg.1	1.46	0.0083	C12orf4	chromosome 12 open reading frame 4
TC1600008402.hg.1	1.46	0.0233	PSMD7	proteasome 26S subunit, non-ATPase 7
TC0700007596.hg.1	1.46	0.0282	EGFR	epidermal growth factor receptor
TC0100016476.hg.1	1.46	0.0306	KIAA0040	KIAA0040
TC0700013543.hg.1	1.46	0.0334	C7orf25	chromosome 7 open reading frame 25
TC0100016406.hg.1	1.46	0.0338	VAMP4	vesicle associated membrane protein 4
TC1500008202.hg.1	1.46	0.035	AGBL1	ATP/GTP binding protein-like 1
TC0700010621.hg.1	1.46	0.0393	FKBP14	FK506 binding protein 14
TC1200009247.hg.1	1.46	0.0447	TMED2	transmembrane p24 trafficking protein 2
TC1400008684.hg.1	1.45	0.0365	PRMT5	protein arginine methyltransferase 5
TC1500008978.hg.1	1.44	0.0097	EMC7	ER membrane protein complex subunit 7
TC0100018315.hg.1	1.44	0.0169	UCK2; MIR3658	uridine-cytidine kinase 2; microRNA 3658
TC0X00008532.hg.1	1.44	0.0203	BRS3	bombesin-like receptor 3
TC1100009098.hg.1	1.44	0.0306	HTR3A	5-hydroxytryptamine (serotonin) receptor 3A, ionotropic
TC0500010085.hg.1	1.44	0.0445	CMBL	carboxymethylenebutenolidase homolog (Pseudomonas)
TSUnmapped00000690.hg.1	1.44	0.0465	LRIG1	

TC1700007790.hg.1	1.44	0.0494	CDC6	cell division cycle 6
TSUnmapped00000487.hg.1	1.43	0.0112	MANSC1	MANSC domain containing 1
TC2200008465.hg.1	1.43	0.018	SELM	selenoprotein M; selenoprotein M [Source:EntrezGene;Acc:140606]; Salzman2013 ANNOTATED, CDS, coding, OVCODE, OVERLAPTX, OVEXON, UTR3 best transcript NM_080430; Salzman2013 ANNOTATED, CDS, coding, OVCODE, OVERLAPTX, OVEXON, UTR3, UTR5 best transcript NM_080430; Transcript Identified by AceView, Entrez Gene ID(s) 140606
TC1000012423.hg.1	1.43	0.0283	PFKP	phosphofructokinase, platelet
TC1000008431.hg.1	1.42	0.0133	PCGF5	polycomb group ring finger 5
TC0100015417.hg.1	1.42	0.0387	VTCN1	V-set domain containing T cell activation inhibitor 1
TC1900011969.hg.1	1.42	0.044	IRGQ	immunity-related GTPase family, Q
TC0100006926.hg.1	1.41	0.0342	PRAMEF20	PRAME family member 20
TC1600009257.hg.1	1.41	0.0382	C16orf89	chromosome 16 open reading frame 89
TC2200008427.hg.1	1.41	0.0386	OSM	oncostatin M
TC1200012635.hg.1	1.41	0.0431	PRR13	proline rich 13
TSUnmapped00000734.hg.1	1.4	0.0221	HYOU1	hypoxia up-regulated 1
TC2200007909.hg.1	1.4	0.0251	MICAL3	microtubule associated monooxygenase, calponin and LIM domain containing 3
TC0400006714.hg.1	1.4	0.0312	TADA2B	Transcript Identified by AceView, Entrez Gene ID(s) 93624

TC0500013385.hg.1	1.4	0.0184	SH3TC2	SH3 domain and tetratricopeptide repeats 2
TC1500007901.hg.1	1.4	0.0209	SNX33	sorting nexin 33
TC0100015265.hg.1	1.4	0.0211	DENND2D	DENN/MADD domain containing 2D
TC0200007825.hg.1	-1.41	0.0224	CEP68	centrosomal protein 68kDa
TC0100012113.hg.1	-1.41	-1.41	RYR2	ryanodine receptor 2 (cardiac)
TC1700009811.hg.1	-1.41	-1.41	HS3ST3A1	Jeck2013 ALT_ACCEPTOR, ALT_DONOR, coding, INTERNAL, intronic best transcript NM_006042
TC0600010799.hg.1	-1.41	-1.41	GCM2	glial cells missing homolog 2 (Drosophila)
TSUnmapped00000801.hg.1	-1.43	0.0146	VPS11	VPS11, CORVET/HOPS core subunit [Source:HGNC Symbol;Acc:HGNC:14583]
TC0X00010051.hg.1	-1.43	0.0176	NAP1L6	nucleosome assembly protein 1-like 6
TC1900011201.hg.1	-1.43	0.0386	FAM71E1	family with sequence similarity 71, member E1
TC0600009961.hg.1	-1.44	0.0251	ARID1B	AT rich interactive domain 1B (SWI1-like)
TC0700013178.hg.1	-1.44	0.0353	BLACE	B-cell acute lymphoblastic leukemia expressed
TC0200016571.hg.1	-1.45	0.0064	OSBPL6	oxysterol binding protein-like 6
TC0600011823.hg.1	-1.45	0.0216	TRERF1	transcriptional regulating factor 1
TC1200012578.hg.1	-1.45	0.0249	KCNA6; GALNT8	potassium channel, voltage gated shaker related subfamily A, member 6; polypeptide N-acetylgalactosaminyltransferase 8
TC1200007526.hg.1	-1.45	0.0295	LINC00935	long intergenic non-protein coding RNA 935

TC1600009928.hg.1	-1.45	0.0305	LOC388242; LOC613038; RP11-345J4.3	SAGA complex associated factor 29 pseudogene; Transcript Identified by AceView, Entrez Gene ID(s) 388242; Uncharacterized protein [Source:UniProtKB/TrEMBL;Acc:H0YHM3]
TC1100012535.hg.1	-1.45	0.0496	PVRL1	poliovirus receptor-related 1 (herpesvirus entry mediator C)
TC1100007167.hg.1	-1.46	0.0174	FSHB	follicle stimulating hormone, beta polypeptide
TC1100012722.hg.1	-1.46	0.0179	CDON	cell adhesion associated, oncogene regulated
TC0100007660.hg.1	-1.46	0.032	TMEM39B	transmembrane protein 39B
TC1600011517.hg.1	-1.46	0.0322	DOC2A	double C2-like domains, alpha
TC1600009957.hg.1	-1.46	0.044	LOC388242; LOC613038; RP11-347C12.3	Homo sapiens coiled-coil domain containing 101 pseudogene (LOC388242), non-coding RNA.; Homo sapiens SAGA complex associated factor 29 pseudogene (LOC613038), non-coding RNA.; Transcript Identified by AceView, Entrez Gene ID(s) 613038, RefSeq ID(s) NR_002557; Transcript Identified by AceView, Entrez Gene ID(s) 613038; Protein LOC101929849 [Source:UniProtKB/TrEMBL;Acc:H0YHN6]
TC1200009914.hg.1	-1.47	0.0094	TAS2R7	taste receptor, type 2, member 7
TC0200015574.hg.1	-1.47	0.03	CRYGA	crystallin gamma A
TC0200016413.hg.1	-1.47	0.0326	FKBP1B	FK506 binding protein 1B
TC1700008946.hg.1	-1.47	0.0394	AANAT	aralkylamine N-acetyltransferase

TC0400006800.hg.1	-1.48	0.0324	USP17L18; USP17L11	ubiquitin specific peptidase 17-like family member 18; ubiquitin specific peptidase 17-like family member 11
TC0900011613.hg.1	-1.48	0.0396	FAM102A	family with sequence similarity 102, member A
TC0300013915.hg.1	-1.49	0.0058	TPRG1	tumor protein p63 regulated 1
TC0500009303.hg.1	-1.49	0.0071	GABRA1	gamma-aminobutyric acid (GABA) A receptor, alpha 1
TC1700008440.hg.1	-1.49	0.0103	PPM1E	protein phosphatase, Mg2+/Mn2+ dependent, 1E
TC1700010565.hg.1	-1.49	0.0163	FBXL20	F-box and leucine-rich repeat protein 20
TC1200011855.hg.1	-1.49	0.0229	SVOP	SV2 related protein
TC2000006964.hg.1	-1.49	0.0396	CST8	cystatin 8 (cystatin-related epididymal specific)
TC0900007129.hg.1	-1.49	0.0412	MELK	maternal embryonic leucine zipper kinase
TC0700013530.hg.1	-1.49	0.0442	HOXA4	homeobox A4
TC0700012456.hg.1	-1.49	0.0472	IQUB	IQ motif and ubiquitin domain containing
TC0600011465.hg.1	-1.5	0.0221	EHMT2	euchromatic histone-lysine N-methyltransferase 2
TC2000009624.hg.1	-1.5	0.0295	APCDD1L	adenomatosis polyposis coli down-regulated 1-like
TC0600010263.hg.1	-1.5	0.0435	SMOC2	SPARC related modular calcium binding 2
TC0100018207.hg.1	-1.5	0.0441	TSSK3	testis-specific serine kinase 3
TC1600007308.hg.1	-1.5	0.0493	KDM8	lysine (K)-specific demethylase 8
TC1400010058.hg.1	-1.51	0.0142	GSC	goosecoid homeobox
TC1300008072.hg.1	-1.51	0.0395	SPACA7	sperm acrosome associated 7
TC0100016902.hg.1	-1.52	0.0208	ASCL5	achaete-scute family bHLH transcription factor 5
TC0300013813.hg.1	-1.53	0.0137	ACKR2	atypical chemokine receptor 2

TC0200009700.hg.1	-1.53	0.0161	GALNT13	polypeptide N-acetylgalactosaminyltransferase 13
TC1200010921.hg.1	-1.53	0.0389	GLS2	glutaminase 2 (liver, mitochondrial)
TC1100013032.hg.1	-1.53	0.0442	DNAJC4	DnaJ (Hsp40) homolog, subfamily C, member 4
TC1100007650.hg.1	-1.53	0.0467	OR9G1; OR9G9	olfactory receptor, family 9, subfamily G, member 1; olfactory receptor, family 9, subfamily G, member 9
TC0100014133.hg.1	-1.54	0.0019	AGBL4	ATP/GTP binding protein-like 4
TC1100012314.hg.1	-1.54	0.0171	PIH1D2	PIH1 domain containing 2
TC0200013108.hg.1	-1.54	0.0187	LBX2	ladybird homeobox 2
TC1200011083.hg.1	-1.54	0.0427	C12orf56	chromosome 12 open reading frame 56
TC1900008547.hg.1	-1.54	0.0455	PRR12	proline rich 12
TC1100010661.hg.1	-1.55	0.0353	CHST1	carbohydrate (keratan sulfate Gal-6) sulfotransferase 1
TC0600007289.hg.1	-1.55	0.0497	HIST1H4F	histone cluster 1, H4f
TC1100006911.hg.1	-1.56	0.0137	SPON1	spondin 1, extracellular matrix protein
TC0X00011221.hg.1	-1.56	0.0396	GAB3	GRB2-associated binding protein 3
TSUnmapped00000271.hg.1	-1.56	0.0421	OR10A3	olfactory receptor, family 10, subfamily A, member 3
TC1900009943.hg.1	-1.56	0.0457	HAUS8	HAUS augmin like complex subunit 8
TC1200011993.hg.1	-1.57	0.0008	SLC8B1	solute carrier family 8 (sodium/lithium/calcium exchanger), member B1
TC0200010800.hg.1	-1.57	0.0157	VIL1	villin 1
TC0X00007516.hg.1	-1.57	0.0438	LINC00269	long intergenic non-protein coding RNA 269
TC0900006945.hg.1	-1.57	0.0472	TOPORS-AS1; GVQW1	TOPORS antisense RNA 1; GVQW motif containing 1
TC1200010839.hg.1	-1.58	0.0027	ITGA5	integrin alpha 5
TC0700013051.hg.1	-1.58	0.015	ATG9B	autophagy related 9B

TC1400010599.hg.1	-1.58	0.0173	TTC6	tetratricopeptide repeat domain 6
TC0200012776.hg.1	-1.58	0.0213	TMEM17	transmembrane protein 17
TC0900010854.hg.1	-1.58	0.0242	NUTM2F	NUT family member 2F
TC1100010123.hg.1	-1.58	0.0373	DKK3	dickkopf WNT signaling pathway inhibitor 3
TC1100013053.hg.1	-1.58	0.0459	RAD9A	RAD9 checkpoint clamp component A
TC2200009276.hg.1	-1.59	0.0313	SGSM3	small G protein signaling modulator 3
TC0800008487.hg.1	-1.6	0.0027	RIMS2	regulating synaptic membrane exocytosis 2
TC0300006513.hg.1	-1.6	0.0197	GRM7	glutamate receptor, metabotropic 7
TC0100014790.hg.1	-1.6	0.045	COL24A1	collagen, type XXIV, alpha 1
TC1100008054.hg.1	-1.61	0.009	PACS1	phosphofurin acidic cluster sorting protein 1
TC1100012105.hg.1	-1.61	0.0318	ANGPTL5	angiopoietin like 5
TC1100011202.hg.1	-1.61	0.0381	TMEM262	transmembrane protein 262
TC1900011871.hg.1	-1.61	0.0488	ZNF653	zinc finger protein 653
TC2100007904.hg.1	-1.61	0.0118	KRTAP22-2	keratin associated protein 22-2
TC1100008264.hg.1	-1.62	0.0168	PPFIA1	protein tyrosine phosphatase, receptor type, f polypeptide (PTPRF), interacting protein (liprin), alpha 1
TC0300009302.hg.1	-1.62	0.0257	PQLC2L	PQ loop repeat containing 2-like
TC1700008423.hg.1	-1.62	0.0484	OR4D2	olfactory receptor, family 4, subfamily D, member 2
TC1700010252.hg.1	-1.63	0.0325	CORO6	coronin 6
TC0400012768.hg.1	-1.63	0.0354	FAM200B	family with sequence similarity 200, member B
TC2000008807.hg.1	-1.63	0.0359	DEFB119	defensin, beta 119
TC1400010604.hg.1	-1.64	0.0094	MIA2	melanoma inhibitory activity 2
TC1300009906.hg.1	-1.64	0.0143	ADPRHL1	ADP-ribosylhydrolase like 1
TC1700010961.hg.1	-1.64	0.0263	SCRN2	secernin 2

TC1900011965.hg.1	-1.64	0.0379	PSG2	pregnancy specific beta-1-glycoprotein 2
TC0700013182.hg.1	-1.64	0.0404	CNPY1	canopy FGF signaling regulator 1
TC0900009922.hg.1	-1.65	0.005	FAM205C	family with sequence similarity 205, member C
TC0700009519.hg.1	-1.65	0.0186	OR2A25	olfactory receptor, family 2, subfamily A, member 25
TC2000008052.hg.1	-1.65	0.0217	BIRC7	baculoviral IAP repeat containing 7
TC0200014361.hg.1	-1.65	0.044	LYPD1	LY6/PLAUR domain containing 1
TSUnmapped00000292.hg.1	-1.66	0.0237	TGM4	transglutaminase 4
TC0100007747.hg.1	-1.66	0.0283	GJB3	gap junction protein beta 3
TSUnmapped00000234.hg.1	-1.66	0.0443	HMBS	hydroxymethylbilane synthase
TC1700009640.hg.1	-1.66	0.0462	ZBTB4	zinc finger and BTB domain containing 4
TC1500008670.hg.1	-1.67	0.0265	POTEB	POTE ankyrin domain family, member B
TC2000007516.hg.1	-1.67	0.0371	SLC12A5	solute carrier family 12 (potassium/chloride transporter), member 5
TC0500013220.hg.1	-1.67	0.0421	FAM170A	family with sequence similarity 170, member A
TC0800008349.hg.1	-1.67	0.0437	OSR2	odd-skipped related transciption factor 2
TC0100017064.hg.1	-1.68	0.0081	NUAK2	NUAK family, SNF1-like kinase, 2
TC1100013230.hg.1	-1.68	0.0365	BCL9L	B-cell CLL/lymphoma 9-like
TC0600012329.hg.1	-1.68	0.0445	IMPG1	interphotoreceptor matrix proteoglycan 1
TSUnmapped00000135.hg.1	-1.69	0.0249	INPP5D	inositol polyphosphate-5-phosphatase D

TSUnmapped00000580.hg.1	-1.72	0.0043	INPP5D	inositol polyphosphate-5-phosphatase D
TC1600010174.hg.1	-1.72	0.0288	MYLK3	myosin light chain kinase 3
TC0800011148.hg.1	-1.72	0.0317	CCNE2	cyclin E2
TC1500009261.hg.1	-1.72	0.0426	SPG11	spastic paraplegia 11 (autosomal recessive)
TC0100015872.hg.1	-1.73	0.0058	S100A14	S100 calcium binding protein A14
TC0400006783.hg.1	-1.74	0.0075	USP17L10	ubiquitin specific peptidase 17-like family member 10
TC1100007770.hg.1	-1.74	0.0314	MS4A5	membrane-spanning 4-domains, subfamily A, member 5
TC1400008752.hg.1	-1.74	0.0453	CIDEB	cell death-inducing DFFA-like effector b
TC1500010769.hg.1	-1.74	0.0468	GOLGA6C	golgin A6 family, member C
TC1400007440.hg.1	-1.75	0.0193	MTHFD1	methylenetetrahydrofolate dehydrogenase (NADP+ dependent) 1, methenyltetrahydrofolate cyclohydrolase, formyltetrahydrofolate synthetase
TC0600014074.hg.1	-1.75	0.0231	HIVEP1	human immunodeficiency virus type I enhancer binding protein 1
TC0100016021.hg.1	-1.75	0.0486	MRPL24	mitochondrial ribosomal protein L24
TC1700010811.hg.1	-1.75	0.0021	HDAC5	histone deacetylase 5
TC0500013064.hg.1	-1.75	0.0282	MGAT4B	mannosyl (alpha-1,3-)-glycoprotein beta-1,4-N-acetylglucosaminyltransferase, isozyme B
TC0300010228.hg.1	-1.77	0.0036	FANCD2OS	FANCD2 opposite strand
TC0200013668.hg.1	-1.77	0.0116	CREG2	cellular repressor of E1A-stimulated genes 2
TC0100015932.hg.1	-1.77	0.0159	PBXIP1	pre-B-cell leukemia homeobox interacting protein 1

TC0100015771.hg.1	-1.77	0.0166	SEMA6C	sema domain, transmembrane domain (TM), and cytoplasmic domain, (semaphorin) 6C
TC1900009421.hg.1	-1.77	0.0426	ACER1	alkaline ceramidase 1
TC0100013992.hg.1	-1.78	0.0006	TCTEX1D4	Tctex1 domain containing 4
TC1500006967.hg.1	-1.78	0.0403	GCHFR	GTP cyclohydrolase I feedback regulator
TC0400012663.hg.1	-1.79	0.0323	FLJ38576; RP11-173M11.6	uncharacterized LOC651430; uncharacterized LOC651430 [Source:EntrezGene;Acc:651430]; novel transcript
TC0800009637.hg.1	-1.8	0.009	USP17L2	ubiquitin specific peptidase 17-like family member 2
TC0300011951.hg.1	-1.8	0.0251	MORC1	MORC family CW-type zinc finger 1
TSUnmapped00000661.hg.1	-1.8	0.0465	INPP5D	inositol polyphosphate-5-phosphatase D
TC1700010891.hg.1	-1.8	0.0479	PLEKHM1; MIR4315-1	pleckstrin homology domain containing, family M (with RUN domain) member 1; microRNA 4315-1
TC0300007014.hg.1	-1.81	0.0156	ARPP21	Transcript Identified by AceView, Entrez Gene ID(s) 10777
TC0800012322.hg.1	-1.82	0.0477	CA13	carbonic anhydrase XIII
TC0300008371.hg.1	-1.82	0.0489	GRAMD1C	GRAM domain containing 1C
TC0200012404.hg.1	-1.83	0.0185	ZFP36L2	ZFP36 ring finger protein-like 2
TC0600012952.hg.1	-1.84	0.0041	FRK	fyn-related Src family tyrosine kinase
TC1900011946.hg.1	-1.84	0.01	FBXO27	F-box protein 27
TC1200008795.hg.1	-1.86	0.0022	ACACB	acetyl-CoA carboxylase beta
TC0100006931.hg.1	-1.86	0.0141	PDPN	podoplanin
TC2200007407.hg.1	-1.87	0.0156	CACNA1I	calcium channel, voltage-dependent, T type, alpha 1I subunit
TC1300008793.hg.1	-1.87	0.019	TSC22D1	TSC22 domain family, member 1

TC0200016496.hg.1	-1.87	0.0458	CNNM3	cyclin and CBS domain divalent metal cation transport mediator 3
TC0700007975.hg.1	-1.89	0.005	FKBP6	FK506 binding protein 6
TC0800007335.hg.1	-1.89	0.0494	LETM2	leucine zipper-EF-hand containing transmembrane protein 2
TC1500007753.hg.1	-1.9	0.0151	THSD4	Transcript Identified by AceView, Entrez Gene ID(s) 79875
TC0300013832.hg.1	-1.9	0.0233	CYB561D2	cytochrome b561 family, member D2
TC1100010850.hg.1	-1.91	0.0051	OR5T2	olfactory receptor, family 5, subfamily T, member 2
TC1900007829.hg.1	-1.91	0.012	GRAMD1A	GRAM domain containing 1A
TC1900009188.hg.1	-1.91	0.0422	REXO1; MIR1909	REX1, RNA exonuclease 1 homolog; microRNA 1909
TC0600011605.hg.1	-1.92	0.0097	TCP11	t-complex 11, testis-specific
TC0600007985.hg.1	-1.92	0.0142	TSPO2	translocator protein 2
TC1100012677.hg.1	-1.92	0.0309	OR8B12	olfactory receptor, family 8, subfamily B, member 12
TC0500008872.hg.1	-1.93	0.0207	PCDHB1	protocadherin beta 1
TC0600011123.hg.1	-1.93	0.0232	HIST1H4B	histone cluster 1, H4b
TC1900011031.hg.1	-1.93	0.0418	KPTN	kaptin (actin binding protein)
TC1100006661.hg.1	-1.94	0.0069	OR52I1	olfactory receptor, family 52, subfamily I, member 1
TC1700012353.hg.1	-1.94	0.0141	PER1; MIR6883	period circadian clock 1; microRNA 6883
TC1700010802.hg.1	-1.95	0.0039	MPP2	membrane protein, palmitoylated 2
TC1100009744.hg.1	-1.95	0.0055	DUSP8	dual specificity phosphatase 8
TC1100007657.hg.1	-1.95	0.0392	OR5AK2	olfactory receptor, family 5, subfamily AK, member 2
TC0700012165.hg.1	-1.95	0.0453	ATXN7L1	ataxin 7-like 1

TC0900007654.hg.1	-1.96	0.0199	GNAQ; skeyplorbu	Memczak2013 ANTISENSE, coding, INTERNAL, intronic best transcript NM_002072; Transcript Identified by AceView
TC0200007978.hg.1	-1.98	0.0094	ANKRD53	ankyrin repeat domain 53
TC1600007096.hg.1	-1.98	0.0181	TMC7	transmembrane channel like 7
TC1900006770.hg.1	-1.99	0.0071	DUS3L	Memczak2013 ANTISENSE, CDS, coding, INTERNAL best transcript NM_020175
TC0100010621.hg.1	-2.01	0.0059	C1orf105	chromosome 1 open reading frame 105
TC0100011414.hg.1	-2.03	0.0016	CR1	complement component (3b/4b) receptor 1 (Knops blood group)
TC1100013174.hg.1	-2.04	0.0067	BSCL2	Berardinelli-Seip congenital lipodystrophy 2 (seipin)
TC0200015332.hg.1	-2.05	0.0369	PGAP1	post-GPI attachment to proteins 1
TSUnmapped00000374.hg.1	-2.05	0.0494	DUSP16	dual specificity phosphatase 16
TC1100009407.hg.1	-2.06	0.0266	CCDC15	coiled-coil domain containing 15
TC0100009408.hg.1	-2.07	0.002	PIFO	primary cilia formation
TC1100013045.hg.1	-2.07	0.0047	SIPA1	signal-induced proliferation-associated 1
TC1700007520.hg.1	-2.07	0.0208	CDK5R1	cyclin-dependent kinase 5, regulatory subunit 1 (p35)
TC1200011491.hg.1	-2.1	0.0094	EPYC	epiphycan
TC0900010794.hg.1	-2.11	0.0228	OGN	osteoglycin
TC2200009345.hg.1	-2.13	0.0283	C1QTNF6	C1q and tumor necrosis factor related protein 6
TC0300013350.hg.1	-2.14	0.0292	YEATS2	Memczak2013 ANTISENSE, coding, INTERNAL, intronic best transcript NM_018023
TC1200009834.hg.1	-2.19	0.0437	MFAP5	microfibrillar associated protein 5

TC0100016339.hg.1	-2.21	0.001	DPT	dermatopontin
TC1100008899.hg.1	-2.26	0.0153	CEP126	centrosomal protein 126kDa
TC0500012017.hg.1	-2.31	0.0144	IRF1	interferon regulatory factor 1
TC0100018496.hg.1	-2.35	0.0442	PYGO2	pygopus family PHD finger 2
TC1700007402.hg.1	-2.37	0.028	FLOT2	Memczak2013 ANTISENSE, coding, INTERNAL, UTR3 best transcript NM_004475
TC0100009276.hg.1	-2.38	0.0479	AMY1A; AMY1C	amylase, alpha 1A (salivary); amylase, alpha 1C (salivary)
TC1000012198.hg.1	-2.4	0.0024	C10orf90	chromosome 10 open reading frame 90
TC1400010775.hg.1	-2.42	0.0239	EFCAB11	EF-hand calcium binding domain 11
TC0700006512.hg.1	-2.47	0.0325	INTS1	Memczak2013 ANTISENSE, CDS, coding, INTERNAL best transcript NM_001080453
TC1700010959.hg.1	-2.49	0.0456	OSBPL7	oxysterol binding protein-like 7
TC1100011234.hg.1	-2.52	0.0145	LTBP3	latent transforming growth factor beta binding protein 3
TC0X00009727.hg.1	-2.52	0.0245	SPANXN5	SPANX family, member N5
TC2000008237.hg.1	-2.53	0.0347	MAVS	Jeck2013 ANTISENSE, coding, INTERNAL, OVEXON, UTR3 best transcript NM_020746
TC0300013277.hg.1	-2.58	0.0401	PEX5L	Transcript Identified by AceView, Entrez Gene ID(s) 51555
TC1700010433.hg.1	-2.66	0.0279	SLFN14	schlafen family member 14
TSUnmapped00000422.hg.1	-2.67	0.038	VPS11	VPS11, CORVET/HOPS core subunit [Source:HGNC Symbol;Acc:HGNC:14583]
TC0100011417.hg.1	-2.7	0.0006	CR1L	complement component (3b/4b) receptor 1-like
TC1900011913.hg.1	-2.76	0.0007	LINC00663	long intergenic non-protein coding RNA 663

TC1600010004.hg.1	-2.85	0.0185	ZNF646	Memczak2013 ANTISENSE, coding, INTERNAL, intronic best transcript NM_014699
TC0400012453.hg.1	-3	0.0265	FBXO8	Transcript Identified by AceView, Entrez Gene ID(s) 26269
TC1100012574.hg.1	-3.02	0.0427	SORL1	Memczak2013 ANTISENSE, CDS, coding, INTERNAL best transcript NM_003105
TC0300012123.hg.1	-3.12	0.0061	CD80	CD80 molecule
TSUnmapped00000640.hg.1	-4.15	0.0486	FMN1	formin 1

(F) CCL20 and IL-32α treated T cells

ID	FC CCL20 IL32 vs CONTROL	P-val	Gene Symbol	Description
TC0100015608.hg.1	3.85	0.0017	LOC105371242; AC243756.1	peptidyl-prolyl cis-trans isomerase A-like 4G; Peptidyl-prolyl cis-trans isomerase [Source:UniProtKB/TrEMBL;Acc:A0A075B767]
TC1200012723.hg.1	2.93	0.0471	ZNF664	zinc finger protein 664
TC0600010383.hg.1	2.91	0.0376	HUS1B	HUS1 checkpoint clamp component B
TC0600013007.hg.1	2.83	0.0214	FAM184A	family with sequence similarity 184, member A
TC0500012272.hg.1	2.73	0.0383	SLC25A2	solute carrier family 25 (mitochondrial carrier; ornithine transporter) member 2
TC0100007512.hg.1	2.69	0.0037	GPR3	G protein-coupled receptor 3
TC0600012372.hg.1	2.28	0.0136	LCA5	Leber congenital amaurosis 5
TC0400010940.hg.1	2.22	0.0253	SULT1B1	sulfotransferase family 1B member 1
TSUnmapped00000614.hg.1	2.19	0.0116	ZNF660	zinc finger protein 660
TC1000006975.hg.1	2.17	0.0337	MLLT10	myeloid/lymphoid or mixed-lineage leukemia; translocated to, 10
TC0400008663.hg.1	2.16	0.0137	HSPA4L	heat shock 70kDa protein 4-like
TC1500006963.hg.1	2.15	0.0291	CASC5	cancer susceptibility candidate 5
TC1900011207.hg.1	2.11	0.0297	JOSD2	Josephin domain containing 2
TC0300011259.hg.1	2.08	0.0098	ARHGEF3	Rho guanine nucleotide exchange factor 3
TC1300009924.hg.1	2.06	0.015	GAS6	growth arrest-specific 6
TC0800010506.hg.1	2.06	0.022	PLAG1	pleiomorphic adenoma gene 1
TC1700011929.hg.1	1.96	0.0315	RBFOX3	RNA binding protein, fox-1 homolog (C. elegans) 3
TC1300009907.hg.1	1.92	0.0417	DCUN1D2	DCN1, defective in cullin neddylation 1, domain containing 2
TC1900012026.hg.1	1.91	0.0155	ZNF468	zinc finger protein 468

TC0100007481.hg.1	1.91	0.0346	PIGV	phosphatidylinositol glycan anchor biosynthesis class V
TSUnmapped00000384.hg.1	1.89	0.0081	ZNF546	zinc finger protein 546
TC0600008515.hg.1	1.88	0.0152	KCNQ5	Jeck2013 ALT_ACCEPTOR, ALT_DONOR, coding, INTERNAL, intronic best transcript NM_001160133
TC0700013611.hg.1	1.85	0.0204	TFEC	transcription factor EC
TC0600007087.hg.1	1.85	0.0217	CAP2	CAP, adenylate cyclase-associated protein, 2 (yeast)
TC1900011226.hg.1	1.85	0.0463	KLK4	kallikrein related peptidase 4
TC1200011327.hg.1	1.83	0.0138	CSRP2	cysteine and glycine-rich protein 2
TC0500012605.hg.1	1.82	0.0151	SOX30	SRY box 30
TC1900007458.hg.1	1.82	0.0227	ZNF93	zinc finger protein 93
TC0200007803.hg.1	1.81	0.0241	LGALSL	lectin, galactoside-binding-like
TC0900010796.hg.1	1.79	0.017	OMD	osteomodulin
TC1100007185.hg.1	1.78	0.0134	ELP4	elongator acetyltransferase complex subunit 4
TC1200010011.hg.1	1.77	0.0225	HIST4H4	histone cluster 4, H4
TC0100007396.hg.1	1.76	0.0116	RHD	Rh blood group, D antigen
TC1300010005.hg.1	1.76	0.0184	MCF2L	MCF.2 cell line derived transforming sequence like
TC0300006793.hg.1	1.75	0.0099	KAT2B	Transcript Identified by AceView, Entrez Gene ID(s) 8850
TC0600014140.hg.1	1.74	0.0056	PRIM2	Salzman2013 ANNOTATED, CDS, coding, OVCODE, OVEXON, UTR3 best transcript NM_000947
TC0X00008264.hg.1	1.73	0.021	AKAP14	A kinase (PRKA) anchor protein 14
TC1100011240.hg.1	1.73	0.0464	KCNK7	potassium channel, two pore domain subfamily K, member 7
TC0200013021.hg.1	1.72	0.0307	PAIP2B	poly(A) binding protein interacting protein 2B

TC0900007472.hg.1	1.71	0.0081	ANKRD20A1	ankyrin repeat domain 20 family, member A1
TC0200009167.hg.1	1.71	0.0159	CNTNAP5	contactin associated protein-like 5
TC2000007581.hg.1	1.7	0.0496	SULF2	Memczak2013 ANTISENSE, coding, INTERNAL, intronic best transcript NM_001161841
TC2100006442.hg.1	1.69	0.0191	SIK1	salt-inducible kinase 1
TC0900008156.hg.1	1.69	0.0304	NCBP1	nuclear cap binding protein subunit 1
TC0600013781.hg.1	1.68	0.0028	LPA	lipoprotein, Lp(a)
TC1200008477.hg.1	1.68	0.0108	CRADD	CASP2 and RIPK1 domain containing adaptor with death domain
TC0900011837.hg.1	1.66	0.0337	ABO	ABO blood group (transferase A, alpha 1-3-N-acetylgalactosaminyltransferase; transferase B, alpha 1-3-galactosyltransferase)
TC1700009372.hg.1	1.66	0.0348	CRK	v-crk avian sarcoma virus CT10 oncogene homolog
TC0500009109.hg.1	1.66	0.0416	GPX3	glutathione peroxidase 3
TC0200016505.hg.1	1.65	0.007	CCDC138	coiled-coil domain containing 138
TC0100007910.hg.1	1.65	0.0401	OXCT2P1	3-oxoacid CoA-transferase 2 pseudogene 1
TC1700006611.hg.1	1.65	0.0402	CYB5D2	cytochrome b5 domain containing 2
TC0700007294.hg.1	1.64	0.027	SUGCT	succinyl-CoA:glutarate-CoA transferase
TC2200008465.hg.1	1.63	0.0241	SELM	selenoprotein M; selenoprotein M [Source:EntrezGene;Acc:140606]; Salzman2013 ANNOTATED, CDS, coding, OVCODE, OVERLAPTX, OVEXON, UTR3 best transcript NM_080430; Salzman2013 ANNOTATED, CDS, coding, OVCODE, OVERLAPTX, OVEXON, UTR3, UTR5 best transcript NM_080430; Transcript Identified by AceView, Entrez Gene ID(s) 140606
TC1800006912.hg.1	1.62	0.0304	IMPACT	impact RWD domain protein
TC1400006823.hg.1	1.61	0.0266	SCFD1	sec1 family domain containing 1

TC1200012722.hg.1	1.61	0.0346	FAM101A	family with sequence similarity 101, member A
TSUnmapped00000352.hg.1	1.6	0.0412	HMBS	hydroxymethylbilane synthase
TC1500010923.hg.1	1.59	0.0085	DET1	de-etiolated homolog 1 (Arabidopsis)
TC0500013310.hg.1	1.59	0.0211	LOC102467081; CTD-2313F11.1; slygee	uncharacterized LOC102467081; novel transcript, antisense to GZMK; Transcript Identified by AceView
TC0X00007285.hg.1	1.59	0.0316	XAGE1B; XAGE1E	X antigen family, member 1B; X antigen family, member 1E
TC0100010648.hg.1	1.59	0.0327	PRDX6	peroxiredoxin 6
TC1800006905.hg.1	1.58	0.019	CABYR	calcium binding tyrosine-(Y)-phosphorylation regulated
TC2200009235.hg.1	1.58	0.0338	SNRPD3	small nuclear ribonucleoprotein D3 polypeptide
TC0700013488.hg.1	1.58	0.0068	REPIN1	replication initiator 1
TC0Y00006512.hg.1	1.58	0.0294	TSPY2	testis specific protein, Y-linked 2
TC1400010734.hg.1	1.57	0.0164	NKX2-1	NK2 homeobox 1
TC1600011476.hg.1	1.55	0.0062	ZNF500	zinc finger protein 500
TC0X00006723.hg.1	1.55	0.0098	CDKL5	cyclin-dependent kinase-like 5
TC0500010085.hg.1	1.54	0.0181	CMBL	carboxymethylenebutenolidase homolog (Pseudomonas)
TC0100011899.hg.1	1.53	0.0207	TMEM78	transmembrane protein 78
TC2000007973.hg.1	1.53	0.0219	CDH4	cadherin 4, type 1, R-cadherin (retinal)
TC0400013001.hg.1	1.53	0.0221	LRP2BP	LRP2 binding protein
TC0900012288.hg.1	1.53	0.0404	LCN10	lipocalin 10
TC2000007213.hg.1	1.53	0.0496	MMP24	matrix metallopeptidase 24 (membrane-inserted)
TSUnmapped00000532.hg.1	1.51	0.0191	LRIG1	
TC0900008545.hg.1	1.51	0.0318	PAPPA	pregnancy-associated plasma protein A, pappalysin 1

TC1900011334.hg.1	1.5	0.0111	ZNF677	zinc finger protein 677
TC1500007510.hg.1	1.49	0.0288	TPM1	tropomyosin 1 (alpha)
TC0400007988.hg.1	1.49	0.0297	THAP9	THAP domain containing 9
TC0700008397.hg.1	1.49	0.0456	DYNC1I1	dynein, cytoplasmic 1, intermediate chain 1
TC0X00008178.hg.1	1.48	0.0245	HTR2C	5-hydroxytryptamine (serotonin) receptor 2C, G protein-coupled
TC0700013324.hg.1	1.48	0.0302	GET4	golgi to ER traffic protein 4
TC1300008424.hg.1	1.46	0.01	USP12	ubiquitin specific peptidase 12
TC0500013230.hg.1	1.46	0.0278	PCBD2	pterin-4 alpha-carbinolamine dehydratase/dimerization cofactor of hepatocyte nuclear factor 1 alpha (TCF1) 2
TC0400011503.hg.1	1.46	0.028	PPA2	pyrophosphatase (inorganic) 2
TC1100006509.hg.1	1.46	0.0308	MUC5B	mucin 5B, oligomeric mucus/gel-forming
TC1600009656.hg.1	1.46	0.0494	PDILT	protein disulfide isomerase-like, testis expressed
TC0800006968.hg.1	1.45	0.0193	FGF17	fibroblast growth factor 17
TC1500010350.hg.1	1.45	0.0372	NTRK3	neurotrophic tyrosine kinase, receptor, type 3
TC1000009404.hg.1	1.45	0.0427	PWWP2B	PWWP domain containing 2B
TC0100006650.hg.1	1.44	0.028	AJAP1	adherens junctions associated protein 1
TC1900010050.hg.1	1.43	0.0439	SUGP1	SURP and G-patch domain containing 1
TC0700006656.hg.1	1.43	0.046	RSPH10B2	radial spoke head 10 homolog B2 (Chlamydomonas)
TC0X00010460.hg.1	1.42	0.0148	RIPPLY1	ripply transcriptional repressor 1
TC1900011690.hg.1	1.42	0.017	ZNF493	zinc finger protein 493
TC1000006633.hg.1	1.41	0.0388	FAM208B	family with sequence similarity 208, member B
TC0300010989.hg.1	1.41	0.0437	CSPG5	chondroitin sulfate proteoglycan 5 (neuroglycan C)

TC1500010739.hg.1	1.41	0.05	SLC12A1	solute carrier family 12 (sodium/potassium/chloride transporter), member 1
TC0500009039.hg.1	1.4	0.0148	FBXO38	F-box protein 38
TC0Y00007240.hg.1	-1.4	0.0139	CSPG4P1Y; DNM1P24	chondroitin sulfate proteoglycan 4 pseudogene 1, Y-linked; dynamin 1 pseudogene 24
TC0500013246.hg.1	-1.41	0.0267	PCDHB10	protocadherin beta 10
TSUnmapped00000794.hg.1	-1.41	0.0315	PRAMEF9	
TC0200016309.hg.1	-1.41	0.0464	OTOS	otospiralin
TC1200011495.hg.1	-1.41	0.0479	LUM	lumican
TC0900010355.hg.1	-1.42	0.0266	C9orf135-AS1	C9orf135 antisense RNA 1 (head to head)
TC1400008599.hg.1	-1.42	0.0464	OR6S1	olfactory receptor, family 6, subfamily S, member 1
TC0100008897.hg.1	-1.43	0.0199	WDR63	WD repeat domain 63
TC0100010011.hg.1	-1.43	0.0226	LCE6A	late cornified envelope 6A
TC0400011727.hg.1	-1.43	0.0377	PRDM5	PR domain containing 5
TC0100016919.hg.1	-1.44	0.0131	CSRP1; markobu	Memczak2013 ALT_ACCEPTOR, ALT_DONOR, coding, INTERNAL, intronic best transcript NM_001193571; Transcript Identified by AceView
TC1200012159.hg.1	-1.44	0.0194	MSI1	musashi RNA binding protein 1
TC1700008014.hg.1	-1.44	0.038	ADAM11	ADAM metallopeptidase domain 11
TC1800006447.hg.1	-1.44	0.0459	CLUL1	clusterin-like 1 (retinal)
TSUnmapped00000415.hg.1	-1.45	0.0388	INPP5D	inositol polyphosphate-5-phosphatase D
TSUnmapped00000292.hg.1	-1.45	0.0435	TGM4	transglutaminase 4
TC0200012382.hg.1	-1.45	0.0476	OXER1	oxoeicosanoid (OXE) receptor 1
TC0800009600.hg.1	-1.45	0.0478	FAM167A	family with sequence similarity 167, member A

TC0X00006489.hg.1	-1.45	0.0484	ARSF	arylsulfatase F
TC1200012578.hg.1	-1.46	0.0046	KCNA6; GALNT8	potassium channel, voltage gated shaker related subfamily A, member 6; polypeptide N-acetylgalactosaminyltransferase 8
TC1400010698.hg.1	-1.46	0.0046	INF2	inverted formin, FH2 and WH2 domain containing
TC0X00010051.hg.1	-1.46	0.0098	NAP1L6	nucleosome assembly protein 1-like 6
TC0400009080.hg.1	-1.46	0.0215	NPY2R	neuropeptide Y receptor Y2
TC0800008769.hg.1	-1.46	0.0231	TRMT12	tRNA methyltransferase 12 homolog (S. cerevisiae)
TC1000012511.hg.1	-1.46	0.0239	PRAP1	proline-rich acidic protein 1
TC0100007486.hg.1	-1.46	0.031	SFN	stratifin
TC1900010963.hg.1	-1.46	0.0489	NANOS2	nanos homolog 2 (Drosophila)
TC2200009107.hg.1	-1.47	0.0148	C22orf34	chromosome 22 open reading frame 34
TC1500008670.hg.1	-1.47	0.0439	POTEB	POTE ankyrin domain family, member B
TC0500012282.hg.1	-1.47	0.0483	HDAC3	histone deacetylase 3
TC0700009514.hg.1	-1.48	0.0057	OR2F1	olfactory receptor, family 2, subfamily F, member 1 (gene/pseudogene)
TC0200011372.hg.1	-1.48	0.0193	AQP12A	aquaporin 12A
TC1100008730.hg.1	-1.48	0.0221	TRIM49C	tripartite motif containing 49C
TC1500010591.hg.1	-1.48	0.0408	FAM169B	family with sequence similarity 169, member B
TC1500009238.hg.1	-1.48	0.0482	FRMD5	FERM domain containing 5
TC0900008665.hg.1	-1.49	0.0177	OR1J2	olfactory receptor, family 1, subfamily J, member 2
TC0100014790.hg.1	-1.49	0.0239	COL24A1	collagen, type XXIV, alpha 1
TC1100012314.hg.1	-1.49	0.0281	PIH1D2	PIH1 domain containing 2
TC0200016571.hg.1	-1.5	0.0102	OSBPL6	oxysterol binding protein-like 6
TC1100011300.hg.1	-1.5	0.0238	SPTBN2	spectrin, beta, non-erythrocytic 2
TSUnmapped00000105.hg.1	-1.5	0.0254	ZNF501	zinc finger protein 501 [Source:HGNC Symbol;Acc:HGNC:23717]

TC0700008961.hg.1	-1.5	0.029	ASB15	ankyrin repeat and SOCS box containing 15
TC1800007233.hg.1	-1.5	0.0329	KATNAL2	katanin p60 subunit A-like 2
TC0300008524.hg.1	-1.5	0.0343	STXBP5L	syntaxin binding protein 5-like
TC0600007305.hg.1	-1.5	0.0345	BTN2A3P	butyrophilin, subfamily 2, member A3, pseudogene
TC1900010031.hg.1	-1.5	0.0346	COMP	cartilage oligomeric matrix protein
TC0500009432.hg.1	-1.5	0.039	SMIM23	small integral membrane protein 23
TC0700009479.hg.1	-1.5	0.0487	OR6V1	olfactory receptor, family 6, subfamily V, member 1
TC0300008029.hg.1	-1.51	0.0095	EPHA3	EPH receptor A3
TC1900011679.hg.1	-1.51	0.0225	CYP4F3	cytochrome P450, family 4, subfamily F, polypeptide 3
TC0200015574.hg.1	-1.51	0.036	CRYGA	crystallin gamma A
TC1200007514.hg.1	-1.51	0.0467	H1FNT	H1 histone family, member N, testis-specific
TC1600011517.hg.1	-1.52	0.0138	DOC2A	double C2-like domains, alpha
TC1700012033.hg.1	-1.52	0.0187	TMEM105	transmembrane protein 105
TC0300013813.hg.1	-1.52	0.0365	ACKR2	atypical chemokine receptor 2
TC1100008994.hg.1	-1.53	0.0015	C11orf87	chromosome 11 open reading frame 87
TC1600007285.hg.1	-1.53	0.0024	HS3ST4	heparan sulfate (glucosamine) 3-O-sulfotransferase 4
TC1800007490.hg.1	-1.53	0.0078	CDH20	cadherin 20, type 2
TC0900010333.hg.1	-1.53	0.0104	PRKACG	protein kinase, cAMP-dependent, catalytic, gamma
TC0500012166.hg.1	-1.53	0.0457	CDC25C	cell division cycle 25C
TC2200006552.hg.1	-1.54	0.0124	TMEM191B	transmembrane protein 191B
TC0300007733.hg.1	-1.54	0.018	FAM19A1	family with sequence similarity 19 (chemokine (C-C motif)-like), member A1
TC0100009408.hg.1	-1.54	0.0201	PIFO	primary cilia formation
TC0200016553.hg.1	-1.54	0.0218	DAPL1; OR7E89P	death associated protein like 1; olfactory receptor, family 7, subfamily E, member 89 pseudogene

TSUnmapped00000151.hg.1	-1.54	0.0225	ADAMTS13	ADAM metallopeptidase with thrombospondin type 1 motif 13
TC1900010370.hg.1	-1.54	0.0239	SLC7A9	solute carrier family 7 (amino acid transporter light chain, bo,+ system), member 9
TC0X00010893.hg.1	-1.54	0.0293	CT55	cancer/testis antigen 55
TC0300014077.hg.1	-1.54	0.0391	WDR49	WD repeat domain 49
TC0700012870.hg.1	-1.54	0.0484	KEL	Kell blood group, metallo-endopeptidase
TC1600007723.hg.1	-1.55	0.0016	GPT2	glutamic pyruvate transaminase (alanine aminotransferase) 2
TC2000008807.hg.1	-1.55	0.0042	DEFB119	defensin, beta 119
TC1000012198.hg.1	-1.55	0.0273	C10orf90	chromosome 10 open reading frame 90
TC1200007882.hg.1	-1.55	0.0376	GLI1	GLI family zinc finger 1
TC0300013080.hg.1	-1.55	0.0428	ACTRT3	actin-related protein T3
TC2200007101.hg.1	-1.56	0.0161	LINC01521	long intergenic non-protein coding RNA 1521
TC0800011462.hg.1	-1.56	0.0189	TMEM74	transmembrane protein 74
TC0X00009101.hg.1	-1.56	0.0234	GPM6B	glycoprotein M6B
TC1500010715.hg.1	-1.56	0.0318	BUB1B	BUB1 mitotic checkpoint serine/threonine kinase B
TC1000012481.hg.1	-1.56	0.0352	CYP2C19	cytochrome P450, family 2, subfamily C, polypeptide 19
TC0500010615.hg.1	-1.56	0.0422	SEPP1	selenoprotein P, plasma, 1
TC1100007783.hg.1	-1.57	0.0068	ZP1	zona pellucida glycoprotein 1 (sperm receptor)
TC1900011201.hg.1	-1.57	0.0094	FAM71E1	family with sequence similarity 71, member E1
TC0900009922.hg.1	-1.57	0.017	FAM205C	family with sequence similarity 205, member C
TC0X00011291.hg.1	-1.57	0.0361	PAGE2	P antigen family, member 2 (prostate associated)
TC0900010147.hg.1	-1.57	0.0372	CNTNAP3B	contactin associated protein-like 3B

TSUnmapped00000040.hg.1	-1.57	0.0425	ZNF660	zinc finger protein 660
TC0200015645.hg.1	-1.58	0.0011	ABCA12	ATP binding cassette subfamily A member 12
TC0600007985.hg.1	-1.58	0.045	TSPO2	translocator protein 2
TC1100010015.hg.1	-1.59	0.0125	NLRP10	NLR family, pyrin domain containing 10
TC0100016902.hg.1	-1.59	0.0342	ASCL5	achaete-scute family bHLH transcription factor 5
TC0X00006709.hg.1	-1.59	0.0354	NHS	Nance-Horan syndrome (congenital cataracts and dental anomalies)
TC0X00010445.hg.1	-1.6	0.0011	ESX1	ESX homeobox 1
TC1700012410.hg.1	-1.6	0.0125	KRTAP4-3	keratin associated protein 4-3
TC0600007446.hg.1	-1.6	0.0419	OR2J1	olfactory receptor, family 2, subfamily J, member 1 (gene/pseudogene)
TC1400009919.hg.1	-1.6	0.0441	KCNK10	potassium channel, two pore domain subfamily K, member 10
TC1100009175.hg.1	-1.6	0.0449	CEP164	centrosomal protein 164kDa
TC0300011941.hg.1	-1.61	0.0085	MYH15	myosin, heavy chain 15
TC0300014040.hg.1	-1.61	0.0103	TXNRD3NB; TXNRD3	thioredoxin reductase 3 neighbor; thioredoxin reductase 3
TC0400008083.hg.1	-1.61	0.0197	DSPP	dentin sialophosphoprotein
TC0200015023.hg.1	-1.61	0.0201	EVX2	even-skipped homeobox 2
TC0100015872.hg.1	-1.61	0.0231	S100A14	S100 calcium binding protein A14
TC0300011550.hg.1	-1.61	0.0303	PDZRN3	PDZ domain containing ring finger 3
TC1900009368.hg.1	-1.62	0.0142	TICAM1	toll-like receptor adaptor molecule 1
TC1600009766.hg.1	-1.62	0.0148	LINC01567	long intergenic non-protein coding RNA 1567
TC0400011159.hg.1	-1.62	0.0172	PRKG2	protein kinase, cGMP-dependent, type II
TC1400006686.hg.1	-1.62	0.024	CMTM5	CKLF-like MARVEL transmembrane domain containing 5

TC0700012871.hg.1	-1.62	0.0377	OR9A2	olfactory receptor, family 9, subfamily A, member 2
TC1200012833.hg.1	-1.62	0.0464	PAH	phenylalanine hydroxylase
TC1900009791.hg.1	-1.63	0.0307	C19orf57	chromosome 19 open reading frame 57
TC1600008219.hg.1	-1.63	0.034	CHTF8	Jeck2013 ANTISENSE, coding, INTERNAL, OVEXON, UTR3 best transcript NM_001039690
TC2100007872.hg.1	-1.63	0.0451	GRIK1	glutamate receptor, ionotropic, kainate 1
TC0100016276.hg.1	-1.64	0.0165	FAM78B	family with sequence similarity 78, member B
TC1200012768.hg.1	-1.64	0.0465	RERG	RAS-like, estrogen-regulated, growth inhibitor
TC0100010651.hg.1	-1.65	0.006	LOC730159; RP11-360D2.1	uncharacterized LOC730159; putative novel transcript
TC0500013215.hg.1	-1.65	0.0256	LVRN	laeverin
TC0X00008688.hg.1	-1.65	0.0285	MAGEA9B; MAGEA9	MAGE family member A9B; MAGE family member A9
TC0700007975.hg.1	-1.65	0.0318	FKBP6	FK506 binding protein 6
TC1300008552.hg.1	-1.65	0.0399	ZAR1L	zygote arrest 1-like
TC0400009695.hg.1	-1.65	0.045	MFSD7	major facilitator superfamily domain containing 7
TC0100010006.hg.1	-1.66	0.0016	KPRP	keratinocyte proline-rich protein
TC0300011951.hg.1	-1.66	0.0178	MORC1	MORC family CW-type zinc finger 1
TC0900008679.hg.1	-1.66	0.0198	OR1K1	olfactory receptor, family 1, subfamily K, member 1
TC0200008481.hg.1	-1.66	0.0324	TRIM43	tripartite motif containing 43
TC1100013157.hg.1	-1.66	0.0487	DCDC1	doublecortin domain containing 1
TC0300013606.hg.1	-1.67	0.0189	TMEM44	transmembrane protein 44
TSUnmapped00000181.hg.1	-1.67	0.0397	HMBS	hydroxymethylbilane synthase
TC1700011720.hg.1	-1.67	0.0448	FDXR	ferredoxin reductase

TC1000010070.hg.1	-1.67	0.047	PRTFDC1	phosphoribosyl transferase domain containing 1
TC1100007601.hg.1	-1.68	0.0066	OR4S2	olfactory receptor, family 4, subfamily S, member 2
TC1200012577.hg.1	-1.68	0.0141	GALNT8	polypeptide N-acetylgalactosaminyltransferase 8
TC0900006655.hg.1	-1.68	0.0151	LURAP1L	leucine rich adaptor protein 1-like
TSUnmapped00000648.hg.1	-1.68	0.025	SAG	S-antigen; retina and pineal gland (arrestin)
TC0700009300.hg.1	-1.68	0.0293	LOC100130880; AC022173.2	uncharacterized LOC100130880; novel transcript
TC0100011153.hg.1	-1.69	0.0201	C1orf106	chromosome 1 open reading frame 106
TC0300009139.hg.1	-1.69	0.0214	TM4SF4	transmembrane 4 L six family member 4
TC0100010867.hg.1	-1.69	0.048	LAMC2	laminin, gamma 2
TC0400012836.hg.1	-1.7	0.0044	ENPEP	glutamyl aminopeptidase (aminopeptidase A)
TC1500009795.hg.1	-1.7	0.006	IGDCC3	immunoglobulin superfamily, DCC subclass, member 3
TC0100015594.hg.1	-1.7	0.0141	ITGA10	integrin alpha 10
TC1500007143.hg.1	-1.7	0.0184	SEMA6D	sema domain, transmembrane domain (TM), and cytoplasmic domain, (semaphorin) 6D
TC1100013031.hg.1	-1.7	0.0411	NUDT22	nudix hydrolase 22
TC1400008637.hg.1	-1.7	0.0484	SALL2	spalt-like transcription factor 2
TC0100015109.hg.1	-1.7	0.0499	LOC101928436; RP11-153F1.1; RP11-347K2.2; teyfawbu	uncharacterized LOC101928436; novel transcript; Transcript Identified by AceView
TC1500009760.hg.1	-1.71	0.009	PIF1	PIF1 5-to-3 DNA helicase
TC0300007014.hg.1	-1.71	0.0256	ARPP21	Transcript Identified by AceView, Entrez Gene ID(s) 10777
TC1600008008.hg.1	-1.72	0.0127	DRC7	dynein regulatory complex subunit 7

TSUnmapped00000052.hg.1	-1.72	0.0209	HHAT	hedgehog acyltransferase
TC0400009037.hg.1	-1.72	0.0236	MND1	meiotic nuclear divisions 1
TC2000007448.hg.1	-1.74	0.0191	GDAP1L1	ganglioside induced differentiation associated protein 1-like 1
TC0600012540.hg.1	-1.74	0.0456	GABRR2	gamma-aminobutyric acid (GABA) A receptor, rho 2
TC0800012166.hg.1	-1.75	0.0028	SCRIB	Zhang2013 ALT_ACCEPTOR, ALT_DONOR, coding, INTERNAL, intronic best transcript NM_182706
TC0200006831.hg.1	-1.75	0.0072	VSNL1	visinin like 1
TC2100007239.hg.1	-1.75	0.0115	UMODL1	uromodulin-like 1
TC1100010998.hg.1	-1.75	0.0247	MS4A4E	membrane-spanning 4-domains, subfamily A, member 4E
TC0X00009044.hg.1	-1.75	0.0269	GPR143	G protein-coupled receptor 143
TC1700010802.hg.1	-1.75	0.0304	MPP2	membrane protein, palmitoylated 2
TC0X00007516.hg.1	-1.76	0.0089	LINC00269	long intergenic non-protein coding RNA 269
TC0200013668.hg.1	-1.76	0.0121	CREG2	cellular repressor of E1A-stimulated genes 2
TC0900011977.hg.1	-1.76	0.0273	SNAPC4	Zhang2013 ALT_ACCEPTOR, ALT_DONOR, coding, INTERNAL, intronic best transcript NM_003086
TC0100016822.hg.1	-1.76	0.0319	KCNT2	potassium channel, sodium activated subfamily T, member 2
TC0200011129.hg.1	-1.77	0.0237	SAG	S-antigen; retina and pineal gland (arrestin)
TC1100011131.hg.1	-1.77	0.0491	C11orf95	chromosome 11 open reading frame 95
TC2000008802.hg.1	-1.78	0.0193	DEFB116	defensin, beta 116
TC0700012228.hg.1	-1.78	0.0433	LAMB4	laminin, beta 4
TC0200014361.hg.1	-1.79	0.0094	LYPD1	LY6/PLAUR domain containing 1
TC2200008528.hg.1	-1.79	0.0097	LARGE	like-glycosyltransferase
TC0400007055.hg.1	-1.79	0.0329	CDC42	Transcript Identified by AceView, Entrez Gene ID(s) 998

TC1500007839.hg.1	-1.8	0.0042	ISLR	immunoglobulin superfamily containing leucine-rich repeat
TC0500009038.hg.1	-1.8	0.0193	SPINK9	serine peptidase inhibitor, Kazal type 9
TC0400008086.hg.1	-1.8	0.024	IBSP	integrin-binding sialoprotein
TC1400010651.hg.1	-1.8	0.0301	DLK1	delta-like 1 homolog (Drosophila)
TC0100011669.hg.1	-1.8	0.0466	HLX	H2.0-like homeobox
TC0X00010468.hg.1	-1.81	0.0133	FRMPD3-AS1	FRMPD3 antisense RNA 1
TC1100010857.hg.1	-1.81	0.0188	OR5M3	olfactory receptor, family 5, subfamily M, member 3
TSUnmapped00000366.hg.1	-1.82	0.0129	BCL2L14	BCL2-like 14 (apoptosis facilitator)
TC1700007108.hg.1	-1.82	0.0266	MIEF2	mitochondrial elongation factor 2
TC0200011257.hg.1	-1.83	0.0086	PRLH	prolactin releasing hormone
TC0800009637.hg.1	-1.83	0.0212	USP17L2	ubiquitin specific peptidase 17-like family member 2
TC0300010228.hg.1	-1.83	0.0218	FANCD2OS	FANCD2 opposite strand
TC0100016339.hg.1	-1.84	0.0131	DPT	dermatopontin
TC0400011535.hg.1	-1.84	0.0234	DKK2	dickkopf WNT signaling pathway inhibitor 2
TC1200010865.hg.1	-1.84	0.0458	ITGA7	integrin alpha 7
TC1200008731.hg.1	-1.86	0.0044	RFX4	regulatory factor X, 4 (influences HLA class II expression)
TSUnmapped00000622.hg.1	-1.86	0.0342	GREM1	gremlin 1, DAN family BMP antagonist [Source:HGNC Symbol;Acc:HGNC:2001]
TC1100013045.hg.1	-1.87	0.0384	SIPA1	signal-induced proliferation-associated 1
TC0100012942.hg.1	-1.88	0.0253	PRAMEF18	PRAME family member 18
TC1500008252.hg.1	-1.88	0.0358	FANCI	Fanconi anemia complementation group I
TC1900008303.hg.1	-1.88	0.045	CLASRP	CLK4-associating serine/arginine rich protein
TC2000007936.hg.1	-1.9	0.0098	PHACTR3	phosphatase and actin regulator 3
TC0600011308.hg.1	-1.9	0.034	MAS1L	MAS1 proto-oncogene like, G protein-coupled receptor

TC1100007770.hg.1	-1.92	0.003	MS4A5	membrane-spanning 4-domains, subfamily A, member 5
TC1000011599.hg.1	-1.92	0.0301	CPN1	carboxypeptidase N, polypeptide 1
TC0X00009677.hg.1	-1.94	0.0264	AKAP4	A kinase (PRKA) anchor protein 4
TC0100015853.hg.1	-1.94	0.0353	SPRR2G	small proline-rich protein 2G
TC1300009725.hg.1	-1.95	0.0175	FAM155A	family with sequence similarity 155, member A
TC1100010971.hg.1	-1.97	0.0491	OR5A2	olfactory receptor, family 5, subfamily A, member 2
TC0500013239.hg.1	-1.98	0.0179	PCDHB3	protocadherin beta 3
TC2100006883.hg.1	-1.99	0.0005	KRTAP20-3	keratin associated protein 20-3
TC0100015401.hg.1	-1.99	0.0027	IGSF3	immunoglobulin superfamily, member 3
TC0600011401.hg.1	-1.99	0.021	C6orf15	chromosome 6 open reading frame 15
TC1000011973.hg.1	-2.02	0.0063	PRLHR	prolactin releasing hormone receptor
TC0400006783.hg.1	-2.03	0.0056	USP17L10	ubiquitin specific peptidase 17-like family member 10
TC0600011605.hg.1	-2.04	0.0085	TCP11	t-complex 11, testis-specific
TC0X00010990.hg.1	-2.06	0.0098	SPANXC; SPANXD	SPANX family, member C; SPANX family, member D
TC1600006666.hg.1	-2.08	0.0067	CASP16P	caspase 16, pseudogene
TC0100013992.hg.1	-2.09	0.0000516	TCTEX1D4	Tctex1 domain containing 4
TC1700010640.hg.1	-2.09	0.0026	KRT20	keratin 20, type I
TC0800008782.hg.1	-2.09	0.0143	ZNF572	zinc finger protein 572
TC1100010850.hg.1	-2.1	0.001	OR5T2	olfactory receptor, family 5, subfamily T, member 2
TC1900009492.hg.1	-2.11	0.0065	CLEC4G	C-type lectin domain family 4, member G
TC1900012010.hg.1	-2.14	0.0115	SHANK1	SH3 and multiple ankyrin repeat domains 1
TC0300007499.hg.1	-2.15	0.0258	ITIH1	inter-alpha-trypsin inhibitor heavy chain 1

TC0900008842.hg.1	-2.17	0.0055	SLC25A25	Zhang2013 ALT_ACCEPTOR, ALT_DONOR, coding, INTERNAL, intronic best transcript NM_001006641
TC1000012108.hg.1	-2.2	0.0013	CPXM2	carboxypeptidase X (M14 family), member 2
TC0600011131.hg.1	-2.21	0.0137	HIST1H1T	histone cluster 1, H1t
TC1400010603.hg.1	-2.24	0.0062	TTC6	tetratricopeptide repeat domain 6
TC1200006469.hg.1	-2.28	0.0135	WNT5B	wingless-type MMTV integration site family, member 5B
TC1500007753.hg.1	-2.3	0.0027	THSD4	Transcript Identified by AceView, Entrez Gene ID(s) 79875
TC0100014958.hg.1	-2.3	0.0482	BCAR3	breast cancer anti-estrogen resistance 3
TC0900007654.hg.1	-2.36	0.0013	GNAQ; skeyplorbu	Memczak2013 ANTISENSE, coding, INTERNAL, intronic best transcript NM_002072; Transcript Identified by AceView
TC0800006488.hg.1	-2.4	0.0036	ARHGEF10	Jeck2013 ALT_ACCEPTOR, ALT_DONOR, coding, INTERNAL, intronic best transcript NM_014629
TC0100008588.hg.1	-2.48	0.0325	EFCAB7; DLEU2L	EF-hand calcium binding domain 7; deleted in lymphocytic leukemia 2-like
TC0300013350.hg.1	-2.5	0.0078	YEATS2	Memczak2013 ANTISENSE, coding, INTERNAL, intronic best transcript NM_018023
TC1600010004.hg.1	-2.76	0.0273	ZNF646	Memczak2013 ANTISENSE, coding, INTERNAL, intronic best transcript NM_014699
TC1200010133.hg.1	-2.92	0.0484	SOX5	Transcript Identified by AceView, Entrez Gene ID(s) 6660
TC0700007031.hg.1	-3.32	0.0068	JAZF1	Memczak2013 ANTISENSE, coding, INTERNAL, intronic best transcript NM_175061

(G) CXCL8 and IL-15 treated T cells

ID	FC CXCL8 IL15 vs CONTROL	P-val	Gene Symbol	Description
TC0300011124.hg.1	34.87	0.0000000111	CISH	cytokine inducible SH2-containing protein
TC1000009691.hg.1	10.21	0.000000000443	IL2RA	interleukin 2 receptor, alpha
TC1400010195.hg.1	9.98	0.0145	WARS	tryptophanyl-tRNA synthetase
TC1200008474.hg.1	8.69	0.000000692	SOCS2	suppressor of cytokine signaling 2
TC0800007382.hg.1	8.41	0.0343	IDO1	indoleamine 2,3-dioxygenase 1
TC0400011052.hg.1	6.49	0.000094	CXCL9	chemokine (C-X-C motif) ligand 9
TC0400012956.hg.1	6.46	0.0201	ELOVL6	ELOVL fatty acid elongase 6
TC0100012734.hg.1	6.4	0.0428	TNFRSF9	tumor necrosis factor receptor superfamily, member 9
TC0600007596.hg.1	5.98	0.0006	TNF	tumor necrosis factor
TC1700010461.hg.1	5.59	0.024	CCL3L3; CCL3L1	chemokine (C-C motif) ligand 3-like 3; chemokine (C-C motif) ligand 3-like 1
TC0100014857.hg.1	5.34	0.0005	GBP5	guanylate binding protein 5
TC0600006442.hg.1	5.29	0.00000459	IRF4	interferon regulatory factor 4
TC1200007461.hg.1	5.26	0.0143	PCED1B	PC-esterase domain containing 1B
TC1800008891.hg.1	5.11	0.0015	BCL2	B-cell CLL/lymphoma 2
TC0600012036.hg.1	4.87	0.0075	IL17F	interleukin 17F
TC1200011177.hg.1	4.72	0.0482	IFNG	interferon, gamma
TC0X00009667.hg.1	4.7	0.0002	FOXP3	forkhead box P3
TC0100011012.hg.1	4.55	0.0019	RGS1	regulator of G-protein signaling 1
TC1900010807.hg.1	4.19	0.00000645	CEACAM1	carcinoembryonic antigen-related cell adhesion molecule 1 (biliary glycoprotein)

TC0600007862.hg.1	4.02	0.0007	PIM1	Pim-1 proto-oncogene, serine/threonine kinase
TC0100014852.hg.1	3.99	0.0000322	GBP1	guanylate binding protein 1, interferon-inducible
TC0300013555.hg.1	3.85	0.0088	MB21D2	Mab-21 domain containing 2
TC1200007822.hg.1	3.81	0.0051	IKZF4	IKAROS family zinc finger 4
TC1000011368.hg.1	3.63	0.0421	ANKRD22	ankyrin repeat domain 22
TC1900011004.hg.1	3.54	0.014	SLC1A5	solute carrier family 1 (neutral amino acid transporter), member 5
TC1200011179.hg.1	3.49	0.0342	IL22	interleukin 22
TC1000008891.hg.1	3.39	0.003	DUSP5	dual specificity phosphatase 5
TC1400007165.hg.1	3.36	0.000000545	PTGER2	prostaglandin E receptor 2
TC0600013007.hg.1	3.21	0.0199	FAM184A	family with sequence similarity 184, member A
TC0300008385.hg.1	3.18	0.0258	TIGIT	T-cell immunoreceptor with Ig and ITIM domains
TC2000008815.hg.1	2.98	0.0028	BCL2L1	BCL2-like 1
TC0100014855.hg.1	2.87	0.0032	GBP4	guanylate binding protein 4
TC0300007096.hg.1	2.84	0.0187	CCR8	chemokine (C-C motif) receptor 8
TC2200007242.hg.1	2.79	0.0033	APOL1	apolipoprotein L1
TC0600007595.hg.1	2.73	0.0163	LTA	lymphotoxin alpha
TC0600011674.hg.1	2.71	0.0327	PPIL1	peptidylprolyl isomerase (cyclophilin)-like 1
TC0900006559.hg.1	2.69	0.0043	CD274	CD274 molecule
TC0800011334.hg.1	2.67	0.0465	KLF10	Kruppel-like factor 10
TC0500012017.hg.1	2.65	0.0069	IRF1	interferon regulatory factor 1
TC1600007557.hg.1	2.62	0.0005	ZNF267	zinc finger protein 267
TC1900011779.hg.1	2.58	0.0002	FLT3LG	fms-related tyrosine kinase 3 ligand
TC1100012981.hg.1	2.56	0.0004	RCN1	reticulocalbin 1, EF-hand calcium binding domain

TC1200008906.hg.1	2.55	0.0035	TRAFD1	TRAF-type zinc finger domain containing 1
TC0100007481.hg.1	2.54	0.0025	PIGV	phosphatidylinositol glycan anchor biosynthesis class V
TC0200008666.hg.1	2.54	0.0229	IL1R1	interleukin 1 receptor, type I
TC0600008247.hg.1	2.51	0.0103	IL17A	interleukin 17A
TC1500010368.hg.1	2.48	0.0483	HAPLN3	hyaluronan and proteoglycan link protein 3
TC0100008975.hg.1	2.46	0.0375	GBP6	guanylate binding protein family, member 6
TC0700011785.hg.1	2.45	0.008	CDK6	cyclin-dependent kinase 6
TC1900011741.hg.1	2.41	0.0008	CEACAM5	carcinoembryonic antigen-related cell adhesion molecule 5
TC1200006555.hg.1	2.38	0.0481	CCND2	cyclin D2
TC0700010941.hg.1	2.37	0.0469	MYO1G	myosin IG
TC0X00011159.hg.1	2.34	0.0003	HAUS7; TREX2	HAUS augmin like complex subunit 7; three prime repair exonuclease 2
TC0600014255.hg.1	2.33	0.0033	UBD	ubiquitin D
TC2200008370.hg.1	2.31	0.0134	XBP1	X-box binding protein 1
TC0300006437.hg.1	2.31	0.0345	CHL1	cell adhesion molecule L1-like
TC0600010688.hg.1	2.3	0.0136	LYRM4	LYR motif containing 4
TC0200015242.hg.1	2.28	0.0008	STAT1	signal transducer and activator of transcription 1
TC1200009912.hg.1	2.28	0.0088	STYK1	serine/threonine/tyrosine kinase 1
TC1300009674.hg.1	2.27	0.0203	KDELC1	KDEL (Lys-Asp-Glu-Leu) containing 1
TC0700009232.hg.1	2.26	0.0013	BPGM	2,3-bisphosphoglycerate mutase
TC1600006658.hg.1	2.26	0.0178	IL32	interleukin 32
TC1600009395.hg.1	2.25	0.0025	SOCS1	suppressor of cytokine signaling 1
TC0600008094.hg.1	2.25	0.0143	TJAP1	Zhang2013 ALT_ACCEPTOR, ALT_DONOR, coding, INTERNAL, intronic best transcript NM_001146018

TC1400010721.hg.1	2.23	0.0361	PSME2; MIR7703	proteasome activator subunit 2; microRNA 7703
TC0600014074.hg.1	2.22	0.0044	HIVEP1	human immunodeficiency virus type I enhancer binding protein 1
TC0700007477.hg.1	2.22	0.0291	UPP1	uridine phosphorylase 1
TC0300012880.hg.1	2.21	0.0045	C3orf33	chromosome 3 open reading frame 33
TC0200009091.hg.1	2.21	0.0329	RALB	v-ral simian leukemia viral oncogene homolog B
TC0800006980.hg.1	2.18	0.0015	SLC39A14	solute carrier family 39 (zinc transporter), member 14
TC0300011259.hg.1	2.13	0.0423	ARHGEF3	Rho guanine nucleotide exchange factor 3
TC0100017834.hg.1	2.12	0.0026	GNG4	guanine nucleotide binding protein (G protein), gamma 4
TC0300006483.hg.1	2.12	0.0031	BHLHE40	basic helix-loop-helix family, member e40
TC1400006548.hg.1	2.12	0.0161	RNASE7	ribonuclease, RNase A family, 7
TC0200013913.hg.1	2.12	0.0451	IL1A	interleukin 1 alpha
TC0100009524.hg.1	2.11	0.0488	VANGL1	VANGL planar cell polarity protein 1
TC0700006984.hg.1	2.1	0.0341	SNX10	sorting nexin 10
TC1400008758.hg.1	2.08	0.048	CBLN3	cerebellin 3 precursor
TSUnmapped00000076.hg.1	2.06	0.0294	SURF4	surfeit 4
TC1200008541.hg.1	2.05	0.0243	CFAP54	cilia and flagella associated 54
TC0100014769.hg.1	2.04	0.0076	MCOLN3	mucolipin 3
TC0400007592.hg.1	2.03	0.003	PAICS	phosphoribosylaminoimidazole carboxylase, phosphoribosylaminoimidazole succinocarboxamide synthetase
TC1400009421.hg.1	2.03	0.035	GPX2	glutathione peroxidase 2
TC0500011702.hg.1	2.02	0.0336	STARD4	StAR-related lipid transfer domain containing 4

TC0100010648.hg.1	2	0.002	PRDX6	peroxiredoxin 6
TC0900011302.hg.1	1.99	0.0000748	TNFSF8	tumor necrosis factor (ligand) superfamily, member 8
TC0X00011393.hg.1	1.98	0.006	CT47A5; CT47A2; CT47A1; CT47A11; CT47A12	cancer/testis antigen family 47, member A5; cancer/testis antigen family 47, member A2; cancer/testis antigen family 47, member A1; cancer/testis antigen family 47, member A11; cancer/testis antigen family 47, member A12
TC1200012516.hg.1	1.98	0.024	GALNT9	polypeptide N-acetylgalactosaminyltransferase 9
TC0200015332.hg.1	1.97	0.0488	PGAP1	post-GPI attachment to proteins 1
TC1100013021.hg.1	1.94	0.014	FEN1	flap structure-specific endonuclease 1
TC0200016584.hg.1	1.94	0.0169	HSPE1	heat shock 10kDa protein 1
TC1600010732.hg.1	1.94	0.0399	NQO1	NAD(P)H dehydrogenase, quinone 1
TC0100016476.hg.1	1.93	0.005	KIAA0040	KIAA0040
TC0400012458.hg.1	1.93	0.0075	HPGD	hydroxyprostaglandin dehydrogenase 15-(NAD)
TC0900011177.hg.1	1.91	0.0086	TXN	thioredoxin
TSUnmapped00000327.hg.1	1.9	0.0157	HYOU1	hypoxia up-regulated 1
TC0600011945.hg.1	1.89	0.0026	RCAN2	regulator of calcineurin 2
TC1400007440.hg.1	1.89	0.0067	MTHFD1	methylenetetrahydrofolate dehydrogenase (NADP+ dependent) 1, methenyltetrahydrofolate cyclohydrolase, formyltetrahydrofolate synthetase
TSUnmapped00000244.hg.1	1.89	0.0356	HYOU1	hypoxia up-regulated 1
TSUnmapped00000322.hg.1	1.89	0.0447	HYOU1	hypoxia up-regulated 1
TC0200016702.hg.1	1.89	0.0469	POLR1A	polymerase (RNA) I polypeptide A

TC0600008539.hg.1	1.88	0.0088	CD109	CD109 molecule
TC0200016099.hg.1	1.88	0.0147	HJURP	Holliday junction recognition protein
TC0900008207.hg.1	1.87	0.0061	SEC61B	Sec61 translocon beta subunit
TC0800008032.hg.1	1.87	0.008	ZC2HC1A	zinc finger, C2HC-type containing 1A
TC0900008314.hg.1	1.87	0.0438	FSD1L	fibronectin type III and SPRY domain containing 1-like
TC0500011095.hg.1	1.87	0.048	MRPS27	mitochondrial ribosomal protein S27
TC0600007769.hg.1	1.86	0.0187	SNRPC	small nuclear ribonucleoprotein polypeptide C
TC1800007905.hg.1	1.86	0.0255	LPIN2	lipin 2
TC0600014275.hg.1	1.86	0.0352	TAP2	transporter 2, ATP-binding cassette, sub-family B (MDR/TAP)
TC1400007300.hg.1	1.86	0.044	PSMA3	proteasome subunit alpha 3
TC1200008642.hg.1	1.85	0.0002	PARPBP	PARP1 binding protein
TC1100012510.hg.1	1.85	0.0332	HYOU1	hypoxia up-regulated 1
TC0200010696.hg.1	1.85	0.0335	SPAG16	sperm associated antigen 16
TC1100012219.hg.1	1.84	0.0036	SLC35F2	solute carrier family 35, member F2
TC0900008337.hg.1	1.82	0.0052	ZNF462	zinc finger protein 462
TC0300012812.hg.1	1.82	0.0074	GPR171	G protein-coupled receptor 171
TC0800011472.hg.1	1.82	0.0394	NUDCD1	NudC domain containing 1
TC2200007780.hg.1	1.81	0.0043	CRELD2	cysteine rich with EGF-like domains 2
TC1700007099.hg.1	1.81	0.0051	DRG2	developmentally regulated GTP binding protein 2
TC0600014314.hg.1	1.81	0.0074	KHDC1	KH homology domain containing 1
TC0500006730.hg.1	1.81	0.0244	CCT5	chaperonin containing TCP1, subunit 5 (epsilon)
TSUnmapped00000558.hg.1	1.8	0.0065	HYOU1	hypoxia up-regulated 1
TC0200008679.hg.1	1.8	0.0194	SLC9A4	solute carrier family 9, subfamily A (NHE4, cation proton antiporter 4), member 4

TC1300007546.hg.1	1.79	0.0199	IRG1	immunoresponsive 1 homolog (mouse)
TC0100014014.hg.1	1.79	0.038	PRDX1	peroxiredoxin 1
TC1900008013.hg.1	1.78	0.0359	PSMD8	proteasome 26S subunit, non-ATPase 8
TC1700010714.hg.1	1.77	0.028	KCNH4	potassium channel, voltage gated eag related subfamily H, member 4
TC0500013242.hg.1	1.77	0.0326	PCDHB6	protocadherin beta 6
TC1500009148.hg.1	1.76	0.02	OIP5	Opa interacting protein 5
TC0800011129.hg.1	1.75	0.0029	CDH17	cadherin 17, LI cadherin (liver-intestine)
TC0100012778.hg.1	1.75	0.0036	ENO1	enolase 1, (alpha)
TC1100010984.hg.1	1.75	0.0133	OR10V1	olfactory receptor, family 10, subfamily V, member 1
TC1900008968.hg.1	1.75	0.0393	ZNF71	zinc finger protein 71
TSUnmapped00000383.hg.1	1.74	0.0233	HYOU1	hypoxia up-regulated 1
TC2100008559.hg.1	1.74	0.0432	C21orf59	chromosome 21 open reading frame 59
TC1300010005.hg.1	1.74	0.0496	MCF2L	MCF.2 cell line derived transforming sequence like
TC1400006718.hg.1	1.73	0.0272	PSME1	proteasome activator subunit 1
TC1100011052.hg.1	1.73	0.0353	FADS3	fatty acid desaturase 3
TC0200009813.hg.1	1.72	0.0074	PSMD14	proteasome 26S subunit, non-ATPase 14
TC0200013101.hg.1	1.72	0.0284	C2orf81	chromosome 2 open reading frame 81
TC1300009088.hg.1	1.72	0.0398	DIAPH3	diaphanous-related formin 3
TC0400011994.hg.1	1.72	0.0423	INPP4B	inositol polyphosphate-4-phosphatase type II B
TSUnmapped00000484.hg.1	1.71	0.0093	HYOU1	hypoxia up-regulated 1
TC1000006633.hg.1	1.71	0.0127	FAM208B	family with sequence similarity 208, member B
TC2200007214.hg.1	1.71	0.0304	APOL6	apolipoprotein L, 6

TC0X00008759.hg.1	1.71	0.0476	NSDHL	NAD(P) dependent steroid dehydrogenase-like
TC0600009439.hg.1	1.7	0.0032	TMEM200A	transmembrane protein 200A
TC1000007305.hg.1	1.7	0.0066	ANKRD30A	ankyrin repeat domain 30A
TC0200007803.hg.1	1.7	0.0129	LGALSL	lectin, galactoside-binding-like
TC0300008713.hg.1	1.7	0.013	SEC61A1	Sec61 translocon alpha 1 subunit
TC1200011535.hg.1	1.7	0.0235	UBE2N	ubiquitin conjugating enzyme E2N
TSUnmapped00000224.hg.1	1.7	0.0355	ZDHHC3	zinc finger, DHHC-type containing 3
TC0600008972.hg.1	1.69	0.01	PRDM1	PR domain containing 1, with ZNF domain
TSUnmapped00000487.hg.1	1.69	0.0118	MANSC1	MANSC domain containing 1
TC0300006596.hg.1	1.69	0.0261	SLC6A11	solute carrier family 6 (neurotransmitter transporter), member 11
TC0X00010678.hg.1	1.68	0.0069	C1GALT1C1	C1GALT1 specific chaperone 1
TC1300007780.hg.1	1.66	0.0221	RAP2A	RAP2A, member of RAS oncogene family
TC1400010760.hg.1	1.66	0.0393	ATP6V1D	ATPase, H+ transporting, lysosomal 34kDa, V1 subunit D
TC0100007672.hg.1	1.65	0.0007	EIF3I	eukaryotic translation initiation factor 3, subunit I
TC0200010100.hg.1	1.65	0.0408	AGPS	alkylglycerone phosphate synthase
TC0700008857.hg.1	1.65	0.0456	MDFIC	MyoD family inhibitor domain containing
TC1300006729.hg.1	1.64	0.0119	POMP	proteasome maturation protein
TC0X00008940.hg.1	1.63	0.0108	MXRA5	matrix-remodelling associated 5
TC1100008469.hg.1	1.63	0.016	SERPINH1	serpin peptidase inhibitor, clade H (heat shock protein 47), member 1, (collagen binding protein 1)
TC0700012443.hg.1	1.63	0.031	FEZF1	FEZ family zinc finger 1
TC0Y00007250.hg.1	1.63	0.0483	DAZ3	deleted in azoospermia 3

TSUnmapped00000489.hg.1	1.62	0.0097	SURF4	surfeit 4
TC0500012059.hg.1	1.62	0.0159	VDAC1	voltage-dependent anion channel 1
TC0100007858.hg.1	1.62	0.0165	UTP11L	UTP11-like, U3 small nucleolar ribonucleoprotein (yeast)
TC1800007367.hg.1	1.62	0.0178	TCF4	Memczak2013 ANTISENSE, coding, INTERNAL, intronic best transcript NM_001243234
TC1400009770.hg.1	1.62	0.0372	ZDHHC22	zinc finger, DHHC-type containing 22
TC1200012576.hg.1	1.61	0.0108	NDUFA9	NADH dehydrogenase (ubiquinone) 1 alpha subcomplex, 9, 39kDa
TC1100009450.hg.1	1.61	0.0237	DCPS	decapping enzyme, scavenger
TC0100018435.hg.1	1.61	0.0269	EBNA1BP2; MIR6733	EBNA1 binding protein 2; microRNA 6733
TC0300008152.hg.1	1.61	0.0345	TFG	TRK-fused gene
TC1700011787.hg.1	1.61	0.043	RNF157	ring finger protein 157
TC0600013010.hg.1	1.61	0.0459	MAN1A1	mannosidase, alpha, class 1A, member 1
TC1900009052.hg.1	1.6	0.0053	ZNF324B	zinc finger protein 324B
TC1100013223.hg.1	1.6	0.0464	CARD17	caspase recruitment domain family, member 17
TC0800007431.hg.1	1.59	0.0056	AP3M2	adaptor-related protein complex 3, mu 2 subunit
TC1200007558.hg.1	1.59	0.0113	PRPH	peripherin
TC2200008592.hg.1	1.59	0.0146	APOL2	apolipoprotein L, 2
TC0500010148.hg.1	1.59	0.0254	DNAH5	dynein, axonemal, heavy chain 5
TC0300009167.hg.1	1.59	0.0429	EIF2A	eukaryotic translation initiation factor 2A, 65kDa
TC1600008139.hg.1	1.58	0.0006	CES4A	carboxylesterase 4A
TC0200009806.hg.1	1.58	0.0035	TANK	TRAF family member-associated NFKB activator
TC1100006995.hg.1	1.58	0.0087	SAA1	serum amyloid A1

TC0200015893.hg.1	1.58	0.0304	DOCK10	dedicator of cytokinesis 10
TC0400012173.hg.1	1.58	0.0378	SFRP2	secreted frizzled-related protein 2
TC0100016406.hg.1	1.57	0.0141	VAMP4	vesicle associated membrane protein 4
TC0500009031.hg.1	1.57	0.0144	SPINK5	serine peptidase inhibitor, Kazal type 5
TC1600010685.hg.1	1.57	0.03	SMPD3	sphingomyelin phosphodiesterase 3, neutral membrane (neutral sphingomyelinase II)
TC0600013119.hg.1	1.57	0.0343	SOGA3; KIAA0408	SOGA family member 3; KIAA0408
TC1200011722.hg.1	1.56	0.0051	PMCH	pro-melanin-concentrating hormone
TC0300007739.hg.1	1.56	0.0274	PSMC1	Homo sapiens proteasome (prosome, macropain) 26S subunit, ATPase, 1, mRNA (cDNA clone MGC:8541 IMAGE:2822718), complete cds.; Homo sapiens proteasome (prosome, macropain) 26S subunit, ATPase, 1, mRNA (cDNA clone MGC:24583 IMAGE:4133348), complete cds.; Homo sapiens proteasome (prosome, macropain) 26S subunit, ATPase, 1, mRNA (cDNA clone MGC:86994 IMAGE:5264945), complete cds.; Homo sapiens proteasome (prosome, macropain) 26S subunit, ATPase, 1, mRNA (cDNA clone MGC:88853 IMAGE:5456334), complete cds.
TC0X00007188.hg.1	1.56	0.0313	FTSJ1	FtsJ RNA methyltransferase homolog 1 (E. coli)
TC1600006653.hg.1	1.55	0.0268	THOC6	THO complex 6
TC0300014093.hg.1	1.54	0.0098	PCYT1A	phosphate cytidylyltransferase 1, choline, alpha
TC0200007775.hg.1	1.54	0.0125	MDH1	malate dehydrogenase 1

TC0300007437.hg.1	1.54	0.0187	MANF	mesencephalic astrocyte-derived neurotrophic factor
TC1100011283.hg.1	1.54	0.0448	RIN1	Ras and Rab interactor 1
TC1800007218.hg.1	1.53	0.0356	C18orf25	chromosome 18 open reading frame 25
TC1700009478.hg.1	1.53	0.0401	OR1E1	olfactory receptor, family 1, subfamily E, member 1
TC1200011711.hg.1	1.52	0.0048	GNPTAB	N-acetylglucosamine-1-phosphate transferase, alpha and beta subunits
TC0200009167.hg.1	1.52	0.0135	CNTNAP5	contactin associated protein-like 5
TC1700007720.hg.1	1.52	0.0269	PSMB3	proteasome subunit beta 3
TC0X00007704.hg.1	1.52	0.0459	COX7B	cytochrome c oxidase subunit VIIb
TC0400009210.hg.1	1.52	0.0488	TRIM60	tripartite motif containing 60
TC1000011003.hg.1	1.5	0.0019	PLA2G12B	phospholipase A2, group XIIB
TC0100018315.hg.1	1.5	0.0095	UCK2; MIR3658	uridine-cytidine kinase 2; microRNA 3658
TC1900007787.hg.1	1.5	0.0132	KCTD15	potassium channel tetramerization domain containing 15
TC0100015931.hg.1	1.5	0.0409	PMVK	phosphomevalonate kinase
TC0200008230.hg.1	1.49	0.0176	TMSB10	thymosin beta 10
TC0600011508.hg.1	1.49	0.0325	TAP1	transporter 1, ATP-binding cassette, sub-family B (MDR/TAP)
TC0800010043.hg.1	1.49	0.0402	PPP2CB	protein phosphatase 2, catalytic subunit, beta isozyme
TC1900011846.hg.1	1.49	0.0423	PLIN5	perilipin 5
TC1600010604.hg.1	1.49	0.0459	FAM96B	family with sequence similarity 96, member B
TC2100008417.hg.1	1.49	0.0463	COL18A1-AS1	COL18A1 antisense RNA 1
TC1900010622.hg.1	1.49	0.047	LGALS7; LGALS7B	lectin, galactoside-binding, soluble, 7; lectin, galactoside-binding, soluble, 7B
TC0300011089.hg.1	1.48	0.0199	MON1A	MON1 secretory trafficking family member A

TC0800008370.hg.1	1.48	0.029	POLR2K	polymerase (RNA) II (DNA directed) polypeptide K, 7.0kDa
TC0X00010804.hg.1	1.48	0.0404	ZNF280C	zinc finger protein 280C
TC0600009789.hg.1	1.47	0.0014	TAB2	TGF-beta activated kinase 1/MAP3K7 binding protein 2
TC0800007245.hg.1	1.47	0.0033	MAK16	MAK16 homolog
TC0600013781.hg.1	1.47	0.0064	LPA	lipoprotein, Lp(a)
TSUnmapped00000384.hg.1	1.47	0.0146	ZNF546	zinc finger protein 546
TC1100010524.hg.1	1.47	0.0475	APIP	Transcript Identified by AceView, Entrez Gene ID(s) 51074
TSUnmapped00000258.hg.1	1.46	0.0394	SURF4	surfeit 4
TC0900008545.hg.1	1.46	0.0462	PAPPA	pregnancy-associated plasma protein A, pappalysin 1
TC1600008007.hg.1	1.45	0.0204	ADGRG3	adhesion G protein-coupled receptor G3
TC0500006744.hg.1	1.45	0.0205	ROPN1L	rhophilin associated tail protein 1-like
TC0200010157.hg.1	1.45	0.0296	ITGA4	integrin alpha 4
TC1800009214.hg.1	1.45	0.0419	NDUFV2	NADH dehydrogenase (ubiquinone) flavoprotein 2, 24kDa
TC0100018182.hg.1	1.45	0.0458	SZRD1	SUZ RNA binding domain containing 1
TC1700012192.hg.1	1.43	0.0161	NDEL1	nudE neurodevelopment protein 1-like 1
TC1900010301.hg.1	1.43	0.0225	C19orf12	chromosome 19 open reading frame 12
TC2200007361.hg.1	1.43	0.0401	TOMM22	translocase of outer mitochondrial membrane 22 homolog (yeast)
TC0700009233.hg.1	1.43	0.0417	BPGM	Transcript Identified by AceView, Entrez Gene ID(s) 669
TC1100010990.hg.1	1.42	0.0171	MRPL16	mitochondrial ribosomal protein L16
TC0600008027.hg.1	1.42	0.0198	BYSL	bystin-like
TC1200011327.hg.1	1.42	0.0231	CSRP2	cysteine and glycine-rich protein 2
TC0900008156.hg.1	1.42	0.0338	NCBP1	nuclear cap binding protein subunit 1

TC0100013837.hg.1	1.42	0.0365	RIMS3	regulating synaptic membrane exocytosis 3
TC0100014626.hg.1	1.42	0.0416	ASB17	ankyrin repeat and SOCS box containing 17
TC0600012288.hg.1	1.41	0.0151	DPPA5	developmental pluripotency associated 5
TC0900011837.hg.1	1.41	0.0292	ABO	ABO blood group (transferase A, alpha 1-3-N-acetylgalactosaminyltransferase; transferase B, alpha 1-3-galactosyltransferase)
TC1900007004.hg.1	1.4	0.039	C19orf52	chromosome 19 open reading frame 52
TC1000007847.hg.1	1.4	0.047	MYPN	myopalladin
TC1000008431.hg.1	0.02	0.0000699	PCGF5	polycomb group ring finger 5
TC1500009218.hg.1	-1.4	0.0164	ZSCAN29	zinc finger and SCAN domain containing 29
TC1900006539.hg.1	-1.4	0.0256	PLK5	polo-like kinase 5
TC2100007376.hg.1	-1.4	0.0486	KRTAP10-9	keratin associated protein 10-9
TC1600011306.hg.1	-1.41	0.0101	PRDM7	PR domain containing 7
TC0X00011144.hg.1	-1.41	0.0198	PNMA5	paraneoplastic Ma antigen family member 5
TC0800010667.hg.1	-1.41	0.0238	PDE7A	phosphodiesterase 7A
TC0900008842.hg.1	-1.42	0.0086	SLC25A25	Zhang2013 ALT_ACCEPTOR, ALT_DONOR, coding, INTERNAL, intronic best transcript NM_001006641
TC0100014133.hg.1	-1.42	0.0117	AGBL4	ATP/GTP binding protein-like 4
TC0500013016.hg.1	-1.42	0.0209	COL23A1	collagen, type XXIII, alpha 1
TC0100010167.hg.1	-1.42	0.0318	TSACC	TSSK6 activating co-chaperone
TC0500007641.hg.1	-1.42	0.0361	PIK3R1	phosphoinositide-3-kinase, regulatory subunit 1 (alpha)
TC1100008054.hg.1	-1.43	0.0061	PACS1	phosphofurin acidic cluster sorting protein 1

TC0500013385.hg.1	-1.43	0.0234	SH3TC2	SH3 domain and tetratricopeptide repeats 2
TC1700009186.hg.1	-1.43	0.0274	CCDC137	coiled-coil domain containing 137
TC0100015401.hg.1	-1.43	0.0333	IGSF3	immunoglobulin superfamily, member 3
TC0900009040.hg.1	-1.43	0.0385	C9orf9	chromosome 9 open reading frame 9
TC1900011908.hg.1	-1.43	0.0422	PDE4C	phosphodiesterase 4C, cAMP-specific
TC0200007825.hg.1	-1.44	0.018	CEP68	centrosomal protein 68kDa
TC0400010694.hg.1	-1.44	0.0312	ERVMER34-1	endogenous retrovirus group MER34, member 1
TC1200007356.hg.1	-1.45	0.031	MUC19	mucin 19, oligomeric
TC0700011477.hg.1	-1.45	0.0349	TRIM74	tripartite motif containing 74
TC1700010565.hg.1	-1.45	0.0366	FBXL20	F-box and leucine-rich repeat protein 20
TC0400009080.hg.1	-1.46	0.0296	NPY2R	neuropeptide Y receptor Y2
TC1700007777.hg.1	-1.46	0.0401	THRA	thyroid hormone receptor, alpha
TC0600012116.hg.1	-1.46	0.0443	COL21A1	collagen, type XXI, alpha 1
TC0100018207.hg.1	-1.47	0.0189	TSSK3	testis-specific serine kinase 3
TSUnmapped00000415.hg.1	-1.47	0.0256	INPP5D	inositol polyphosphate-5-phosphatase D
TC0800009190.hg.1	-1.47	0.0316	ZNF696	zinc finger protein 696
TC1600009397.hg.1	-1.47	0.0394	PRM3	protamine 3
TC1100010015.hg.1	-1.47	0.0406	NLRP10	NLR family, pyrin domain containing 10
TC0900008532.hg.1	-1.47	0.0428	DEC1	deleted in esophageal cancer 1
TC0500007050.hg.1	-1.48	0.0169	PDZD2	PDZ domain containing 2
TC0100013809.hg.1	-1.48	0.045	TRIT1	tRNA isopentenyltransferase 1
TC1600008008.hg.1	-1.49	0.0108	DRC7	dynein regulatory complex subunit 7
TC0200015645.hg.1	-1.5	0.0099	ABCA12	ATP binding cassette subfamily A member 12
TC0900007827.hg.1	-1.5	0.0202	SPATA31E1	SPATA31 subfamily E, member 1
TC0300007438.hg.1	-1.5	0.0289	RBM15B	RNA binding motif protein 15B
TC1600010291.hg.1	-1.5	0.0308	SALL1	spalt-like transcription factor 1

TC1100009912.hg.1	-1.5	0.0339	OR52A1	olfactory receptor, family 52, subfamily A, member 1
TSUnmapped00000390.hg.1	-1.5	0.048	RPS6KA1	ribosomal protein S6 kinase, 90kDa, polypeptide 1
TC0500007396.hg.1	-1.51	0.0174	SNX18	sorting nexin 18
TC0900011613.hg.1	-1.51	0.0235	FAM102A	family with sequence similarity 102, member A
TC1100011206.hg.1	-1.51	0.0358	ZNHIT2	zinc finger, HIT-type containing 2
TC0900010147.hg.1	-1.52	0.0199	CNTNAP3B	contactin associated protein-like 3B
TC0200012092.hg.1	-1.52	0.0273	CCDC121	coiled-coil domain containing 121
TC0100018180.hg.1	-1.52	0.0297	CLCNKA	chloride channel, voltage-sensitive Ka
TC0100014790.hg.1	-1.52	0.0325	COL24A1	collagen, type XXIV, alpha 1
TC0500009141.hg.1	-1.52	0.0326	GRIA1	glutamate receptor, ionotropic, AMPA 1
TC0900012275.hg.1	-1.53	0.0252	GOLGA1	golgin A1
TC0300013820.hg.1	-1.53	0.0437	ZNF660	zinc finger protein 660
TC1700010865.hg.1	-1.53	0.0443	PLCD3	phospholipase C, delta 3
TC0300008705.hg.1	-1.54	0.0158	ABTB1	ankyrin repeat and BTB (POZ) domain containing 1
TC2000008052.hg.1	-1.55	0.006	BIRC7	baculoviral IAP repeat containing 7
TC0700007984.hg.1	-1.55	0.0275	VPS37D	vacuolar protein sorting 37 homolog D (S. cerevisiae)
TC0X00008017.hg.1	-1.55	0.0337	PLP1	proteolipid protein 1
TC1200012153.hg.1	-1.55	0.037	PXN	paxillin
TC1700010811.hg.1	-1.56	0.002	HDAC5	histone deacetylase 5
TC1600011517.hg.1	-1.56	0.0108	DOC2A	double C2-like domains, alpha
TC1200011491.hg.1	-1.56	0.0281	EPYC	epiphycan
TC1600010467.hg.1	-1.56	0.0476	CNGB1	cyclic nucleotide gated channel beta 1
TC0600008745.hg.1	-1.56	0.0485	SPACA1	sperm acrosome associated 1

TC1100007571.hg.1	-1.58	0.0027	OR4A8	olfactory receptor, family 4, subfamily A, member 8 (gene/pseudogene) [Source:HGNC Symbol;Acc:HGNC:15165]
TSUnmapped00000580.hg.1	-1.58	0.0116	INPP5D	inositol polyphosphate-5-phosphatase D
TC0300013813.hg.1	-1.58	0.0155	ACKR2	atypical chemokine receptor 2
TC1900007829.hg.1	-1.58	0.0261	GRAMD1A	GRAM domain containing 1A
TC0200016330.hg.1	-1.58	0.0341	AQP12B	aquaporin 12B
TC1900010031.hg.1	-1.58	0.0454	COMP	cartilage oligomeric matrix protein
TC0100010651.hg.1	-1.59	0.0015	LOC730159; RP11-360D2.1	uncharacterized LOC730159; putative novel transcript
TC1000012198.hg.1	-1.59	0.0053	C10orf90	chromosome 10 open reading frame 90
TC0300013915.hg.1	-1.59	0.013	TPRG1	tumor protein p63 regulated 1
TC1900011947.hg.1	-1.59	0.016	LRFN1	leucine rich repeat and fibronectin type III domain containing 1
TC0500013336.hg.1	-1.59	0.0231	SSBP2	single-stranded DNA binding protein 2
TC1500010460.hg.1	-1.59	0.0372	HDDC3	HD domain containing 3
TC1400009780.hg.1	-1.59	0.0411	NOXRED1	NADP-dependent oxidoreductase domain containing 1
TC0400009037.hg.1	-1.59	0.0474	MND1	meiotic nuclear divisions 1
TC0100013992.hg.1	-1.6	0.0003	TCTEX1D4	Tctex1 domain containing 4
TC0200013108.hg.1	-1.6	0.0024	LBX2	ladybird homeobox 2
TC1900010370.hg.1	-1.6	0.0388	SLC7A9	solute carrier family 7 (amino acid transporter light chain, bo,+ system), member 9
TC1700009640.hg.1	-1.6	0.0411	ZBTB4	zinc finger and BTB domain containing 4
TC1700008820.hg.1	-1.6	0.0457	C17orf80	chromosome 17 open reading frame 80
TC1100007577.hg.1	-1.6	0.0471	TRIM48	tripartite motif containing 48
TC0100015865.hg.1	-1.61	0.0143	S100A5	S100 calcium binding protein A5

TC0300007733.hg.1	-1.61	0.0153	FAM19A1	family with sequence similarity 19 (chemokine (C-C motif)-like), member A1
TC0900009165.hg.1	-1.61	0.0428	LCN1	lipocalin 1
TC0100012950.hg.1	-1.61	0.0436	LRRC38	leucine rich repeat containing 38
TC1200010839.hg.1	-1.62	0.005	ITGA5	integrin alpha 5
TC0X00006686.hg.1	-1.62	0.0189	GRPR	gastrin-releasing peptide receptor
TC0300012768.hg.1	-1.62	0.0322	CP	ceruloplasmin (ferroxidase)
TC0600014136.hg.1	-1.62	0.0433	ADGRF4	adhesion G protein-coupled receptor F4
TC0100015872.hg.1	-1.63	0.0053	S100A14	S100 calcium binding protein A14
TC1100007624.hg.1	-1.63	0.0357	OR8I2	olfactory receptor, family 8, subfamily I, member 2
TC1000010483.hg.1	-1.64	0.0174	C10orf25	chromosome 10 open reading frame 25
TC0200007978.hg.1	-1.64	0.0306	ANKRD53	ankyrin repeat domain 53
TC2000009210.hg.1	-1.64	0.0464	WFDC5	WAP four-disulfide core domain 5
TC1900010988.hg.1	-1.65	0.0146	PNMAL1	paraneoplastic Ma antigen family-like 1
TC2200006881.hg.1	-1.65	0.0245	CABIN1	calcineurin binding protein 1
TC0X00006877.hg.1	-1.65	0.0323	MAGEB3	Transcript Identified by AceView, Entrez Gene ID(s) 4114
TC0300012392.hg.1	-1.65	0.0368	EFCAB12	EF-hand calcium binding domain 12
TC1200012768.hg.1	-1.65	0.0477	RERG	RAS-like, estrogen-regulated, growth inhibitor
TC2000009912.hg.1	-1.65	0.0491	CNBD2	cyclic nucleotide binding domain containing 2
TC0700011010.hg.1	-1.66	0.0278	SUN3	Sad1 and UNC84 domain containing 3
TC0200014688.hg.1	-1.67	0.003	ERMN	ermin
TC0X00007424.hg.1	-1.67	0.0226	ZXDB	zinc finger, X-linked, duplicated B
TC0600011738.hg.1	-1.67	0.0342	KCNK17	potassium channel, two pore domain subfamily K, member 17
TC0100012328.hg.1	-1.67	0.0423	TRIM58	tripartite motif containing 58
TC0300013958.hg.1	-1.67	0.0479	HHATL	hedgehog acyltransferase-like
TC0100016171.hg.1	-1.68	0.0124	MPZ	myelin protein zero

TC1100007865.hg.1	-1.68	0.0191	METTL12; SNORA57	methyltransferase like 12; small nucleolar RNA, H/ACA box 57
TC0100012169.hg.1	-1.68	0.0274	WDR64	WD repeat domain 64
TC0600011131.hg.1	-1.68	0.0304	HIST1H1T	histone cluster 1, H1t
TC0700012448.hg.1	-1.68	0.0325	RNF133	ring finger protein 133
TC0200015127.hg.1	-1.68	0.0472	PDE1A	Transcript Identified by AceView, Entrez Gene ID(s) 5136
TC2000007936.hg.1	-1.68	0.0483	PHACTR3	phosphatase and actin regulator 3
TC0100013024.hg.1	-1.69	0.0217	FAM131C	family with sequence similarity 131, member C
TC0700009300.hg.1	-1.69	0.026	LOC100130880; AC022173.2	uncharacterized LOC100130880; novel transcript
TSUnmapped00000654.hg.1	-1.69	0.0308	FMN1	formin 1
TC0600014138.hg.1	-1.69	0.0355	MLIP	muscular LMNA-interacting protein
TC0X00008694.hg.1	-1.69	0.0402	HSFX1	heat shock transcription factor family, X-linked 1
TC0X00009044.hg.1	-1.69	0.0416	GPR143	G protein-coupled receptor 143
TC0400009467.hg.1	-1.71	0.0062	TRAPPC11	trafficking protein particle complex 11
TC0X00008002.hg.1	-1.71	0.0198	NGFRAP1	nerve growth factor receptor (TNFRSF16) associated protein 1
TC1100009615.hg.1	-1.71	0.0222	VPS26B	VPS26 retromer complex component B
TC0X00010990.hg.1	-1.71	0.0434	SPANXC; SPANXD	SPANX family, member C; SPANX family, member D
TC1200011083.hg.1	-1.72	0.0125	C12orf56	chromosome 12 open reading frame 56
TC0X00010893.hg.1	-1.72	0.0453	CT55	cancer/testis antigen 55
TC1400008637.hg.1	-1.73	0.0039	SALL2	spalt-like transcription factor 2
TC0700011066.hg.1	-1.73	0.0091	COBL	cordon-bleu WH2 repeat protein
TC1900011668.hg.1	-1.73	0.0247	ZNF763	zinc finger protein 763

TC0300012319.hg.1	-1.74	0.0118	goyborbu; MCM2	Transcript Identified by AceView; Memczak2013 ANTISENSE, CDS, coding, INTERNAL, UTR3 best transcript NM_004526
TC1600006738.hg.1	-1.74	0.0176	DNAJA3	DnaJ (Hsp40) homolog, subfamily A, member 3
TSUnmapped00000135.hg.1	-1.74	0.0294	INPP5D	inositol polyphosphate-5-phosphatase D
TC1900009791.hg.1	-1.75	0.008	C19orf57	chromosome 19 open reading frame 57
TC0300013989.hg.1	-1.75	0.0132	ABHD14B	abhydrolase domain containing 14B
TC0300009139.hg.1	-1.75	0.0149	TM4SF4	transmembrane 4 L six family member 4
TC0500013246.hg.1	-1.75	0.0348	PCDHB10	protocadherin beta 10
TC0200012404.hg.1	-1.75	0.038	ZFP36L2	ZFP36 ring finger protein-like 2
TC0200007388.hg.1	-1.76	0.0175	LINC01126	long intergenic non-protein coding RNA 1126
TC1700010604.hg.1	-1.76	0.024	NR1D1	nuclear receptor subfamily 1, group D, member 1
TC1500010051.hg.1	-1.76	0.0245	RPP25	ribonuclease P/MRP 25kDa subunit
TSUnmapped00000446.hg.1	-1.76	0.0348	INPP5D	inositol polyphosphate-5-phosphatase D
TC0Y00006861.hg.1	-1.76	0.0439	GTPBP6	Homo sapiens GTP binding protein 6 (putative) (GTPBP6), mRNA.; GTP binding protein 6 (putative) [Source:HGNC Symbol;Acc:HGNC:30189]; Homo sapiens GTP binding protein 6 (putative), mRNA (cDNA clone MGC:1170 IMAGE:3528598), complete cds.; Salzman2013 ANNOTATED, CDS, coding, INTERNAL, OVCODE, OVERLAPTX, OVEXON best transcript NM_012227
TC0200015023.hg.1	-1.77	0.0068	EVX2	even-skipped homeobox 2

TC0500013064.hg.1	-1.78	0.0307	MGAT4B	mannosyl (alpha-1,3-)-glycoprotein beta-1,4-N-acetylglucosaminyltransferase, isozyme B
TC1900008303.hg.1	-1.79	0.0292	CLASRP	CLK4-associating serine/arginine rich protein
TC1700007520.hg.1	-1.79	0.0403	CDK5R1	cyclin-dependent kinase 5, regulatory subunit 1 (p35)
TC0100018100.hg.1	-1.79	0.0447	OR2B11	olfactory receptor, family 2, subfamily B, member 11
TC0300007161.hg.1	-1.8	0.0263	VIPR1	vasoactive intestinal peptide receptor 1
TC2100008457.hg.1	-1.8	0.0441	COL6A2	Jeck2013 ANTISENSE, CDS, coding, INTERNAL, intronic, OVCODE, OVEXON best transcript NM_058175
TC0600011401.hg.1	-1.81	0.0384	C6orf15	chromosome 6 open reading frame 15
TC1500009179.hg.1	-1.82	0.0102	PLA2G4D	phospholipase A2, group IVD (cytosolic)
TC1900008604.hg.1	-1.82	0.0103	MGC45922; CTD-2568A17.5	uncharacterized LOC284365; uncharacterized LOC284365 [Source:EntrezGene;Acc:284365]; novel transcript
TC0900009922.hg.1	-1.82	0.0113	FAM205C	family with sequence similarity 205, member C
TC0200010839.hg.1	-1.82	0.0225	TUBA4B	tubulin, alpha 4b
TC0300009454.hg.1	-1.83	0.0139	LRRIQ4	leucine-rich repeats and IQ motif containing 4
TC0200016420.hg.1	-1.83	0.0152	MRPL33	mitochondrial ribosomal protein L33
TC1000012481.hg.1	-1.83	0.0167	CYP2C19	cytochrome P450, family 2, subfamily C, polypeptide 19
TC1300008793.hg.1	-1.84	0.033	TSC22D1	TSC22 domain family, member 1
TC0600012329.hg.1	-1.84	0.0397	IMPG1	interphotoreceptor matrix proteoglycan 1
TC1200012577.hg.1	-1.84	0.0457	GALNT8	polypeptide N-acetylgalactosaminyltransferase 8

TC1200011993.hg.1	-1.85	0.0002	SLC8B1	solute carrier family 8 (sodium/lithium/calcium exchanger), member B1
TC0100013293.hg.1	-1.85	0.0199	ID3	inhibitor of DNA binding 3, dominant negative helix-loop-helix protein
TC0100011472.hg.1	-1.85	0.0214	HHAT	hedgehog acyltransferase
TC0300011951.hg.1	-1.85	0.0337	MORC1	MORC family CW-type zinc finger 1
TC0300006993.hg.1	-1.85	0.0379	CRTAP	cartilage associated protein
TC0800011148.hg.1	-1.87	0.0091	CCNE2	cyclin E2
TC1100013174.hg.1	-1.87	0.0143	BSCL2	Berardinelli-Seip congenital lipodystrophy 2 (seipin)
TC0500013239.hg.1	-1.88	0.0263	PCDHB3	protocadherin beta 3
TC0300007121.hg.1	-1.88	0.0363	ZNF619	zinc finger protein 619
TC0100012453.hg.1	-1.89	0.0049	TNFRSF4	tumor necrosis factor receptor superfamily, member 4
TC1600011581.hg.1	-1.89	0.0409	KLHDC4	kelch domain containing 4
TC0600011228.hg.1	-1.89	0.0456	HIST1H2AK	histone cluster 1, H2ak
TSUnmapped00000306.hg.1	-1.89	0.0482	ZKSCAN7	zinc finger with KRAB and SCAN domains 7
TC0X00010468.hg.1	-1.9	0.0117	FRMPD3-AS1	FRMPD3 antisense RNA 1
TC0200007200.hg.1	-1.9	0.0213	LTBP1	latent transforming growth factor beta binding protein 1
TC0600007694.hg.1	-1.91	0.0274	B3GALT4	UDP-Gal:betaGlcNAc beta 1,3-galactosyltransferase 4
TC1500010853.hg.1	-1.91	0.0337	FSIP1	fibrous sheath interacting protein 1
TC1900009158.hg.1	-1.92	0.0367	RPS15	Memczak2013 ANTISENSE, coding, INTERNAL, intronic best transcript NM_001018
TC1500007753.hg.1	-1.93	0.0069	THSD4	Transcript Identified by AceView, Entrez Gene ID(s) 79875
TC1400010604.hg.1	-1.93	0.01	MIA2	melanoma inhibitory activity 2
TC1500007804.hg.1	-1.94	0.0041	BBS4	Bardet-Biedl syndrome 4

TC1500009261.hg.1	-1.94	0.0104	SPG11	spastic paraplegia 11 (autosomal recessive)
TC2000006508.hg.1	-1.94	0.0346	TGM3	transglutaminase 3
TC1700010802.hg.1	-1.95	0.0028	MPP2	membrane protein, palmitoylated 2
TSUnmapped00000572.hg.1	-1.95	0.0165	RPS6KA1	ribosomal protein S6 kinase, 90kDa, polypeptide 1
TC0300013914.hg.1	-1.96	0.0107	TPRG1	tumor protein p63 regulated 1
TC1100009407.hg.1	-1.97	0.0119	CCDC15	coiled-coil domain containing 15
TC1100006911.hg.1	-1.97	0.04	SPON1	spondin 1, extracellular matrix protein
TC1400008752.hg.1	-1.99	0.0475	CIDEB	cell death-inducing DFFA-like effector b
TC1600007308.hg.1	-2.01	0.0005	KDM8	lysine (K)-specific demethylase 8
TC0900012185.hg.1	-2.01	0.0487	CEL	carboxyl ester lipase
TC0600007092.hg.1	-2.03	0.0069	FAM8A1	family with sequence similarity 8, member A1
TC1200012594.hg.1	-2.05	0.0196	ZNF705A; FAM66C	zinc finger protein 705A; family with sequence similarity 66, member C
TC2100007904.hg.1	-2.06	0.026	KRTAP22-2	keratin associated protein 22-2
TC1900011913.hg.1	-2.07	0.0176	LINC00663	long intergenic non-protein coding RNA 663
TC0700010015.hg.1	-2.07	0.0475	INTS1	integrator complex subunit 1
TC0300013277.hg.1	-2.08	0.0396	PEX5L	Transcript Identified by AceView, Entrez Gene ID(s) 51555
TC1500007645.hg.1	-2.1	0.0345	MAP2K5	mitogen-activated protein kinase kinase 5
TC0100007922.hg.1	-2.1	0.0358	PPIE	Transcript Identified by AceView, Entrez Gene ID(s) 10450
TC1400010603.hg.1	-2.11	0.011	TTC6	tetratricopeptide repeat domain 6
TC0800010252.hg.1	-2.12	0.0394	ANK1	ankyrin 1, erythrocytic
TC0700013254.hg.1	-2.17	0.009	PTPRN2	protein tyrosine phosphatase, receptor type, N polypeptide 2
TC1900011879.hg.1	-2.2	0.0162	ZNF44	zinc finger protein 44
TC1700010515.hg.1	-2.23	0.0156	TBC1D3	TBC1 domain family, member 3

TC1100013045.hg.1	-2.25	0.0057	SIPA1	signal-induced proliferation-associated 1
TC2200009345.hg.1	-2.26	0.0109	C1QTNF6	C1q and tumor necrosis factor related protein 6
TC1100007220.hg.1	-2.26	0.0388	DEPDC7	DEP domain containing 7
TC0100012942.hg.1	-2.28	0.0145	PRAMEF18	PRAME family member 18
TC0300007014.hg.1	-2.29	0.0065	ARPP21	Transcript Identified by AceView, Entrez Gene ID(s) 10777
TSUnmapped00000401.hg.1	-2.32	0.0497	INPP5D	inositol polyphosphate-5-phosphatase D
TC1200008795.hg.1	-2.37	0.0002	ACACB	acetyl-CoA carboxylase beta
TC0300008183.hg.1	-2.37	0.016	CEP97	Transcript Identified by AceView, Entrez Gene ID(s) 79598
TC1200009834.hg.1	-2.46	0.0362	MFAP5	microfibrillar associated protein 5
TC0700012995.hg.1	-2.49	0.0339	ZNF777	zinc finger protein 777
TC0400006930.hg.1	-2.54	0.0301	CPEB2	cytoplasmic polyadenylation element binding protein 2
TC1800007092.hg.1	-2.56	0.0359	MOCOS	molybdenum cofactor sulfurase
TC0100016296.hg.1	-2.59	0.009	GPA33	glycoprotein A33 (transmembrane)
TC0300013350.hg.1	-2.64	0.0248	YEATS2	Memczak2013 ANTISENSE, coding, INTERNAL, intronic best transcript NM_018023
TC1600011474.hg.1	-2.68	0.0224	ANKS3	ankyrin repeat and sterile alpha motif domain containing 3
TC0200011379.hg.1	-2.76	0.0187	SNED1	sushi, nidogen and EGF-like domains 1
TC0100018129.hg.1	-2.99	0.0316	OR2T29	olfactory receptor, family 2, subfamily T, member 29

(H) CXCL8 and IL-32α treated T cells

ID	FC CXCL8 IL32 vs CONTROL	P-val	Gene Symbol	Description
TC0100015608.hg.1	3.95	0.0046	LOC105371242; AC243756.1	peptidyl-prolyl cis-trans isomerase A-like 4G; Peptidyl-prolyl cis-trans isomerase [Source:UniProtKB/TrEMBL;Acc:A0A075B767]
TC0100014393.hg.1	3.69	0.0425	TM2D1	Transcript Identified by AceView, Entrez Gene ID(s) 83941
TC0300011049.hg.1	3.56	0.0294	QRICH1	Transcript Identified by AceView, Entrez Gene ID(s) 54870
TC1100009947.hg.1	2.58	0.0171	OR52E6	olfactory receptor, family 52, subfamily E, member 6
TSUnmapped00000213.hg.1	2.46	0.0484	DGKD	diacylglycerol kinase, delta 130kDa
TC0300012880.hg.1	2.44	0.0109	C3orf33	chromosome 3 open reading frame 33
TC0500007179.hg.1	2.39	0.0239	WDR70	WD repeat domain 70
TC1000006907.hg.1	2.35	0.0331	STAM	Memczak2013 ALT_ACCEPTOR, ALT_DONOR, INTERNAL, intronic, ncRNA best transcript NR_037774
TSUnmapped00000024.hg.1	2.34	0.0054	PSMC4	proteasome 26S subunit, ATPase 4 [Source:HGNC Symbol;Acc:HGNC:9551]
TC0700013458.hg.1	2.25	0.0196	HILPDA	hypoxia inducible lipid droplet-associated
TC0X00007562.hg.1	2.23	0.0411	ITGB1BP2	integrin beta 1 binding protein (melusin) 2
TC0X00009986.hg.1	2.2	0.0056	RP5-1091N2.9; IL2RG	Transcript Identified by AceView, Entrez Gene ID(s) 3561; putative novel transcript
TC0100013805.hg.1	2.2	0.0336	OXCT2	3-oxoacid CoA-transferase 2
TC0400012881.hg.1	2.17	0.006	DUX4	double homeobox 4
TC0400010749.hg.1	2.16	0.0472	PDCL2	phosducin like 2

TC1900008848.hg.1	2.15	0.0447	LILRA5	Memczak2013 ANTISENSE, coding, INTERNAL, intronic best transcript NM_181985
TC1500009148.hg.1	2.12	0.0017	OIP5	Opa interacting protein 5
TC0200016099.hg.1	2.11	0.0255	HJURP	Holliday junction recognition protein
TC0400008641.hg.1	2.11	0.0314	FAT4	FAT atypical cadherin 4
TC0200016505.hg.1	2.1	0.0014	CCDC138	coiled-coil domain containing 138
TC0800012376.hg.1	2.1	0.0302	ZNF707	zinc finger protein 707
TC0800012316.hg.1	2.09	0.0203	XKR9	X-linked Kx blood group related 9
TSUnmapped00000821.hg.1	2.07	0.0083	DUX5	double homeobox 5
TC0400010529.hg.1	2.04	0.0032	APBB2	Jeck2013 ALT_ACCEPTOR, ALT_DONOR, coding, INTERNAL, intronic best transcript NM_004307
TC1100011716.hg.1	2.01	0.0021	GDPD4	glycerophosphodiester phosphodiesterase domain containing 4
TC1900008726.hg.1	2	0.0017	ERVV-1	endogenous retrovirus group V, member 1
TC0400008444.hg.1	1.99	0.0374	LARP7	Zhang2013 ALT_DONOR, coding, INTERNAL, intronic, OVERLAPTX, OVEXON best transcript NM_016648
TC0100007512.hg.1	1.98	0.0445	GPR3	G protein-coupled receptor 3
TC0200016073.hg.1	1.96	0.0098	KCNJ13	potassium channel, inwardly rectifying subfamily J, member 13
TC1900009639.hg.1	1.96	0.0175	AP1M2	adaptor-related protein complex 1, mu 2 subunit
TC1500007757.hg.1	1.95	0.0257	NR2E3	nuclear receptor subfamily 2, group E, member 3
TC0900008851.hg.1	1.94	0.0225	DNM1	dynamin 1
TC0600011406.hg.1	1.93	0.0076	POU5F1	POU class 5 homeobox 1
TC1900011690.hg.1	1.93	0.012	ZNF493	zinc finger protein 493
TC0X00010460.hg.1	1.92	0.002	RIPPLY1	ripply transcriptional repressor 1

TC1400008181.hg.1	1.87	0.0194	HHIPL1	HHIP-like 1
TC1800006806.hg.1	1.87	0.0341	ANKRD30B	ankyrin repeat domain 30B
TC1900011334.hg.1	1.86	0.0033	ZNF677	zinc finger protein 677
TC0500013372.hg.1	1.86	0.0157	ECSCR	endothelial cell surface expressed chemotaxis and apoptosis regulator
TC1600010837.hg.1	1.86	0.0237	C16orf47	chromosome 16 open reading frame 47
TC2100007600.hg.1	1.84	0.0266	LOC388813; bafor; AF165138.7	uncharacterized protein ENSP00000383407-like; Transcript Identified by AceView; HCG1818297; Protein LOC388813 [Source:UniProtKB/TrEMBL;Acc:A0A087WSY0]
TSUnmapped00000384.hg.1	1.83	0.0091	ZNF546	zinc finger protein 546
TC1500010739.hg.1	1.82	0.0106	SLC12A1	solute carrier family 12 (sodium/potassium/chloride transporter), member 1
TC2000009550.hg.1	-1.82	0.0144	AURKA	aurora kinase A
TC1700006611.hg.1	1.81	0.0093	CYB5D2	cytochrome b5 domain containing 2
TC1800007595.hg.1	1.79	0.0275	CCDC102B	coiled-coil domain containing 102B
TC0900010618.hg.1	1.79	0.0287	C9orf153	chromosome 9 open reading frame 153
TC0X00010943.hg.1	1.79	0.0287	GPR101	G protein-coupled receptor 101
TC1600006965.hg.1	1.79	0.0458	SHISA9	shisa family member 9
TC1100007414.hg.1	1.78	0.0192	LOC399886; RP11-430H10.2; FLJ41423	uncharacterized LOC399886; Transcript Identified by AceView, Entrez Gene ID(s) 399886; novel transcript
TC1600007131.hg.1	1.78	0.0265	ACSM2A	acyl-CoA synthetase medium-chain family member 2A
TC1500009384.hg.1	1.77	0.0224	HDC	histidine decarboxylase
TC1600009351.hg.1	1.77	0.0299	GRIN2A	Transcript Identified by AceView, Entrez Gene ID(s) 2903

TSUnmapped00000573.hg.1	1.77	0.0359	PRAMEF25	PRAME family member 25
TC1100006704.hg.1	1.76	0.0143	OR52N4	olfactory receptor, family 52, subfamily N, member 4 (gene/pseudogene)
TC0500013242.hg.1	1.76	0.0353	PCDHB6	protocadherin beta 6
TC1500007866.hg.1	1.76	0.0407	PPCDC	phosphopantothenoylcysteine decarboxylase
TC1500007238.hg.1	1.75	0.0211	TMOD2	tropomodulin 2 (neuronal)
TC1100010236.hg.1	1.75	0.0328	KCNJ11	potassium channel, inwardly rectifying subfamily J, member 11
TC0300006793.hg.1	1.73	0.0191	KAT2B	Transcript Identified by AceView, Entrez Gene ID(s) 8850
TC1900011511.hg.1	1.72	0.0159	ZSCAN5A	zinc finger and SCAN domain containing 5A
TC2100008517.hg.1	1.72	0.0322	WRB	tryptophan rich basic protein
TC1200011690.hg.1	1.71	0.0008	SLC5A8	solute carrier family 5 (sodium/monocarboxylate cotransporter), member 8
TC0600014140.hg.1	1.71	0.0059	PRIM2	Salzman2013 ANNOTATED, CDS, coding, OVCODE, OVEXON, UTR3 best transcript NM_000947
TC1900011846.hg.1	1.71	0.0124	PLIN5	perilipin 5
TC0200016693.hg.1	1.7	0.0498	LRRTM4	leucine rich repeat transmembrane neuronal 4
TC1900009052.hg.1	1.69	0.008	ZNF324B	zinc finger protein 324B
TC1600008438.hg.1	1.69	0.0189	CTRB1	chymotrypsinogen B1

TC15_KI270727v1_random00006441.hg.1	1.69	0.0424	POTEB3; POTEB; AC136352.4	Homo sapiens POTE ankyrin domain family, member B3 (POTEB3), transcript variant 1, mRNA.; Homo sapiens POTE ankyrin domain family, member B, mRNA (cDNA clone MGC:119371 IMAGE:40006483), complete cds.; Homo sapiens POTE ankyrin domain family, member B, mRNA (cDNA clone MGC:119372 IMAGE:40006485), complete cds.; Homo sapiens POTE ankyrin domain family, member B, mRNA (cDNA clone MGC:119373 IMAGE:40006489), complete cds.; Homo sapiens POTE ankyrin domain family, member B, mRNA (cDNA clone MGC:156116 IMAGE:40006490), complete cds.
TC1400008622.hg.1	1.68	0.0048	OR5AU1	olfactory receptor, family 5, subfamily AU, member 1
TSUnmapped00000487.hg.1	1.67	0.0034	MANSC1	MANSC domain containing 1
TC0X00011159.hg.1	1.67	0.0211	HAUS7; TREX2	HAUS augmin like complex subunit 7; three prime repair exonuclease 2
TC0X00007169.hg.1	1.67	0.0236	SSX6	synovial sarcoma, X breakpoint 6 (pseudogene)
TC1200009362.hg.1	1.65	0.0135	TMEM132C	transmembrane protein 132C
TC1100006689.hg.1	1.65	0.0407	OR51B6	olfactory receptor, family 51, subfamily B, member 6
TC0600013781.hg.1	1.64	0.0029	LPA	lipoprotein, Lp(a)

TC0500013230.hg.1	1.64	0.0118	PCBD2	pterin-4 alpha-carbinolamine dehydratase/dimerization cofactor of hepatocyte nuclear factor 1 alpha (TCF1) 2
TC1100006759.hg.1	1.64	0.0337	OLFML1	olfactomedin like 1
TC0800011062.hg.1	1.64	0.0421	NBN	Transcript Identified by AceView, Entrez Gene ID(s) 4683
TC1000011072.hg.1	1.63	0.0055	DUSP13	dual specificity phosphatase 13
TC1000012365.hg.1	1.63	0.0389	CFAP46	cilia and flagella associated protein 46
TC0800007377.hg.1	1.63	0.0495	ADAM18	ADAM metallopeptidase domain 18
TC1900007787.hg.1	1.62	0.015	KCTD15	potassium channel tetramerization domain containing 15
TC0700012443.hg.1	1.62	0.0195	FEZF1	FEZ family zinc finger 1
TC1400009770.hg.1	1.62	0.0217	ZDHHC22	zinc finger, DHHC-type containing 22
TC0100008183.hg.1	1.62	0.0446	CYP4Z1	cytochrome P450, family 4, subfamily Z, polypeptide 1
TC1500010291.hg.1	1.61	0.0136	NMB	neuromedin B
TC0X00007937.hg.1	1.61	0.0287	DRP2	dystrophin related protein 2
TC0X00008136.hg.1	1.6	0.0052	ALG13	ALG13, UDP-N-acetylglucosaminyltransferase subunit
TC1100007610.hg.1	1.59	0.0044	OR5D14	olfactory receptor, family 5, subfamily D, member 14
TC1100012420.hg.1	1.59	0.0274	APOA5	apolipoprotein A-V
TC0700009346.hg.1	1.59	0.0357	CLEC2L	C-type lectin domain family 2, member L
TC1200008642.hg.1	1.58	0.0024	PARPBP	PARP1 binding protein
TC1500007678.hg.1	1.57	0.0052	CORO2B	Memczak2013 ALT_ACCEPTOR, ALT_DONOR, coding, INTERNAL, intronic best transcript NM_001190457
TC0700010412.hg.1	1.57	0.0068	SP8	Sp8 transcription factor

TC0X00011393.hg.1	1.55	0.0106	CT47A5; CT47A2; CT47A1; CT47A11; CT47A12	cancer/testis antigen family 47, member A5; cancer/testis antigen family 47, member A2; cancer/testis antigen family 47, member A1; cancer/testis antigen family 47, member A11; cancer/testis antigen family 47, member A12
TC1700008011.hg.1	1.55	0.0323	DBF4B	DBF4 zinc finger B
TC0300010852.hg.1	1.55	0.0405	CCDC13	coiled-coil domain containing 13
TC0100017321.hg.1	1.54	0.0089	GPATCH2	G-patch domain containing 2
TC0800012475.hg.1	1.54	0.0128	JRK	Jrk helix-turn-helix protein
TC0600009406.hg.1	1.54	0.0152	C6orf58	chromosome 6 open reading frame 58
TC0200008464.hg.1	1.54	0.0205	KCNIP3	Kv channel interacting protein 3, calsenilin
TC1500009214.hg.1	1.53	0.0262	TGM5	transglutaminase 5
TC1600009246.hg.1	1.53	0.0465	ROGDI	rogdi homolog
TC1400006823.hg.1	1.53	0.0491	SCFD1	sec1 family domain containing 1
TC1000010661.hg.1	1.53	0.0497	MBL2	mannose-binding lectin (protein C) 2, soluble
TC0X00009029.hg.1	1.52	0.0174	FAM9A	family with sequence similarity 9, member A
TC1200010412.hg.1	1.52	0.0295	ABCD2	Transcript Identified by AceView, Entrez Gene ID(s) 225
TC0200013600.hg.1	1.52	0.0365	KIAA1211L	KIAA1211-like
TC1800007013.hg.1	1.52	0.0432	DSG3	desmoglein 3
TC0200012031.hg.1	1.52	0.0488	HADHA	Transcript Identified by AceView, Entrez Gene ID(s) 3030
TC0100012884.hg.1	1.51	0.0227	FBXO2	F-box protein 2
TC1700006642.hg.1	1.51	0.0339	TM4SF5	transmembrane 4 L six family member 5
TC1000011879.hg.1	1.51	0.0443	NRAP	nebulin-related anchoring protein
TC1100006509.hg.1	1.51	0.0452	MUC5B	mucin 5B, oligomeric mucus/gel-forming
TC0X00006616.hg.1	1.5	0.0228	FRMPD4	FERM and PDZ domain containing 4

TC1100007185.hg.1	1.5	0.0314	ELP4	elongator acetyltransferase complex subunit 4
TC0600008515.hg.1	1.5	0.0353	KCNQ5	Jeck2013 ALT_ACCEPTOR, ALT_DONOR, coding, INTERNAL, intronic best transcript NM_001160133
TC0500013029.hg.1	1.5	0.0404	AACSP1	acetoacetyl-CoA synthetase pseudogene 1
TC0800006968.hg.1	1.49	0.0171	FGF17	fibroblast growth factor 17
TC2000006899.hg.1	1.49	0.0218	XRN2	5-3 exoribonuclease 2
TC1700011088.hg.1	1.49	0.0273	COL1A1	collagen, type I, alpha 1
TC2200008467.hg.1	1.49	0.0359	PLA2G3	phospholipase A2, group III
TC1400009353.hg.1	1.49	0.0436	SIX4	SIX homeobox 4
TC0500012948.hg.1	1.49	0.049	SNCB	synuclein beta
TC0300013932.hg.1	1.48	0.0221	SRGAP3	SLIT-ROBO Rho GTPase activating protein 3
TC0600014251.hg.1	1.48	0.0315	ZBED9	zinc finger, BED-type containing 9
TC0700013567.hg.1	1.47	0.0485	LINC00174	long intergenic non-protein coding RNA 174
TC0X00007256.hg.1	1.47	0.0491	BMP15	bone morphogenetic protein 15
TC0800010171.hg.1	1.46	0.018	C8orf86	chromosome 8 open reading frame 86
TC1600007718.hg.1	1.46	0.0187	ORC6	origin recognition complex subunit 6
TC1500010718.hg.1	1.46	0.0365	DISP2	dispatched homolog 2 (Drosophila)
TC0400011563.hg.1	1.45	0.0122	ETNPPL	ethanolamine-phosphate phospho-lyase
TC1500006963.hg.1	1.44	0.0235	CASC5	cancer susceptibility candidate 5
TC2200008078.hg.1	1.44	0.0313	GGT2	gamma-glutamyltransferase 2
TC0900010667.hg.1	1.44	0.0317	SPATA31C2	SPATA31 subfamily C, member 2
TC0600009171.hg.1	1.44	0.0396	RFPL4B	ret finger protein-like 4B
TC1000012583.hg.1	1.43	0.0061	RRP12	ribosomal RNA processing 12 homolog
TC0800011138.hg.1	1.43	0.01	RAD54B	Transcript Identified by AceView, Entrez Gene ID(s) 25788
TC0X00010875.hg.1	1.43	0.043	PLAC1	placenta specific 1

TC0100015791.hg.1	1.42	0.0493	POGZ	Transcript Identified by AceView, Entrez Gene ID(s) 23126
TSUnmapped00000438.hg.1	1.41	0.0267	HMBS	hydroxymethylbilane synthase
TC1100010726.hg.1	1.41	0.0271	PACSIN3	protein kinase C and casein kinase substrate in neurons 3
TSUnmapped00000292.hg.1	-1.4	0.0114	TGM4	transglutaminase 4
TC0X00011194.hg.1	-1.4	0.0471	FLNA	filamin A, alpha
TC1600008008.hg.1	-1.41	0.0161	DRC7	dynein regulatory complex subunit 7
TC0X00008492.hg.1	-1.41	0.0206	SMIM10	small integral membrane protein 10
TC0200016555.hg.1	-1.41	0.042	TBR1	T-box, brain, 1
TC0600008955.hg.1	-1.42	0.0278	LIN28B	lin-28 homolog B (C. elegans)
TC1200011495.hg.1	-1.42	0.0414	LUM	lumican
TC1100010850.hg.1	-1.42	0.0471	OR5T2	olfactory receptor, family 5, subfamily T, member 2
TC1200012578.hg.1	-1.43	0.0081	KCNA6; GALNT8	potassium channel, voltage gated shaker related subfamily A, member 6; polypeptide N-acetylgalactosaminyltransferase 8
TC2000008807.hg.1	-1.43	0.0303	DEFB119	defensin, beta 119
TC1600009992.hg.1	-1.43	0.0416	BCL7C	B-cell CLL/lymphoma 7C
TC1400008488.hg.1	-1.44	0.0044	TMEM121	transmembrane protein 121
TC1600010669.hg.1	-1.44	0.0138	DDX28	DEAD (Asp-Glu-Ala-Asp) box polypeptide 28
TC1100007783.hg.1	-1.44	0.0193	ZP1	zona pellucida glycoprotein 1 (sperm receptor)
TC0X00007209.hg.1	-1.44	0.0261	PQBP1	polyglutamine binding protein 1
TC1000009527.hg.1	-1.44	0.0336	LARP4B	La ribonucleoprotein domain family, member 4B
TC0100009611.hg.1	-1.44	0.04	HSD3B2	hydroxy-delta-5-steroid dehydrogenase, 3 beta- and steroid delta-isomerase 2

TC1500007856.hg.1	-1.45	0.0114	CSK	c-src tyrosine kinase
TC1900011096.hg.1	-1.45	0.0168	FUT1	fucosyltransferase 1 (galactoside 2-alpha-L-fucosyltransferase, H blood group)
TC1600009942.hg.1	-1.45	0.039	CDIPT	CDP-diacylglycerol--inositol 3-phosphatidyltransferase
TC1900007871.hg.1	-1.45	0.0418	COX6B1	cytochrome c oxidase subunit VIb polypeptide 1 (ubiquitous)
TC1100009434.hg.1	-1.46	0.037	DDX25	DEAD (Asp-Glu-Ala-Asp) box helicase 25
TC0100015728.hg.1	-1.46	0.0434	APH1A	APH1A gamma secretase subunit
TC0600014112.hg.1	-1.46	0.0446	ZBTB9	zinc finger and BTB domain containing 9
TC0100013992.hg.1	-1.47	0.0012	TCTEX1D4	Tctex1 domain containing 4
TC0X00006839.hg.1	-1.47	0.0089	MAGEB6	MAGE family member B6
TC0800011625.hg.1	-1.47	0.0107	TAF2	TAF2 RNA polymerase II, TATA box binding protein (TBP)-associated factor, 150kDa
TC0200012417.hg.1	-1.47	0.0238	ABCG5	ATP binding cassette subfamily G member 5
TC0300008355.hg.1	-1.47	0.0239	BOC	BOC cell adhesion associated, oncogene regulated
TC1700007402.hg.1	-1.47	0.0401	FLOT2	Memczak2013 ANTISENSE, coding, INTERNAL, UTR3 best transcript NM_004475
TC0100014133.hg.1	-1.48	0.0058	AGBL4	ATP/GTP binding protein-like 4
TC1600009217.hg.1	-1.48	0.0229	SRL	sarcalumenin
TC0100018526.hg.1	-1.49	0.0159	ILDR2	immunoglobulin-like domain containing receptor 2
TC1900011679.hg.1	-1.49	0.017	CYP4F3	cytochrome P450, family 4, subfamily F, polypeptide 3
TC2200008367.hg.1	-1.49	0.0391	CHEK2	checkpoint kinase 2
TC0X00010051.hg.1	-1.5	0.0076	NAP1L6	nucleosome assembly protein 1-like 6

TSUnmapped00000801.hg.1	-1.5	0.0078	VPS11	VPS11, CORVET/HOPS core subunit [Source:HGNC Symbol;Acc:HGNC:14583]
TC0900012284.hg.1	-1.5	0.0221	DNLZ	DNL-type zinc finger
TC1700006808.hg.1	-1.5	0.0284	RANGRF	RAN guanine nucleotide release factor
TC1400006506.hg.1	-1.5	0.037	OR11G2	olfactory receptor, family 11, subfamily G, member 2
TC1700008014.hg.1	-1.5	0.0391	ADAM11	ADAM metallopeptidase domain 11
TSUnmapped00000351.hg.1	-1.5	0.0484	VPS11	VPS11, CORVET/HOPS core subunit [Source:HGNC Symbol;Acc:HGNC:14583]
TC0100010102.hg.1	-1.51	0.0477	FLAD1	flavin adenine dinucleotide synthetase 1
TC1200006648.hg.1	-1.51	0.0484	USP5	ubiquitin specific peptidase 5 (isopeptidase T)
TC1100008054.hg.1	-1.52	0.0108	PACS1	phosphofurin acidic cluster sorting protein 1
TC0200013108.hg.1	-1.52	0.0347	LBX2	ladybird homeobox 2
TC1600007723.hg.1	-1.53	0.0046	GPT2	glutamic pyruvate transaminase (alanine aminotransferase) 2
TC1900009368.hg.1	-1.53	0.0124	TICAM1	toll-like receptor adaptor molecule 1
TC0100009345.hg.1	-1.53	0.0138	CELSR2	cadherin, EGF LAG seven-pass G-type receptor 2
TC1400010331.hg.1	-1.53	0.0232	XRCC3	X-ray repair complementing defective repair in Chinese hamster cells 3
TC1700012366.hg.1	-1.53	0.0359	TBC1D27	TBC1 domain family, member 27
TC0900008842.hg.1	-1.53	0.0458	SLC25A25	Zhang2013 ALT_ACCEPTOR, ALT_DONOR, coding, INTERNAL, intronic best transcript NM_001006641
TC0100015781.hg.1	-1.54	0.02	PI4KB	phosphatidylinositol 4-kinase, catalytic, beta
TC0800007284.hg.1	-1.54	0.0361	KCNU1	potassium channel, subfamily U, member 1

TC0200015529.hg.1	-1.55	0.0203	MDH1B	malate dehydrogenase 1B
TC1100009911.hg.1	-1.55	0.0224	OR52A5	olfactory receptor, family 52, subfamily A, member 5
TSUnmapped00000552.hg.1	-1.55	0.0442	FBL	fibrillarin
TC1900011947.hg.1	-1.55	0.0446	LRFN1	leucine rich repeat and fibronectin type III domain containing 1
TC0100016917.hg.1	-1.56	0.0341	CSRP1	cysteine and glycine-rich protein 1
TC1000010827.hg.1	-1.57	0.0053	DNAJC12	DnaJ (Hsp40) homolog, subfamily C, member 12
TC0500012979.hg.1	-1.57	0.0266	DDX41	DEAD (Asp-Glu-Ala-Asp) box polypeptide 41
TC0X00008002.hg.1	-1.58	0.0417	NGFRAP1	nerve growth factor receptor (TNFRSF16) associated protein 1
TC0500007050.hg.1	-1.59	0.0086	PDZD2	PDZ domain containing 2
TC0200013779.hg.1	-1.59	0.0245	ST6GAL2	ST6 beta-galactosamide alpha-2,6-sialyltranferase 2
TC0300007733.hg.1	-1.59	0.0361	FAM19A1	family with sequence similarity 19 (chemokine (C-C motif)-like), member A1
TC0300014040.hg.1	-1.6	0.0142	TXNRD3NB; TXNRD3	thioredoxin reductase 3 neighbor; thioredoxin reductase 3
TC0100015593.hg.1	-1.6	0.0443	ANKRD35	ankyrin repeat domain 35
TC1200009914.hg.1	-1.61	0.0037	TAS2R7	taste receptor, type 2, member 7
TC1100007601.hg.1	-1.61	0.0238	OR4S2	olfactory receptor, family 4, subfamily S, member 2
TC2200009276.hg.1	-1.61	0.0457	SGSM3	small G protein signaling modulator 3
TC1900006645.hg.1	-1.61	0.0461	HMG20B	high mobility group 20B
TC1900006770.hg.1	-1.62	0.0203	DUS3L	Memczak2013 ANTISENSE, CDS, coding, INTERNAL best transcript NM_020175
TC1100007577.hg.1	-1.62	0.0306	TRIM48	tripartite motif containing 48
TC1000009067.hg.1	-1.62	0.0391	GRK5	G protein-coupled receptor kinase 5
TC1200007709.hg.1	-1.62	0.0421	PFDN5	prefoldin subunit 5

TC2100007377.hg.1	-1.62	0.0488	KRTAP10-10	keratin associated protein 10-10
TC1100009912.hg.1	-1.63	0.0069	OR52A1	olfactory receptor, family 52, subfamily A, member 1
TC0200012092.hg.1	-1.63	0.0201	CCDC121	coiled-coil domain containing 121
TC1200012577.hg.1	-1.63	0.0282	GALNT8	polypeptide N-acetylgalactosaminyltransferase 8
TC1600007308.hg.1	-1.64	0.0137	KDM8	lysine (K)-specific demethylase 8
TC0Y00006861.hg.1	-1.65	0.017	GTPBP6	Homo sapiens GTP binding protein 6 (putative) (GTPBP6), mRNA.; GTP binding protein 6 (putative) [Source:HGNC Symbol;Acc:HGNC:30189]; Homo sapiens GTP binding protein 6 (putative), mRNA (cDNA clone MGC:1170 IMAGE:3528598), complete cds.; Salzman2013 ANNOTATED, CDS, coding, INTERNAL, OVCODE, OVERLAPTX, OVEXON best transcript NM_012227
TC1500008271.hg.1	-1.65	0.0364	WDR93	WD repeat domain 93
TC1900008604.hg.1	-1.68	0.0094	MGC45922; CTD-2568A17.5	uncharacterized LOC284365; uncharacterized LOC284365 [Source:EntrezGene;Acc:284365]; novel transcript
TC0900009922.hg.1	-1.69	0.0039	FAM205C	family with sequence similarity 205, member C
TC1600009966.hg.1	-1.69	0.0067	DCTPP1	dCTP pyrophosphatase 1
TC1700009640.hg.1	-1.69	0.0295	ZBTB4	zinc finger and BTB domain containing 4
TC1700008186.hg.1	-1.69	0.032	IGF2BP1	insulin-like growth factor 2 mRNA binding protein 1
TC1300009218.hg.1	-1.69	0.0458	DACH1	dachshund family transcription factor 1
TC1800007233.hg.1	-1.7	0.0403	KATNAL2	katanin p60 subunit A-like 2
TC1700010252.hg.1	-1.7	0.0477	CORO6	coronin 6
TC0300013950.hg.1	-1.71	0.0459	EFHB	EF-hand domain family, member B

TC1700012484.hg.1	-1.72	0.0328	PYCR1	pyrroline-5-carboxylate reductase 1
TC0600011465.hg.1	-1.72	0.0425	EHMT2	euchromatic histone-lysine N-methyltransferase 2
TC1400010604.hg.1	-1.73	0.0072	MIA2	melanoma inhibitory activity 2
TC0600013395.hg.1	-1.73	0.0385	NMBR	neuromedin B receptor
TC1600006686.hg.1	-1.73	0.0458	OR2C1	olfactory receptor, family 2, subfamily C, member 1
TC0300007438.hg.1	-1.74	0.0166	RBM15B	RNA binding motif protein 15B
TC0X00008888.hg.1	-1.76	0.0126	PPP2R3B	protein phosphatase 2, regulatory subunit B, beta
TC0Y00006863.hg.1	-1.76	0.0126	PPP2R3B	protein phosphatase 2, regulatory subunit B, beta
TC1100008139.hg.1	-1.76	0.0161	AIP	aryl hydrocarbon receptor interacting protein
TC0200016366.hg.1	-1.76	0.0466	THAP4	THAP domain containing 4
TC1300008072.hg.1	-1.77	0.0059	SPACA7	sperm acrosome associated 7
TC1100012314.hg.1	-1.77	0.0083	PIH1D2	PIH1 domain containing 2
TC1100013174.hg.1	-1.77	0.0183	BSCL2	Berardinelli-Seip congenital lipodystrophy 2 (seipin)
TC1500010051.hg.1	-1.78	0.005	RPP25	ribonuclease P/MRP 25kDa subunit
TC1500007143.hg.1	-1.78	0.0107	SEMA6D	sema domain, transmembrane domain (TM), and cytoplasmic domain, (semaphorin) 6D
TC2000007297.hg.1	-1.8	0.0414	VSTM2L	V-set and transmembrane domain containing 2 like
TC1900006537.hg.1	-1.8	0.0446	REEP6	receptor accessory protein 6
TC0800006439.hg.1	-1.81	0.039	ZNF596	zinc finger protein 596
TC1700012345.hg.1	-1.82	0.0379	CTDNEP1	CTD nuclear envelope phosphatase 1
TC0100012889.hg.1	-1.83	0.0354	MAD2L2	MAD2 mitotic arrest deficient-like 2 (yeast)
TC0100011665.hg.1	-1.84	0.0304	MARC1	mitochondrial amidoxime reducing component 1

TC0400009037.hg.1	-1.85	0.0041	MND1	meiotic nuclear divisions 1
TC0600011376.hg.1	-1.86	0.0282	PPP1R18	protein phosphatase 1, regulatory subunit 18
TC0800012423.hg.1	-1.87	0.027	PPP1R42	protein phosphatase 1, regulatory subunit 42
TC0500013064.hg.1	-1.88	0.0308	MGAT4B	mannosyl (alpha-1,3-)-glycoprotein beta-1,4-N-acetylglucosaminyltransferase, isozyme B
TC1700010802.hg.1	-1.89	0.001	MPP2	membrane protein, palmitoylated 2
TC1300009570.hg.1	-1.9	0.0228	STK24	serine/threonine kinase 24
TC1000011973.hg.1	-1.92	0.0111	PRLHR	prolactin releasing hormone receptor
TC1700012295.hg.1	-1.92	0.0268	ARSG	arylsulfatase G
TC0100018207.hg.1	-1.93	0.0122	TSSK3	testis-specific serine kinase 3
TC1600009962.hg.1	-1.95	0.0114	TBC1D10B	TBC1 domain family, member 10B
TC0600012329.hg.1	-1.95	0.0375	IMPG1	interphotoreceptor matrix proteoglycan 1
TC1700008440.hg.1	-1.97	0.0004	PPM1E	protein phosphatase, Mg2+/Mn2+ dependent, 1E
TC1200012258.hg.1	-1.97	0.0174	PITPNM2	phosphatidylinositol transfer protein, membrane-associated 2
TC1600011554.hg.1	-1.97	0.0467	LCAT	lecithin-cholesterol acyltransferase
TC0100012942.hg.1	-1.99	0.0474	PRAMEF18	PRAME family member 18
TC0500013143.hg.1	-2	0.0075	EXOC3	exocyst complex component 3
TC0700008340.hg.1	-2	0.0108	GNGT1	guanine nucleotide binding protein (G protein), gamma transducing activity polypeptide 1
TC0600013204.hg.1	-2.01	0.0046	VNN3	vanin 3
TC0200011362.hg.1	-2.03	0.0184	RNPEPL1	arginyl aminopeptidase (aminopeptidase B)-like 1
TC0X00010468.hg.1	-2.03	0.022	FRMPD3-AS1	FRMPD3 antisense RNA 1

TC0900006872.hg.1	-2.05	0.0202	LOC100506422; RP11-80I3.1; tayamu	putative deoxyuridine 5-triphosphate nucleotidohydrolase-like protein FLJ16323; novel transcript; Transcript Identified by AceView
TC0500013355.hg.1	-2.06	0.0075	P4HA2	prolyl 4-hydroxylase, alpha polypeptide II
TC1700008326.hg.1	-2.09	0.0212	TOM1L1	target of myb1 like 1 membrane trafficking protein
TC0900007129.hg.1	-2.11	0.0005	MELK	maternal embryonic leucine zipper kinase
TC1500007753.hg.1	-2.11	0.0028	THSD4	Transcript Identified by AceView, Entrez Gene ID(s) 79875
TC1100013045.hg.1	-2.13	0.0024	SIPA1	signal-induced proliferation-associated 1
TC0900011259.hg.1	-2.13	0.0208	HDHD3	haloacid dehalogenase-like hydrolase domain containing 3
TC0300011951.hg.1	-2.14	0.0015	MORC1	MORC family CW-type zinc finger 1
TC2000007015.hg.1	-2.18	0.0166	PYGB	phosphorylase, glycogen; brain
TC1600010498.hg.1	-2.2	0.047	SLC38A7	solute carrier family 38, member 7
TC1000010276.hg.1	-2.26	0.0243	NRP1	Transcript Identified by AceView, Entrez Gene ID(s) 8829
TC1900009297.hg.1	-2.27	0.016	TBXA2R	thromboxane A2 receptor
TC1400010603.hg.1	-2.28	0.0025	TTC6	tetratricopeptide repeat domain 6
TC0X00011259.hg.1	-2.31	0.0346	STS	steroid sulfatase (microsomal), isozyme S
TC0700010015.hg.1	-2.32	0.0445	INTS1	integrator complex subunit 1
TC1900010720.hg.1	-2.42	0.025	C19orf54	chromosome 19 open reading frame 54
TC0400007055.hg.1	-2.49	0.0048	CDC42	Transcript Identified by AceView, Entrez Gene ID(s) 998
TC0400008134.hg.1	-2.49	0.009	MMRN1	multimerin 1
TC1500008225.hg.1	-2.72	0.0104	MRPS11	mitochondrial ribosomal protein S11
TC0700012995.hg.1	-2.8	0.0148	ZNF777	zinc finger protein 777
TC2200008186.hg.1	-2.83	0.0005	CHCHD10	coiled-coil-helix-coiled-coil-helix domain containing 10
TC1600008928.hg.1	-2.94	0.0487	NPRL3	NPR3-like, GATOR1 complex subunit
