# Supplementary material for: Dynamic integration of CXCL8 or CCL20 signaling with IL-15 or IL-32α reprograms human T-cell functional plasticity
Source: Front Immunol. 2026 Aug 18;17:1910983. doi: 10.3389/fimmu.2026.1910983 (PMC13529521; doi:10.3389/fimmu.2026.1910983)
Supplement: Supplementary file 2 [file Image1.pdf]

Supplementary Figures

Figure S1 – Gate strategy for all analysis performed in the manuscript.

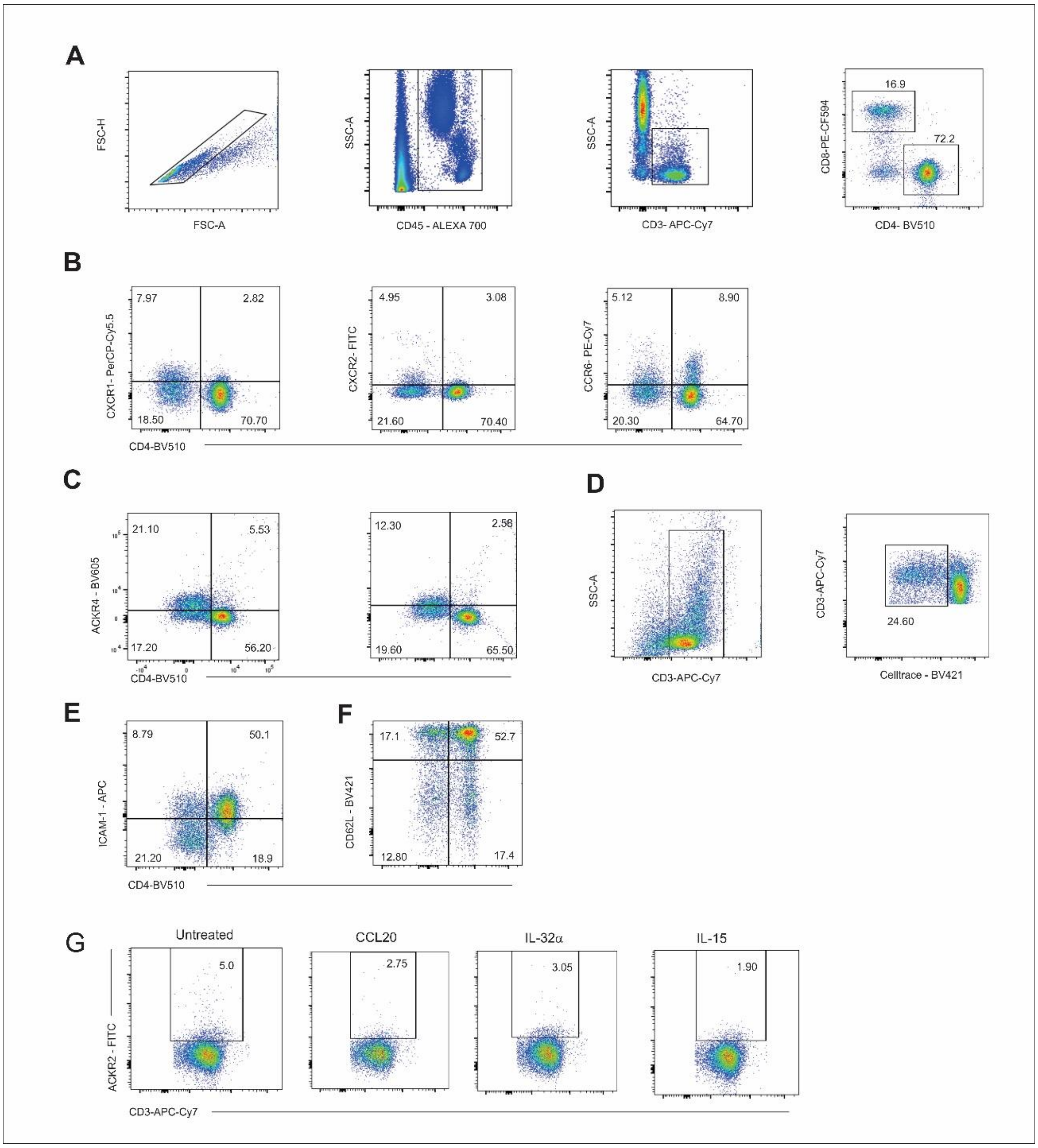

Figure S1: Gate strategy. (A) Gate strategy for Analysis performed I T cell, initial exclusion of doublets, followed by CD45⁺ cells (leukocytes selection), next the CD3⁺ (T cells) selection and identification of CD4+ and CD8+ (T cells subsets); **(B)** Gating on CD3⁺, and following gate on the receptors CXCR1, CXCR2, and CCR6 on T cells subsets CD4⁺ and CD4⁻ (CD8) T cells, **(C)** Gate on ACKR4 on CD3⁺ T cells (Treated and untreated cells), **(D)** Gate on CD3⁺ T cells followed by gating on proliferating T cells (decreasing cell trace), **(E)** Gating on CD3⁺, and following gate on ICAM1 and subsets CD4⁺ and CD4⁻ (CD8) T cells, **(F)** Gating on CD3⁺, and following gate on CD62L and subsets CD4⁺ and CD4⁻ (CD8) T cells **(G)** Gate on ACKR2 on CD3⁺ T cells (Treated and untreated cells).

Figure S2 – Representative of Cytometry flow charts of CD3⁺ (CD4 and CD8) T cells expression of CCR6, CXCR1 and CXCR2 treated and untread with CCL20 and CXCL8.

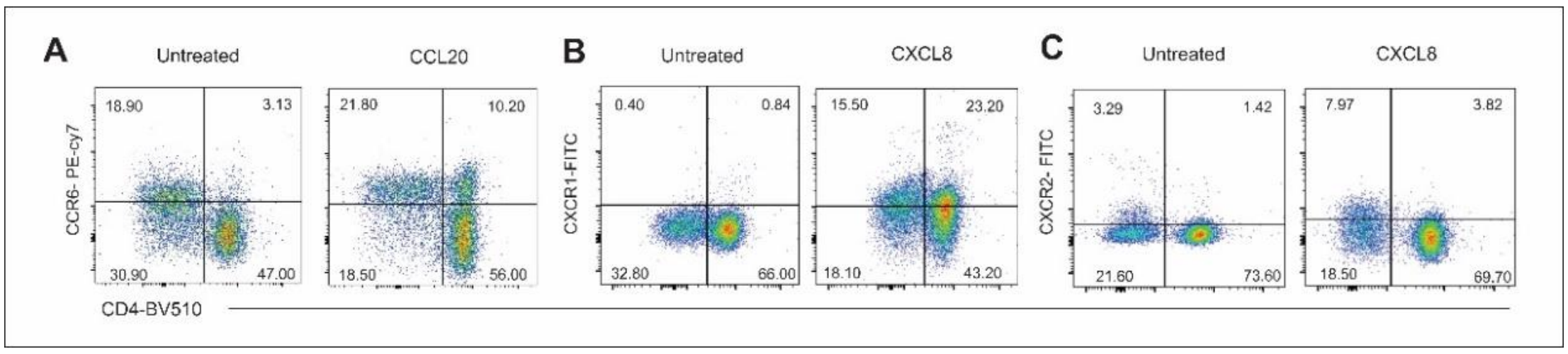

Figure S2: Expression of CCR6, CXCR1 and CXCR2 in T cells. (A) Flow cytometry charts of CCR6 expression on T cells untreated and treated with CCL20, **(B)** Flow cytometry charts of CXCR1 expression on T cells untreated and treated with CXCL8 and, **(C)** Flow cytometry charts of CXC21 expression on T cells untreated and treated with CXCL8 (n=3).

Figure S3 - Flow Cytometry Flow charts representation of CD8⁺ T cells expression of CD62L treated and untread T cells.

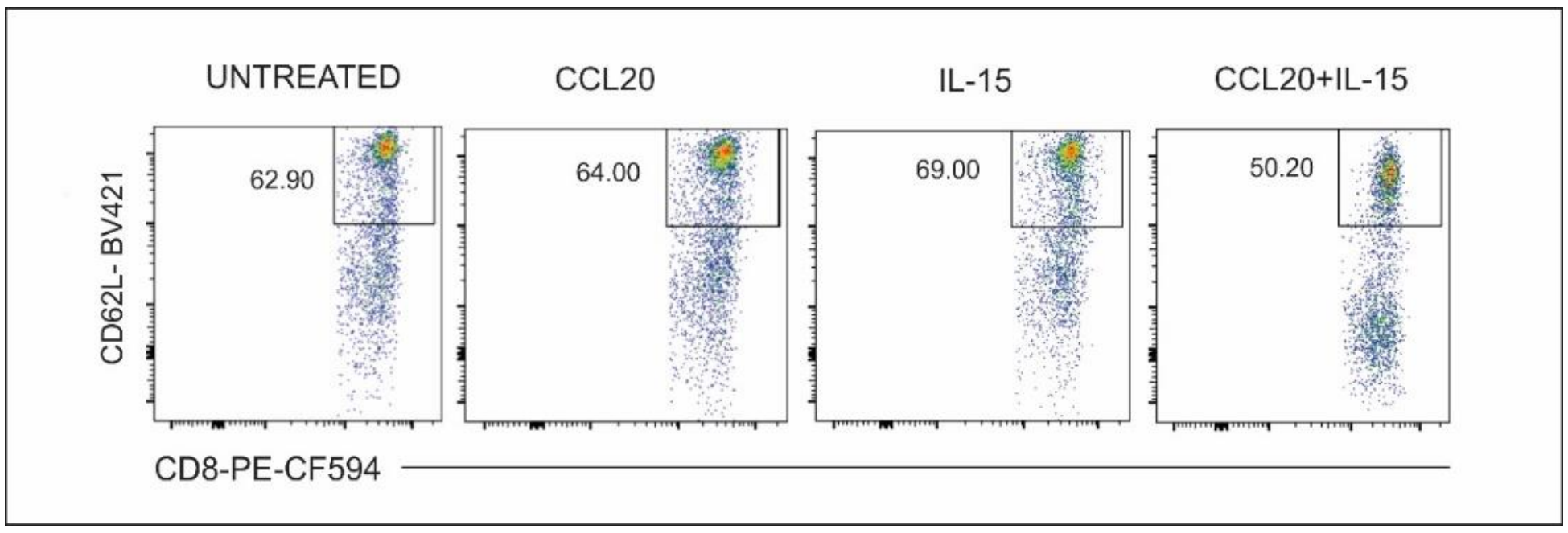

Figure S3: Expression of CD62L in CD8⁺ T cells. (A) Flow cytometry charts of CD62L expression on CD8⁺ T cells untreated and treated with CCL20, or IL-15 or its combination IL-15 with CCL20 (n=3).
