# Supplementary material for: Dynamic integration of CXCL8 or CCL20 signaling with IL-15 or IL-32α reprograms human T-cell functional plasticity
Source: Front Immunol. 2026 Aug 18;17:1910983. doi: 10.3389/fimmu.2026.1910983 (PMC13529521; doi:10.3389/fimmu.2026.1910983)
Supplement: Supplementary file 3 [file Table1.pdf]

Enriched KEGG Pathways Associated with Gene Expression Profiles of CCL20-, CXCL8-, IL-32α-, and IL-15–Treated T Cells

(A) KEGG Pathway for CCL20_treated T cells

Term	PValue	Genes
GO:0019373~epoxygenase P450 pathway	0.002661	CYP2J2, CYP2C9, CYP2A7, CYP2C19
GO:0008202~steroid metabolic process	0.004616	CYP2C9, CYP3A7-CYP3A51P, G6PC, CYP2C19, CYP3A7
GO:0030212~hyaluronan metabolic process	0.014753	ITIH3, IL15, ITIH1
GO:0060347~heart trabecula formation	0.019925	BMP10, ADAMTS1, SRF
GO:0006939~smooth muscle contraction	0.032146	PDE4D, MYH11, NMUR1
GO:0006805~xenobiotic metabolic process	0.034871	CYP2J2, CYP2C9, CYP3A7-CYP3A51P, CYP2C19, CYP3A7
GO:0032094~response to food	0.042784	SLC16A1, G6PC, SLC25A25
GO:0045987~positive regulation of smooth muscle contraction	0.042784	TBXA2R, SRF, NPY2R
GO:0008544~epidermis development	0.045512	SPRR2G, FGF7, FLOT2, LAMC2, SPRR2B
GO:0008306~associative learning	0.054532	GABRA5, SRF, RIN1
GO:1902476~chloride transmembrane transport	0.059651	GABRA1, SLC12A3, GABRA5, CLCA2, CLCNKB
GO:0046596~regulation of viral entry into host cell	0.061652	TRIM62, HS3ST5
GO:0017144~drug metabolic process	0.067264	CYP2C9, CYP2C19, FMO5
GO:0007275~multicellular organism development	0.071219	FIGLA, EBF3, SPATA6, DKK2, PAX2, NPAP1, MN1, HHAT, TXNDC2, ACKR2, QRICH1, MORC1, CYLC1, TCP11
GO:0007229~integrin-mediated signaling pathway	0.071614	IBSP, ADAMTS1, ADAM11, MYH9, ITGB6
GO:0000387~spliceosomal snRNP assembly	0.071707	SNRPD2, GEMIN7, SNRPC
GO:0050911~detection of chemical stimulus involved in sensory perception of smell	0.074855	OR5AU1, OR10J4, OR5M3, OR4C12, OR1J2, OR1K1, OR6A2, OR5D16, OR13C2, OR5D14, OR5T2, OR8H3
GO:0001662~behavioral fear response	0.076242	GABRA5, NPY2R, LYPD1
GO:0000281~mitotic cytokinesis	0.076211	ANLN, CENPA, CEP55
GO:0000212~meiotic spindle organization	0.076464	MYH9, AURKA
GO:0007186~G-protein coupled receptor signaling pathway	0.077003	OR5AU1, OR10J4, OR5M3, OR1J2, OR1K1, TAS2R38, CXCL13, CELSR2, TBXA2R, GPR182, OR4C12, RAPGEF2, NMUR1, ACKR2, OR6A2, OR5D16, OR13C2, OR5D14, NTSR1, OR8H3, RGS7
GO:0002933~lipid hydroxylation	0.091043	CYP3A7-CYP3A51P, CYP3A7
GO:0016098~monoterpenoid metabolic process	0.091043	CYP2C9, CYP2C19
GO:0050808~synapse organization	0.095225	DRP2, LRRTM4, PAK3

(B) KEGG Pathway for CXCL8_treated T cells

Term	PValue	Genes
GO:0030154~cell differentiation	0.013572	NPAP1, OSR2, ADAM18, CSRP2, TSPY8, HIST1H1T, DAZ4, DAZ3, DAZ2, MORC1, TSPY2, DLK1
GO:0007601~visual perception	0.024061	OCLM, LUM, CABP4, SAG, NR2E3, GUCY2F, IMPG1
GO:0070328~triglyceride homeostasis	0.032995	CETP, GCKR, MIA2
GO:0042384~cilium assembly	0.048344	C10ORF90, IFT74, TMEM17, RFX3, FLNA
GO:0007283~spermatogenesis	0.06362	NPAP1, ADAM18, TSPY8, HIST1H1T, DAZ4, DAZ3, DAZ2, MORC1, TSPY2
GO:1902476~chloride transmembrane transport	0.083343	GABRP, GABRA1, CLCNKA, CLIC2
GO:0007506~gonadal mesoderm development	0.08483	TSPY8, TSPY2
GO:0007409~axonogenesis	0.093972	EPYC, LUM, OGN, CTNNA2
GO:0006942~regulation of striated muscle contraction	0.094917	MYL3, MYBPH

(C) KEGG Partway for IL-32α_treated T cells

Term	PValue	Genes
GO:0001946~lymphangiogenesis	0.006888	ACVRL1, FOXC2, PDPN
GO:0033630~positive regulation of cell adhesion mediated by integrin	0.00962	FOXC2, TGFB2, RAC3
GO:0050829~defense response to Gram-negative bacterium	0.026017	FCN2, PRB3, DEFB1, BPI
GO:0032148~activation of protein kinase B activity	0.036326	NTRK3, GAS6, DYNAP
GO:0007155~cell adhesion	0.087976	ISLR, CDH4, LAMA2, PCDHB6, SORBS2, PGM5, GAS6, THBS4, CNTNAP5, EPHA3

GO:0032825~positive regulation of natural killer cell differentiation	0.08923	IL15, GAS6
GO:0050778~positive regulation of immune response	0.099811	TGFB2, IL15

(D) KEGG Partway for IL-15_treated T cells

Term	PValue	Genes
GO:0006163~purine nucleotide metabolic process	0.001785	NME1-NME2, GUK1, NME2, NME1
GO:0015949~nucleobase-containing small molecule interconversion	0.003589	NME1-NME2, GUK1, NME2, TXN, NME1
GO:0002762~negative regulation of myeloid leukocyte differentiation	0.003846	NME1-NME2, NME2, NME1
GO:0072162~metanephric mesenchymal cell differentiation	0.0063	STAT1, PAX2, WNT4
GO:0031424~keratinization	0.007671	SPRR4, CDC42, SPRR2G, KRT17, ABCA12, SPRR2D
GO:2000811~negative regulation of anoikis	0.008847	CEACAM5, BCL2, ITGA5, BCL2L1
GO:0000722~telomere maintenance via recombination	0.008856	PRIM2, POLA1, FEN1, POLD2, SMC6
GO:0033209~tumor necrosis factor-mediated signaling pathway	0.011403	PSMD14, STAT1, TNFRSF9, PSMC1, LTA, PSME2, TNF, TNFRSF4, COMMD7
GO:0090305~nucleic acid phosphodiester bond hydrolysis	0.012806	POLA1, FEN1, PGAP1, TREX2, ENPP3, SND1, REXO4
GO:1901998~toxin transport	0.013386	CD274, NRP1, SCFD1, VPS11, CCT5
GO:0014911~positive regulation of smooth muscle cell migration	0.014009	HDAC4, NRP1, SEMA6D, BCL2
GO:2001240~negative regulation of extrinsic apoptotic signaling pathway in absence of ligand	0.01471	UNC5B, EYA2, BCL2, TNF, BCL2L1
GO:2000378~negative regulation of reactive oxygen species metabolic process	0.016037	BCL2, VDAC1, PAX2, SIRT3
GO:0045668~negative regulation of osteoblast differentiation	0.0176	HDAC4, CDK6, NOG, TNF, HDAC7
GO:0050679~positive regulation of epithelial cell proliferation	0.019062	NME1-NME2, OSR2, NOG, NME2, PAX2, NME1
GO:0006220~pyrimidine nucleotide metabolic process	0.021182	NME1-NME2, NME2, NME1
GO:0007613~memory	0.02167	SLC8A3, FEN1, ITGA8, ITPR3, ITGA5, SHANK3
GO:0043966~histone H3 acetylation	0.024379	KAT2B, YEATS2, IRF4, ELP4, TAF5L
GO:0006259~DNA metabolic process	0.028526	TREX2, TK1, KPNA2, NME1
GO:0007275~multicellular organism development	0.030057	TXNRD3, EYA2, TNFRSF9, PMCH, CREM, DAZ4, EBF3, DAZ2, PAX2, PIM1, SLC26A8, HIVEP1, TXNDC2, ACKR2, TSSK3, MORC1, EVI5, TNFRSF4, ZNF267, TCP11, APOL2, WNT4
GO:0006600~creatine metabolic process	0.03128	GHR, CKMT2, CKMT1A
GO:0071230~cellular response to amino acid stimulus	0.032541	SOCS1, COL4A1, COL4A6, TNF, BCL2L1
GO:0006241~CTP biosynthetic process	0.036907	NME1-NME2, NME2, NME1
GO:0006851~mitochondrial calcium ion transport	0.036907	SLC8A3, VDAC1, SLC8B1
GO:0051560~mitochondrial calcium ion homeostasis	0.036907	SLC8A3, C19ORF12, SLC8B1
GO:0050777~negative regulation of immune response	0.036907	INPP5D, IL2RA, FOXP3
GO:0006228~UTP biosynthetic process	0.036907	NME1-NME2, NME2, NME1
GO:0006183~GTP biosynthetic process	0.042887	NME1-NME2, NME2, NME1
GO:0043069~negative regulation of programmed cell death	0.042887	MECOM, DCPS, PAX2
GO:0071333~cellular response to glucose stimulus	0.044736	NME1-NME2, ENDOG, NME2, PAX2, NME1
GO:0006270~DNA replication initiation	0.048635	PURA, PRIM2, POLA1, MCM2
GO:0007259~JAK-STAT cascade	0.048635	GHR, SOCS2, SOCS1, STAT1
GO:0006820~anion transport	0.049199	SLC26A8, VDAC1, SLC26A3
GO:0009142~nucleoside triphosphate biosynthetic process	0.049199	NME1-NME2, NME2, NME1
GO:0002876~positive regulation of chronic inflammatory response to antigenic stimulus	0.050909	LTA, TNF
GO:0046898~response to cycloheximide	0.050909	GHR, BCL2L1
GO:0070555~response to interleukin-1	0.052511	GHR, HDAC4, SLC30A8, IL1R1
GO:0002931~response to ischemia	0.052511	PANX1, CAMK2A, BCL2, HYOU1
GO:0007608~sensory perception of smell	0.054023	OR8G1, OR8B8, OR2M7, CNGA2, OR2L2, OR51B4, OR52A1, BBS4, OR13C4
GO:0006271~DNA strand elongation involved in DNA replication	0.055821	PRIM2, POLA1, POLD2
GO:0045892~negative regulation of transcription, DNA-templated	0.059118	HDAC4, PRDM5, CREM, ENO1, TNF, COMMD7, FOXP3, PAX2, IKZF4, PURA, RBM15B, PRAMEF18, YEATS2, MECOM, MDFIC, IRF1, BHLHE40, FEZF1, TNFRSF4, WNT4
GO:0040018~positive regulation of multicellular organism growth	0.060692	GHR, VIL1, BCL2, BBS4

GO:0048240~sperm capacitation	0.062735	BSPH1, SLC26A8, SLC26A3
GO:2001234~negative regulation of apoptotic signaling pathway	0.062735	NOG, BCL2, WNT4
GO:0030198~extracellular matrix organization	0.068363	ITGA4, COL4A1, LUM, COL7A1, ITGA8, COL4A6, DMP1, ITGA5, TNF, FBLN5
GO:0043433~negative regulation of sequence-specific DNA binding transcription factor activity	0.068853	HDAC4, BHLHE40, PIM1, TNFRSF4, FOXP3
GO:0016266~O-glycan processing	0.068853	MUC2, GALNT1, MUC4, GALNT8, B4GALT5
GO:0010921~regulation of phosphatase activity	0.075385	PPP1R17, PPP1R42
GO:0001838~embryonic epithelial tube formation	0.075385	SHANK3, WNT4
GO:0002361~CD4-positive, CD25-positive, alpha-beta regulatory T cell differentiation	0.075385	PLA2G2D, FOXP3
GO:0048854~brain morphogenesis	0.077358	SHANK3, PAX2, BBS4
GO:0019373~epoxygenase P450 pathway	0.077358	CYP2J2, CYP2A7, CYP2C19
GO:0006796~phosphate-containing compound metabolic process	0.077358	PPA2, INPP5D, ENPP3
GO:0000302~response to reactive oxygen species	0.078699	PRDX1, TXN, PRDX6, S100A7
GO:0006955~immune response	0.07962	IL32, CD274, CR2, VIPR1, CXCL9, IL1R1, IL15, TNFRSF9, IL2RG, TNF, RGS1, IL2RA, LTA, TNFSF8, ACKR2, ENPP3, TNFRSF4
GO:0006821~chloride transport	0.083526	FXYD3, SLC26A8, NMUR1, SLC26A3
GO:0007155~cell adhesion	0.086064	IL32, SRPX, SPON1, PTPRS, ITGA4, NME2, DPT, BYSL, NME1-NME2, CDH4, CEACAM1, MMRN1, COL7A1, ITGA8, COL4A6, AGGF1, PCDHB3, ITGA5
GO:0007186~G-protein coupled receptor signaling pathway	0.088963	TAS2R7, OR8G1, OR2C1, VIPR1, CXCL9, CCL3L1, OR2M7, PTGER2, CCL3L3, ITPR3, OR4S2, OR8B8, GNG4, RGS1, GPR171, PRLHR, OR8D4, OR51B4, PRLH, GPR156, OR5D14, OR5H1, OR1J2, OR2L2, TAAR3, OR6V1, OR4A8, NMUR1, ACKR2, OR52A1, OR13C4
GO:1902476~chloride transmembrane transport	0.092435	GABRP, FXYD3, SLC26A8, SLC4A3, APOL1, SLC26A3
GO:0008283~cell proliferation	0.092985	ALK, FBXW8, TXN, BYSL, POLA1, IL2RA, PRDX1, PDPN, PIM1, BCL2, TNFSF8, FAM83D, EVI5, SIPA1, BCL2L1
GO:2000741~positive regulation of mesenchymal stem cell differentiation	0.099231	LTBP3, SOX5
GO:0010638~positive regulation of organelle organization	0.099231	SURF4, ANK1
GO:0045682~regulation of epidermis development	0.099231	NME1-NME2, NME2
GO:0051835~positive regulation of synapse structural plasticity	0.099231	CDC42, SHANK3
GO:0038110~interleukin-2-mediated signaling pathway	0.099231	IL2RA, IL2RG
GO:0002925~positive regulation of humoral immune response mediated by circulating immunoglobulin	0.099231	LTA, TNF

(E) KEGG Partway for IL-15_CCL20_treated T cells

Term	PValue	Genes
GO:0033209~tumor necrosis factor-mediated signaling pathway	0.001138	PSMB6, PSMD7, PSMD14, STAT1, TNFRSF9, PSME2, TNF, TNFRSF4, COMMD7, PSMB8
GO:0036498~IRE1-mediated unfolded protein response	0.001776	SEC61A1, XBP1, FKBP14, DNAJB11, HYOU1, SEC61B, PDIA6
GO:0006954~inflammatory response	0.004177	GBP5, IL22, HDAC5, CXCL9, CCL3L1, PTGER2, CCL3L3, TNFRSF9, CXCL13, TNF, BMP2, NCR3, IL2RA, CHST1, IL17F, ACKR2, TNFRSF4, IL17A
GO:0006955~immune response	0.005349	IL32, CD274, IL22, XBP1, CXCL9, IL1R1, TNFRSF9, OSM, CXCL13, TNF, NCR3, RGS1, IL2RA, CCR8, TNFSF8, ACKR2, TNFRSF4, CTSC, IL17A
GO:0034097~response to cytokine	0.005435	CD274, AANAT, STAT1, BCL2, BCL2L1, MCL1
GO:2000811~negative regulation of anoikis	0.005607	BCL2, ITGA5, BCL2L1, MCL1
GO:0045663~positive regulation of myoblast differentiation	0.010282	CXCL9, MYF6, FLT3LG, CDON
GO:0006915~apoptotic process	0.013354	RALB, STAT1, TNFRSF9, PDCD5, CIDEB, USP17L2, DUX4, THOC6, VIL1, CASP7, MELK, NUAK2, IL2RA, IRF1, INPP5D, PIM1, BCL2, TNFSF8, BIRC7, S100A14, CTSC, IL17A
GO:0008283~cell proliferation	0.014953	PRMT5, OSM, TXN, EGFR, ZFP36L2, BYSL, MELK, IL2RA, PRDX1, PDPN, PIM1, BCL2, TNFSF8, SIPA1, CDK5R1, BCL2L1
GO:1900017~positive regulation of cytokine production involved in inflammatory response	0.015493	GBP5, IL17F, IL17A
GO:1902902~negative regulation of autophagosome assembly	0.019089	SCFD1, EHMT2, MTM1
GO:0071230~cellular response to amino acid stimulus	0.019131	XBP1, SOCS1, TNF, EGFR, BCL2L1
GO:0008630~intrinsic apoptotic signaling pathway in response to DNA damage	0.019131	BCL2, CIDEB, TNF, BCL2L1, MCL1
GO:0070098~chemokine-mediated signaling pathway	0.019492	CXCL9, CCL3L1, CCL3L3, CCR8, ACKR2, CXCL13

GO:0001934~positive regulation of protein phosphorylation	0.021575	MAVS, XBP1, RAP2A, BMP2, RALB, FLT3LG, TNF, EGFR
GO:0007267~cell-cell signaling	0.024402	IL22, CRB1, BMP2, CXCL9, FSHB, CD80, SEMA3B, TNFSF8, CARTPT, TXN, CXCL13, IL17A
GO:0001662~behavioral fear response	0.024854	FBXL20, GRM7, BCL2, LYPD1
GO:0006521~regulation of cellular amino acid metabolic process	0.025055	PSMB6, PSMD7, PSMD14, PSME2, PSMB8
GO:0006869~lipid transport	0.025347	OSBPL7, OSBPL6, APOL6, APOL1, SORL1, APOL2
GO:0045892~negative regulation of transcription, DNA-templated	0.026473	HDAC5, PRAMEF20, PBXIP1, ENO1, TNF, ZBTB4, COMMD7, FOXP3, DKK3, PER1, BMP2, YEATS2, MDFIC, IRF1, BHLHE40, PRAMEF25, MYF6, TNFRSF4, CDK5R1
GO:0050777~negative regulation of immune response	0.027204	INPP5D, IL2RA, FOXP3
GO:0031145~anaphase-promoting complex-dependent catabolic process	0.029347	PSMB6, PSMD7, PSMD14, PSME2, PSMB8, AURKA
GO:0061029~eyelid development in camera-type eye	0.031693	OSR2, EGFR, STRA6
GO:0048546~digestive tract morphogenesis	0.031693	BCL2, EGFR, STRA6
GO:0045579~positive regulation of B cell differentiation	0.036449	XBP1, INPP5D, PRDM1
GO:0051284~positive regulation of sequestering of calcium ion	0.043235	RYR2, FKBP1B
GO:0042981~regulation of apoptotic process	0.044996	WRN, STAT1, GLS2, TNFRSF9, QRICH1, USP17L2, TNFRSF4, BCL2L1, MCL1, BMP15
GO:0007565~female pregnancy	0.045447	HPGD, EPYC, FSHB, BCL2, PSG2, BYSL
GO:2001240~negative regulation of extrinsic apoptotic signaling pathway in absence of ligand	0.046557	BCL2, TNF, BCL2L1, MCL1
GO:0045786~negative regulation of cell cycle	0.046557	BMP2, CDK6, HPGD, SIPA1
GO:0002479~antigen processing and presentation of exogenous peptide antigen via MHC class I, TAP-dependent	0.048851	PSMB6, PSMD7, PSMD14, PSME2, PSMB8
GO:0030335~positive regulation of cell migration	0.049611	VIL1, SEMA6C, CD274, XBP1, BMP2, FSHB, SEMA3B, ITGA5, EGFR
GO:1901741~positive regulation of myoblast fusion	0.052194	CXCL9, MYF6, FLT3LG
GO:2000778~positive regulation of interleukin-6 secretion	0.052194	XBP1, IL17F, IL17A
GO:0008285~negative regulation of cell proliferation	0.053642	WARS, CCL3L1, CCL3L3, TNFRSF9, FBXO2, PDCD5, OSM, DPT, CDC6, DUX4, FOXP3, BMP2, CDK6, IRF1, FRK
GO:0038061~NIK/NF-kappaB signaling	0.05623	PSMB6, PSMD7, PSMD14, PSME2, PSMB8
GO:0006006~glucose metabolic process	0.058815	PDHA1, KCNJ11, BPGM, TNF, BRS3
GO:0006811~ion transport	0.06019	GABRP, GABRA1, SLC12A5, TMC7, HTR3A, SLC8B1, CNNM3
GO:0008637~apoptotic mitochondrial changes	0.063792	PPP2CB, BCL2L1, MCL1
GO:0006469~negative regulation of protein kinase activity	0.065929	SOCS2, CEACAM1, SOCS1, CISH, GNAQ, PPM1E
GO:0007275~multicellular organism development	0.067091	GCM2, TNFRSF9, OSM, PBXIP1, TRERF1, DKK3, PIM1, HIVEP1, ACKR2, QRICH1, TSSK3, MORC1, TNFRSF4, ZNF267, TCP11, HOXA4, APOL2, MCL1
GO:1904837~beta-catenin-TCF complex assembly	0.067293	PYGO2, HIST1H4B, HIST1H4F, BCL9L
GO:0000413~protein peptidyl-prolyl isomerization	0.067293	PPIL1, FKBP14, FKBP1B, FKBP6
GO:0071347~cellular response to interleukin-1	0.069778	CCL3L1, CCL3L3, TANK, MYLK3, IL17A
GO:0051436~negative regulation of ubiquitin-protein ligase activity involved in mitotic cell cycle	0.069778	PSMB6, PSMD7, PSMD14, PSME2, PSMB8
GO:0098609~cell-cell adhesion	0.07504	PSMB6, CEACAM1, BAIAP2L1, DIAPH3, STAT1, PRDX1, ENO1, MB21D2, PRDX6, PAICS, PFKP
GO:0043488~regulation of mRNA stability	0.075352	PSMB6, PSMD7, PSMD14, PSME2, PSMB8, ZFP36L2
GO:0006886~intracellular protein transport	0.07554	RIMS2, AP3M2, SEC23A, ADPRHL1, SGSM3, VPS11, TMED2, SNX33, SEC61B, ARFGAP3
GO:0090305~nucleic acid phosphodiester bond hydrolysis	0.081713	WRN, PGAP1, REXO1, TREX2, RAD9A
GO:2001243~negative regulation of intrinsic apoptotic signaling pathway	0.082603	BCL2, BCL2L1, MCL1
GO:0009267~cellular response to starvation	0.083064	WRN, RALB, EHMT2, CARTPT
GO:0031064~negative regulation of histone deacetylation	0.084605	USP17L2, FOXP3
GO:0042063~gliogenesis	0.084605	GCM2, CDK6
GO:2000065~negative regulation of cortisol biosynthetic process	0.084605	BMP2, DKK3
GO:0032348~negative regulation of aldosterone biosynthetic process	0.084605	BMP2, DKK3
GO:0051437~positive regulation of ubiquitin-protein ligase activity involved in regulation of mitotic cell cycle transition	0.084845	PSMB6, PSMD7, PSMD14, PSME2, PSMB8
GO:0045944~positive regulation of transcription from RNA polymerase II promoter	0.09016	GCM2, TOP2A, HDAC5, TNF, DUX4, EGFR, MYBL1, BCL9L, CDON, OSR2, XBP1, PCGF5, STAT1, FSHB, OSM, FLT3LG, FOXP3, PER1, PYGO2, BMP2, MAVS, IRF4, IRF1, MYF6, TCF4, IL17F, TNFSF8, NEUROG1, IL17A

GO:0034341~response to interferon-gamma	0.095961	SEC61A1, GCH1, UBD
GO:0051149~positive regulation of muscle cell differentiation	0.095961	MYF6, MAPK12, CDON

(F) KEGG Partway for IL-32α_CCL20_treated T cells

Term	PValue	Genes
GO:0007155~cell adhesion	0.001739	LAMB4, OMD, DPT, LAMC2, DSPP, AJAP1, COMP, CDH4, ISLR, IBSP, ITGA10, CNTNAP3B, ITGA7, PCDHB3, GAS6, CNTNAP5, EPHA3
GO:0031424~keratinization	0.005905	CDC42, SPRR2G, LCE6A, SFN, ABCA12
GO:0030154~cell differentiation	0.010924	NANOS2, TXNRD3, DAPL1, HIST1H1T, TSPY2, DLK1, CSRP2, HLX, SNAPC4, NHS, CSPG5, MORC1, GPM6B, TCP11, FKBP6
GO:0051893~regulation of focal adhesion assembly	0.026691	GREM1, GPM6B, EPHA3
GO:0007275~multicellular organism development	0.027904	NANOS2, TXNRD3, WNT5B, HIST1H1T, DKK2, H1FNT, DSPP, HHAT, CSRP2, HLX, C19ORF57, ACKR2, PDILT, MORC1, TCP11
GO:0030198~extracellular matrix organization	0.029633	COMP, C6ORF15, IBSP, LUM, ITGA10, ITGA7, LAMC2, DSPP
GO:0007268~chemical synaptic transmission	0.029765	GABRR2, DOC2A, NPY2R, HTR2C, GRIK1, KCNQ5, PCDHB3, PCDHB10, OR5T2
GO:0043171~peptide catabolic process	0.043159	ENPEP, ADAMTS13, LVRN
GO:2000345~regulation of hepatocyte proliferation	0.044698	GLI1, SULF2
GO:0030201~heparan sulfate proteoglycan metabolic process	0.044698	HS3ST4, SULF2
GO:0007229~integrin-mediated signaling pathway	0.063336	ADAMTS13, IBSP, ITGA10, ADAM11, ITGA7
GO:0007283~spermatogenesis	0.068506	NANOS2, TXNRD3, PRKACG, HIST1H1T, MORC1, CDC25C, TSPY2, GLI1, FKBP6, TCP11, SOX30
GO:0001662~behavioral fear response	0.070842	NPY2R, LYPD1, HTR2C
GO:0036336~dendritic cell migration	0.073386	CDC42, NLRP10
GO:0021696~cerebellar cortex morphogenesis	0.073386	RFX4, GLI1
GO:0033138~positive regulation of peptidyl-serine phosphorylation	0.089645	CDC42, BCAR3, NTRK3, GAS6

(G) KEGG Partway for IL-15_CXCL8_treated T cells

Term	PValue	Genes
GO:0033209~tumor necrosis factor-mediated signaling pathway	8.71E-05	PSMD8, PSMA3, PSMD14, STAT1, PSMB3, TNFRSF9, PSMC1, LTA, PSME1, PSME2, TNF, TNFRSF4
GO:0006521~regulation of cellular amino acid metabolic process	1.56E-04	PSMD8, NQO1, PSMA3, PSMD14, PSMB3, PSMC1, PSME1, PSME2
GO:0050852~T cell receptor signaling pathway	1.61E-04	PSMD14, PIK3R1, FOXP3, PSMD8, PSMA3, IFNG, PSMB3, PSMC1, INPP5D, UBE2N, PSME1, PSME2, TAB2
GO:0010950~positive regulation of endopeptidase activity	9.13E-04	SFRP2, PSMD14, PSME1, PSME2
GO:0006915~apoptotic process	0.001484	RALB, APIP, COMP, INPP5D, RPS6KA1, PIM1, C19ORF12, S100A14, LGALS7, LGALS7B, STAT1, TNFRSF9, CIDEB, THOC6, IL1A, SFRP2, IFNG, IRF1, IL2RA, LTA, BCL2, TNFSF8, VDAC1, BIRC7, MCM2, IL17A
GO:0050777~negative regulation of immune response	0.002272	INPP5D, IL2RA, SPINK5, FOXP3
GO:0002223~stimulatory C-type lectin receptor signaling pathway	0.00291	PSMD8, PSMA3, PSMD14, PSMB3, PSMC1, UBE2N, PSME1, PSME2, TAB2
GO:0002479~antigen processing and presentation of exogenous peptide antigen via MHC class I, TAP-dependent	0.003197	PSMD8, PSMA3, PSMD14, PSMB3, PSMC1, PSME1, PSME2
GO:0038061~NIK/NF-kappaB signaling	0.004037	PSMD8, PSMA3, PSMD14, PSMB3, PSMC1, PSME1, PSME2
GO:0006955~immune response	0.004119	IL32, GBP6, CD274, IL22, XBP1, VIPR1, CXCL9, IL1R1, TNFRSF9, TNF, IL1A, IFNG, RGS1, IL2RA, LTA, CCR8, TNFSF8, ACKR2, TNFRSF4, IL17A
GO:0060071~Wnt signaling pathway, planar cell polarity pathway	0.005279	PSMD8, VANGL1, PSMA3, PSMD14, PSMB3, PSMC1, PSME1, PSME2
GO:0051436~negative regulation of ubiquitin-protein ligase activity involved in mitotic cell cycle	0.005785	PSMD8, PSMA3, PSMD14, PSMB3, PSMC1, PSME1, PSME2
GO:0045892~negative regulation of transcription, DNA-templated	0.005877	KLF10, HDAC5, THRA, NR1D1, ENO1, TNF, ZBTB4, FOXP3, IKZF4, RBM15B, SFRP2, SALL1, PRAMEF18, YEATS2, MDFIC, IRF1, BHLHE40, ZNF280C, ID3, FEZF1, TNFRSF4, CDK5R1
GO:2000811~negative regulation of anoikis	0.006449	CEACAM5, BCL2, ITGA5, BCL2L1
GO:2000778~positive regulation of interleukin-6 secretion	0.006449	XBP1, NLRP10, IL17F, IL17A
GO:0051437~positive regulation of ubiquitin-protein ligase activity involved in regulation of mitotic cell cycle transition	0.008028	PSMD8, PSMA3, PSMD14, PSMB3, PSMC1, PSME1, PSME2
GO:0045672~positive regulation of osteoclast differentiation	0.008884	KLF10, IFNG, TNF, IL17A
GO:0031145~anaphase-promoting complex-dependent catabolic process	0.009642	PSMD8, PSMA3, PSMD14, PSMB3, PSMC1, PSME1, PSME2

GO:0043488~regulation of mRNA stability	0.009648	PSMD8, PSMA3, PSMD14, PSMB3, PSMC1, PSME1, PSME2, ZFP36L2
GO:0007268~chemical synaptic transmission	0.009752	GRIA1, VIPR1, DOC2A, PMCH, NPY2R, SLC6A11, PCDHB10, MPZ, LRFN1, PCDHB6, PLP1, PCDHB3, SPG11
GO:2001240~negative regulation of extrinsic apoptotic signaling pathway in absence of ligand	0.009979	IL1A, BCL2, TNF, MAP2K5, BCL2L1
GO:0060396~growth hormone receptor signaling pathway	0.013464	SOCS2, PXN, PIK3R1
GO:1900017~positive regulation of cytokine production involved in inflammatory response	0.01705	GBP5, IL17F, IL17A
GO:0007275~multicellular organism development	0.018324	TNFRSF9, PMCH, HIST1H1T, DAZ3, PRM3, VANGL1, HHAT, SFRP2, CSRP2, PIM1, HIVEP1, C19ORF57, ID3, ACKR2, TSSK3, DPPA5, MORC1, TNFRSF4, ZNF267, KCTD15, APOL2
GO:0090263~positive regulation of canonical Wnt signaling pathway	0.020884	PSMD8, PSMA3, SFRP2, PSMD14, PSMB3, PSMC1, PSME1, PSME2
GO:0038095~Fc-epsilon receptor signaling pathway	0.02211	PSMD8, PSMA3, PSMD14, PSMB3, PSMC1, UBE2N, PSME1, PSME2, TAB2, PIK3R1
GO:0008630~intrinsic apoptotic signaling pathway in response to DNA damage	0.02255	BCL2, CIDEB, PIK3R1, TNF, BCL2L1
GO:0033077~T cell differentiation in thymus	0.023431	CDK6, DNAJA3, BCL2, ZFP36L2
GO:0016339~calcium-dependent cell-cell adhesion via plasma membrane cell adhesion molecules	0.025806	PCDHB6, PCDHB3, CDH17, PCDHB10
GO:0001662~behavioral fear response	0.028306	FBXL20, NPY2R, BCL2, VDAC1
GO:0006954~inflammatory response	0.029426	GBP5, IL22, HDAC5, CXCL9, CCL3L1, PTGER2, CCL3L3, TNFRSF9, TNF, IL1A, IL2RA, PLP1, IL17F, ACKR2, TNFRSF4, IL17A
GO:0032693~negative regulation of interleukin-10 production	0.029872	CD274, FOXP3, IDO1
GO:0032869~cellular response to insulin stimulus	0.032132	HDAC5, XBP1, CEACAM1, STAT1, PIK3R1, CPEB2
GO:0060334~regulation of interferon-gamma-mediated signaling pathway	0.034775	SOCS1, IFNG, STAT1
GO:0007155~cell adhesion	0.036855	IL32, SPON1, ITGA4, PXN, HAPLN3, BYSL, COMP, CEACAM1, PDZD2, CHL1, CNTNAP3B, COL6A2, PCDHB6, CCR8, PCDHB3, ITGA5, CDH17, CNTNAP5
GO:0045579~positive regulation of B cell differentiation	0.039966	XBP1, INPP5D, PRDM1
GO:0043161~proteasome-mediated ubiquitin-dependent protein catabolic process	0.045258	PSMD8, ABTB1, PPP2CB, PSMA3, C18ORF25, PSMD14, PSMB3, PSMC1, PSME1, PSME2
GO:0006198~cAMP catabolic process	0.045427	PDE1A, PDE4C, PDE7A
GO:0002876~positive regulation of chronic inflammatory response to antigenic stimulus	0.045447	LTA, TNF
GO:0060557~positive regulation of vitamin D biosynthetic process	0.045447	IFNG, TNF
GO:0046967~cytosol to ER transport	0.045447	TAP2, TAP1
GO:0043433~negative regulation of sequence-specific DNA binding transcription factor activity	0.049015	BHLHE40, PIM1, ID3, TNFRSF4, FOXP3
GO:0061136~regulation of proteasomal protein catabolic process	0.051145	PSMD14, PSME1, PSME2
GO:2000045~regulation of G1/S transition of mitotic cell cycle	0.051145	PLK5, PSME1, PSME2
GO:0044130~negative regulation of growth of symbiont in host	0.051145	IFNG, LTA, TNF
GO:0010832~negative regulation of myotube differentiation	0.051145	HDAC5, XBP1, CEACAM5
GO:0033365~protein localization to organelle	0.051145	CEP68, BBS4, CNGB1
GO:0007565~female pregnancy	0.054255	HPGD, EPYC, PAPPA, BCL2, BYSL, IDO1
GO:0008625~extrinsic apoptotic signaling pathway via death domain receptors	0.056261	TNFRSF9, BCL2, PIK3R1, TNF
GO:0042157~lipoprotein metabolic process	0.056261	APOL6, LPA, APOL1, APOL2
GO:0000302~response to reactive oxygen species	0.05995	GPX2, PRDX1, TXN, PRDX6
GO:0043066~negative regulation of apoptotic process	0.061774	NQO1, XBP1, APIP, PIK3R1, THOC6, SOCS2, COMP, PRAMEF18, CCND2, DNAJA3, MPZ, CEACAM5, RPS6KA1, PIM1, BCL2, BIRC7, BCL2L1
GO:0000209~protein polyubiquitination	0.062372	PSMD8, PSMA3, C18ORF25, PSMD14, PSMB3, PSMC1, BCL2, PSME1, PSME2
GO:0061512~protein localization to cilium	0.063291	SNX10, ATP6V1D, BBS4
GO:0001895~retina homeostasis	0.06375	PRDX1, LCN1, BBS4, CNGB1
GO:0042384~cilium assembly	0.066607	C10ORF90, SPAG16, DNAH5, SNX10, CFAP54, ATP6V1D, BBS4
GO:2000391~positive regulation of neutrophil extravasation	0.067392	IL1A, IL1R1
GO:0060559~positive regulation of calcidiol 1-monooxygenase activity	0.067392	IFNG, TNF
GO:0070859~positive regulation of bile acid biosynthetic process	0.067392	STARD4, NR1D1
GO:0072092~ureteric bud invasion	0.067392	SALL1, FMN1
GO:0018345~protein palmitoylation	0.076294	HHAT, ZDHHC22, ZDHHC3
GO:0008283~cell proliferation	0.080512	KLF10, TXN, GRPR, ZFP36L2, BYSL, IL1A, IL2RA, PRDX1, PIM1, BCL2, TNFSF8, SIPA1, CDK5R1, BCL2L1

GO:0070098~chemokine-mediated signaling pathway	0.080618	CXCL9, CCL3L1, CCL3L3, CCR8, ACKR2
GO:0060333~interferon-gamma-mediated signaling pathway	0.080618	IFNG, STAT1, IRF4, IRF1, GBP1
GO:0090090~negative regulation of canonical Wnt signaling pathway	0.082627	PSMD8, PSMA3, SFRP2, PSMD14, PSMB3, PSMC1, PSME1, PSME2
GO:2000378~negative regulation of reactive oxygen species metabolic process	0.083084	BCL2, VDAC1, PLIN5
GO:0002925~positive regulation of humoral immune response mediated by circulating immunoglobulin	0.088834	LTA, TNF
GO:0010638~positive regulation of organelle organization	0.088834	SURF4, ANK1
GO:0031999~negative regulation of fatty acid beta-oxidation	0.088834	ACACB, PLIN5
GO:0046006~regulation of activated T cell proliferation	0.088834	CD274, IDO1
GO:0071230~cellular response to amino acid stimulus	0.093266	XBP1, SOCS1, TNF, BCL2L1
GO:0006869~lipid transport	0.09763	APOL6, LPA, ABCA12, APOL1, APOL2

(H) KEGG Partway for IL-32_CXCL8_treated T cells

Term	PValue	Genes
GO:1901998~toxin transport	0.015703	NRP1, SCFD1, VPS11, DNM1
GO:0000086~G2/M transition of mitotic cell cycle	0.051947	MELK, HAUS7, CHEK2, KDM8, TAF2, AURKA
GO:0070328~triglyceride homeostasis	0.055573	ACSM2A, MIA2, APOA5
GO:0019932~second-messenger-mediated signaling	0.057557	DGKD, TBXA2R
GO:0051835~positive regulation of synapse structural plasticity	0.057557	CDC42, FRMPD4
GO:0009611~response to wounding	0.06562	NRP1, GRIN2A, POU5F1, AURKA
GO:0042632~cholesterol homeostasis	0.06813	ABCG5, LCAT, MIA2, APOA5
GO:0061551~trigeminal ganglion development	0.071423	NRP1, SIX4
GO:0034504~protein localization to nucleus	0.071616	CTDNEP1, COL1A1, SIX4
GO:0007131~reciprocal meiotic recombination	0.071616	XRCC3, MND1, RAD54B
GO:0006302~double-strand break repair	0.07328	MAD2L2, CHEK2, MND1, NBN
GO:0042177~negative regulation of protein catabolic process	0.080133	MAD2L2, GRIN2A, FLNA
GO:0050808~synapse organization	0.084503	DRP2, LRRTM4, SNCB
GO:0071526~semaphorin-plexin signaling pathway	0.084503	NRP1, SEMA6D, FLNA
GO:0032527~protein exit from endoplasmic reticulum	0.085086	RANGRF, MIA2
GO:0007165~signal transduction	0.089302	MPP2, NRP1, NMB, DGKD, KCNIP3, AIP, STAM, IL2RG, TICAM1, NR2E3, OR52N4, GNGT1, FGF17, TOM1L1, STK24, OR51B6, OR52E6, SRGAP3, PI4KB, OR52A1, ITGB1BP2, SIPA1, OR52A5, TBC1D10B
GO:0050911~detection of chemical stimulus involved in sensory perception of smell	0.098068	OR5AU1, OR2C1, OR11G2, OR51B6, OR52E6, OR52A1, OR52N4, OR5D14, OR52A5, OR4S2, OR5T2
